# 25-hydroxycholesterol promotes proliferation and metastasis of lung adenocarcinoma cells by regulating ERβ/TNFRSF17 axis

**DOI:** 10.1186/s12885-024-12227-4

**Published:** 2024-04-22

**Authors:** Mengting He, Wenbo Jiang, Xingkai Li, Hongjin Liu, Hongsheng Ren, Yanliang Lin

**Affiliations:** 1 Shandong Key Laboratory of Reproductive Medicine, Department of Obstetrics and Gynecology, Department of Reproductive Medicine, Shandong Provincial Hospital Affiliated to Shandong First Medical University, 250021 Jinan, Shandong China; 2 Department of Critical Care Medicine, Shandong University of Traditional Chinese Medicine, 250000 Jinan, Shandong China; 3 Department of Thoracic Surgery, Daqing Longnan Hospital, 163453 Daqing, Heilongjiang China; 4 Department of Thoracic Surgery, National Clinical Research Center for Cancer/Cancer Hospital, National Cancer Center, Chinese Academy of Medical Sciences and Peking Union Medical College, 100021 Beijing, China; 5 Department of Critical Care Medicine, Shandong provincial Hospital Affiliated to Shandong First MedicalUniversity, 250021 Jinan, Shandong China

**Keywords:** Lung adenocarcinoma, 25-hydroxycholesterol, Estrogen receptor beta, TNFRSF17, Metastasis, Proliferation

## Abstract

Lung adenocarcinoma is the main type of lung cancer in women. Our previous findings have evidenced that 25-hydroxycholesterol (25-HC) promotes migration and invasion of lung adenocarcinoma cells (LAC), during which LXR as a 25-HC receptor plays an important role. Estrogen receptor beta (ERβ) is a receptor of 27-hydroxycholesterol that is structurally analogous to 25-HC, but its role in the functional actions of 25-HC remained largely unknown. In this study, we demonstrated that 25-HC treatment triggered ERβ expression in LAC. Knockdown of ERβ inhibited 25-HC-mediated proliferation, migration and invasion, and reduced 25-HC-induced LAC metastasis in vivo. Further investigation revealed that ERβ knockdown restrained the expression of TNFRSF17 (BCMA). In vivo experiments also confirmed that ERβ knockdown blocked 25-HC-induced TNFRSF17 expression. TNFRSF17 knockdown also restrained 25-HC-induced proliferation, migration and invasion. Bioinformatic analysis showed that the levels of ERβ and TNFRSF17 were elevated in lung adenocarcinoma, and were closely related to tumor stages and nodal metastasis status. These results suggested that 25-HC promoted the proliferation and metastasis of LAC by regulating ERβ/TNFRSF17 axis.

**Supplementary Information:**

The online version contains supplementary material available at 10.1186/s12885-024-12227-4.

## Introduction

Lung cancer is the leading cause of cancer-related deaths worldwide, and lung adenocarcinoma (LAC) is the main type of lung cancer, especially in women [1]. The proliferation and metastasis of LAC are still obstructions for its effective therapy. Thus, it is necessary to explore more therapeutic targets for LAC.

The sex difference in susceptibility suggests pivotal roles of hormones in the progression of LAC [2]. The association between early menopause and a decreased risk of LAC has been demonstrated [3], indicating that estrogen is important for LAC, which is confirmed by the evidences that the estrogen treatment slightly increases a risk of LAC [4]. Estrogen accelerates tumor progression at a receptor-dependent manner [5]. Estrogen receptor contains two subtypes, ERα and ERβ, both of which show strong affinities to estradiol [6]. Since their tissue distributions are different, ERβ is specifically expressed in lung tissue [7]. Therefore, the agonists of ERβ might affect the proliferation and metastasis of LAC.

25-hydroxycholesterol (25-HC) is a metabolite of cholesterol catalyzed by cholesterol 25 hydroxylase (CH25H), and regulates cholesterol biosynthesis by inhibiting SREBPs [8]. 25-HC has been involved in innate and adaptive immunity [9]. Meantime, 25-HC also participates in tumorigenesis, and promotes the migration and invasion of lung, gastric, brain and breast cancer cells [10–13]. Our previous findings have demonstrated that 25-HC enhances the migratory capacity of LAC through the LXR signaling [10]. However, knockout of LXR could not completely block the proliferation, migration and invasion of LAC induced by 25-HC. Considering that ERβ is a potential receptor of 25-HC, in this study, we examined the role of ERβ in 25-HC-mediated LAC proliferation and metastasis.

TNFRSF17encoding BCMA is the member of the tumor necrosis factor (TNF) receptor superfamily, and is mainly expressed in mature B lymphocytes [14]. TNFRSF17 has been demonstrated to specifically recognize the member 13b of the TNF superfamily (TNFSF13B/TALL-1/BAFF), activating NF-kappaB and MAPK8/JNK, which in turn supports cell proliferation and migration [15–16]. However, the function of TNFRSF17 in LAC remains unknown. In the present study, we evidenced that 25-HC-induced ERβ positively regulated the expression of TNFRSF17, which was key for 25-HC-mediated LAC proliferation and metastasis.

## Materials and methods

### Materials

The human lung adenocarcinoma cells A549 and SPC-A1 were acquired from the Cell Bank of the Chinese Academy ofSciences(Shanghai, China)0.25-hydroxycholesterol (25-HC) was purchased from Sigma(St.Louis, MO, USA), and dissolved in anhydrous ethanol.

### Cell culture

A549 and SPC-A1 cells were seeded in RPMI 1640 medium (Gibco, NY, USA) containing 10% fetal bovine serum (Gibco),100 µg/ml penicillin(Gibco) and 100 µg/ml streptomycin(Gibco). All cells were cultured at 37^o^C with 5% CO_2_ in a humidified atmosphere, and were treated with the indicated concentrations of 25-HC.

### Cell transfection

Cells were seeded in six-well plates, and were transfected with the lentivirus carrying ESR2-sgRNA(sgRNA1:TGTATATGGAGCCGTGCTCC; sgRNA2:TGTCTGCAGCGATTACGCAT; sgRNA3: CGTTGCGCCAGCCCTGTTAC), NR1H3-sgRNA (sgRNA1: TCGGCTTCGCAAATGCCGTC; sgRNA2: AGCGCCGGTTACACTGTTGC; sgRNA3: CTACATGCGTCGCAAGTGCC) or small interfering RNAs against TNFRSF17(siRNA1:CCACGAAAACGAAUGACUA; siRNA2:CAUGUCAGCGUUAUUGUAA; siRNA3:CUUCGAUGUUCUUCUAAUA). After 72 h of transfection, the collected cells were subjected to further experiments.

### Cell viability assay

A549 and SPC-A1 cells were seeded in 96-well plates, and incubated overnight, followed by treatment with different concentrations of 25-HC for 48 h. The cell viability was determined using a CCK-8 Kit (Dojindo, Kumamoto, Japan)according to the manufacturer’s instructions. Briefly, 10 µL of CCK-8 solution were added to each well and incubated 1 h. The absorbance at 450 nm was detected using the multifunctional microplate reader (Thermo Fisher Scientific, MA, USA).

### Edu proliferation assay

A549 and SPC-A1 cells were incubated in 96-well plates overnight, followed by treatment with the different concentrations of 25-HC for 48 h. The cell proliferation capacity was evaluated using the Cell-light™ EdU Apollo® 567 In Vitro Imaging Kit (Ribobio, Guangzhou, China) according to the manufacturer’s instructions.

### Migration assay

A549 and SPC-A1 cells were seeded in the 6-well plates. After the monolayer cells were formed, a linear wound was created with a sterile 200 ml pipette tip. Cell debris was washed away with PBS and then cultured in complete medium containing different concentrations of 25-HC for 48 h. Images were captured using the olympus microscope (IX53). The linear wound area was calculated using ImageJ software (National Institutes of Health, Bethesda, MD, USA).

### Invasion assay

A549 and SPC-A1 cells in logarithmic growth phase were suspended in serum-free medium and plated into the upper chamber of Transwell (Corning, USA) coating with Matrigel (diluted with RPMI 1640 medium at 4^o^Cat a ratio of 1:8, BD Biosciences, USA). The lower chambers were supplemented with complete medium containing different concentrations of 25-HC.After48 h of incubation, cells on the lower surface were stained with 0.1% crystal violet, and were captured using the olympus microscope (IX53).

### Cell apoptosis assay

After LAC cells were treated with different concentrations of 25-HCfor 48 h, apoptotic cells were tested using the FITC Annexin V Apoptosis Kit(BD, NJ, USA) according to the manufacturer’s instructions.

### Western blot

The cells treated with 25-HC were harvested and lysed in RIPA lysis buffer. Equal amounts of protein samples were loaded onto 10% SDS-PAGE gels. The location of the targeted proteins in gels was cut according to molecular weight, and was electrotransferred to polyvinylidene difluoride membranes (EMD Millipore, Billerica, MA, USA). After blocking with 5% skim milk for 1 h, the membranes were incubated overnight at 4 °C with primary antibodies anti-GAPDH (Proteintech, WuHan, China), 1:5000; anti-ERβ (BIOSS, Beijing, China), 1:1000; anti-TNFRSF17 (Proteintech, WuHan, China),1:1000. After washed with TBST, the membranes were incubated with secondary antibodies and the signal was detected using enhanced chemiluminescence and quantified using ImageJ software.

### qPCR analysis

Total RNA was extracted using TRIzol reagent (Takara, Japan). Then, the RNA was reversely transcribed using the PrimeScript RT Reagent Kit (Takara, Japan).The target and control genes were analyzed via qRT-PCR using SYBR Master Mix (Life Technologies, USA). The relative expression of target genes was calculated using the comparative Ct method formula 2^−ΔΔCt^. GAPDH was used as a control. Primer sequences were presented in Supplementary Table 1.

### Bioinformatics analysis

Bioinformatics analysis was performed on data from the GEO dataset, dataset GSE50081 (127LAC patient samples) downloaded from the Gene Expression Omnibus (GEO, https://www.ncbi.nih.gov/geo/).Based on the median level of ERβ expression, LAC patient samples were divided into ERβ-high and ERβ-low groups. The empirical Bayesian approach was applied to extract differentially expressed genes (DEGs) between the distinct groups. All the DEGs were presented in the volcano plots, and the correlation of some representative DEGs with ERβ was presented in heatmaps. The significance criterion was set as an adjusted *p* value < 0.05 and abs (logFC) > 0.5. According to the results of DEGs, TNFRSF17 was identified as the co-expression gene of ERβ. Then, tumor Immune Estimation Resource 2.0 (TIMER2.0) database (http://timer.cistrome.org/) was used to analyze the correlation between ERβ and TNFRSF17 in LAC. The correlation of gene expression was evaluated by Spearman’s correlation and statistical significance.

UALCAN (http://ualcan.path.uab.edu/index.html) which is an interactive web-based tool to perform analyses of gene expression data from The Cancer Genome Atlas (TCGA). In this study, we employed UALCAN database to analyze the expression of ERβ and TNFRSF17 between normal tissues and LAC tissues. Meanwhile, the UALCAN database was used to explore ERβ and TNFRSF17 gene expression in different pathological stages and N stages of LAC. Kaplan–Meier survival analyses were also developed based on UALCAN database.

### Immunohistochemistry (IHC) staining

Lung tissues were fixed with 4% paraformaldehyde overnight, and were then rehydrated, paraffin embedded, sectioned and dewaxed at room temperature. The sections were blocked with serum, followed by incubation with primary antibodies ERβ and TNFRSF17 overnight at 4 °C. The sections were incubated in the corresponding secondary antibodies for 2 h at room temperature. Finally, the sections were stained with DAB reagent and hematoxylin, and were observed using the olympus microscope (IX53). The expression of ERβ and TNFRSF17 were analyzed using ImageJ software.

### In vivo LAC model

The experimental protocols for mice have been approved by the Institutional Laboratory Animal Care and Use Committee at Shandong provincial hospital. Four-week-old male BALB/c nude mice (SCBS Biotechnology Co., LTD, Henan, China) were intravenously injected with A549 cells, A549-depleting ERβ. Subsequently, different concentrations of25-HC were injected via tail vein every 2 days. After 6 weeks, nude mice were sacrificed and lung tissues were extracted by tracheal perfusion fixation with 4% paraformaldehyde.

### Statistical analysis

All statistical analyses were performed using Graphpad Prism software version 8.0. The data are presented as mean ± SD of at least three independent experiments. The differences between two groups were measured by Student’s t-test. *P* < 0.05 indicates that the difference is statistically significant.

## Results

### 25-hydroxycholesterol promoted ERβ expression

**Fig. 1:**
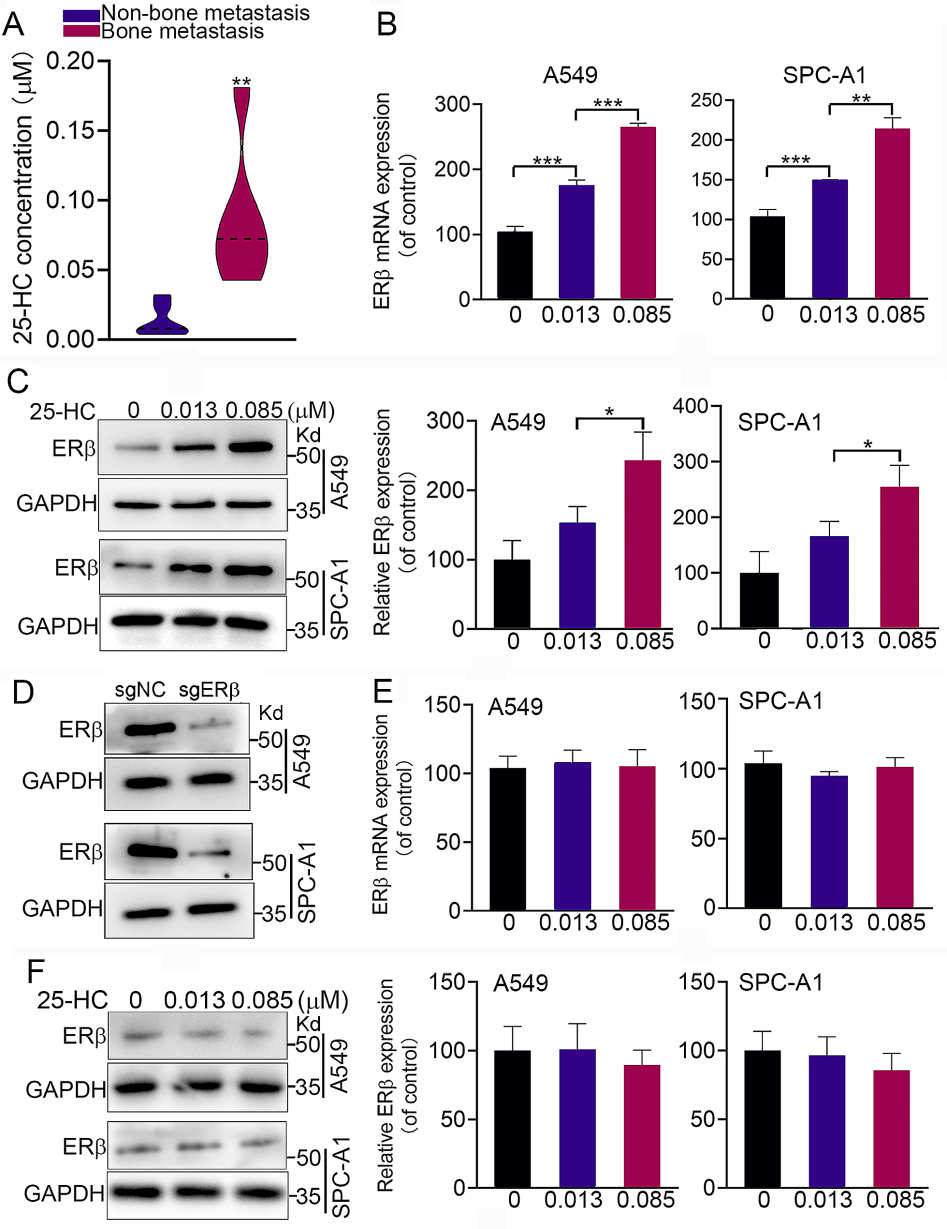
25-hydroxycholesterol induced ERβ expression. (**A**) BALB/c nude mice were intravenously inoculated A549 cells, and feed with normal diet or high cholesterol diet for 8 weeks. The serum levels of 25-hydroxycholesterol (25-HC) in non-bone metastatic and bone metastatic mice were determined using LC-MS method. (**B**) A549 and SPC-A1 cells were exposed to 0, 0.013 and 0.085 µM 25-HC for 48 h. The mRNA expression of ERβ was measured by qPCR analysis. (**C**) A549 and SPC-A1 cells were exposed to 0, 0.013 and 0.085 µM 25-HC for 48 h. The protein expression of ERβ was measured by western blot analysis. (**D**) A549 and SPC-A1 cells were transfected with the lentivirus carrying ESR2-sgRNA, and the protein expression of ERβ was measured by western blot analysis. (**E**) After ERβ knockdown, A549 and SPC-A1 cells were exposed to indicated concentration of 25-HC for 48 h. The protein expression of ERβ was measured by western blot analysis. Data were shown as mean ± SD of at least three independent experiments. *, *p* < 0.05; **, *p* < 0.01; ***, *p* < 0.0001

Since high cholesterol diet enhances LAC metastasis [17], we analyzed the serum levels of cholesterol metabolites. Among the mice with bone metastatic LAC, the serum level of 25-hydroxycholesterol (25-HC) was significantly higher than those in mice with non-bone metastatic LAC (0.085 vs. 0.013 µM, Fig. 1A). Considering that 25-HC might be an agonist of ERβ that plays an important role in LAC proliferation and metastasis, we first examined the different concentrations of 25-HC on the ERβ expression. As shown in Fig. 1B and C, exposure of A549 and SPC cells to 0.013 µM and 0.085 µM 25-HC remarkably increased the mRNA level of ERβ, but only 0.085 µM 25-HC elevated the protein level of ERβ. Knockdown of ERβ by Crispr/Cas9 method significantly reduced the protein expression of ERβ despite in the presence of 0.085 µM 25-HC (Fig. 1D, E and F). Since LXR has been well known as a receptor for 25-HC, we also examined the effect of LXR knockdown on ERβ expression. The results showed that LXR knockdown did not affect 25-HC-induced ERβ expression (Supplementary Fig. 1). These results suggested that 25-HC promoted ERβ expression independent of LXR.

### ERβ knockdown blocked 25-HC-induced proliferation, migration and invasion of LAC

**Fig. 2:**
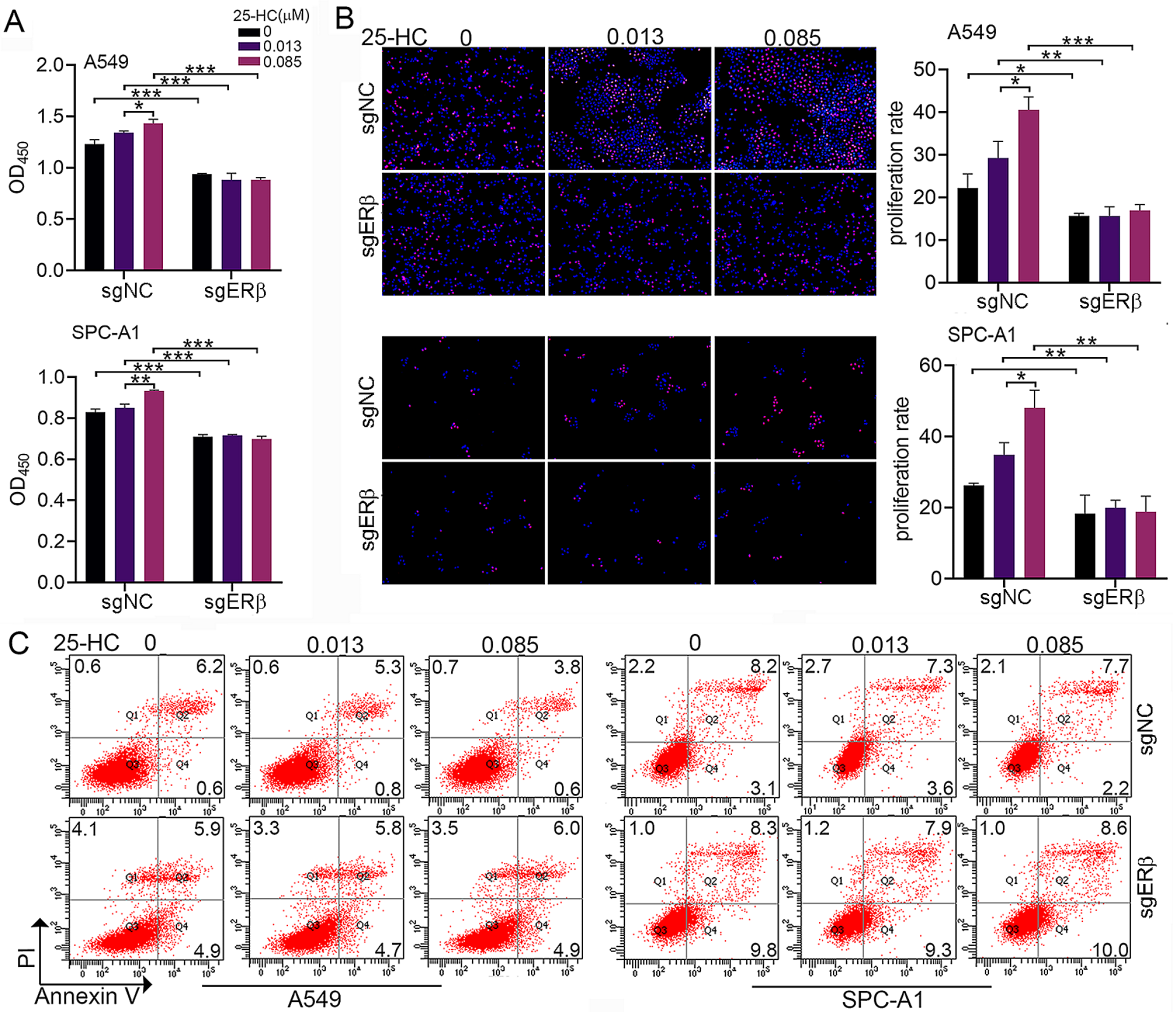
ERβ knockdown inhibited 25-HC-induced cell proliferation. A549 and SPC-A1 cells were transfected with the lentivirus carrying ctrl-sgRNA or ESR2-sgRNA, and were then exposed to 0, 0.013 and 0.085 µM 25-HC for 48 h. Cell proliferation was determined by CCK8 assay (**A**) and Edu proliferation assay (**B**). Cell necrosis and apoptosis were determined by flow cytometry analysis. Data were shown as mean ± SD of at least three independent experiments.*, *p* < 0.05; **, *p* < 0.01;*** *p* < 0.0001

**Fig. 3:**
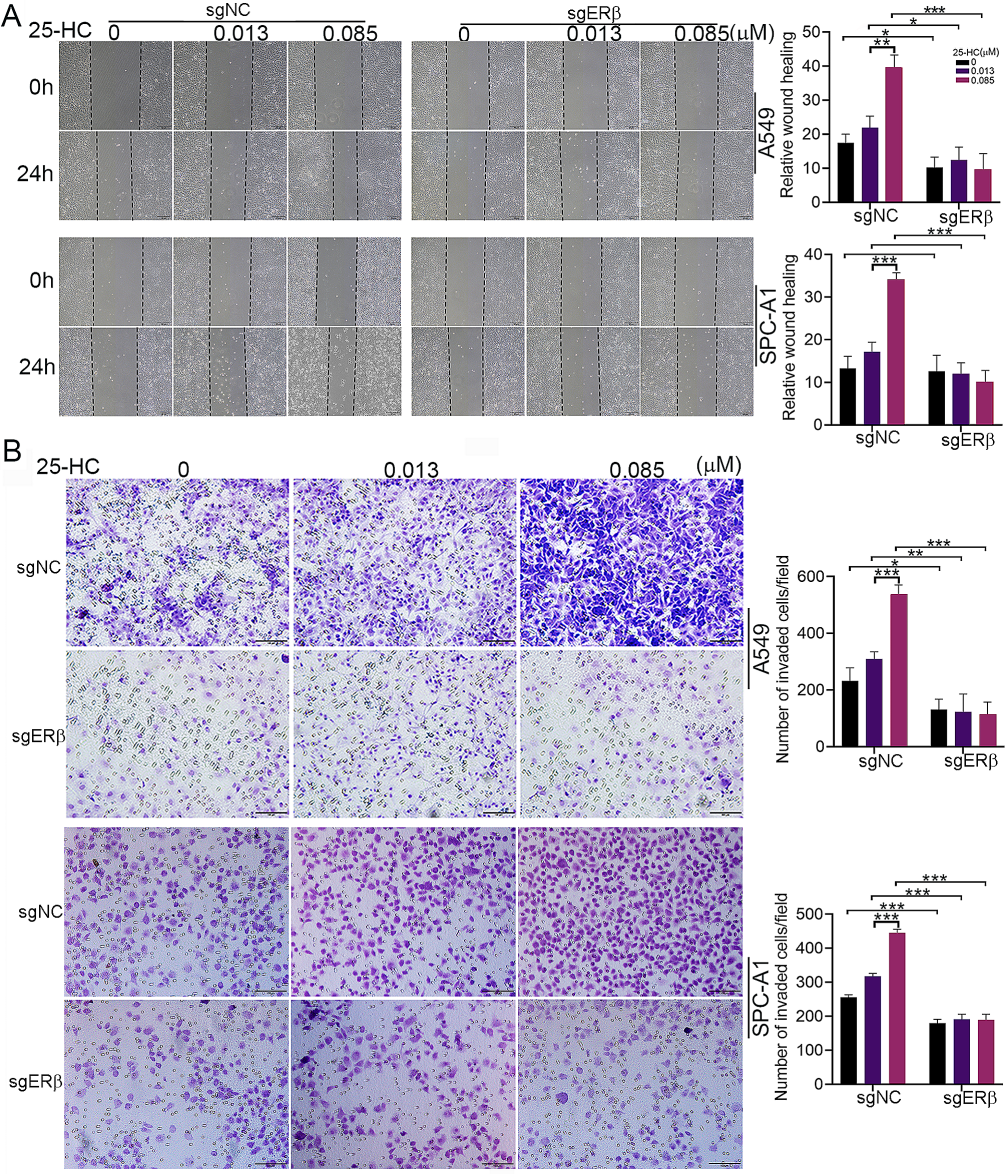
ERβ knockdown inhibited 25-HC-induced cell migration and invasion. (**A**) A549 and SPC-A1 cells were transfected with the lentivirus carrying ctrl-sgRNA or ESR2-sgRNA, followed by treatment with 0, 0.013 and 0.085 µM 25-HC for 48 h. Wound healing assay was performed to determine the effect of 25-HC on cell migration. (**B**) After ERβ knockdown, the cells were exposed to 0, 0.013 and 0.085 µM 25-HC for 48 h. Transwell assay was performed to determine the effect of 25-HC on cell invasion. Data were shown as mean ± SD of at least three independent experiments.*, *p* < 0.05; **, *p* < 0.01;*** *p* < 0.0001

Since we have demonstrated that 25-HC induces proliferation, migration and invasion of LAC [10], we further analyze the role of ERβ in 25-HC-mediated events. CCK8 assay showed that 0.085 µM25-HCslightly facilitated LAC proliferation, which was confirmed by Edu staining (Fig. 2A and B). ERβ knockdown notably blocked 25-HC-induced LAC proliferation (Fig. 2A and B). Flow cytometric analysis illustrated that 25-HCdid not affect apoptosis at concentrations of 0.013 µM and 0.085 µM, while ERβ knockdown promoted cell apoptosis despite in the presence of 0.085 µM 25-HC (Fig. 2C). Wound healing assay revealed that 0.085 µM 25-HC accelerated LAC migration compared to 0.013 µM 25-HC, which was significantly blocked by ERβ knockdown (Fig. 3A). Similarly, Exposure of A549 and SPC-A1 cells to 0.085 µM 25-HC notably enhanced the invasive capacity compared to 0.013 µM 25-HC, which was restrained by ERβ knockdown (Fig. 3B). Furthermore, treatment with 0.085 µM 25-HC remarkably promoted the phosphorylation of AKT and ERK as well as vimentin expression, while inhibited E-Cadherin expression (Supplementary Fig. 2). These results suggested that 25-HC promoted the proliferation, migration and invasion of LAC via ERβ.

### ERβ positively regulated the expression of TNFRSF17

**Fig. 4:**
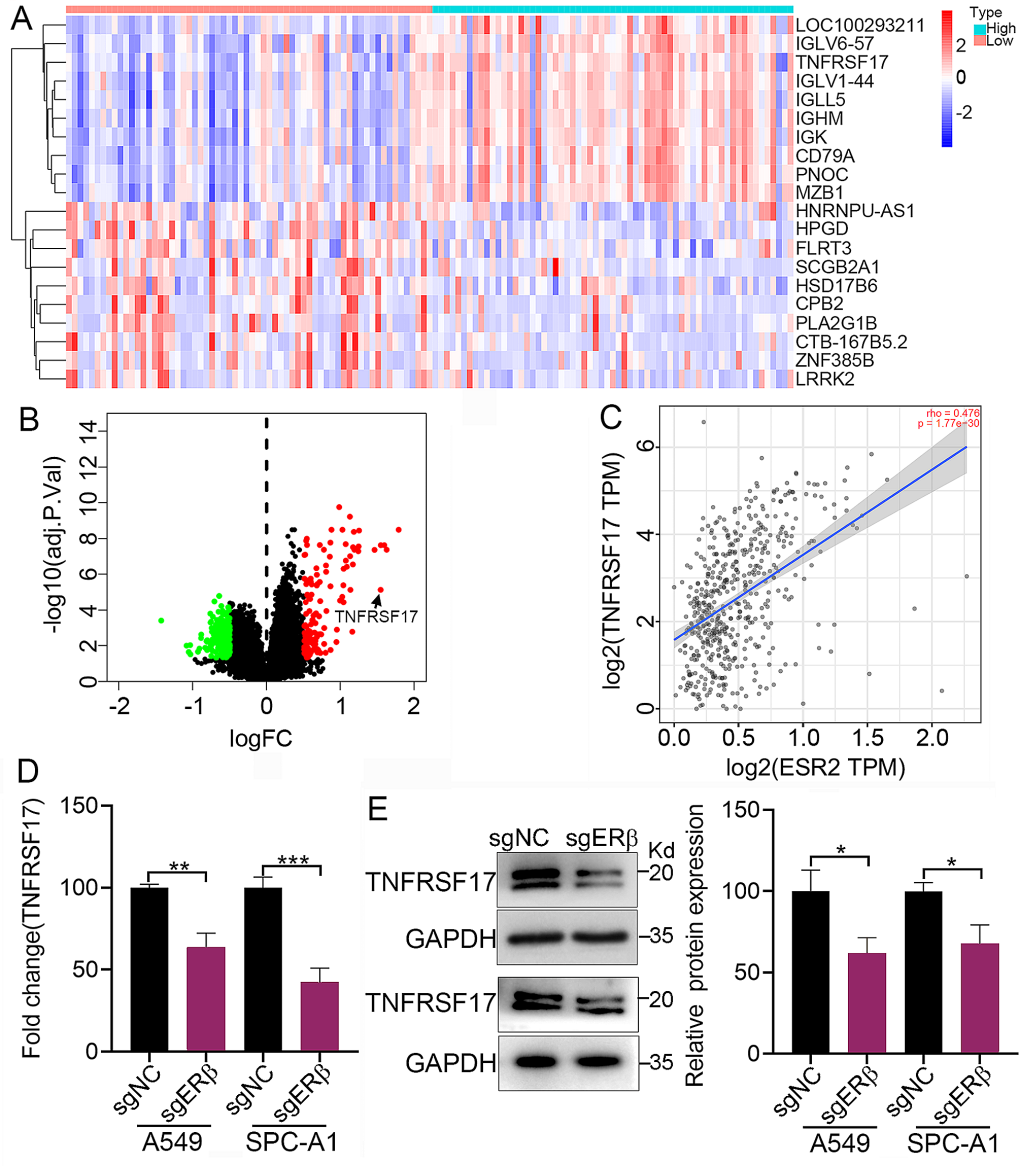
ERβ positively regulated the expression of TNFRSF17. **A**. Differential genes were identified between ERβ-high and ERβ-low groups which were stratified based on the median ERβ expression. Cluster analysis of the top 10 upregulated and downregulated genes. **B**. Volcanic map of differential genes between ERβ-high and ERβ-low groups. **C**. The correlation between the expression of ERβ and TNFRSF17. **D**. The effect of ERβ knockdown on the mRNA expression of TNFRSF17in the presence of 0.085 µM25-HC. **E**. The effect of ERβ knockdown on the protein expression of TNFRSF17 in the presence of 0.085 µM 25-HC.Data were shown as mean ± SD of at least three independent experiments.*, *p* < 0.05; **, *p* < 0.01;*** *p* < 0.0001

Following the above results, we examined the DEGs between ERβ-high and ERβ-low groups which were stratified based on the median ERβ expression. The result demonstrated that 368 mRNAs, containing 138 upregulated and 230 downregulated genes, were differently expressed in ERβ-high groups compared to ERβ-low groups, among which the top 10 genes were shown in Fig. 4A.Relative expression values of the representative DEGs between the two cohorts were shown in the form of volcano plot (Fig. 4B). Then, the TIMER database was applied to explore the correlation between the expression of ERβ and TNFRSF17. The results suggested a positive correlation between the expression of ERβ and TNFRSF17 with a Spearman coefficient value of 0.476 (Fig. 4C). Consistently, ERβ knockdown significantly reduced the mRNA and protein levels of TNFRSF17 (Fig. 4D and E). These results indicated that ERβ positively regulated the expression of TNFRSF17.

### TNFRSF17silencingblocked 25-HC-induced proliferation, migration and invasion of LAC

**Fig. 5:**
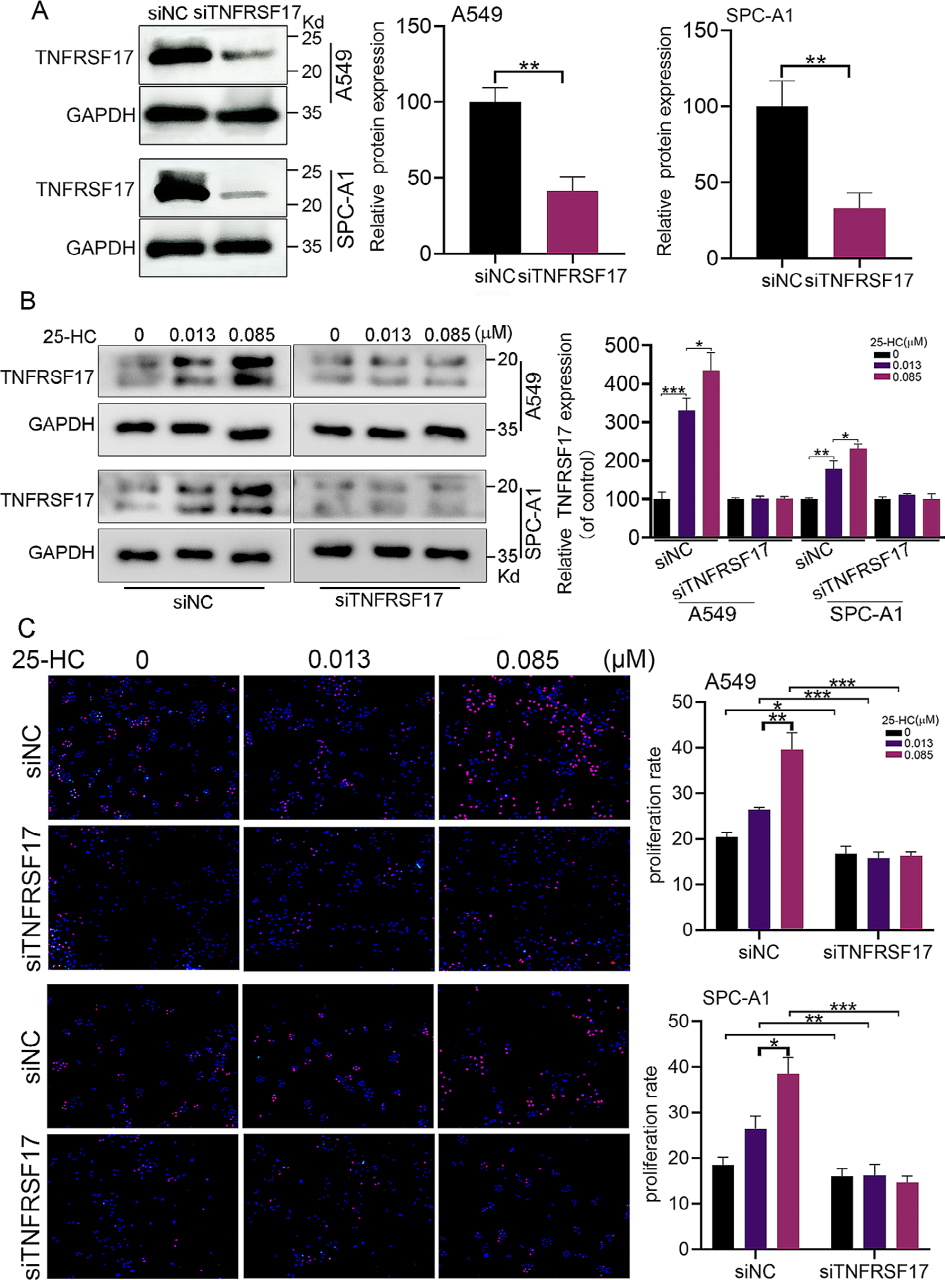
TNFRSF17 silencing inhibited 25-HC-induced LAC proliferation. (**A**) A549 and SPC-A1 cells were transfected with the small interfering RNAs against TNFRSF17 (siTNFRSF17) and the protein expression of TNFRSF17 was determined by western blot analysis. (**B**) A549 and SPC-A1 cells were transfected with siTNFRSF17, followed by treatment with the indicated concentration of 25-HC for 48 h. The protein expression of TNFRSF17 was measured by western blot analysis. (**C**) After TNFRSF17 knockdown, the cells were exposed to 0, 0.013 and 0.085 µM 25-HC for 48 h. Cell proliferation was determined by Edu proliferation assay. Data were shown as mean ± SD of at least three independent experiments.*, *p* < 0.05; **, *p* < 0.01;*** *p* < 0.0001

**Fig. 6:**
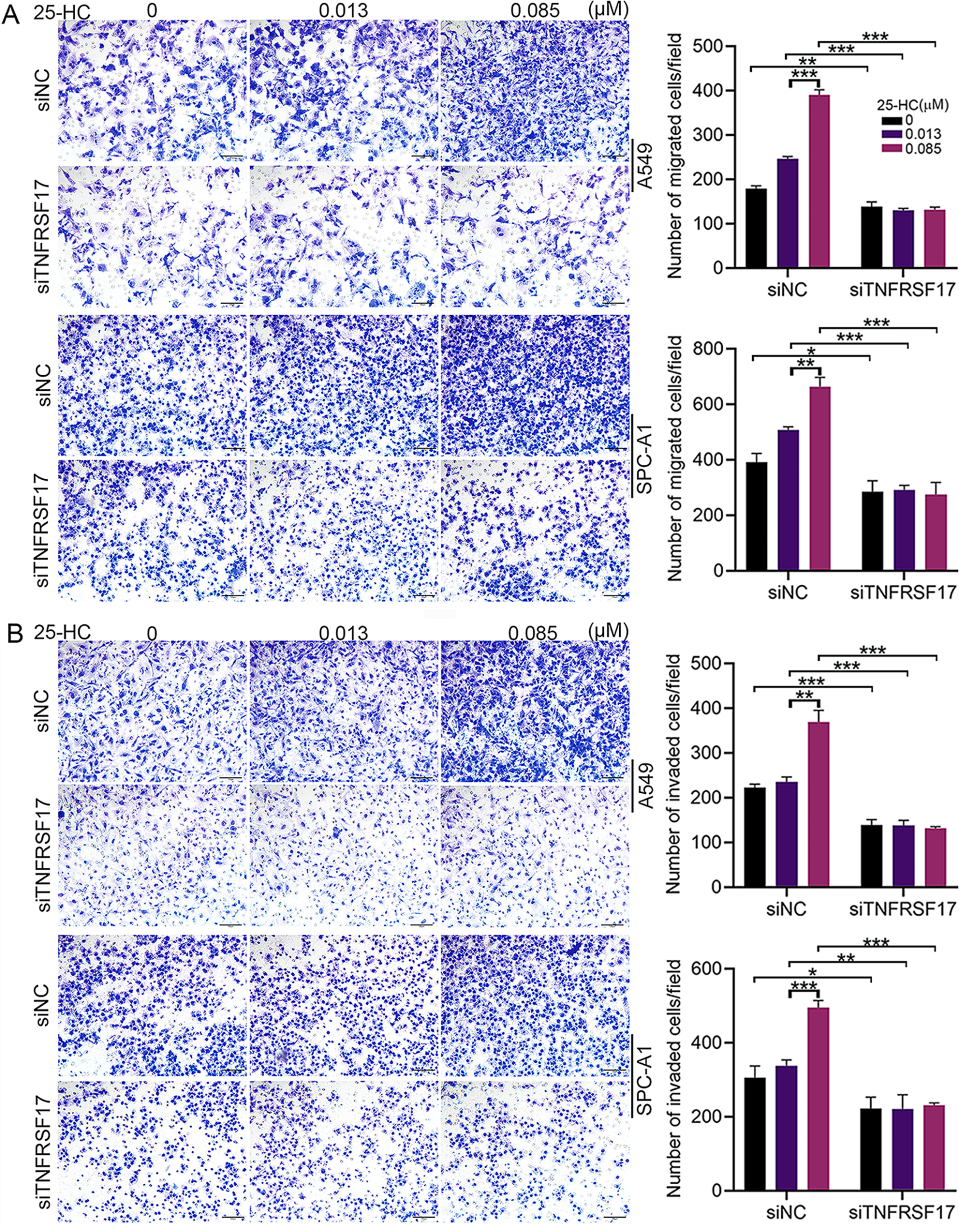
TNFRSF17 silencing inhibited 25-HC-induced cell migration and invasion. A549 and SPC-A1 cells were transfected with the small interfering RNAs carrying siNC or siTNFRSF17, and were then exposed to 0, 0.013 and 0.085 µM 25-HC for 48 h. Cell migration was performed by transwell assay (**A**). Cell invasion was performed by transwell assay (coating with Matrigel) (**B**).Data were shown as mean ± SD of at least three independent experiments.*, *p* < 0.05; **, *p* < 0.01;*** *p* < 0.0001

We further analyzed the role of TNFRSF17 in 25-HC-mediated events.A549 and SPC-A1 cells were transfected with siRNAs against TNFRSF17. The western blot result showed that transfection with siRNA ssignificantly decreased the protein expression of TNFRSF17 (Fig. 5A). Exposure of A549 and SPC-A1 cells to 0.085 µM 25-HC notably elevated the expression of TNFRSF17, which was blocked by TNFRSF17 silencing (Fig. 5B). Edu proliferation assay showed thatTNFRSF17 silencing notably blocked 25-HC-induced LAC proliferation (Fig. 5C). Also, Transwell assay confirmed that TNFRSF17silencinginhibited25-HC-inducedLAC migration (Fig. 6A) and invasion (Fig. 6B). These results suggested that TNFRSF17 expression was required for 25-HC-induced proliferation, migration and invasion.

### ERβ reverses 25-HC-mediatedLAC metastasis in vivo

**Fig. 7:**
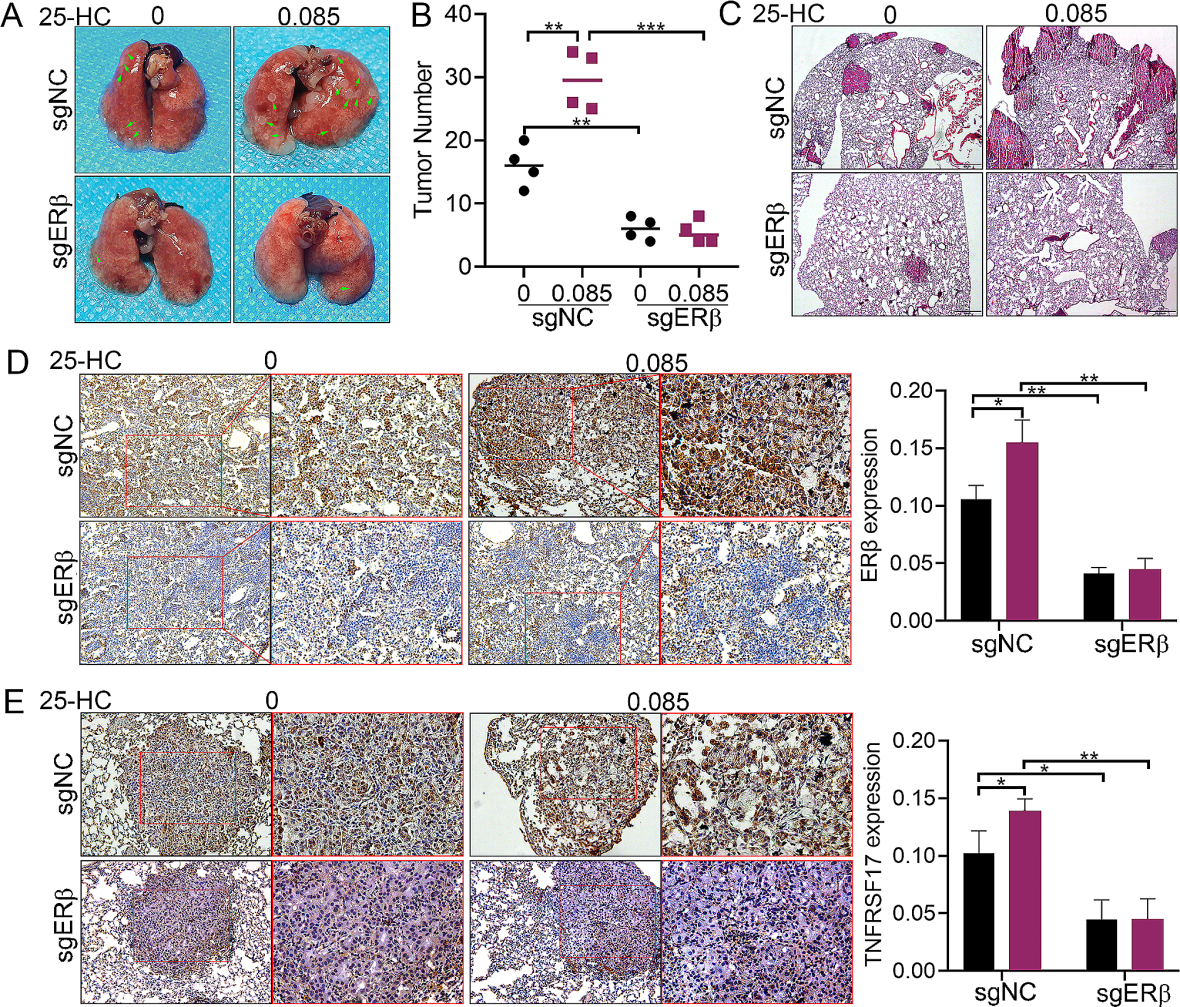
ERβ knockdown reduced 25-HC-mediated LAC metastasis and TNFRSF17 expression. Sixteen male BALB/c nude mice were randomly divided into four groups, and were intravenously injected with A549 cells or A549-depleting ERβ, followed by injecting different concentrations of 25-HC via tail vein every 2 days. After 6 weeks, nude mice were sacrificed and the nodules in lung tissues were analyzed (**A** and **B**). Data were shown as mean ± SD of at least three independent experiments.*, *p* < 0.05; **, *p* < 0.01; ***, *p* < 0.0001.C. Lung nodules were visualized by HE staining. D. ERβ expression in lung tissues was determined by immunohistochemical staining. E. TNFRSF17 expression in lung tissues was determined by immunohistochemical staining. Data were shown as mean ± SD of at least three independent experiments.*, *p* < 0.05; **, *p* < 0.01; ***, *p* < 0.0001

To determine the role of ERβ in 25-HC-mediated LAC metastasis in vivo, we constructed LAC metastatic model by intravenously injecting with A549 cells or A549-depleting ERβ, followed by injecting with 0 or 0.085 µM 25-HC via tail vein every 2days.The results showed that 25-HC increased the number and size of tumor nodules, suggesting that 25-HC accelerated LAC metastasis, which was significantly reduced by ERβ knockdown (Fig. 7A and B).Consistently, HE staining also verified that 25-HCenhanced the number of tumor nodules, which was blocked by ERβ knockdown (Fig. 7C). Further investigation evidenced that 25-HCelevated the level of ERβ in tumor tissues, which was blocked by ERβ knockdown (Fig. 7D). Interestingly, ERβ knockdown also remarkably reduced 25-HC-induced TNFRSF17 expression (Fig. 7E). These results suggested that ERβ was required for 25-HC-mediatedLAC metastasis and TNFRSF17 expression.

### Relationship between the expression of ERβ and TNFRSF17 and clinical features of patients with LAC

**Fig. 8:**
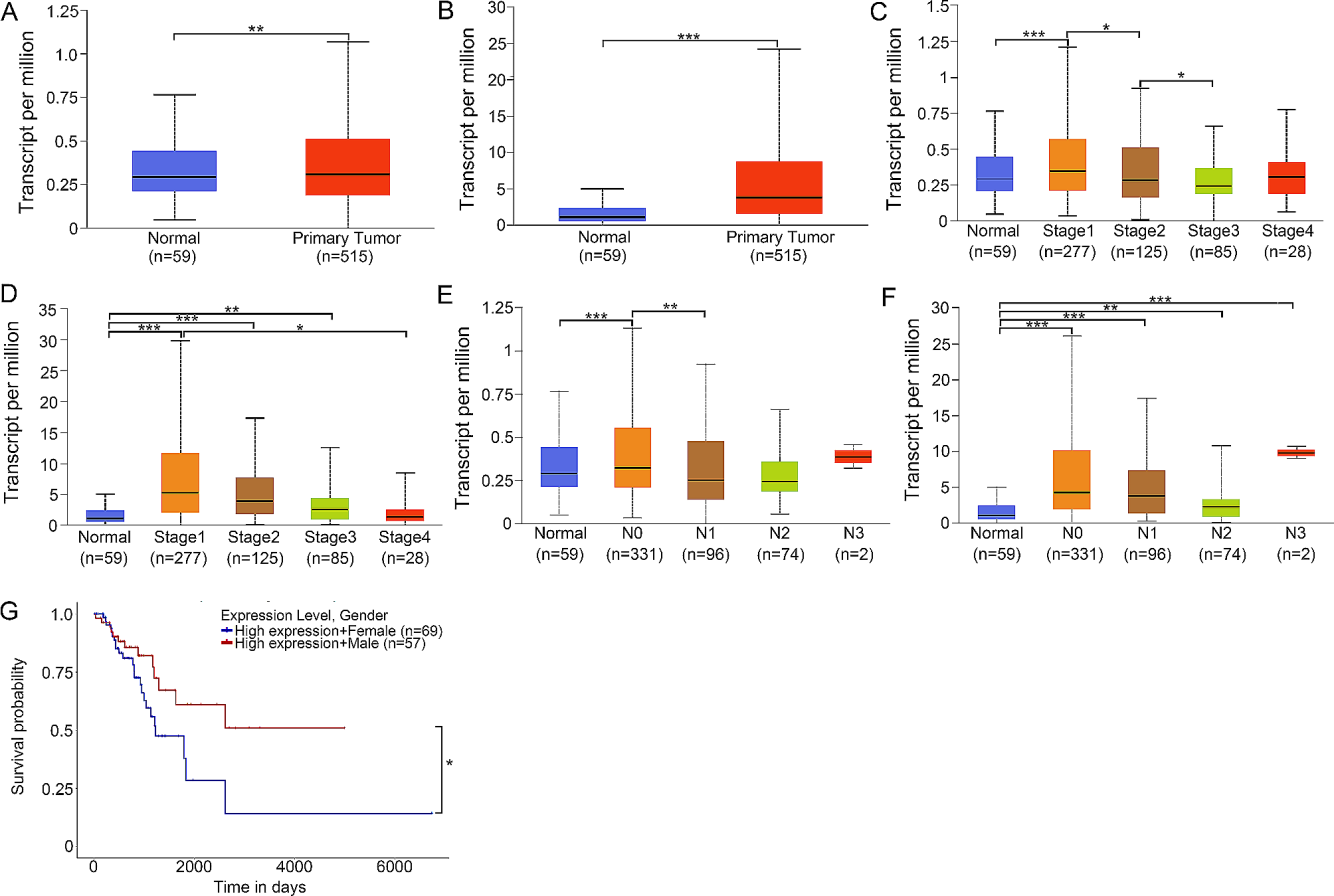
The expression of ERβ and TNFRSF17 were associated with clinical features of patients with LAC. (**A**) The differential expression of ERβ between normal tissues and lung adenocarcinoma. (**B**) The differential expression of TNFRSF17 between normal tissues and lung adenocarcinoma. (**C**) The correlation between ERβ expression and tumor stages. (**D**) The correlation between TNFRSF17 expression and tumor stages. (**E**) The correlation between ERβ expression and nodal metastasis status. (**F**) The correlation between TNFRSF17 expression and nodal metastasis status. (**G**) The effects of high ERβ expression on survival probability in male and female. *P* value was determined using log rank test.*, *p* < 0.05; **, *p* < 0.01; ***, *p* < 0.0001

We finally evaluated the expression of ERβ and TNFRSF17 in patients with LAC included in the TCGA database using the UALCAN portal. As shown in Fig. 8A and B, ERβ and TNFRSF17 is significantly up-regulated in primary LAC tumors (*n* = 515) compared to normal tissues (*n* = 59).The expression of ERβ was higher in LAC stage N1 compared to normal tissues (Fig. 8C), which was consistent with the expression of TNFRSF17 in LAC stage N1(Fig. 8D). Meanwhile, high expression of ERβ was associated with the nodal metastasis status N0 while high expression of TNFRSF17 was closely related to the nodal metastasis status N0, N1, N2 and N3 (Fig. 8E and F). In addition, high expression of TNFRSF17 was significantly associated with pathological stages 2 and 3 (Fig. 8D). Interestingly, high expression of ERβ in female patients with LAC notably reduced the survival probability compared to that in male patients with LAC (Fig. 8G).

## Discussion

25-hydroxycholesterol (25-HC) is an oxysterol catalyzed by cholesterol 25 hydroxylase (CH25H), which plays an important role not only in lipid metabolism, immunomodulation and antiviral activity [18–19], but also in tumor progression [8, 20]. 25-HC promotes cell migration and invasion of lung, gastric, brain, and breast cancer [10–13]. LXR serves as a receptor of 25-HC, and is required for 25-HC-mediated actions [21]. 25-HC also activates ERα-dependent signaling in breast and ovarian cancer cells or in cardiomyocytes [12]. However, in the present study, we evidenced that ERβ was also key for 25-HC-mediated proliferation, migration and metastasis of LAC. Considering that high cholesterol enhances LAC metastasis [17], we analyzed the differential metabolites of cholesterol between non-bone metastatic and bone metastatic mice. The results showed higher serum 25-HC in bone metastatic mice than that in non-bone metastatic mice(0.085 and 0.013 µM, respectively). 0.085µM 25-HC significantly triggered the expression of ERβ. ERβ knockdown blocked the effect of 25-HC on LAC proliferation, migration and invasion.27-hydroxycholesterol, as a structural analogue of 25-HC, has been identified as an ERβ-selective regulator (SERM) that dose-dependently inhibited ERβ transcriptional activity in aortic endothelial cells and breast cancer cells [22], but dose-dependently elevated ERβ transcriptional activity in the hepatocellular carcinoma cells and colon cancer cells [23]. These findings suggest that the actions of 27-hydroxycholesterol are cell-type specific. However, the role of ERβ in 25-HC-mediated actions remained largely unknown.

ERβ, as a member of the nuclear receptor family, is specifically expressed in normal lung tissues and lung tumors [7]. ERβ expression is elevated in lung adenocarcinoma, which is strongly associated with the histologic subtypes and grades [24], suggesting an important role of ERβ in lung adenocarcinoma. Our results also evidenced that ERβ expression was higher in lung adenocarcinoma than that in normal tissues, and was significantly related to the LAC stage and nodal metastasis status. Intriguingly, 25-HC treatment induced ERβ expression, and ERβ knockdown suppressed the proliferation, migration, and invasion of LAC mediated by 25-HC. These results indicated that ERβ was required for the functional actions of 25-HC.

How did ERβ act in LAC? Bioinformatics analysis was performed on GSE50081 dataset from the GEO database (http://www.ncbi.nlm.nih). In the R environment (version 3.5.3, https://www.r-project.org/), the affy package (http://www.bioconductor.org/packages/release/bioc/html/affy.html) was used to carry out raw data preprocessing and normalization. The limma package in R (http://www.bioconductor.org/packages/release/bioc/html/limma.html) was used to identify LAC samples. Based on the median level of ERβ expression, LAC patient samples were divided into ERβ-high and ERβ-low groups.TNFRSF17 was identified as a closely related gene of ERβ. ERβ knockdown reduced the mRNA and protein levels of TNFRSF17, suggesting that TNFRSF17 should be a downstream protein of ERβ. Combined with the result that TNFRSF17 silencing also blocked the effects of 25-HC on the proliferation, migration, and invasion of LAC cells, our results suggested that 25-HC promoted the proliferation, migration and invasion of LAC by activating ERβ/TNFRSF17 axis.

TNFRSF17, known as BCMA, is expressed on the membranes of malignant plasma cells and mature B cells [14]. Ligand activation of TNFRSF17 in multiple myeloma cells facilitates proliferation and drug resistance [25]. TNFRSF17-targeting CAR T cells could effectively kill multiple myeloma cells [26]. However, the role of TNFRSF17 in lung adenocarcinoma remains largely unknown. A recent research demonstrates a close association between TNFRSF17 and patient prognosis [27]. Our results also verified that TNFRSF17 expression was higher in lung adenocarcinoma than that in normal tissues, and was significantly correlated to LAC stage and nodal metastasis status. These results suggested that elevated TNFRSF17 should contribute to LAC progression.

To confirm the above conclusion, we constructed LAC metastatic model by intravenously injecting with A549 cells or A549-depleting ERβ, followed by injecting with 0 or 0.085µM 25-HC. The results showed that ERβ knockdown not only reduced LAC metastasis, but also inhibited the expression of TNFRSF17 in LAC tissues. Meantime, 25-HC treatment simultaneously increased the expression of ERβ and TNFRSF17, and accelerated LAC metastasis, which was blocked by ERβ knockdown. These results suggested that 25-HC promoted the proliferation and metastasis of LAC by regulating ERβ/TNFRSF17 axis.

### Electronic supplementary material

Below is the link to the electronic supplementary material.

Supplementary Material 1

Supplementary Material 2

Supplementary Material 3

Supplementary Material 4

Supplementary Material 5

## Data Availability

All data used or analyzed during this study are included in thispublished article.
