# Supplementary material for: 25-hydroxycholesterol promotes proliferation and metastasis of lung adenocarcinoma cells by regulating ERβ/TNFRSF17 axis
Source: BMC Cancer. 2024 Apr 22;24:505. doi: 10.1186/s12885-024-12227-4 (PMC11034116; doi:10.1186/s12885-024-12227-4)
Supplement: Supplementary file 1 — Supplementary Material 1 [file 12885_2024_12227_MOESM1_ESM.docx]

Supplementary Table 1 The primer sequences

| Gene | Sequences |
| --- | --- |
| ERβ | Forward 5 ′ -GCAGACCACAAGCCCAAATG-3 ′ |
|  | Reverse 5 ′ -AGCGATCTTGCTTCACACCA-3 ′ |
| TNFRSF17 | Forward 5 ′ -CAAGAGCAAACCGAAGGTCG-3 ′ |
|  | Reverse 5 ′ -TGACAAGAATGGTTGCGCCT-3 ′ |
| GAPDH | Forward 5 ′ -GCACCGTCAAGGCTGAGAAC-3 ′ |
|  | Reverse 5 ′ -TGGTGAAGACGCCAGTGGA-3 ′ |
