# Supplementary material for: 25-hydroxycholesterol promotes proliferation and metastasis of lung adenocarcinoma cells by regulating ERβ/TNFRSF17 axis
Source: BMC Cancer. 2024 Apr 22;24:505. doi: 10.1186/s12885-024-12227-4 (PMC11034116; doi:10.1186/s12885-024-12227-4)
Supplement: Supplementary file 2 — Supplementary Material 2 [file 12885_2024_12227_MOESM2_ESM.docx]

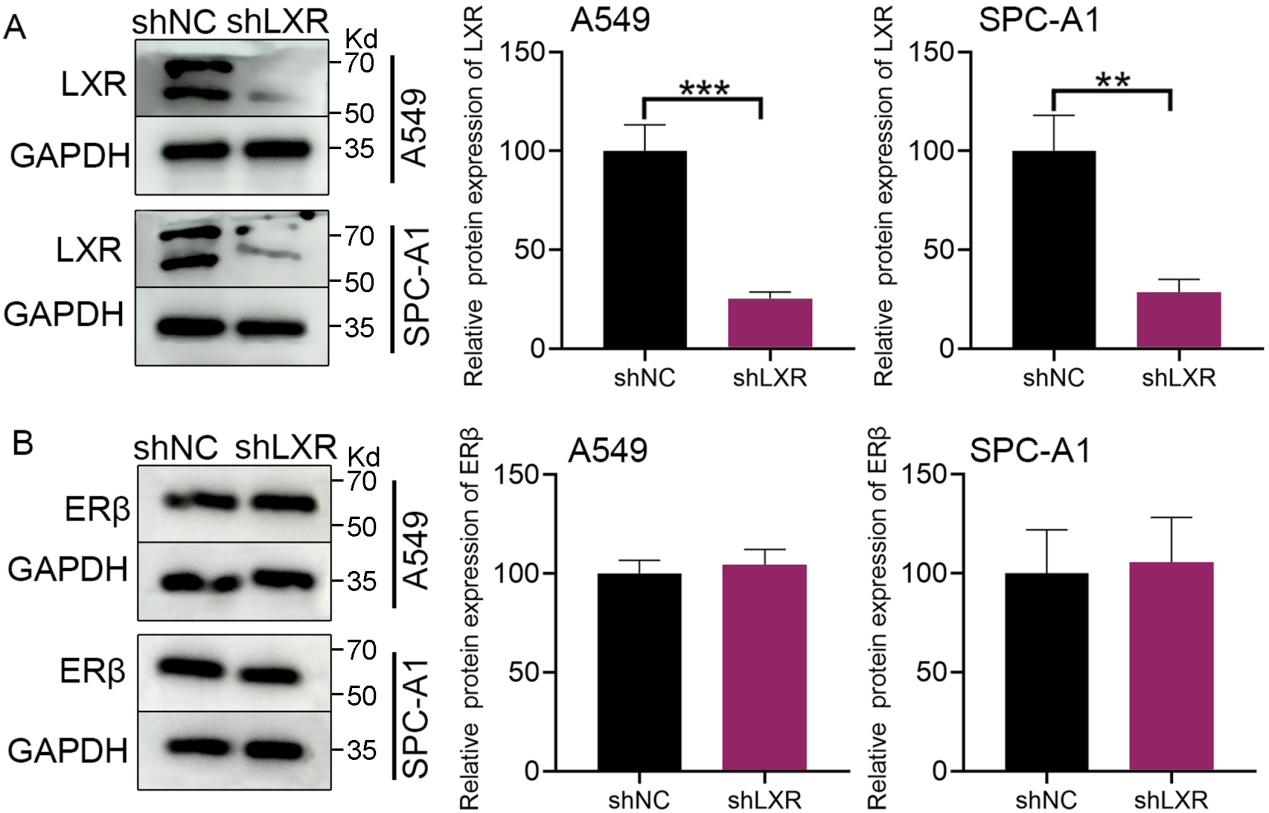


Supplementary Figure 1 LXR Knockdown did not affect the expression of ERβ. A. A549 and SPC-A1 cells were transfected with the lentivirus carrying NR1H3-sgRNA, and the protein expression of LXR was measured by western blot analysis. B. The effect of LXR knockdown on the protein expression of ERβ in the presence of 0.085 μM 25-HC.


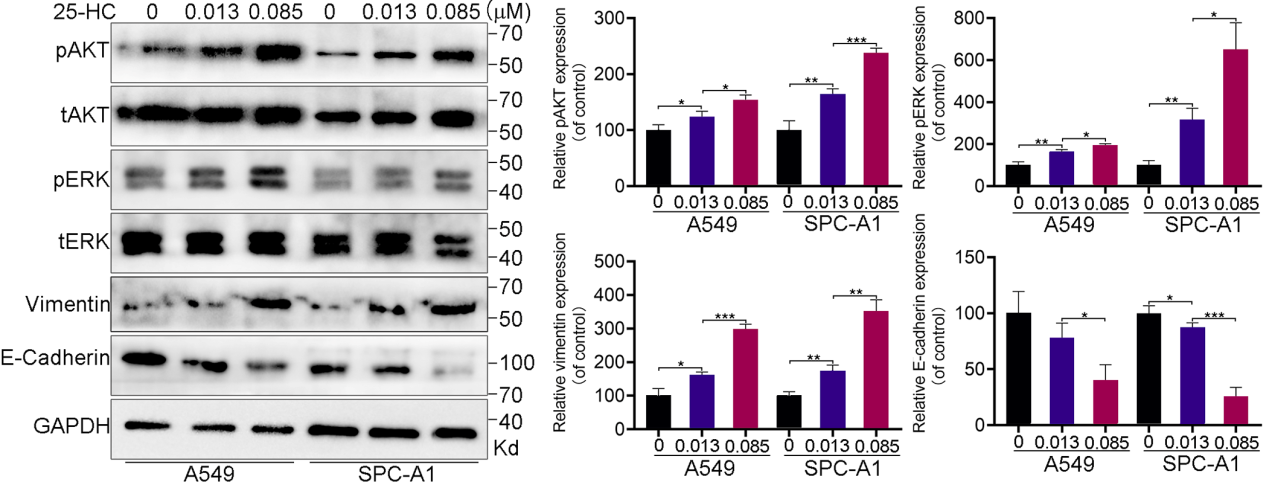


Supplementary Figure 2 25-hydroxycholesterol induced the expression of pAKT, pERK, vimentin, and inhibited E-Cadherin expression. A549 and SPC-A1 cells were exposed to 0, 0.013 and 0.085 μM 25-HC for 48 h. The protein expression of phosphorylated AKT (pAKT), total AKT (tAKT), phosphorylated ERK (pERK), total ERK (tERK), vimentin, and E-Cadherin was measured by western blot analysis. *, p<0.05; **, p<0.01; ***, p<0.0001.
