# Supplementary material for: 25-hydroxycholesterol promotes proliferation and metastasis of lung adenocarcinoma cells by regulating ERβ/TNFRSF17 axis
Source: BMC Cancer. 2024 Apr 22;24:505. doi: 10.1186/s12885-024-12227-4 (PMC11034116; doi:10.1186/s12885-024-12227-4)
Supplement: Supplementary file 3 — Supplementary Material 3 [file 12885_2024_12227_MOESM3_ESM.pdf]

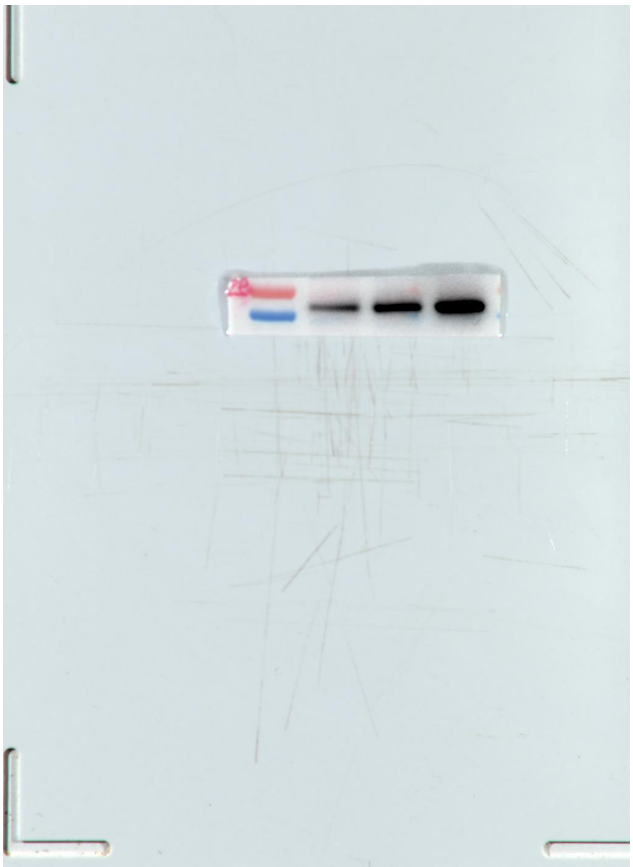

Fig.1C ERβ expression in A549 cells

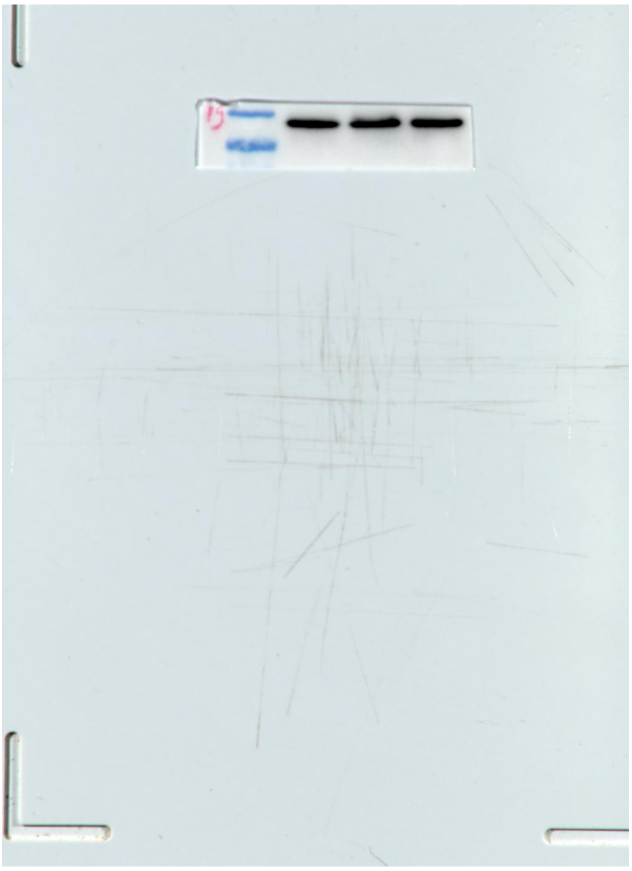

Fig.1C GAPDH expression in A549 cells

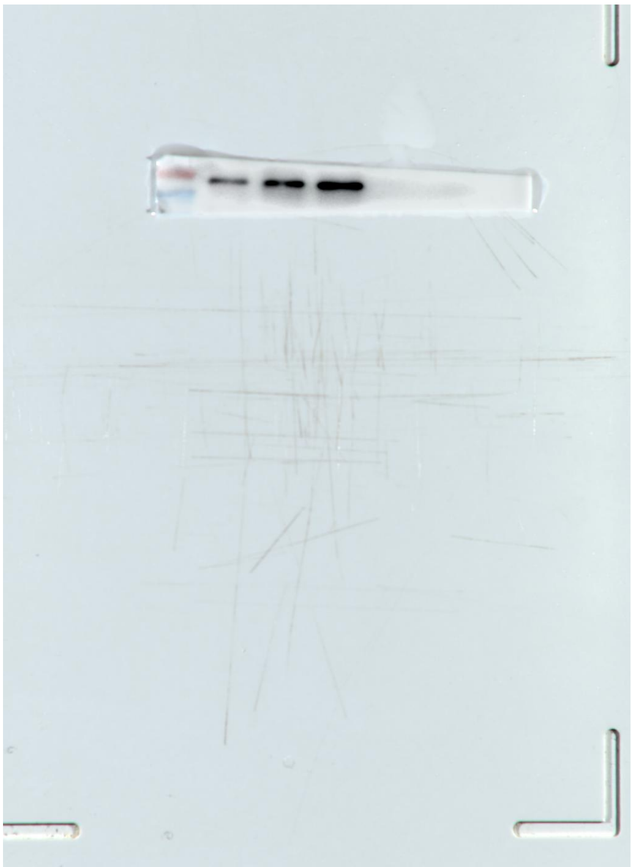

Fig.1C ERβ expression in SPC-A1 cells

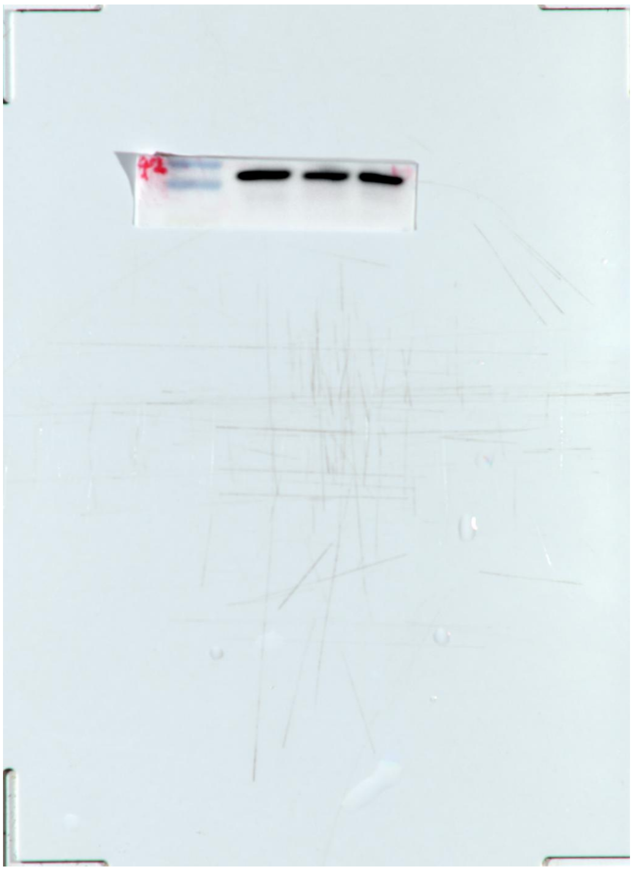

Fig.1C GAPDH expression in SPC-A1 cells

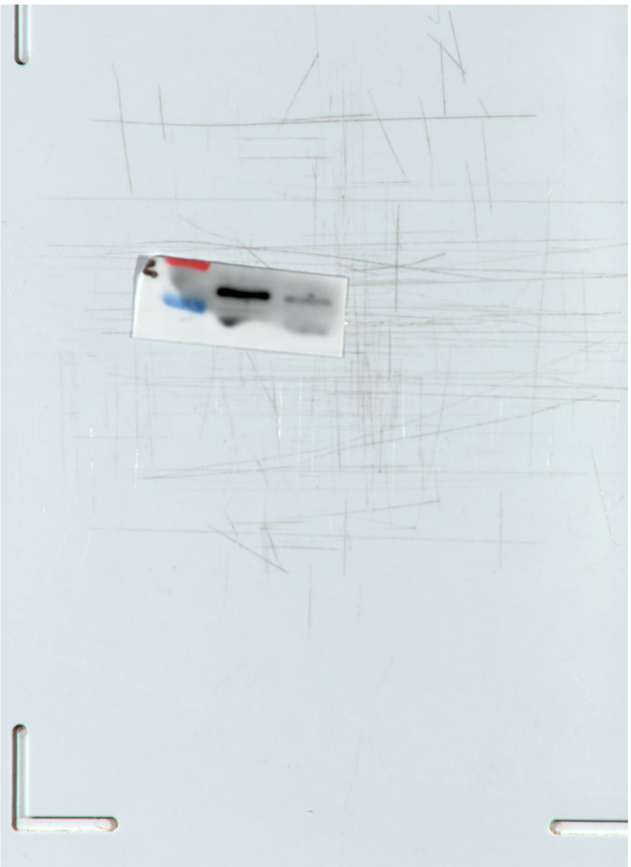

Fig.1D ERβ expression in A549 cells

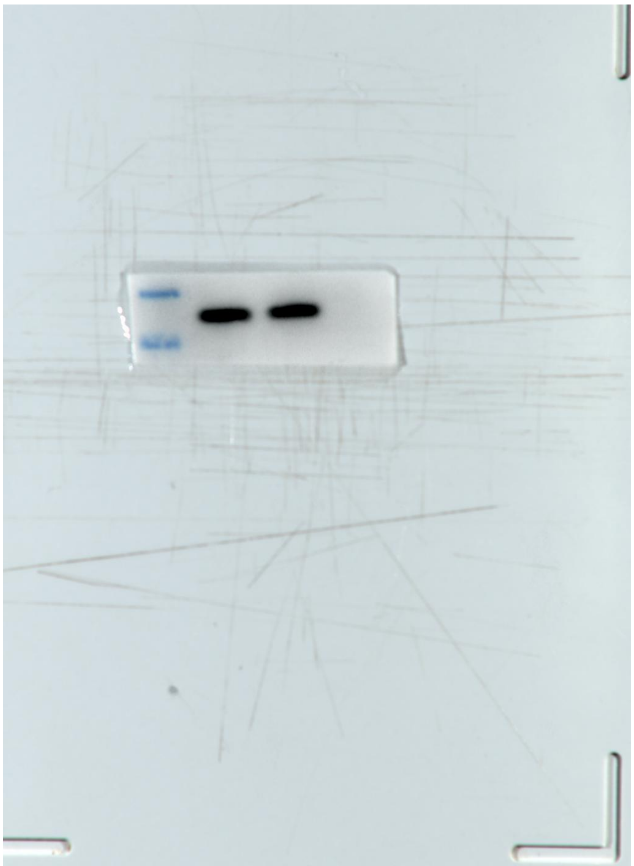

Fig.1D GAPDH expression in A549 cells

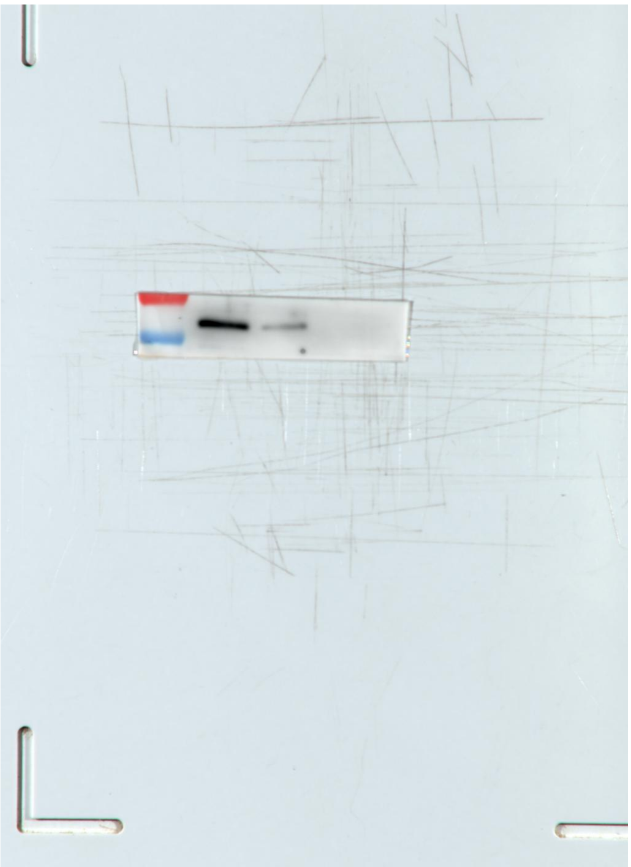

Fig.1D ERβ expression in SPC-A1cells

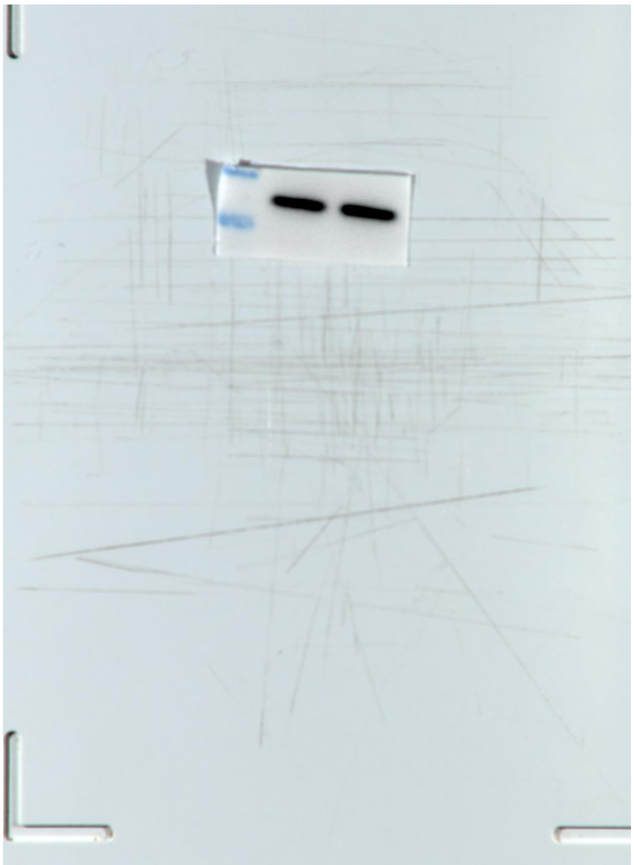

Fig.1D GAPDH expression in SPC-A1 cells

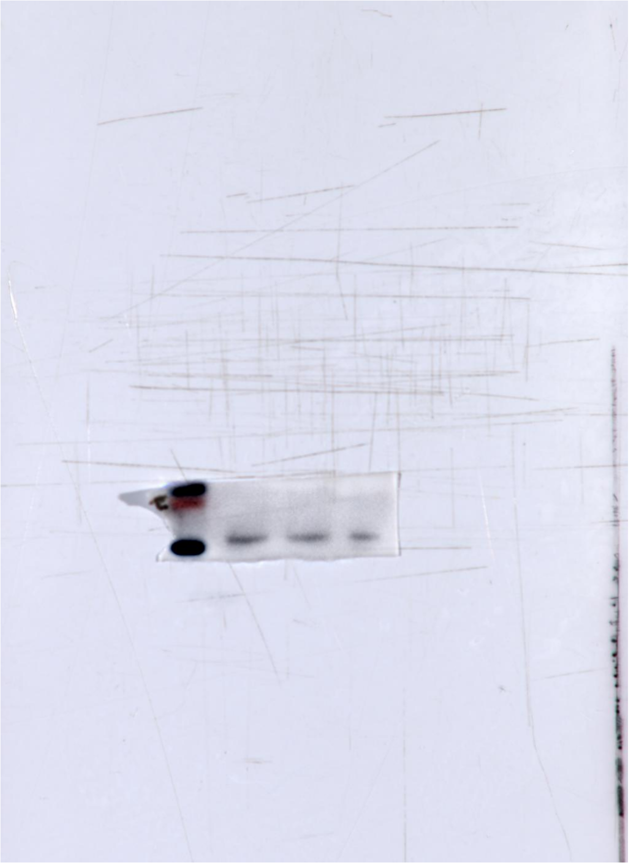

Fig.1E ERβ expression in A549 cells

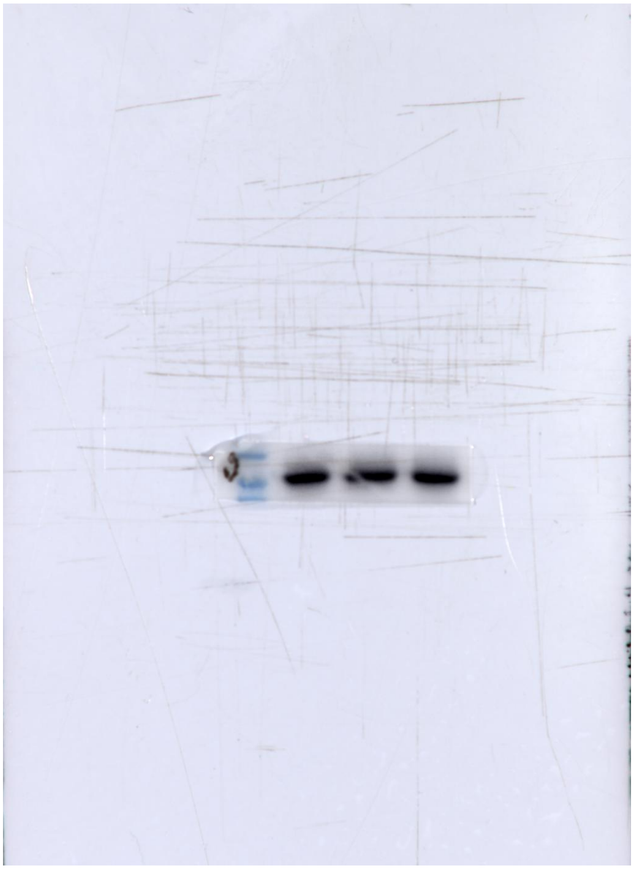

Fig.1E GAPDH expression in A549 cells

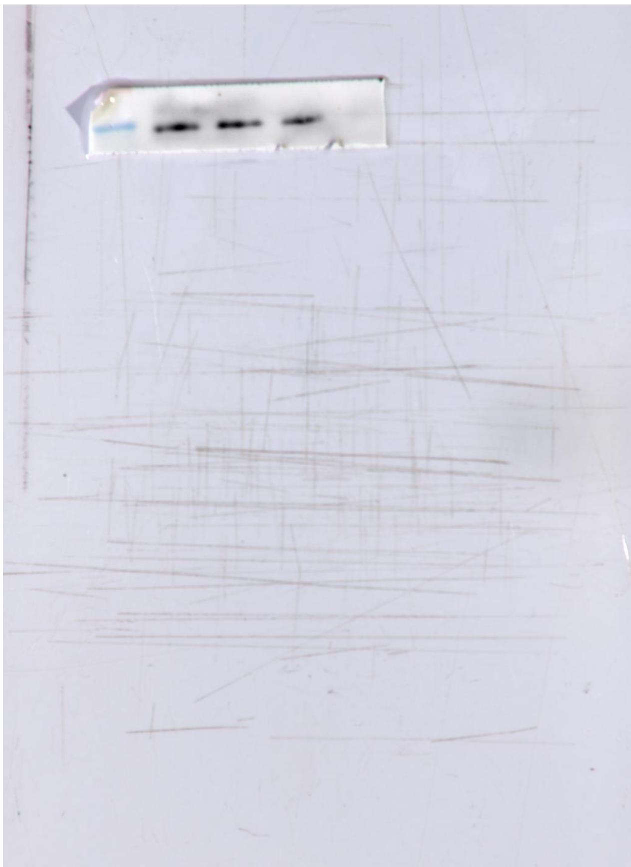

Fig.1E ERβ expression in SPC-A1 cells

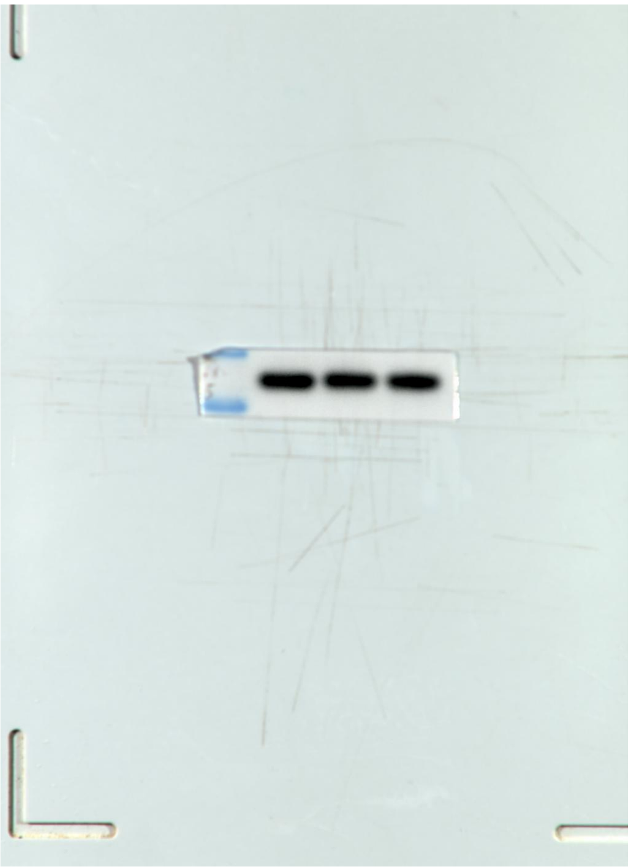

Fig.1E GAPDH expression in SPC-A1 cells

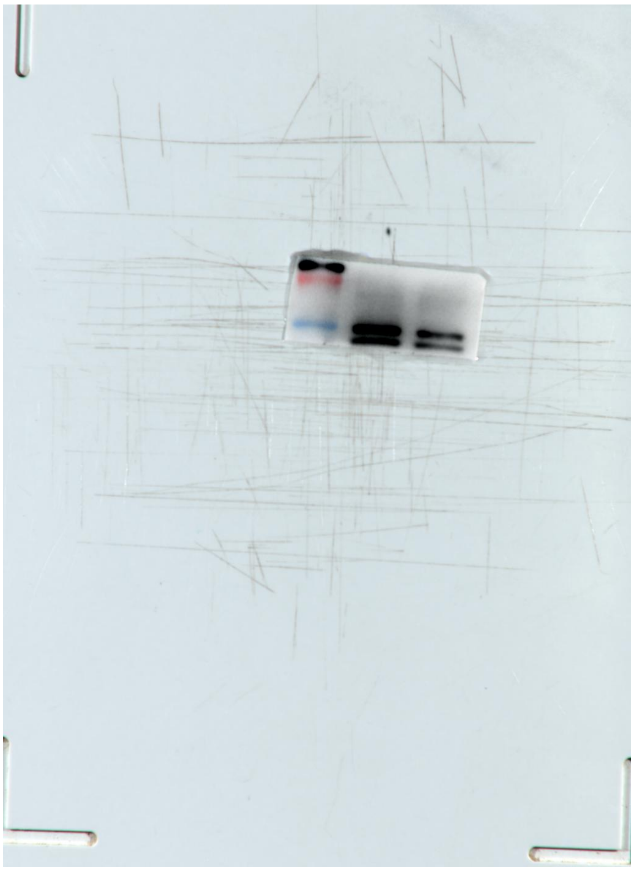

Fig.4E TNFRSF17 expression in A549 cells

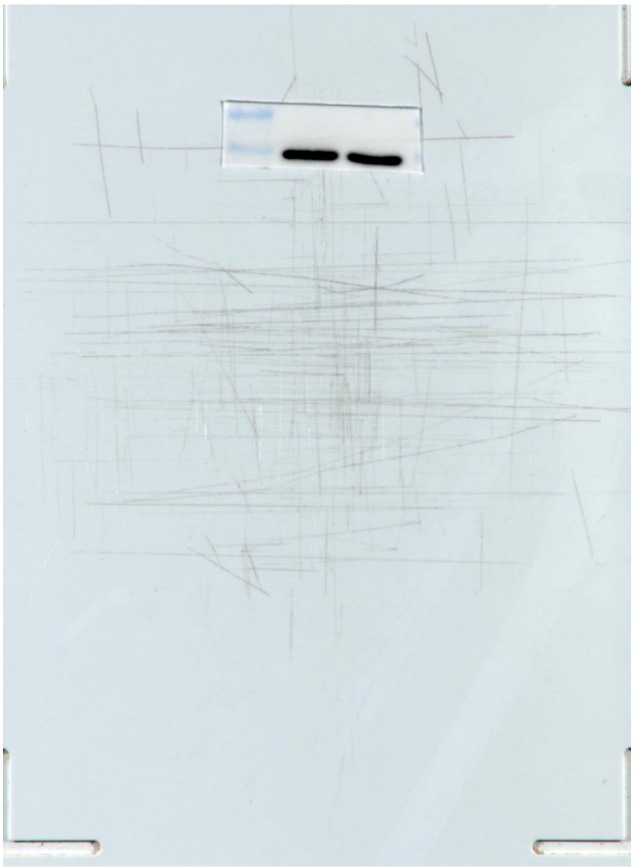

Fig.4E GAPDH expression in A549 cells

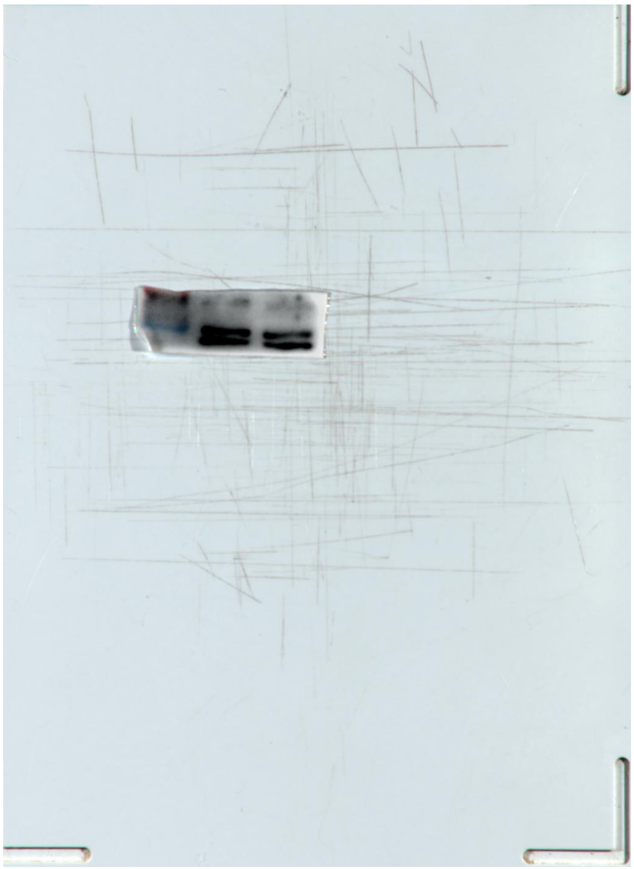

Fig.4E TNFRSF17 expression in SPC-A1 cells

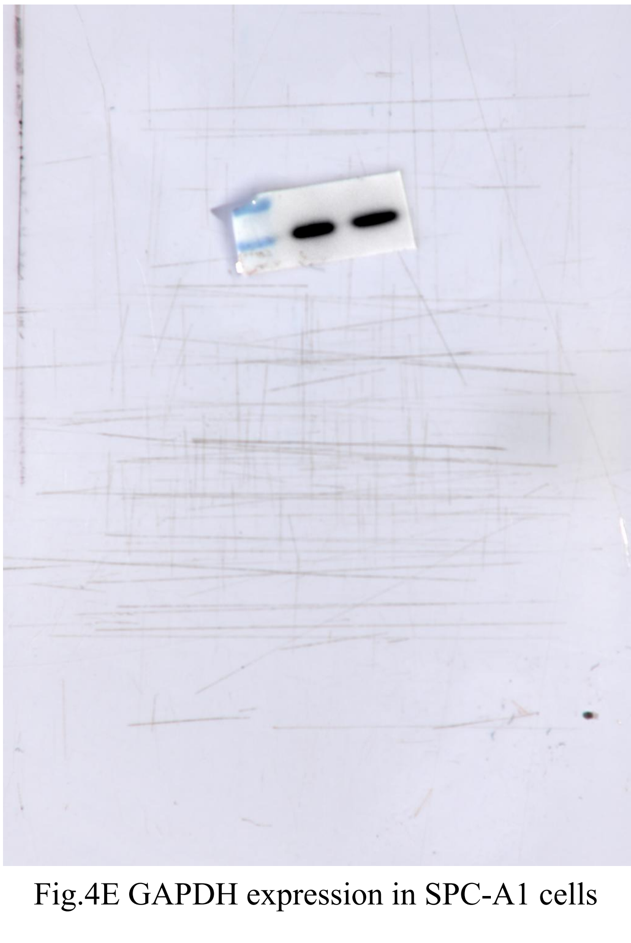

Fig.4E GAPDH expression in SPC-A1 cells

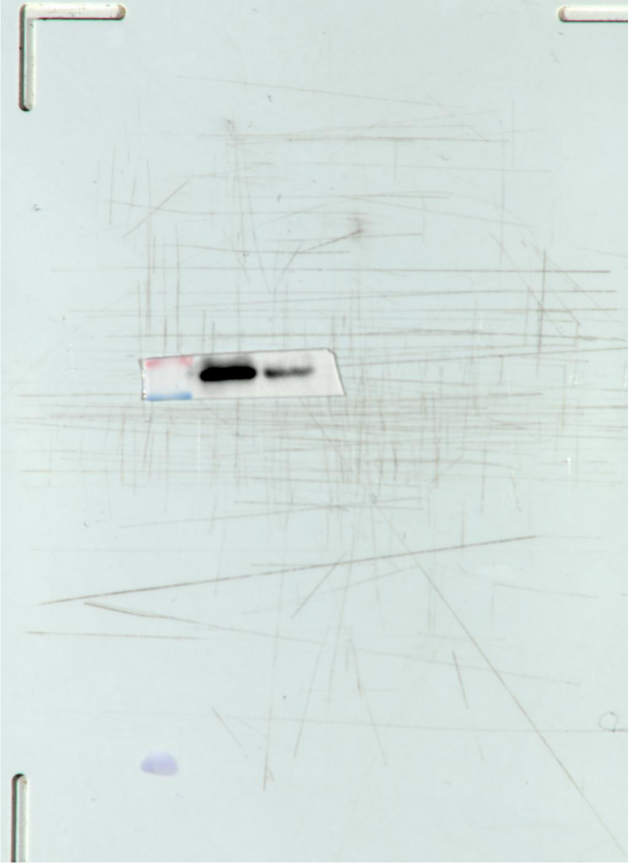

Fig.5A TNFRSF17 expression in A549 cells

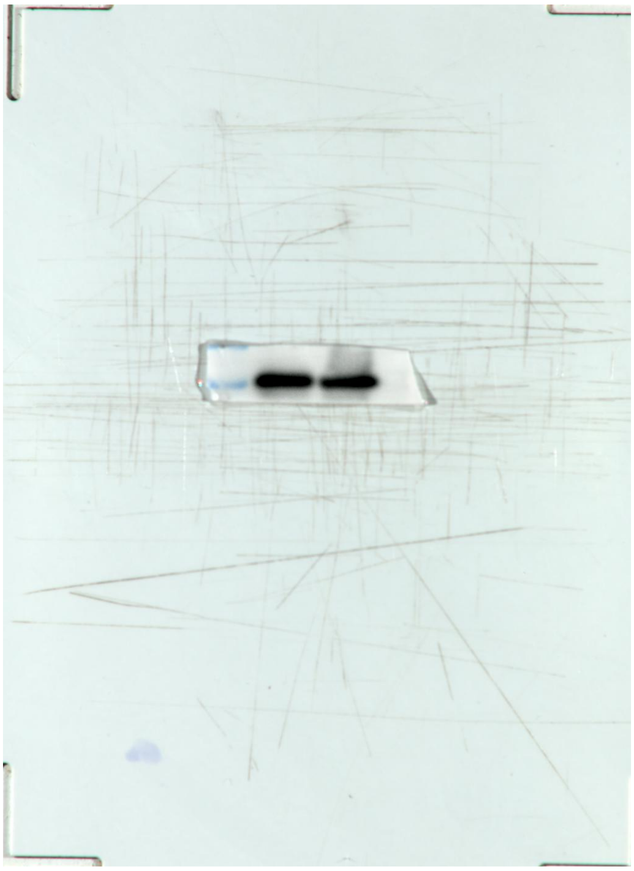

Fig.5A GAPDH expression in A549 cells

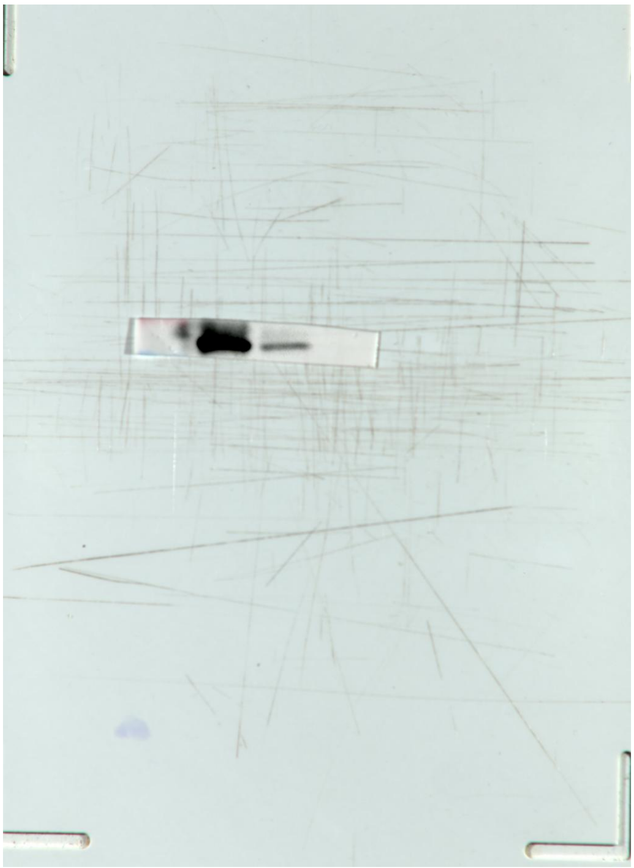

Fig.5A TNFRSF17 expression in SPC-A1 cells

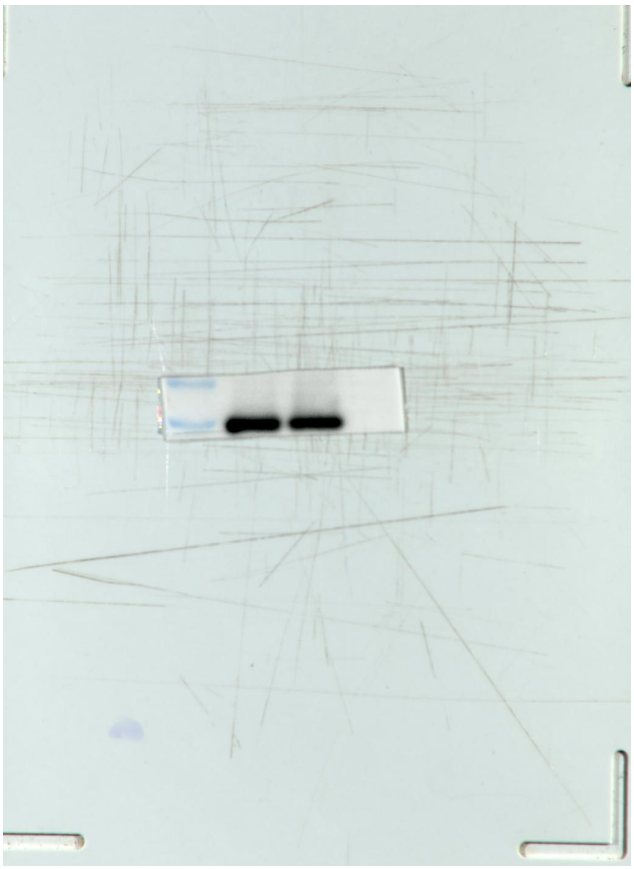

Fig.5A GAPDH expression in SPC-A1 cells

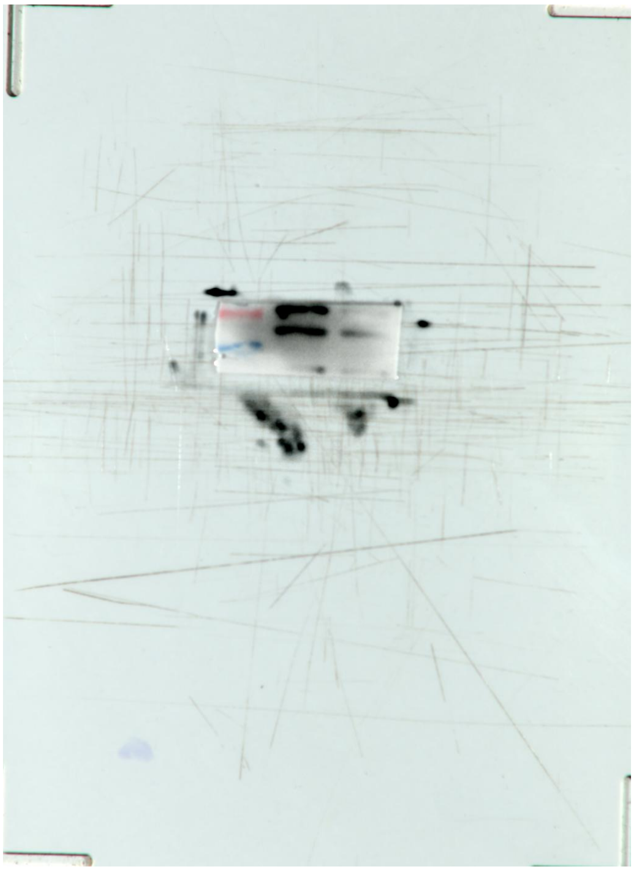

Supplementary Figure 1A LXR expression in A549 cells

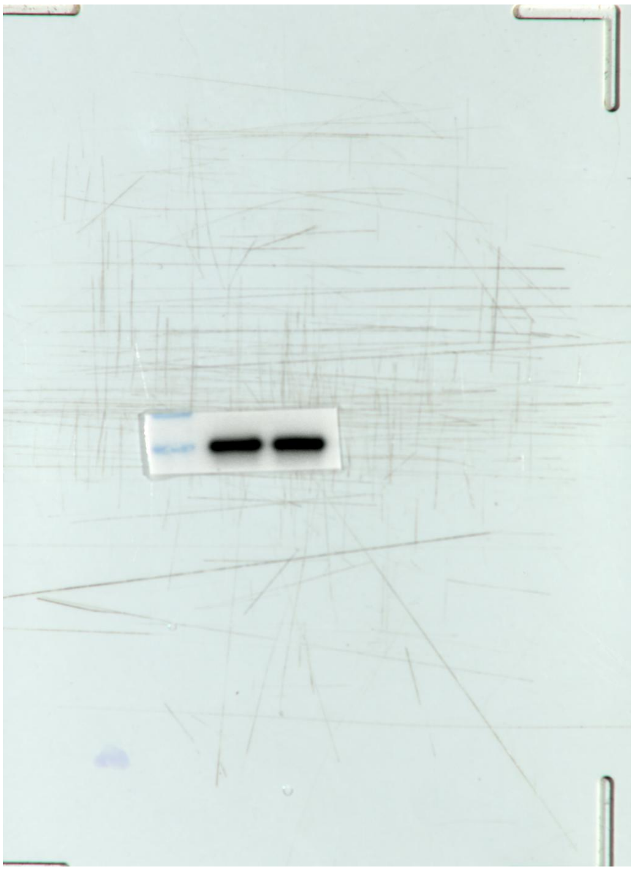

Supplementary Figure 1A GAPDH expression in A549 cells

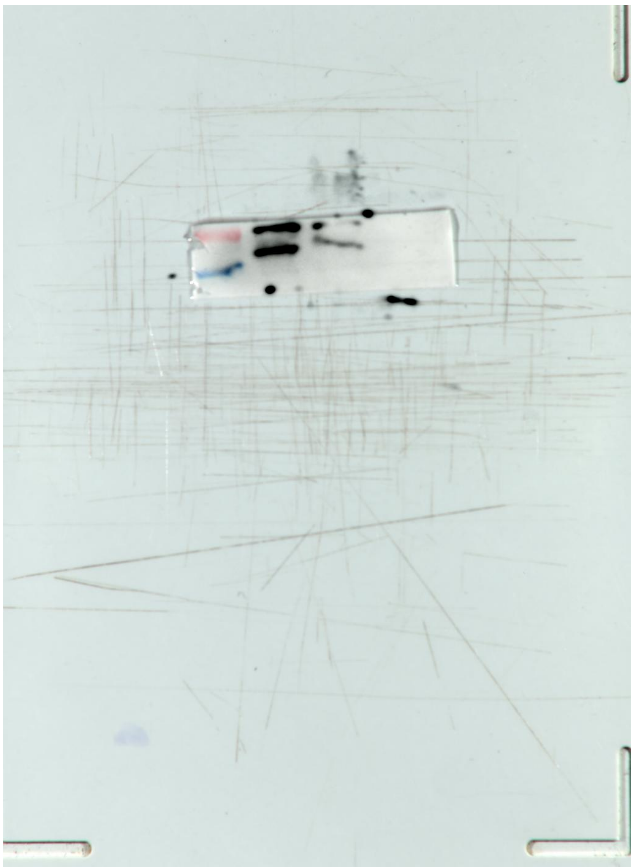

Supplementary Figure 1A LXR expression in SPC-A1 cells

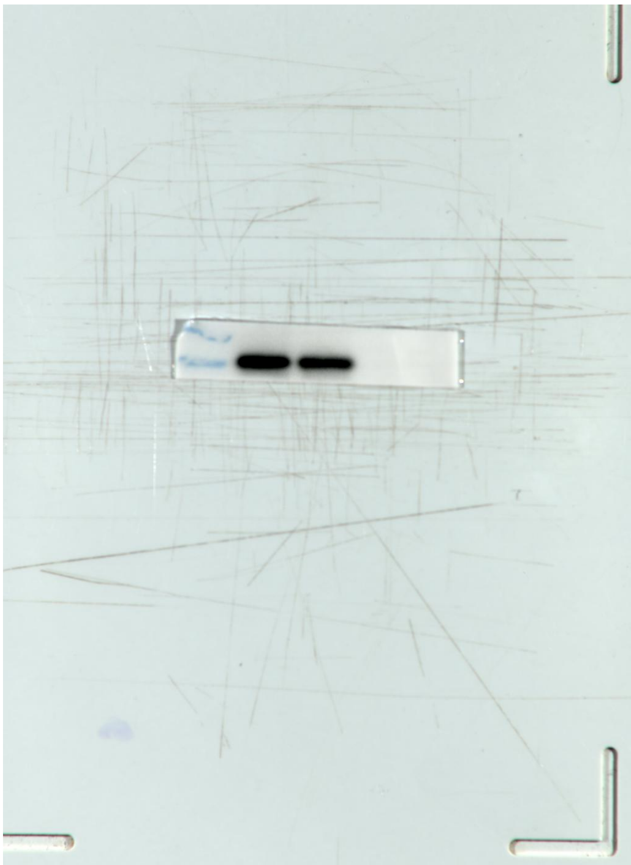

Supplementary Figure 1A GAPDH expression in SPC-A1 cells

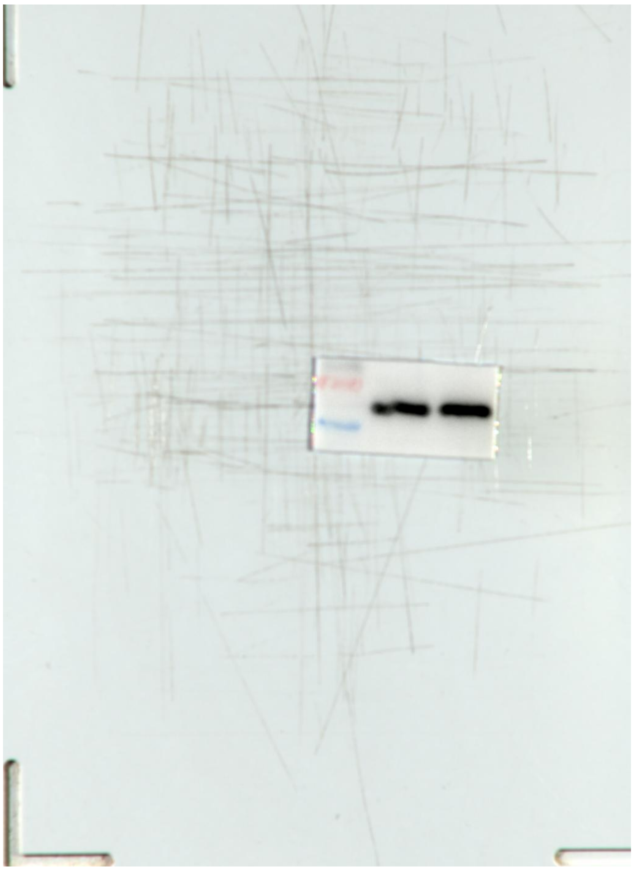

Supplementary Figure 1B ERβ expression in A549 cells

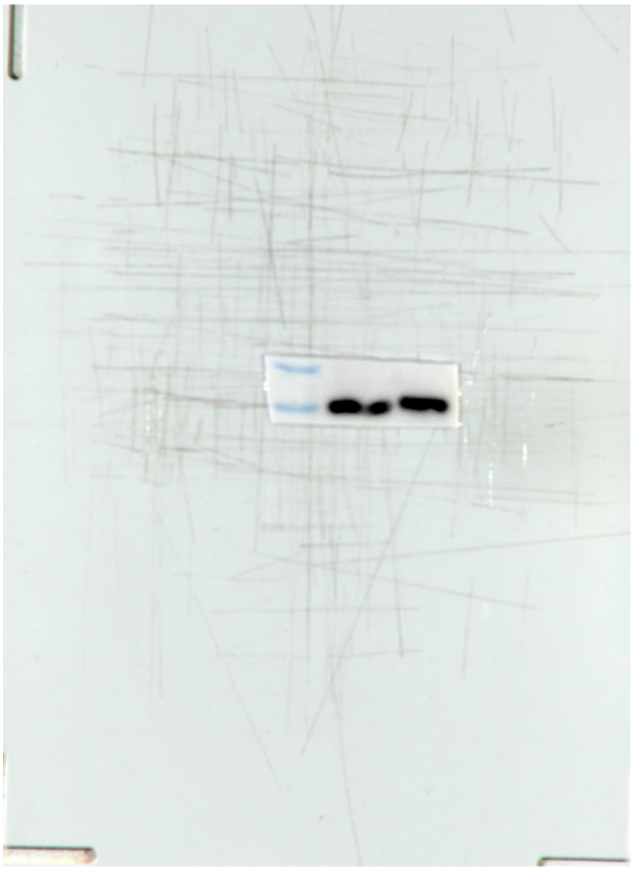

Supplementary Figure 1B GAPDH expression in A549 cells

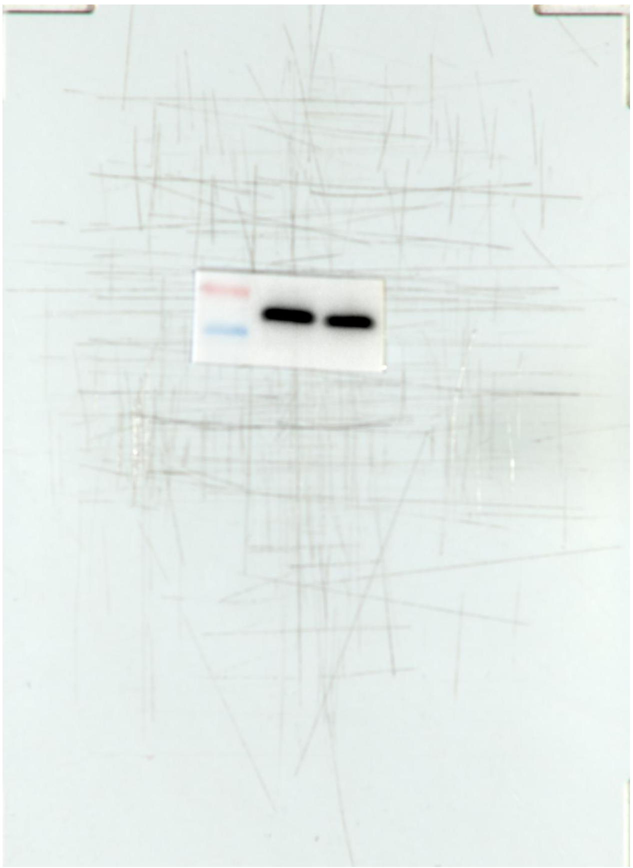

Supplementary Figure 1B ERβ expression in SPC-A1 cells

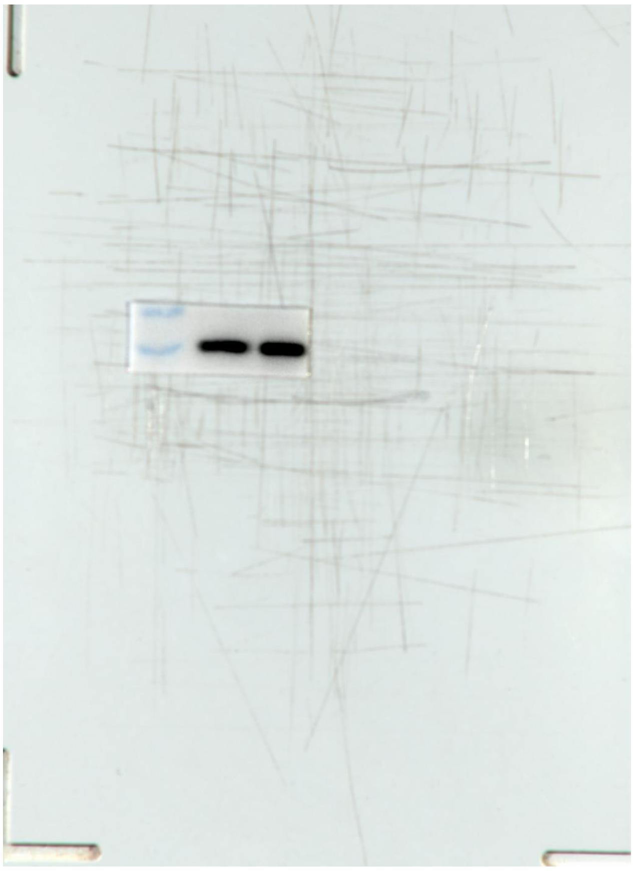

Supplementary Figure 1B GAPDH expression in SPC-A1 cells

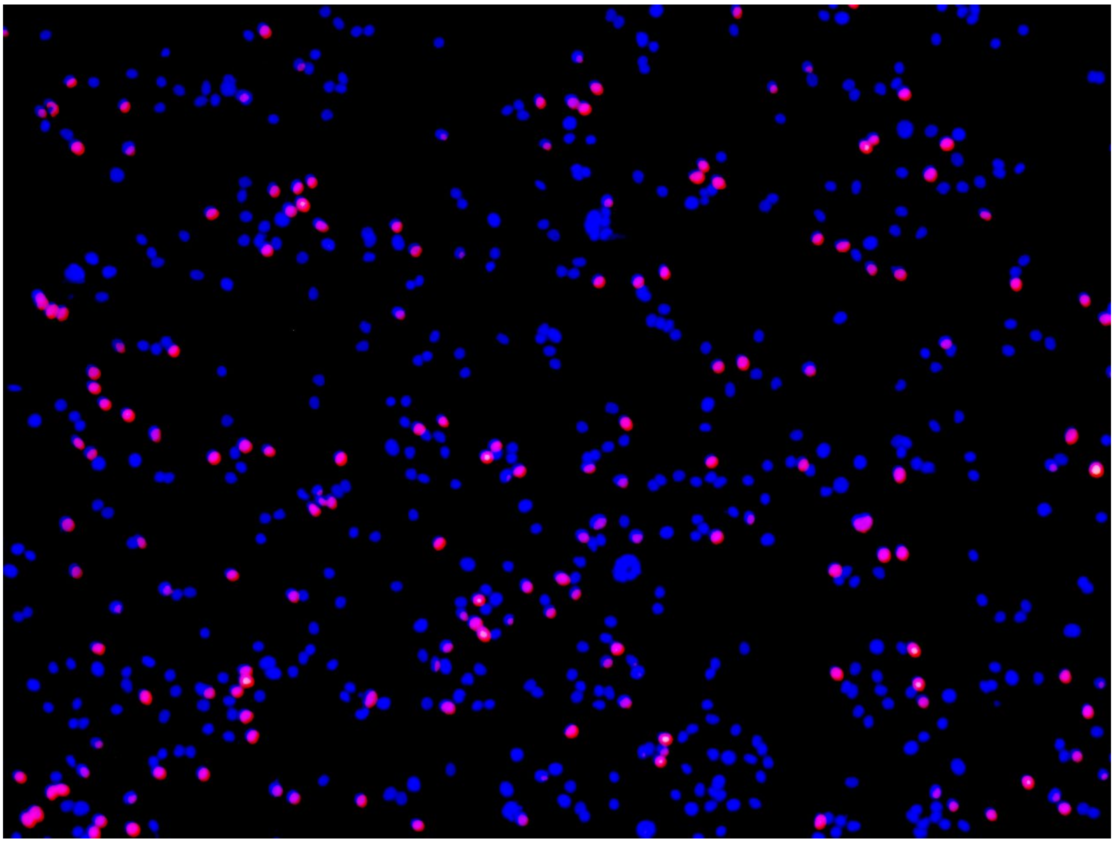

Fig.2B shNC group with 0 μM 25-HC in A549 cells

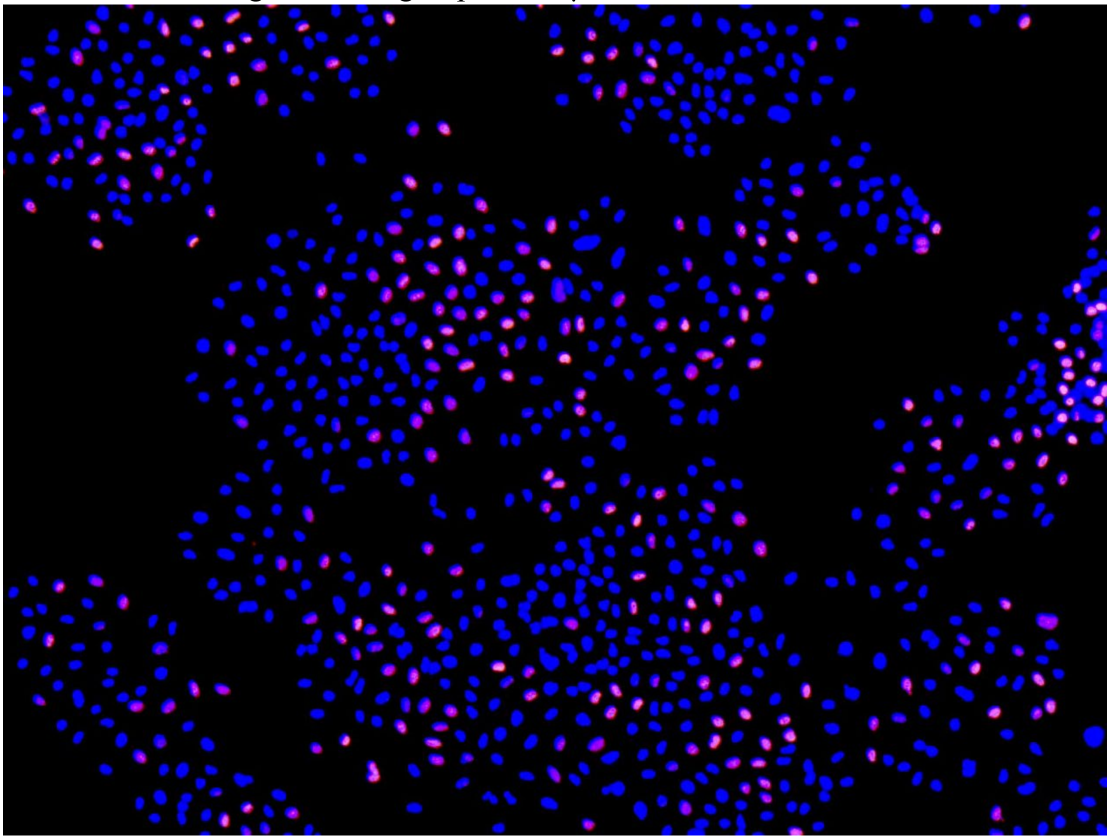

Fig.2B shNC group with 0.013 μM 25-HC in A549 cells

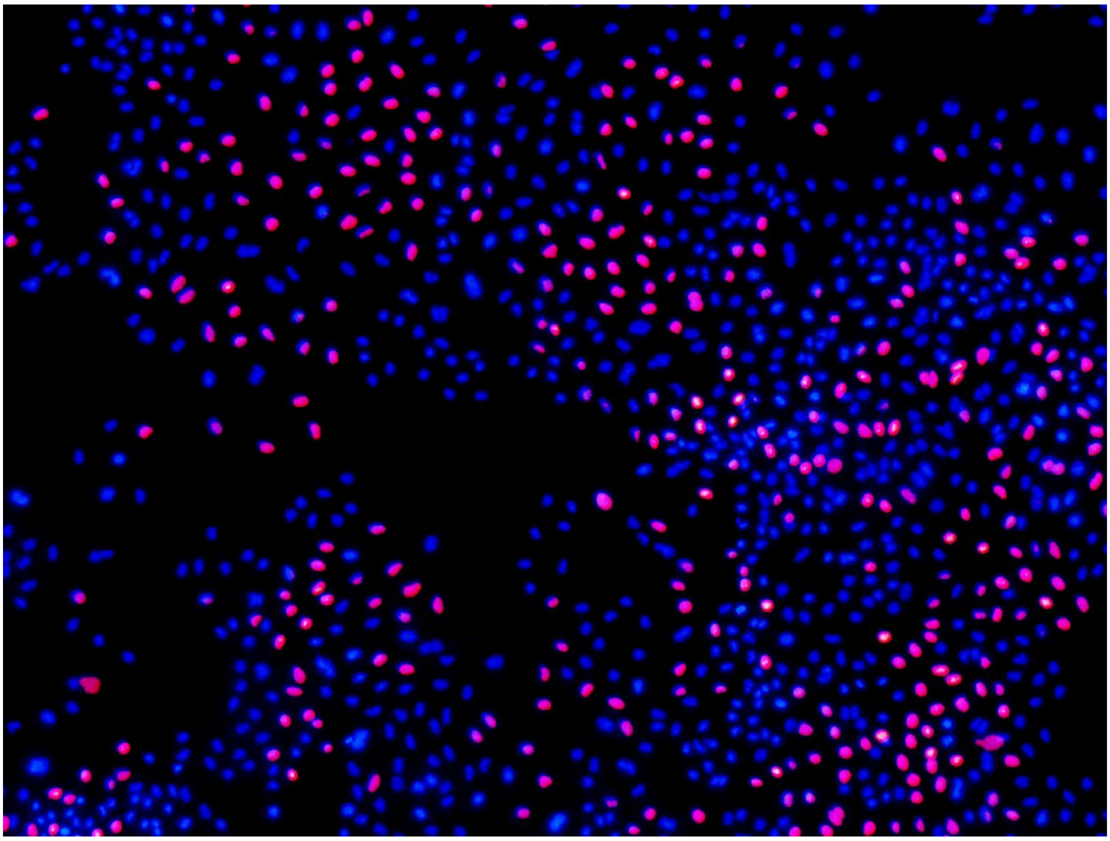
Fig.2B shNC group with 0.085 μM 25-HC in A549 cells
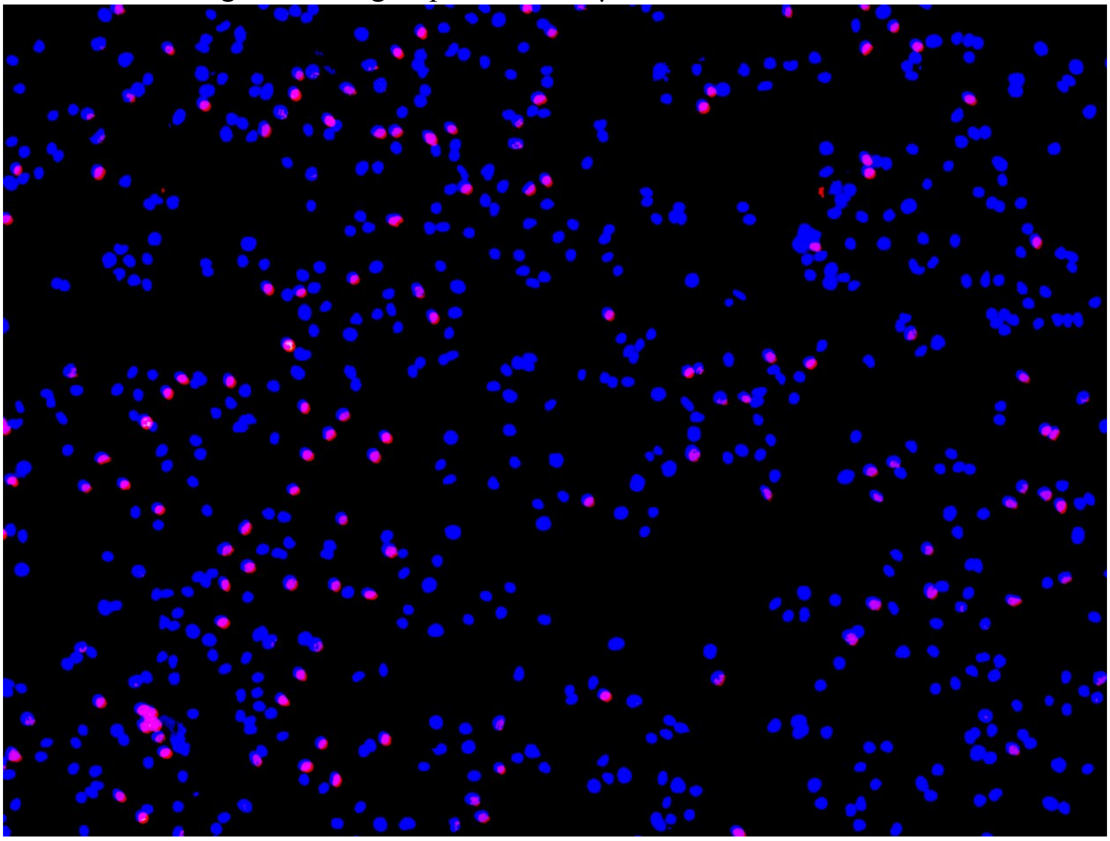
Fig.2B sh ERβ group with 0 μM 25-HC in A549 cells

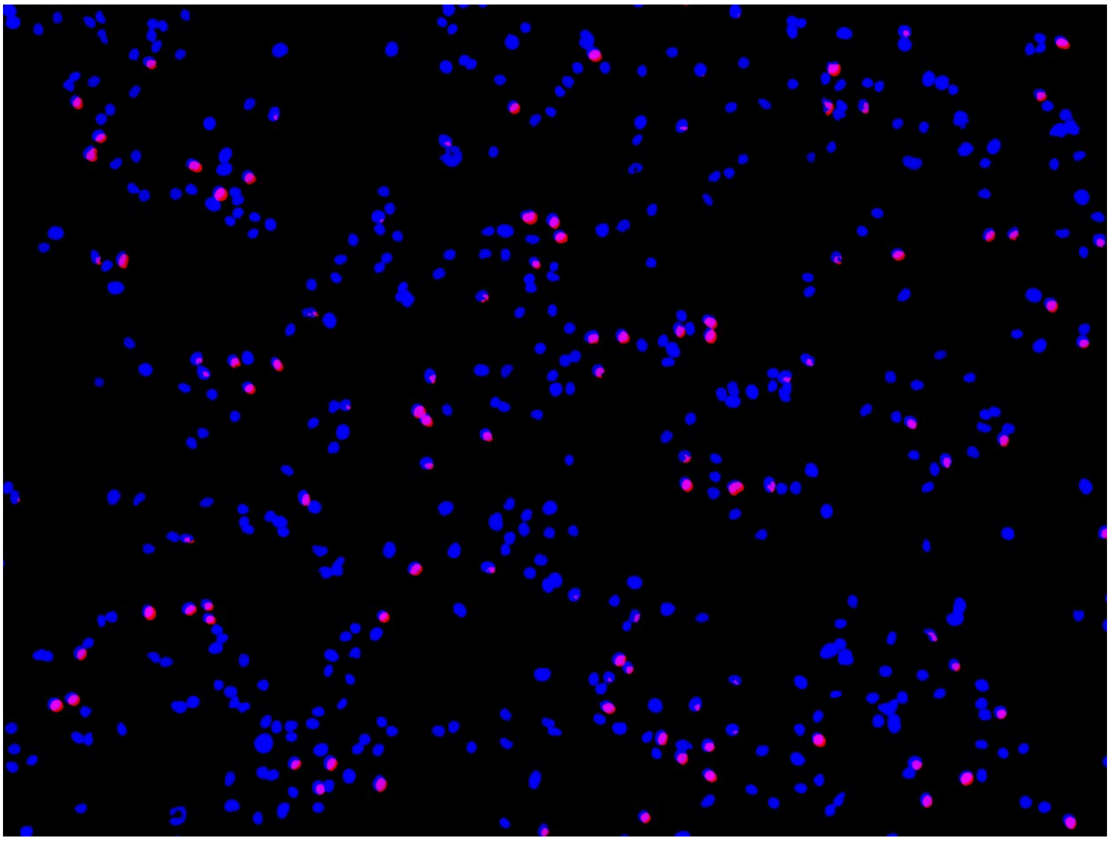

Fig.2B sh ERβ group with 0.013 μM 25-HC in A549 cells

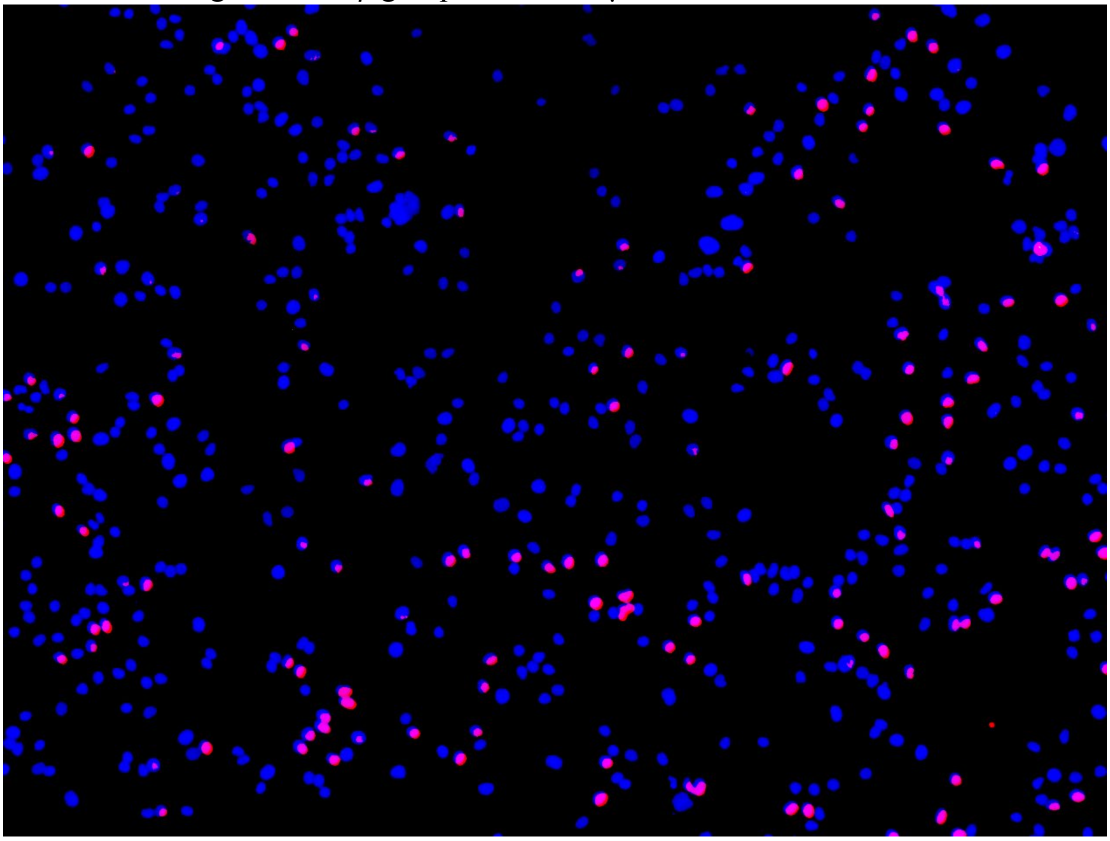

Fig.2B sh ERβ group with 0.085 μM 25-HC in A549 cells

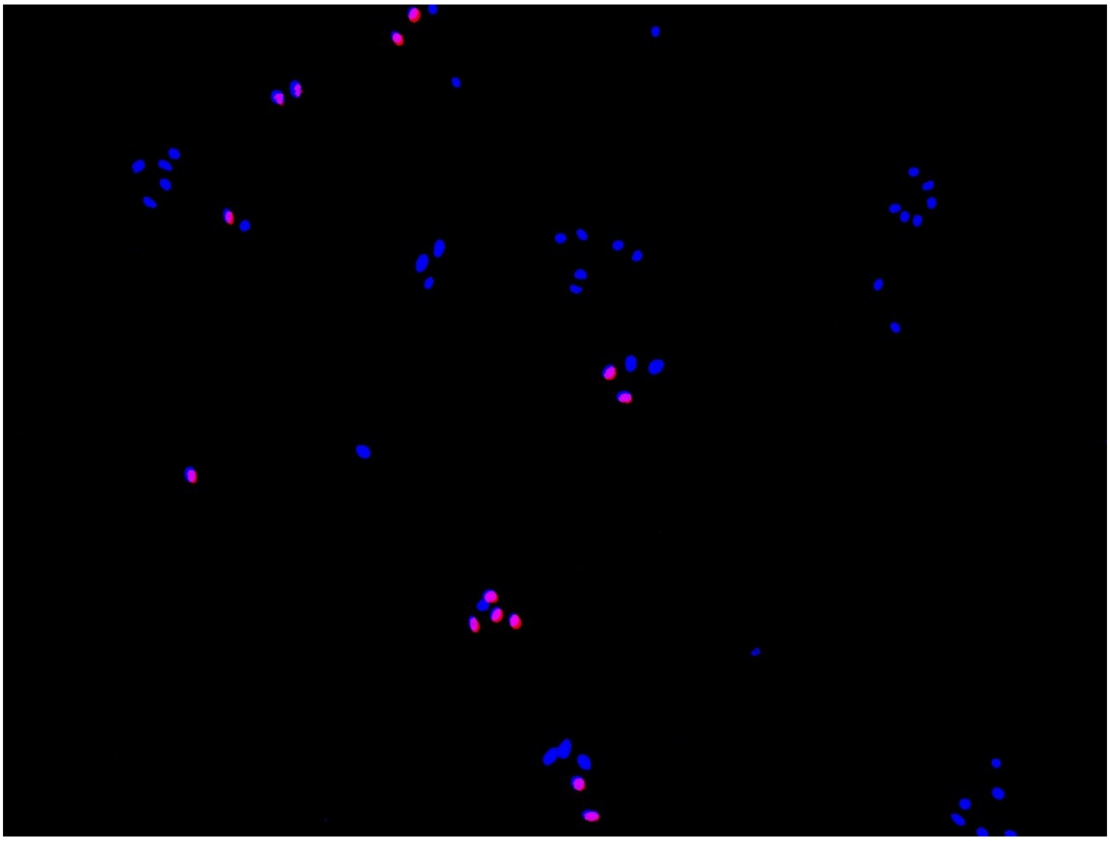

Fig.2B shNC group with 0 μM 25-HC in SPC-A1 cells

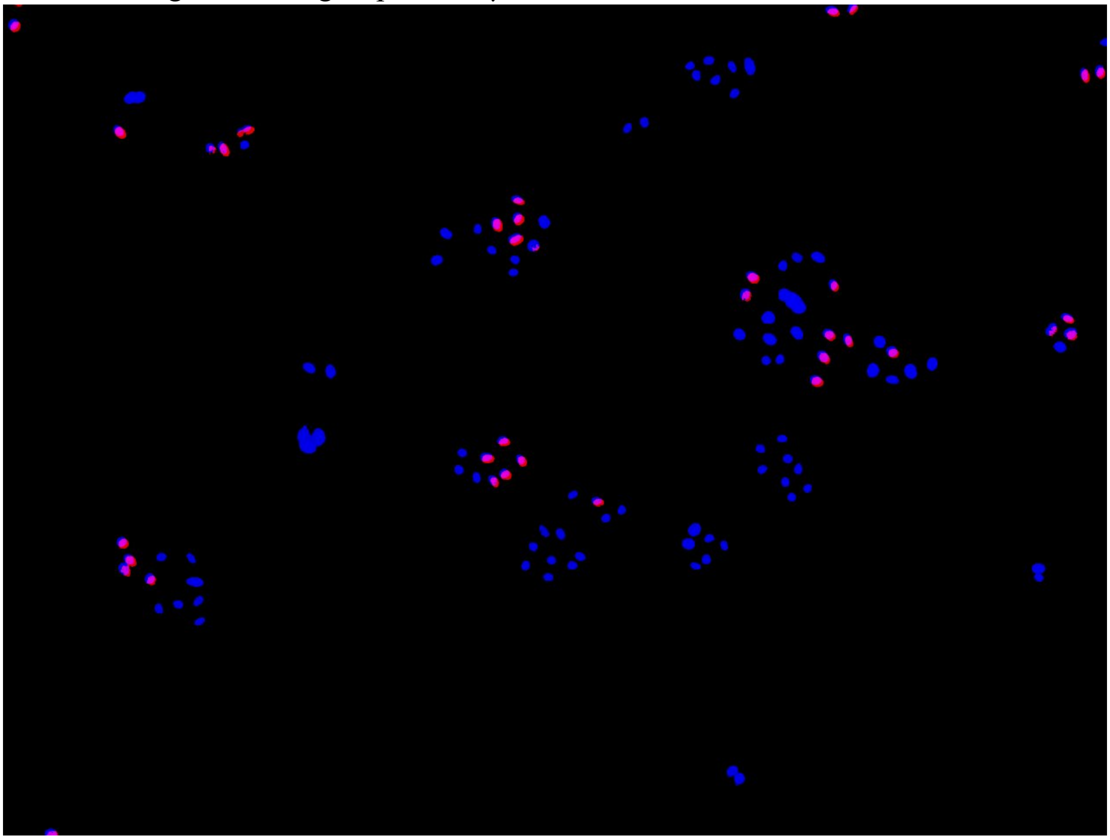

Fig.2B shNC group with 0.013 μM 25-HC in SPC-A1 cells

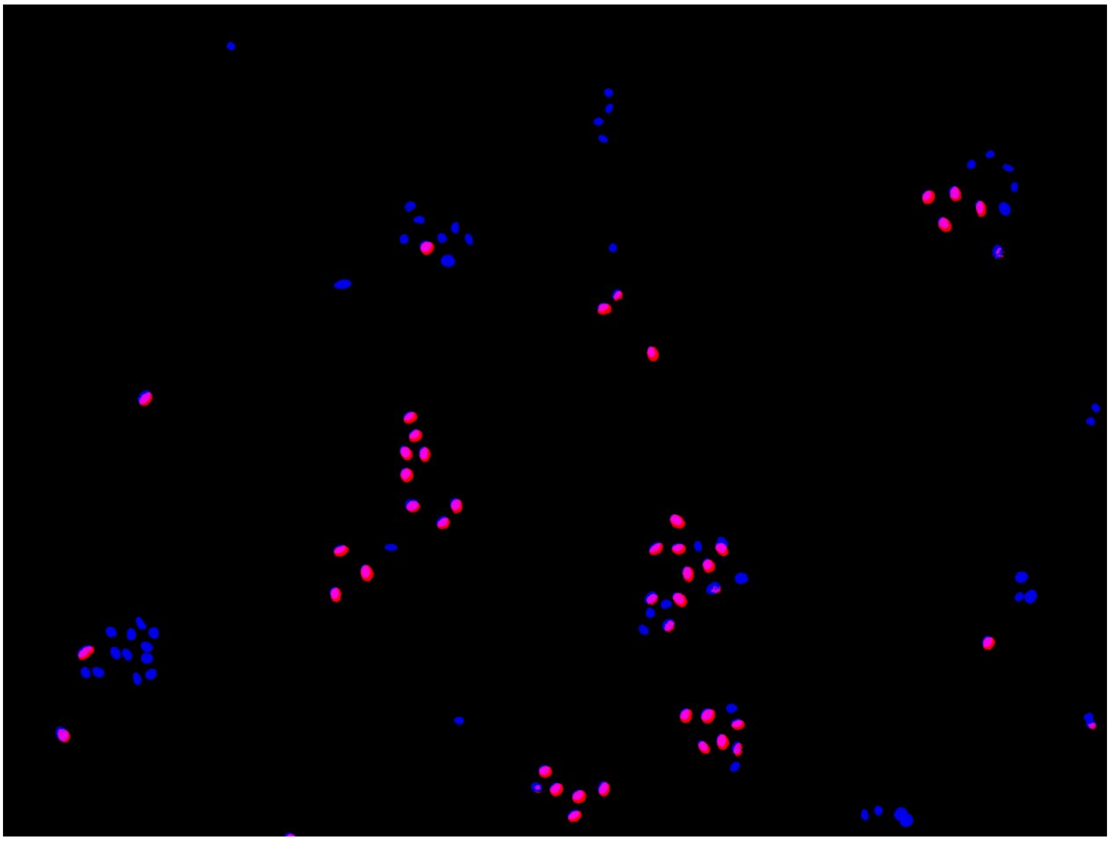

Fig.2B shNC group with 0.085 μM 25-HC in SPC-A1 cells

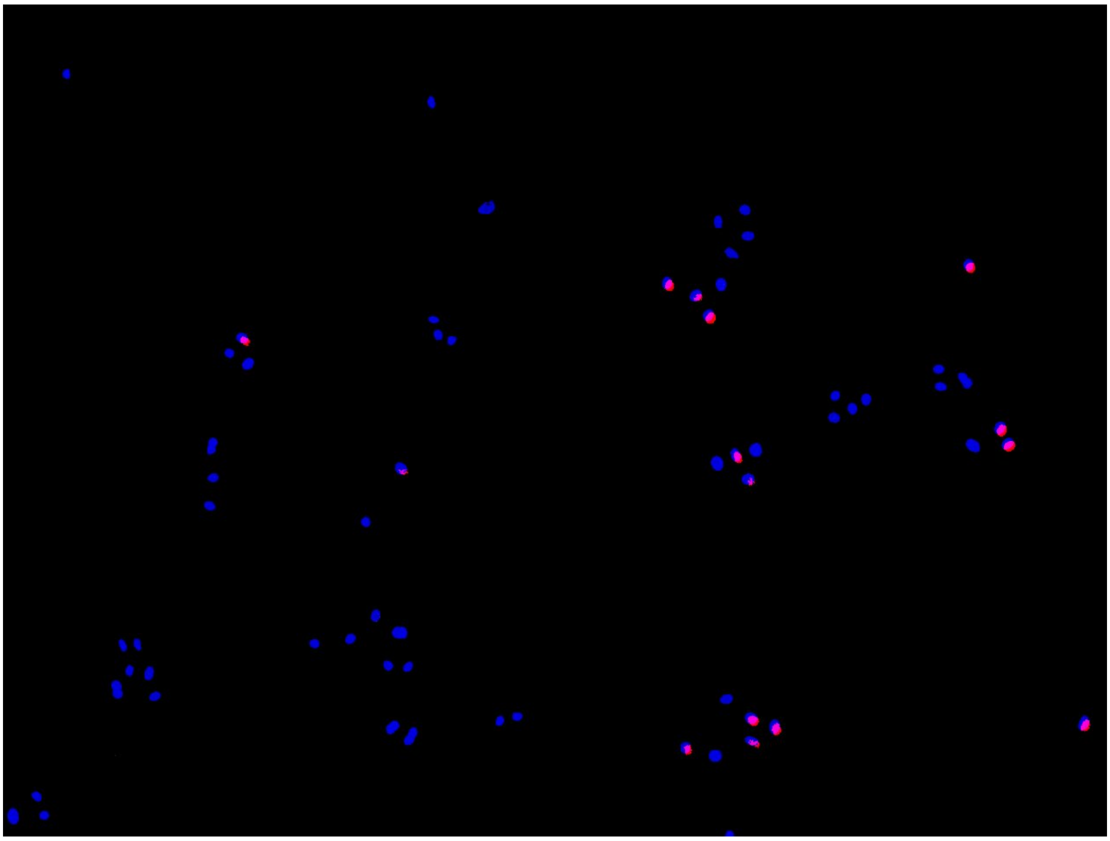

Fig.2B sh ERβ group with 0 μM 25-HC in SPC-A1 cells

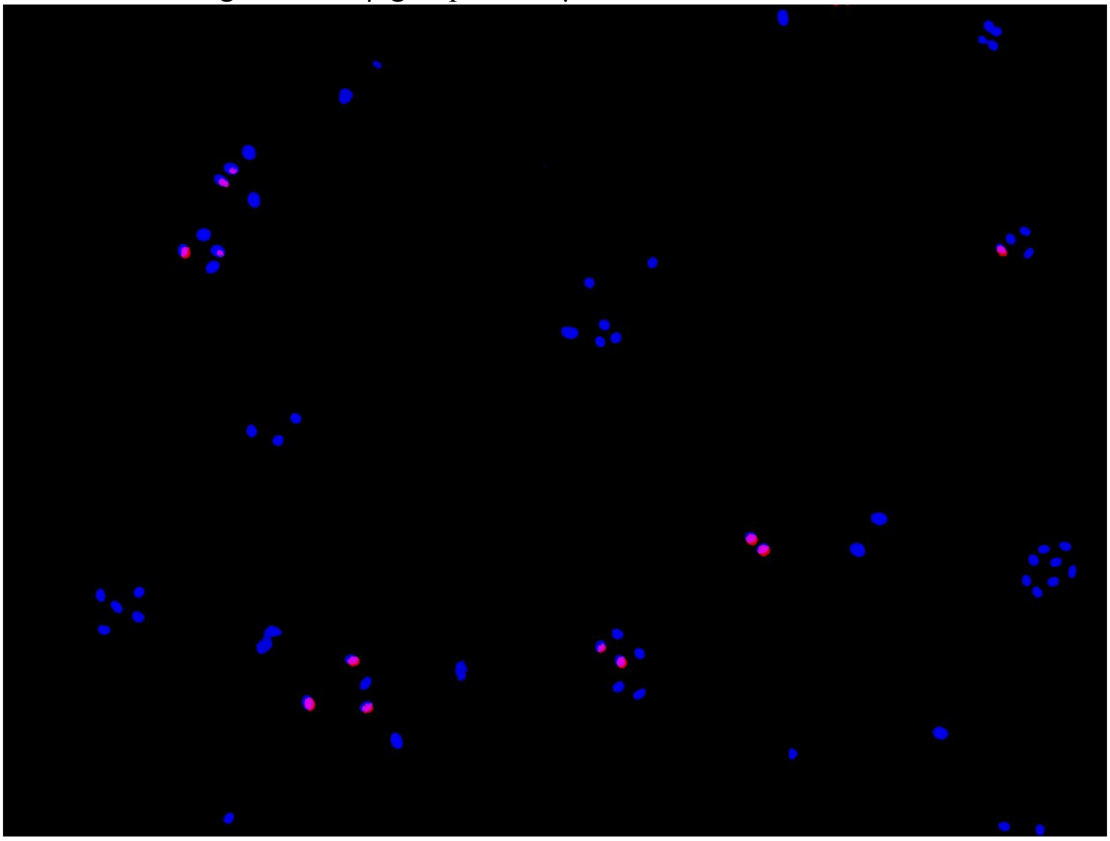

Fig.2B sh ERβ group with 0.013 μM 25-HC in SPC-A1 cells

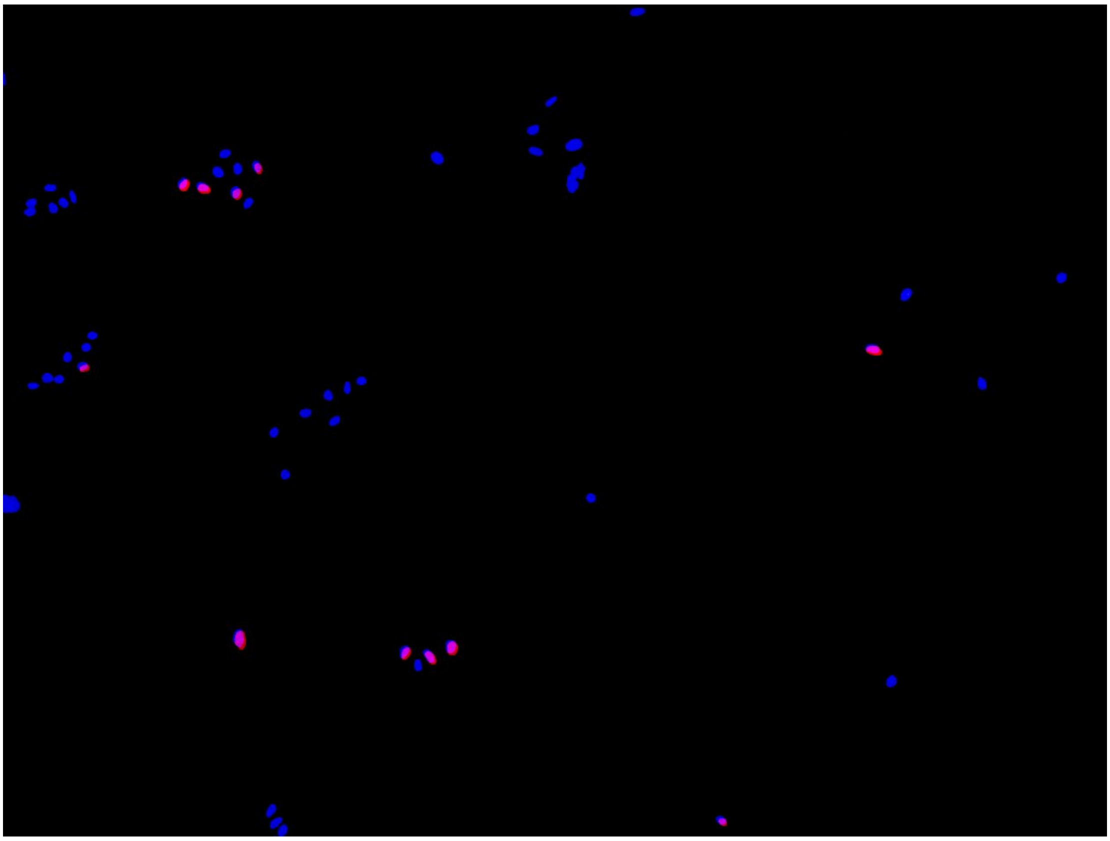

Fig.2B sh ERβ group with 0.085 μM 25-HC in SPC-A1 cells

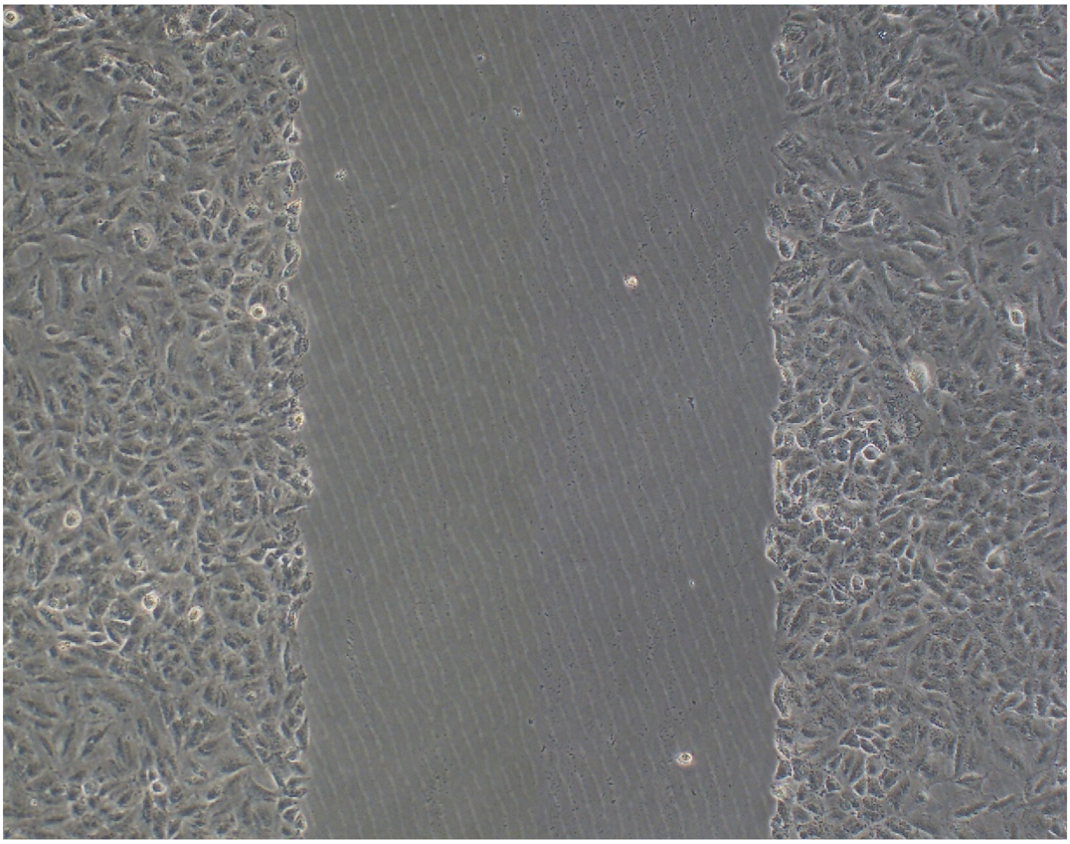

Fig.3A sh NC group with 0 μM 25-HC in A549 cells 0h

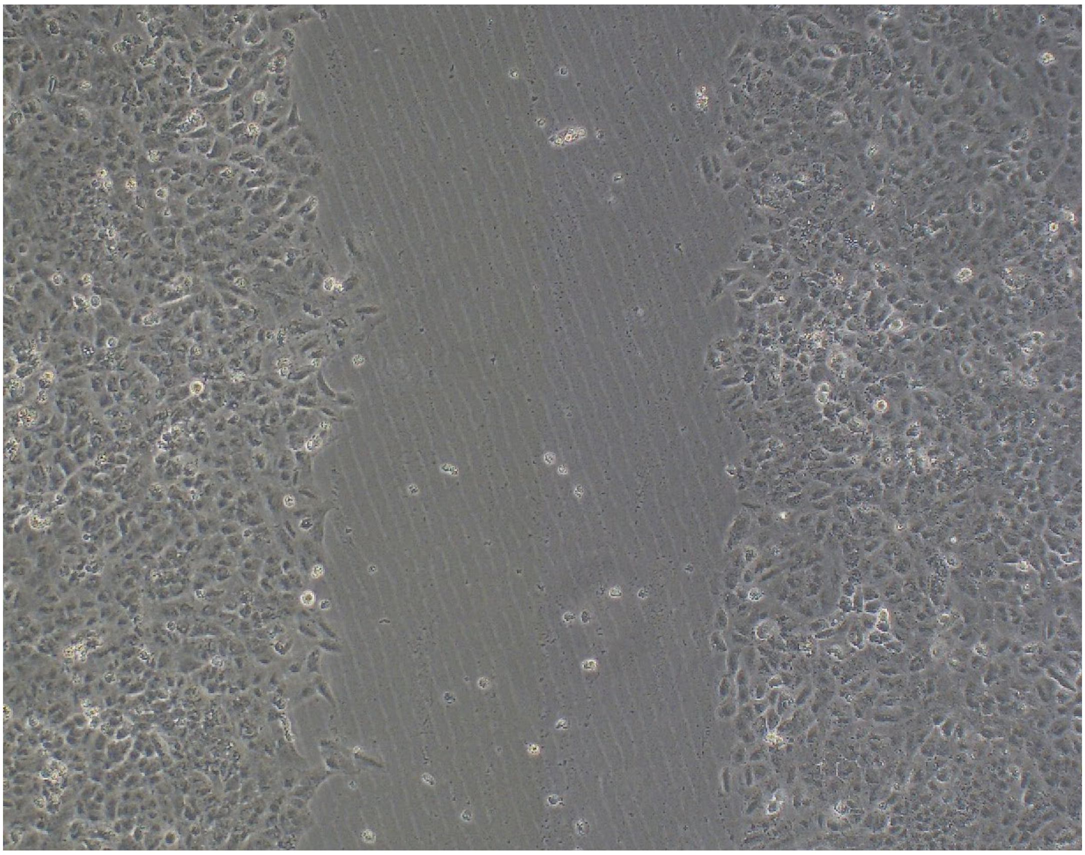

Fig.3A sh NC group with 0 μM 25-HC in A549 cells 48h

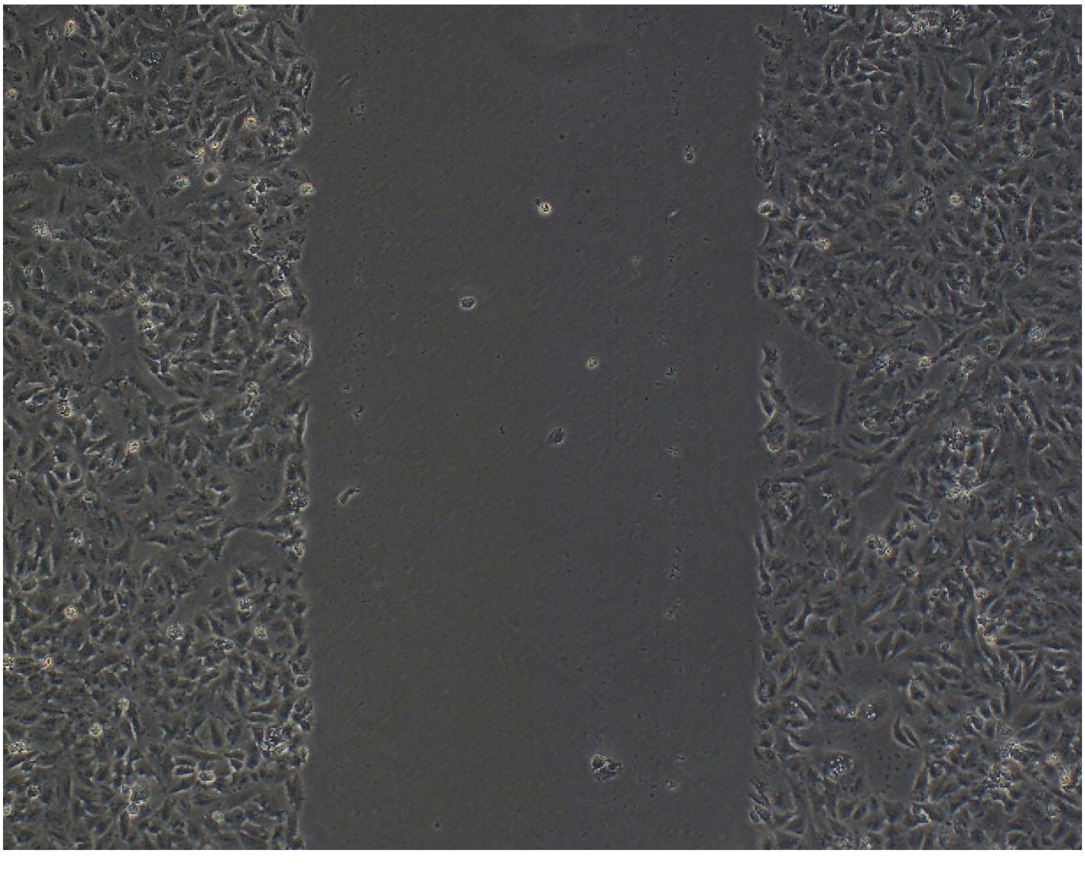

Fig.3A sh NC group with 0.013 μM 25-HC in A549 cells 0h

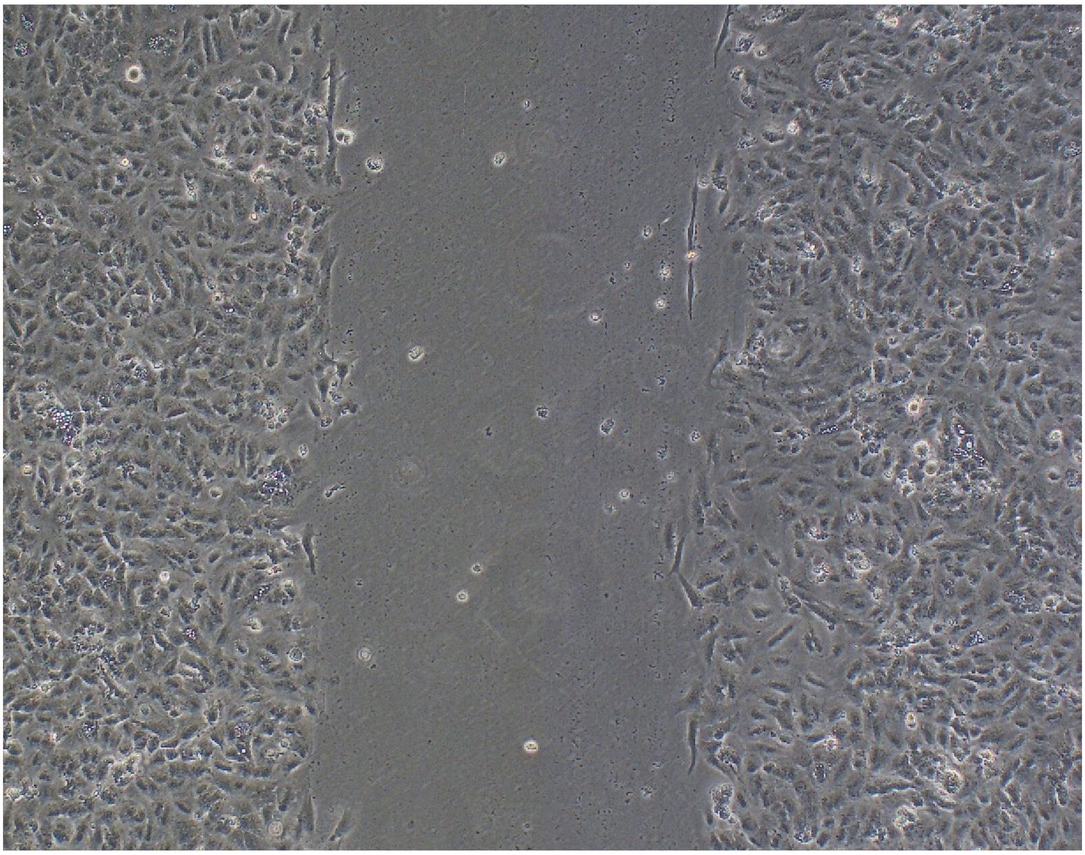

Fig.3A sh NC group with 0.013 μM 25-HC in A549 cells 48h

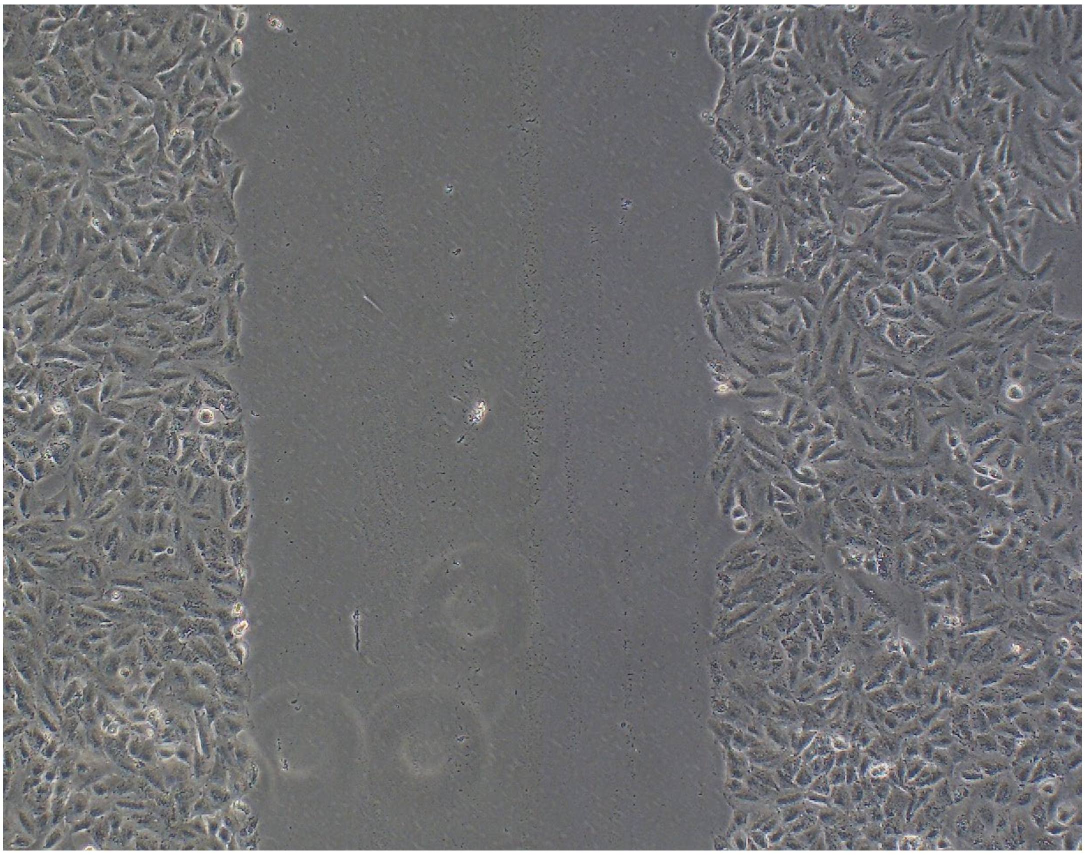

Fig.3A sh NC group with 0.085 μM 25-HC in A549 cells 0h

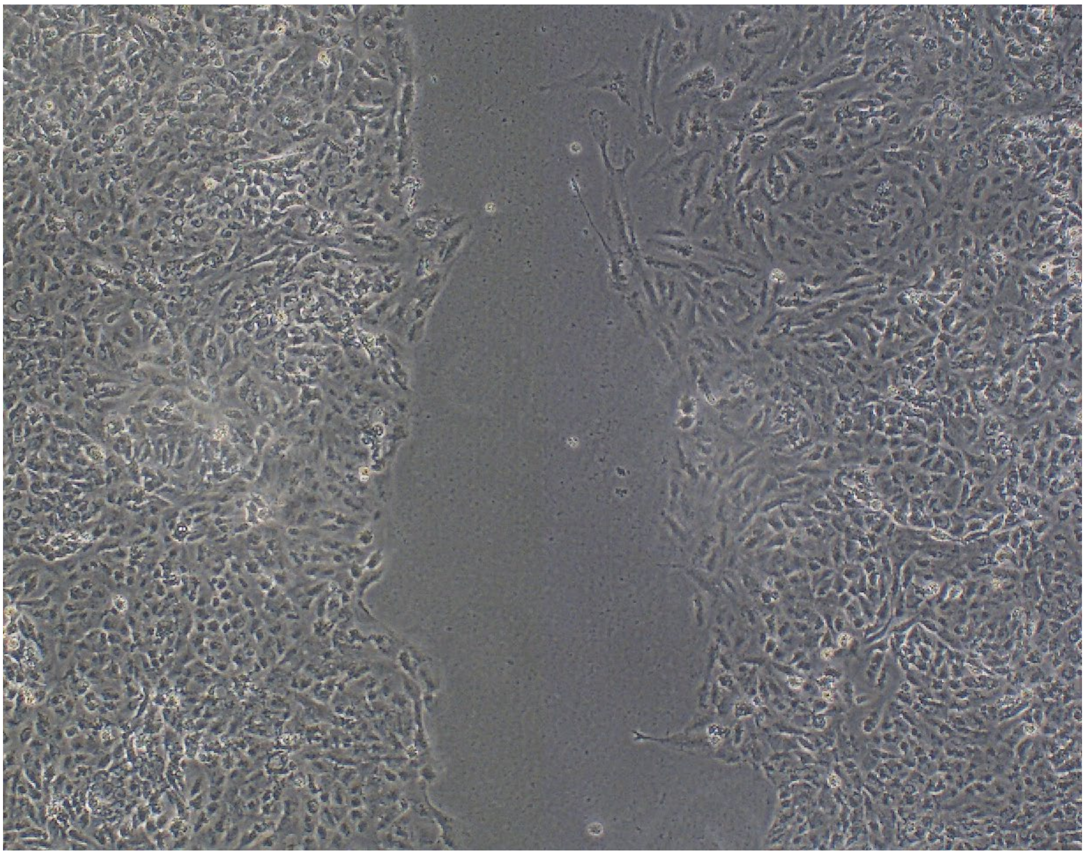

Fig.3A sh NC group with 0.085 μM 25-HC in A549 cells 48h

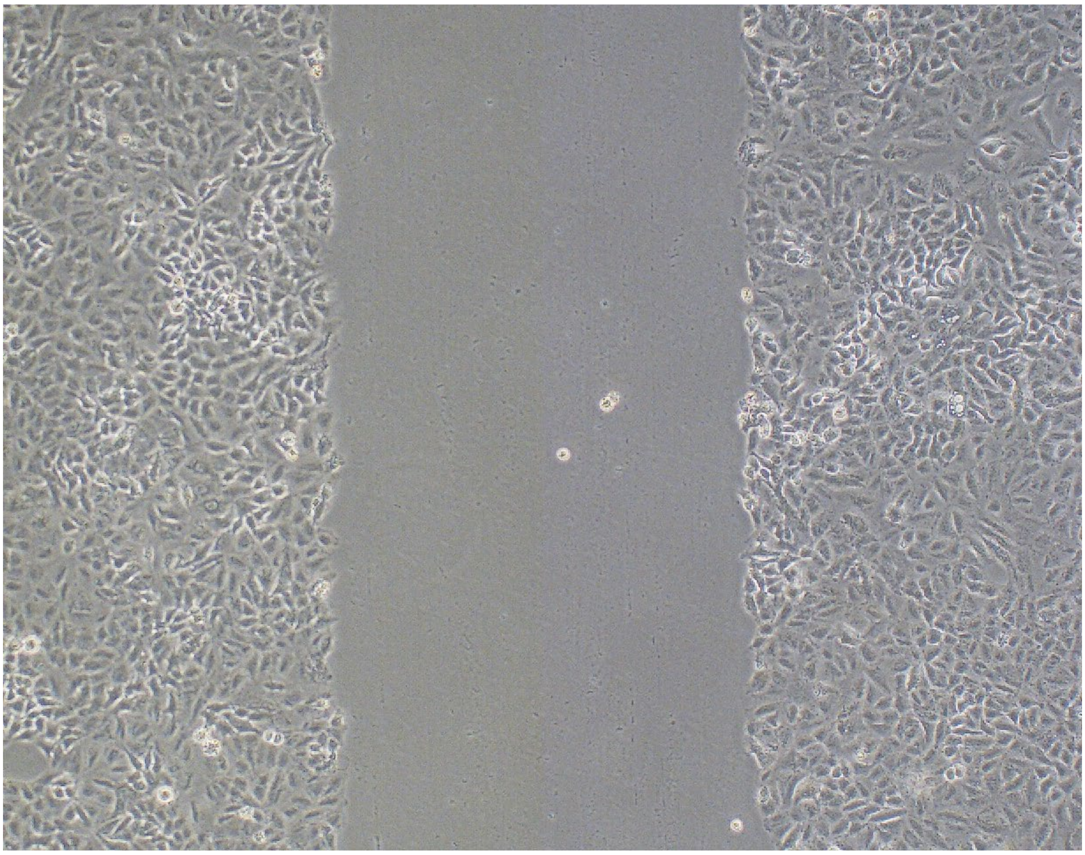

Fig.3A sh ERβ group with 0 μM 25-HC in A549 cells 0h

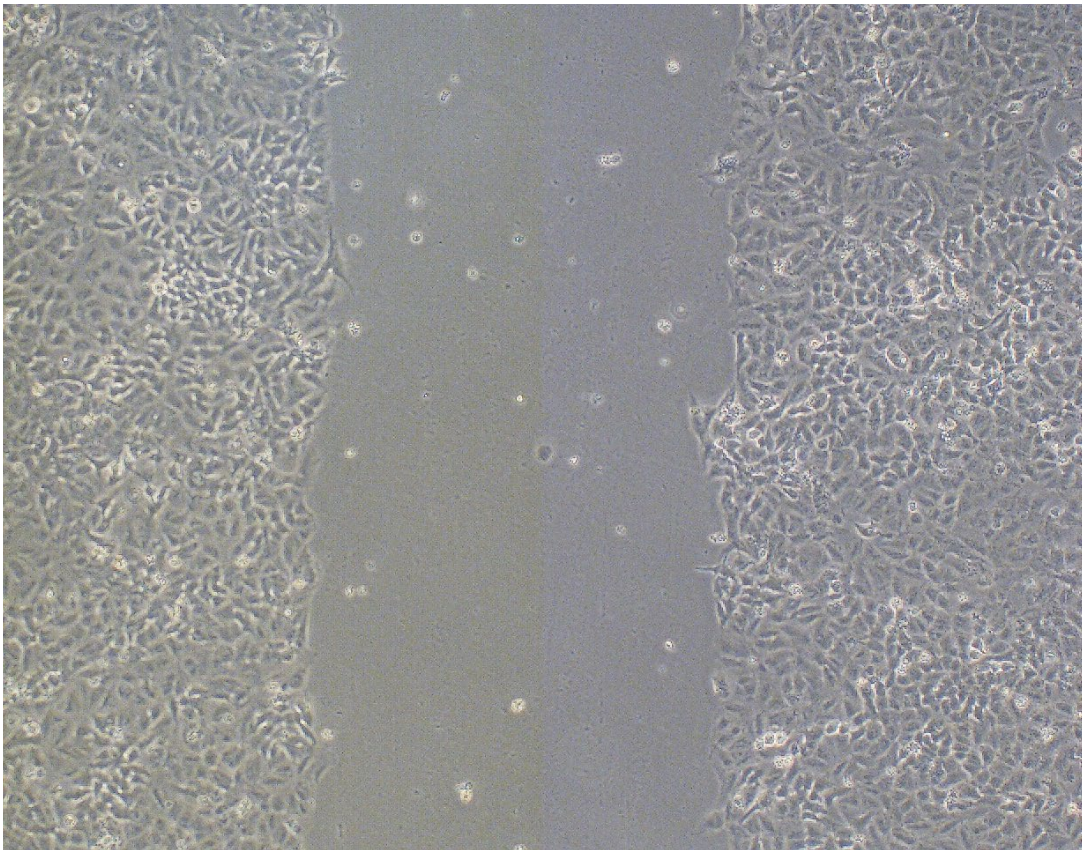

Fig.3A sh ERβ group with 0 μM 25-HC in A549 cells 48h

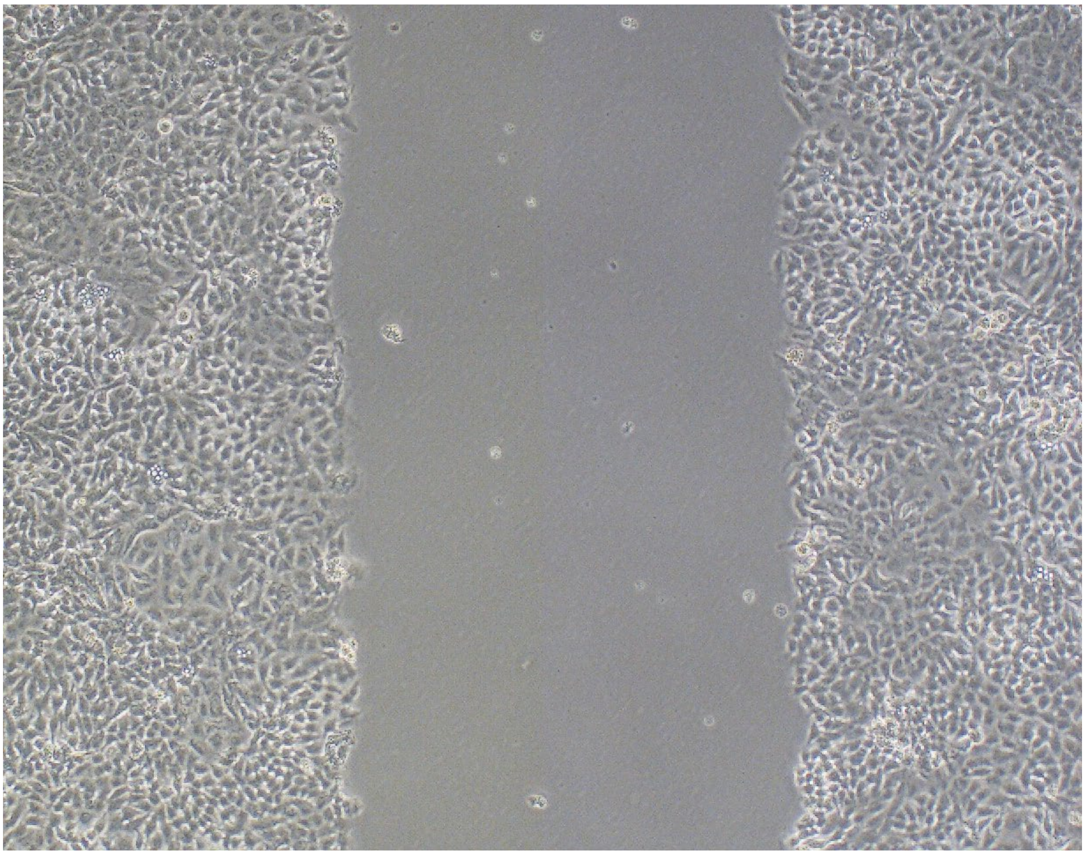

Fig.3A sh ERβ group with 0.013 μM 25-HC in A549 cells 0h

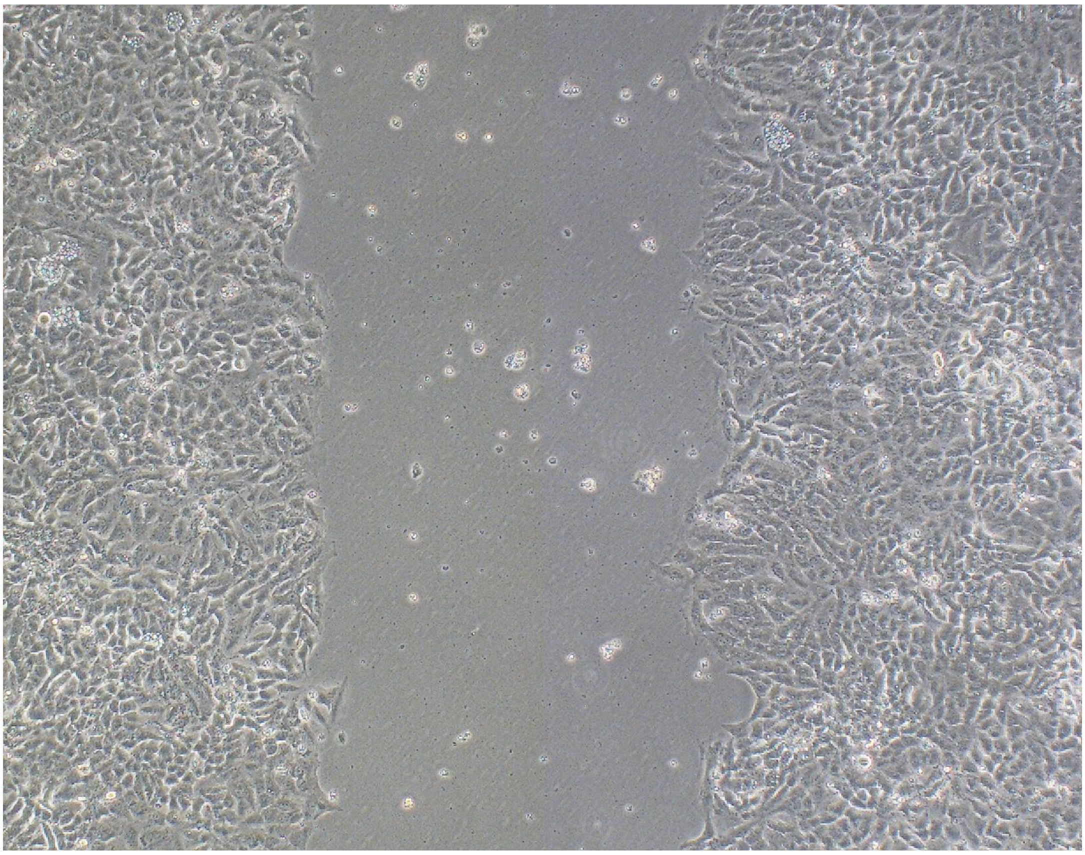

Fig.3A sh ERβ group with 0.013 μM 25-HC in A549 cells 48h

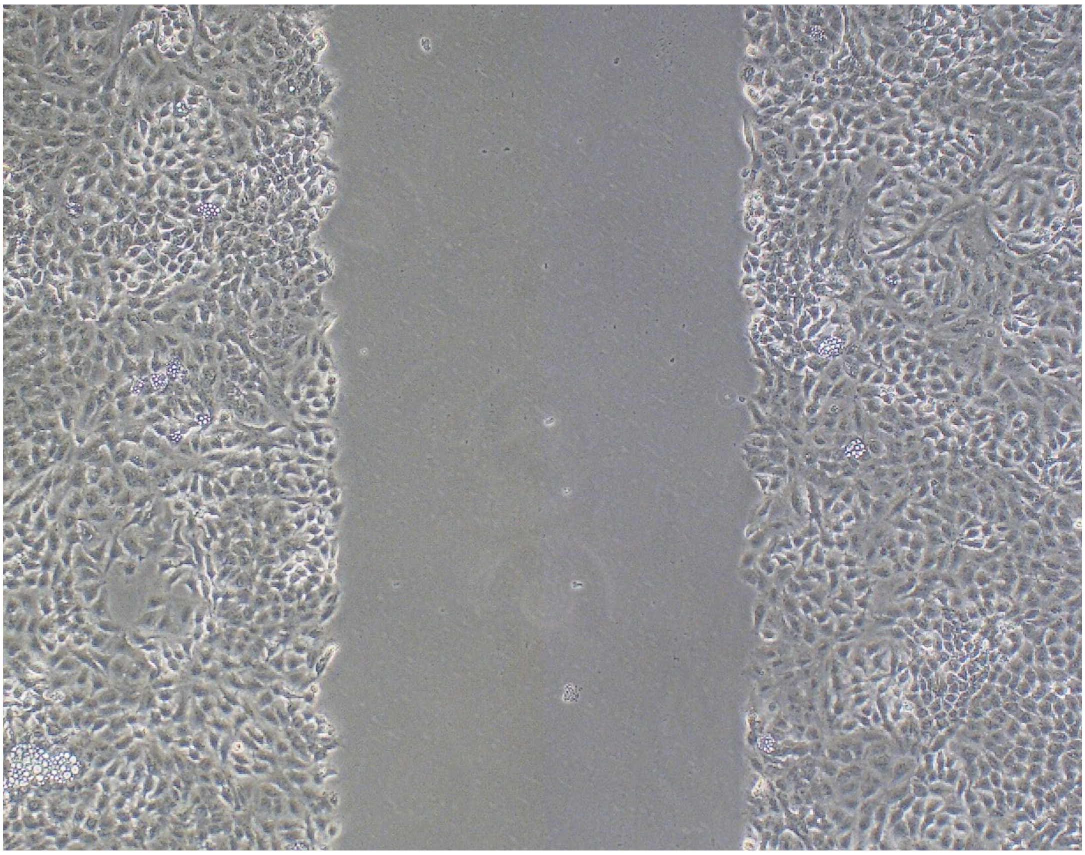

Fig.3A sh ERβ group with 0.085 μM 25-HC in A549 cells 0h

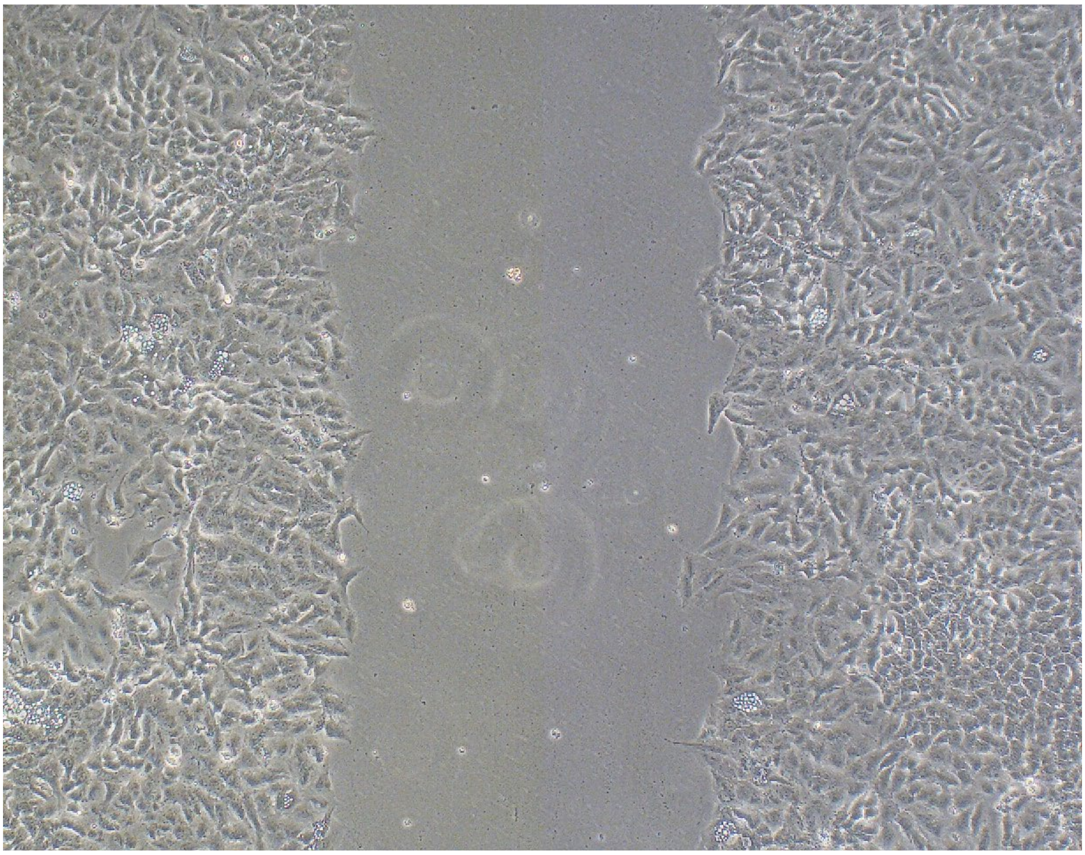

Fig.3A sh ERβ group with 0.085 μM 25-HC in A549 cells 48h

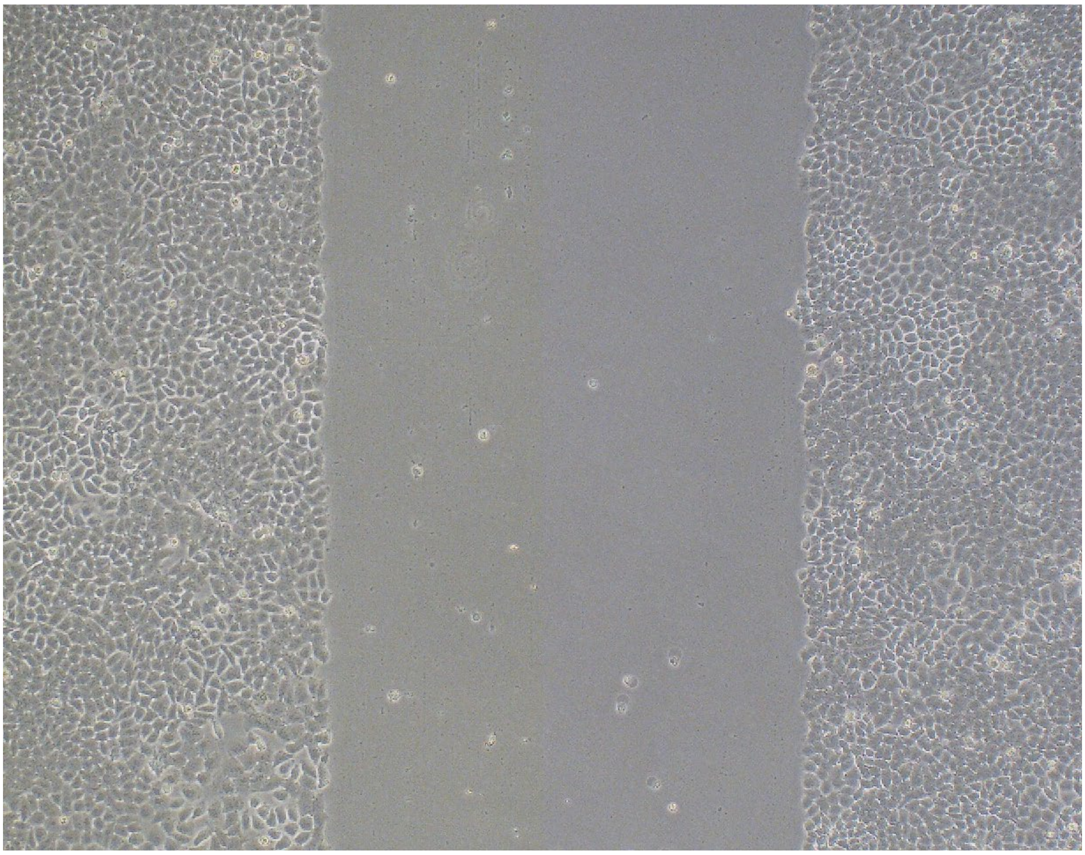

Fig.3A sh NC group with 0 μM 25-HC in SPC-A1 cells 0h

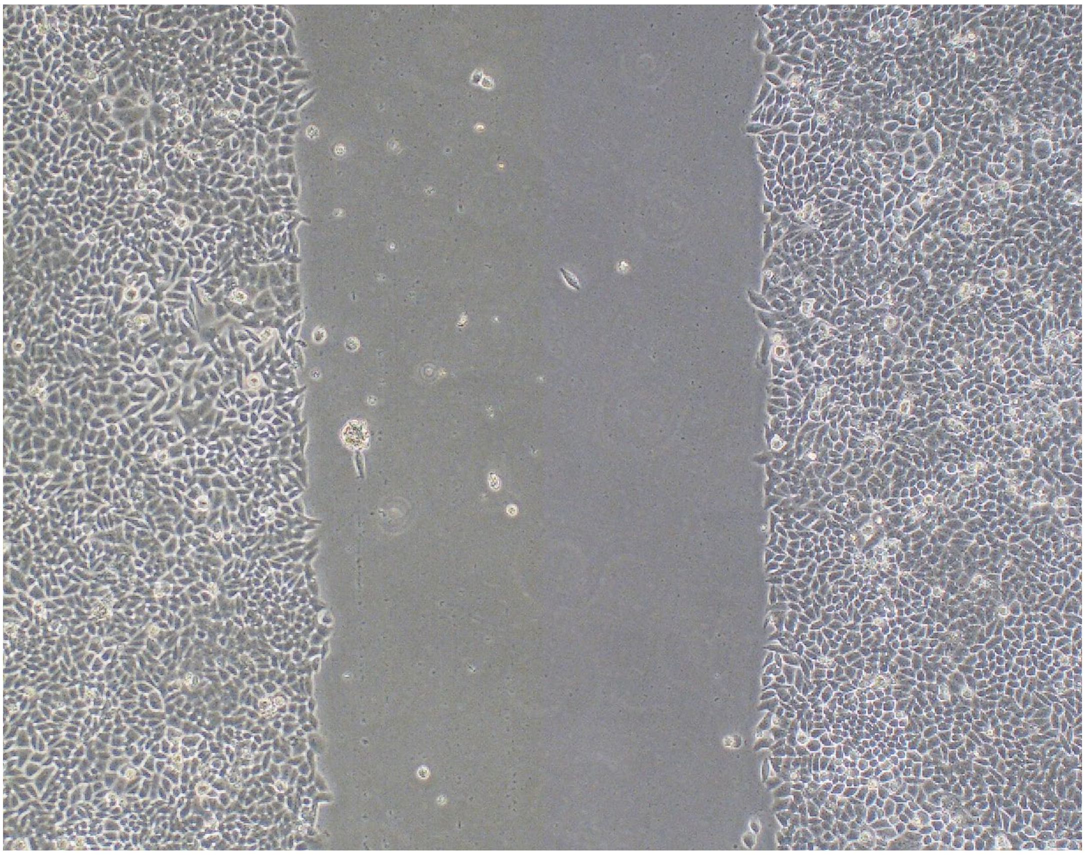

Fig.3A sh NC group with 0 μM 25-HC in SPC-A1 cells 48h

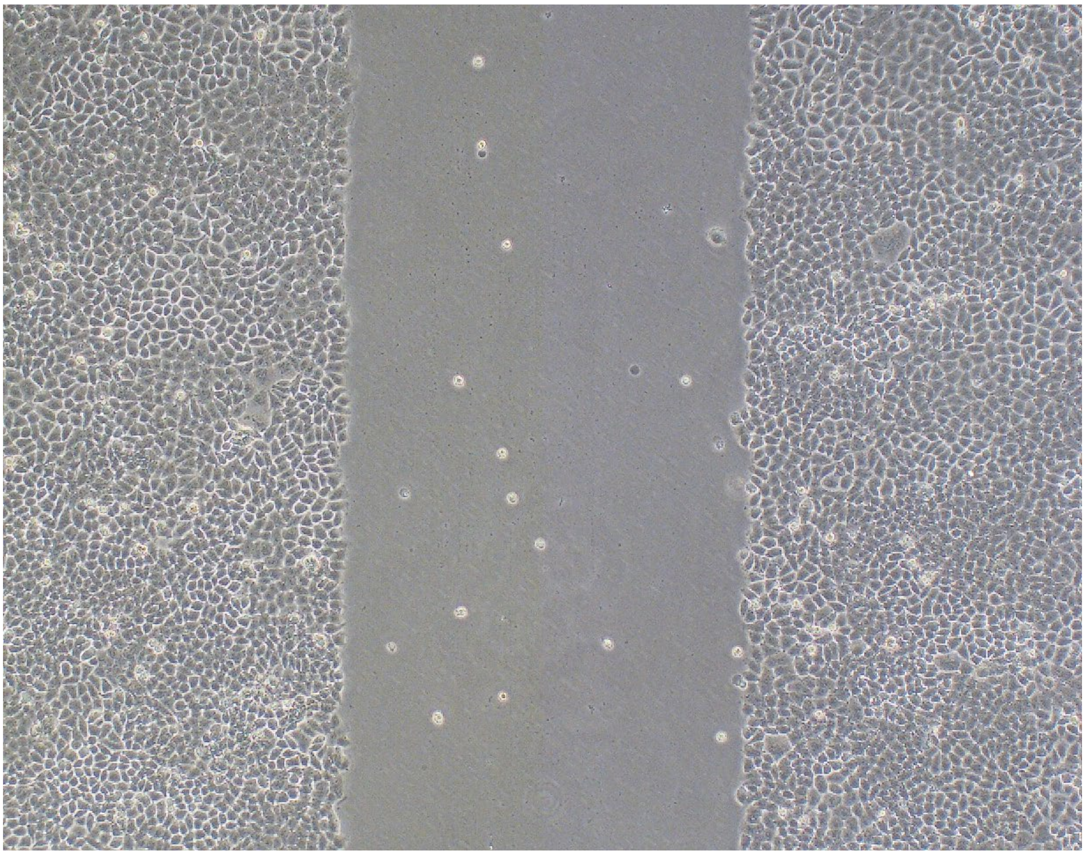

Fig.3A sh NC group with 0.013 μM 25-HC in SPC-A1 cells 0h

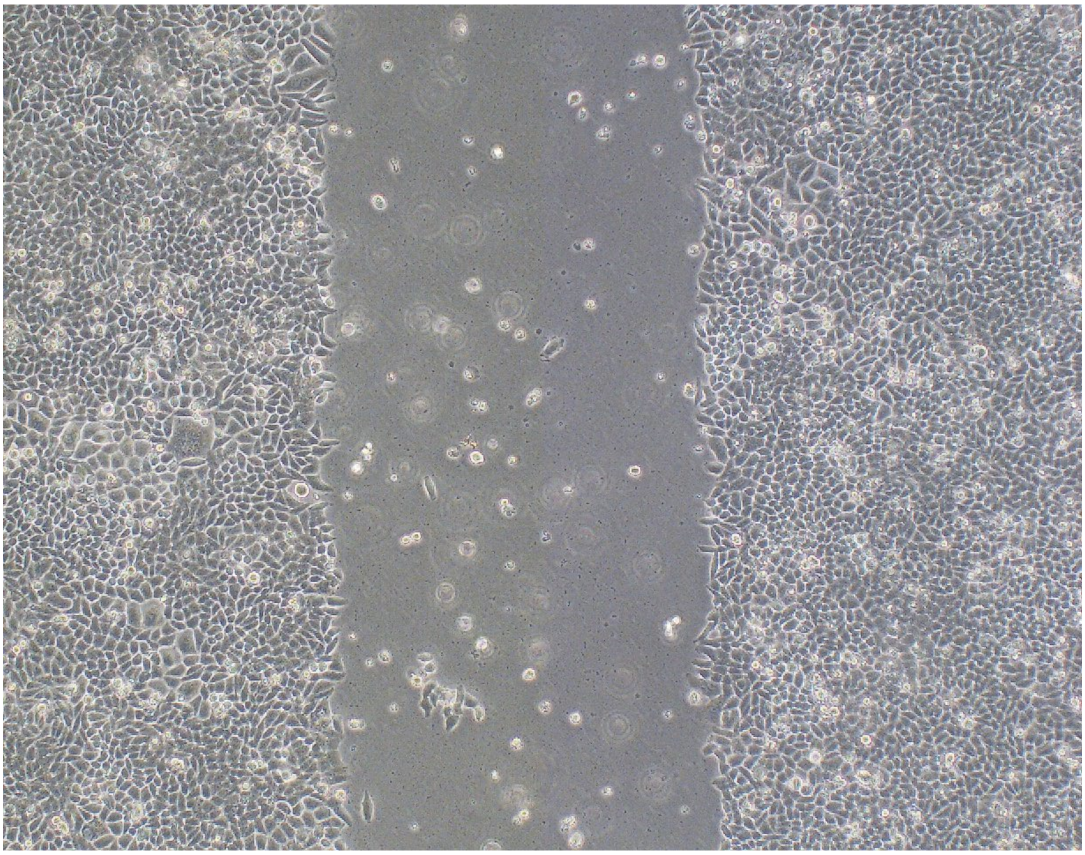

Fig.3A sh NC group with 0.013 μM 25-HC in SPC-A1 cells 48h

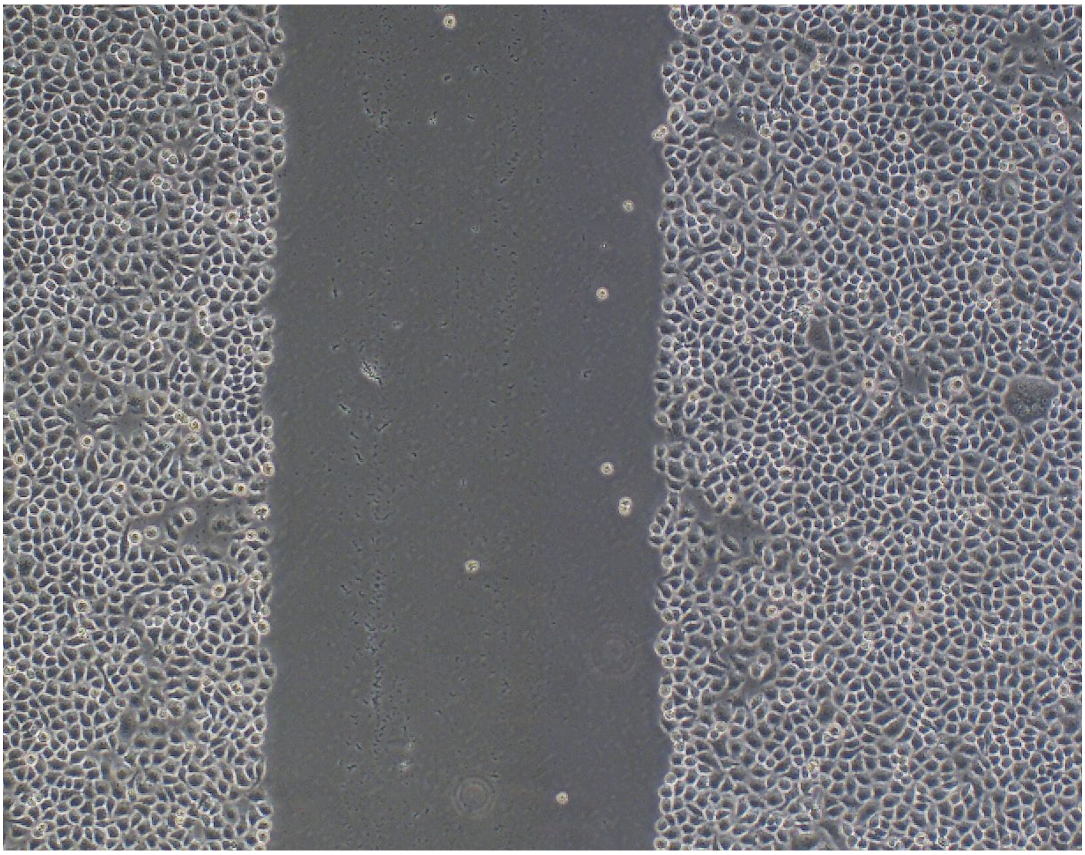

Fig.3A sh NC group with 0.085 μM 25-HC in SPC-A1 cells 0h

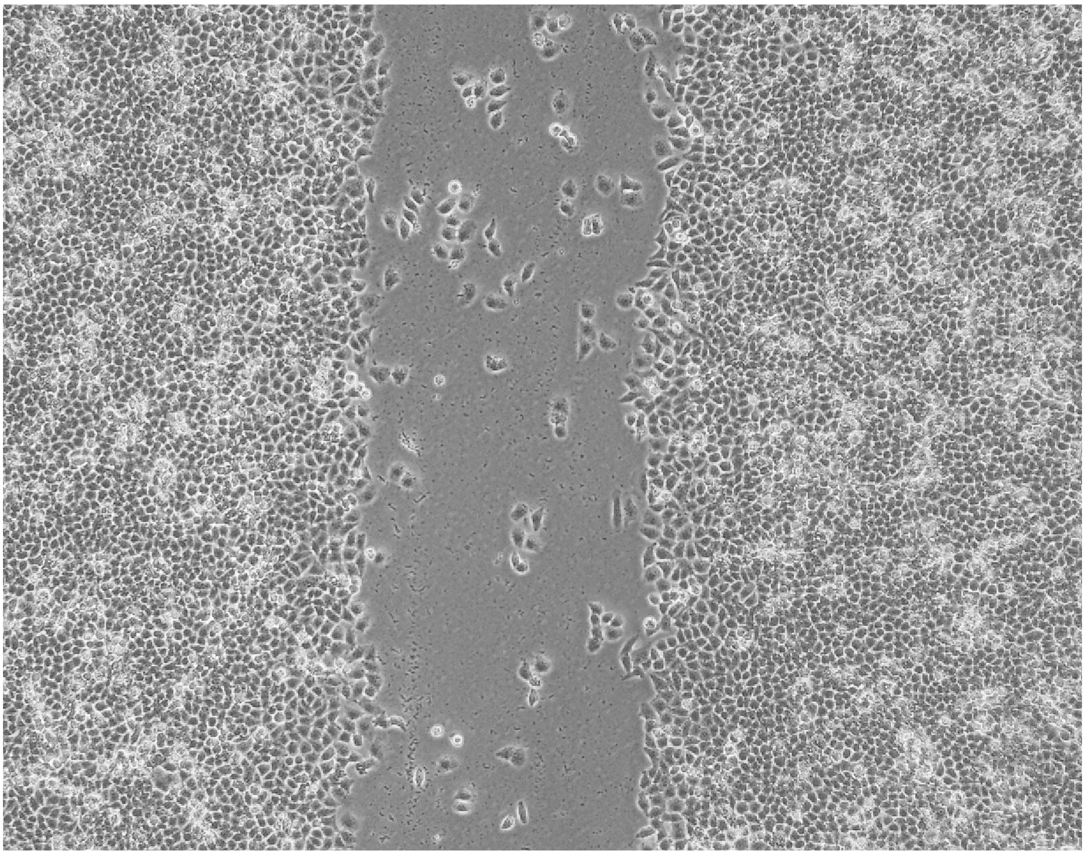

Fig.3A sh NC group with 0.085 μM 25-HC in SPC-A1 cells 48h

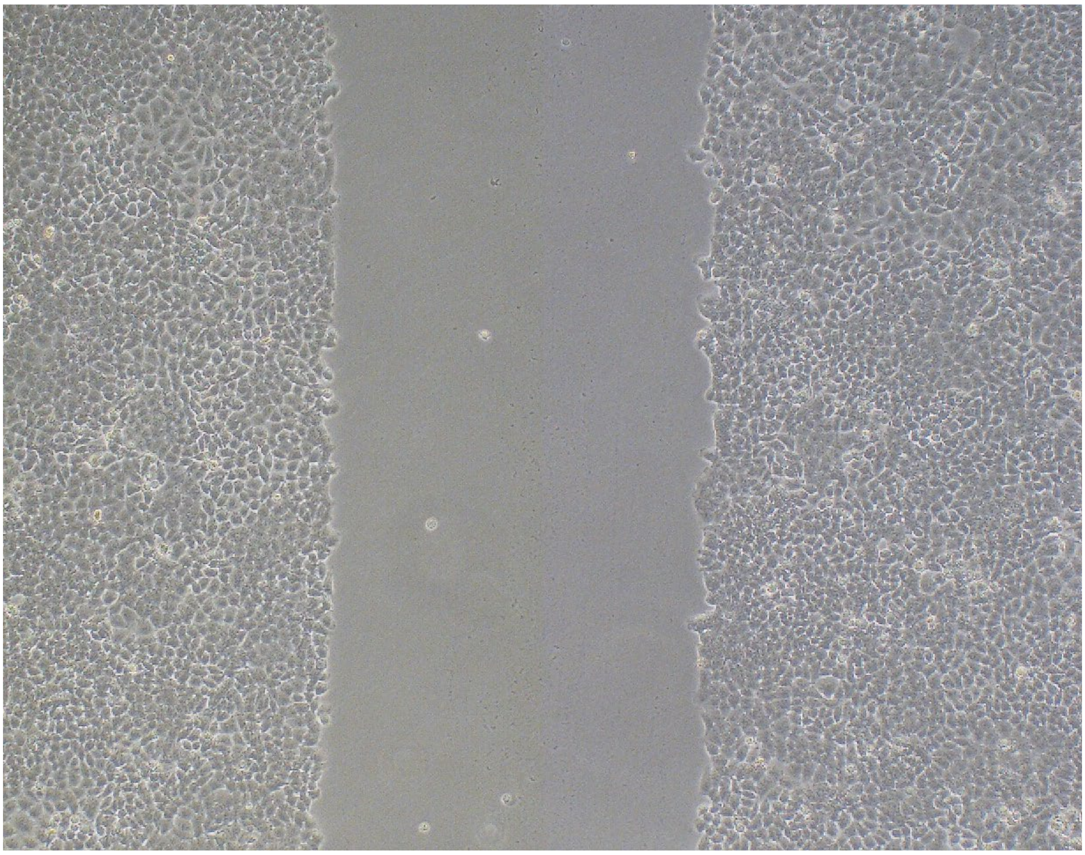

Fig.3A sh ERβ group with 0 μM 25-HC in SPC-A1 cells 0h

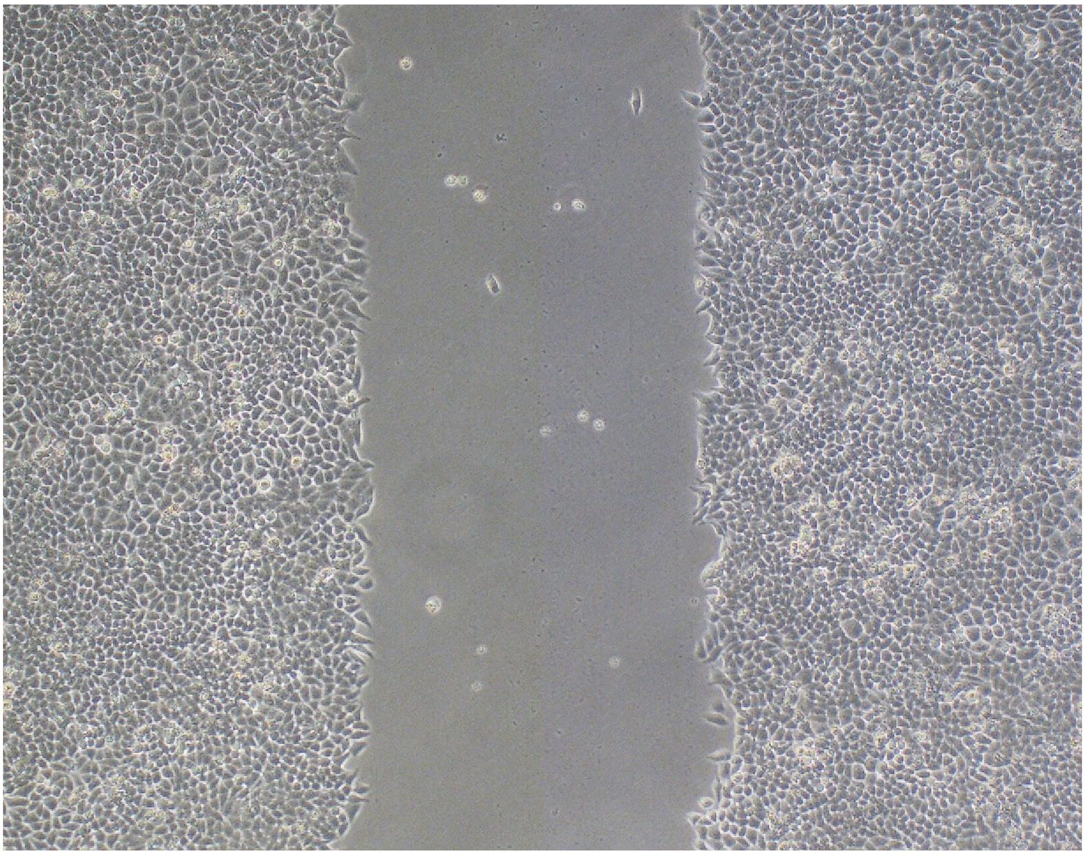

Fig.3A sh ERβ group with 0 μM 25-HC in SPC-A1 cells 48h

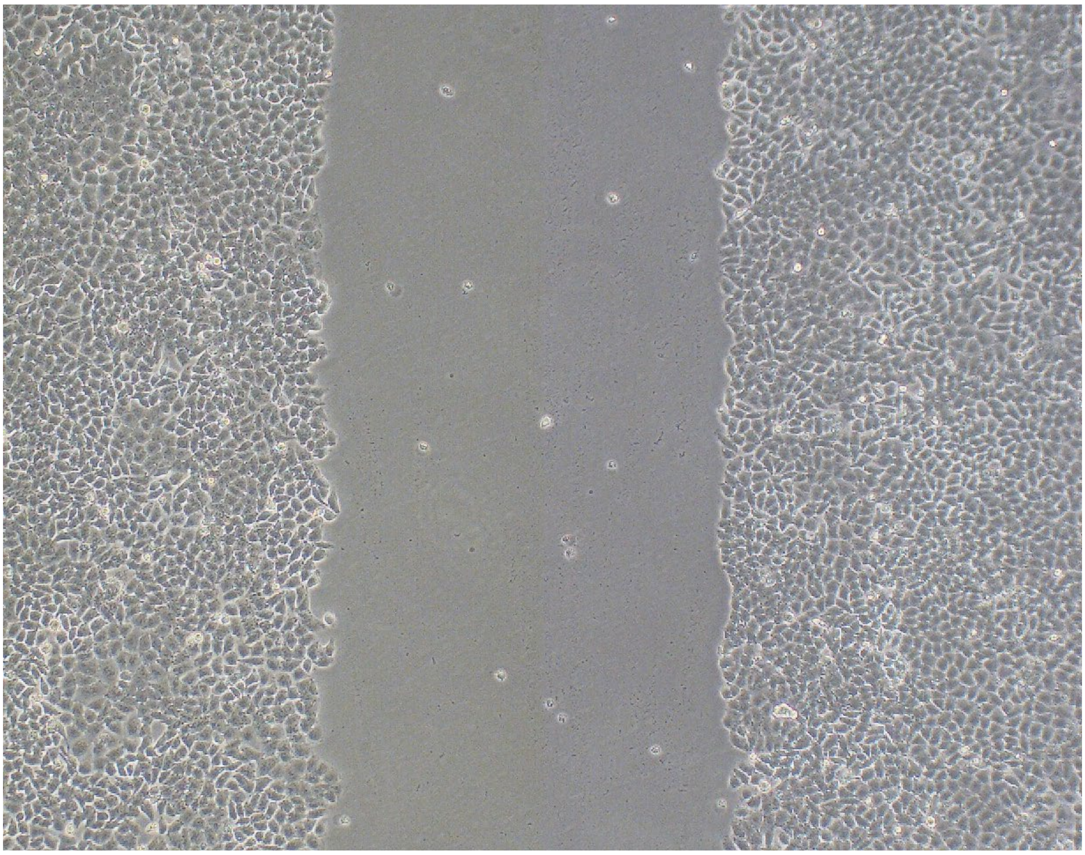

Fig.3A sh ERβ group with 0.013 μM 25-HC in SPC-A1 cells 0h

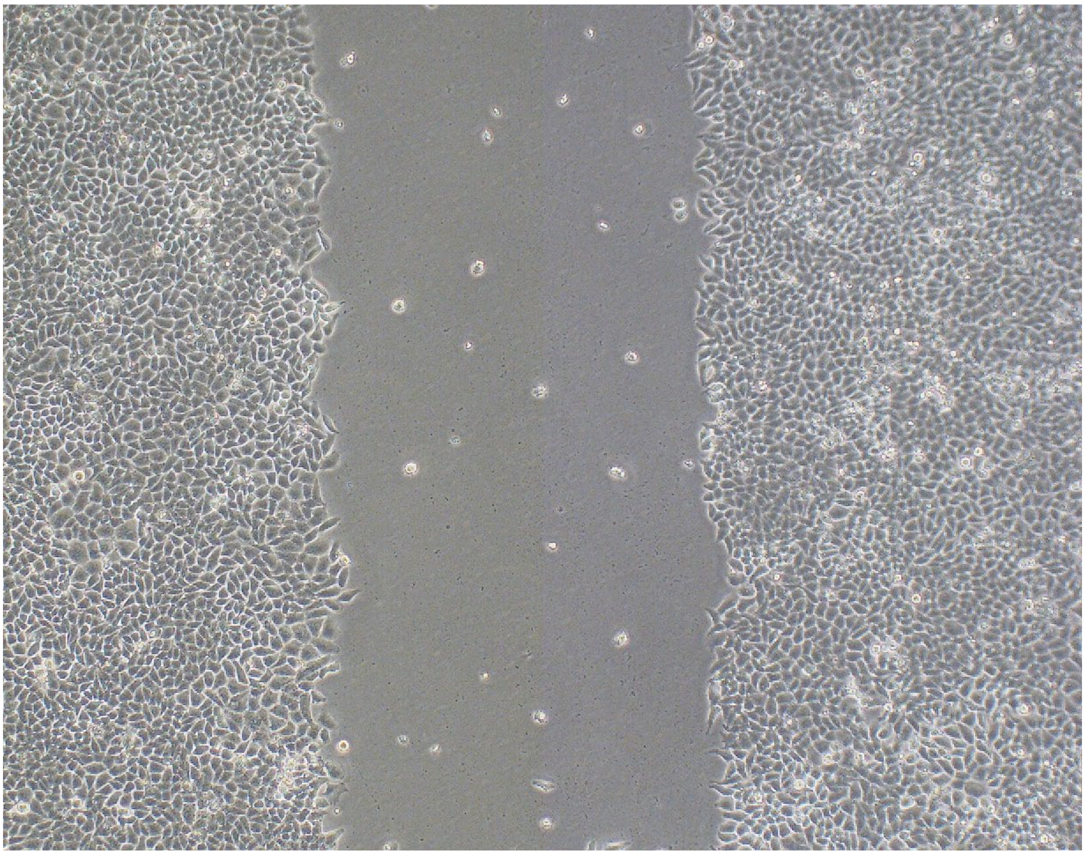

Fig.3A sh ERβ group with 0.013 μM 25-HC in SPC-A1 cells 48h

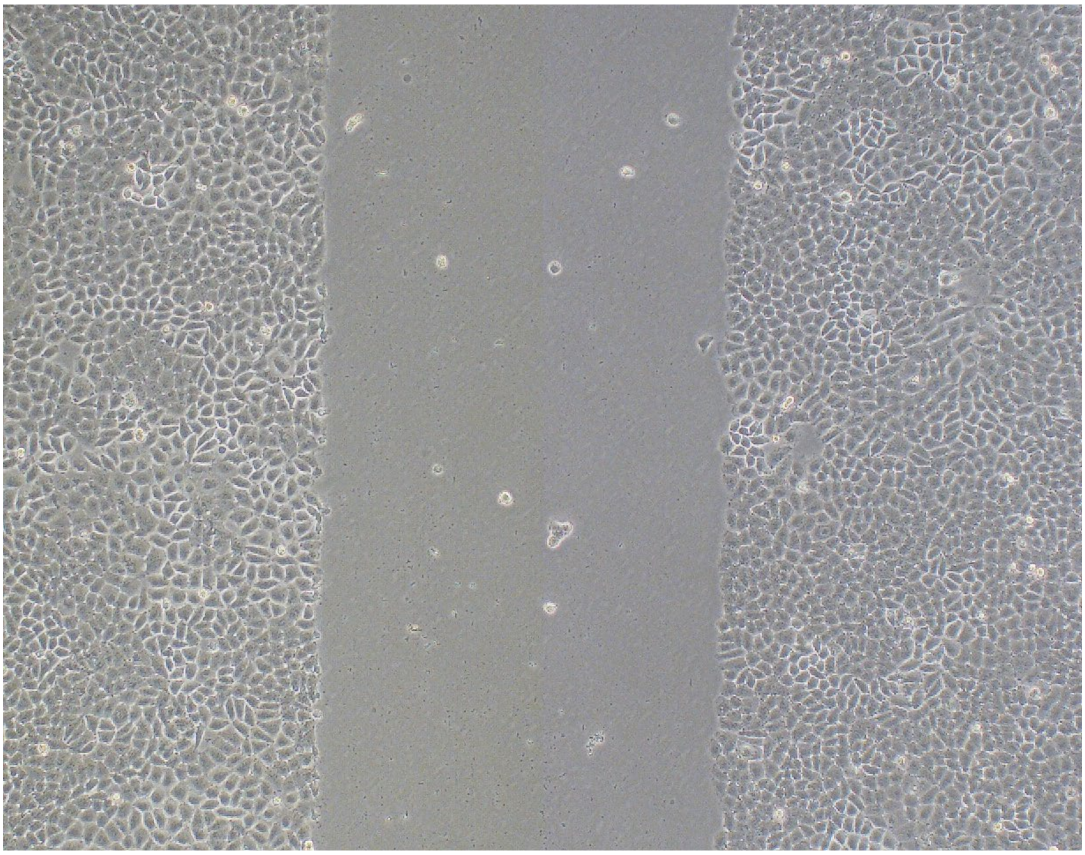

Fig.3A sh ERβ group with 0.085 μM 25-HC in SPC-A1 cells 0h

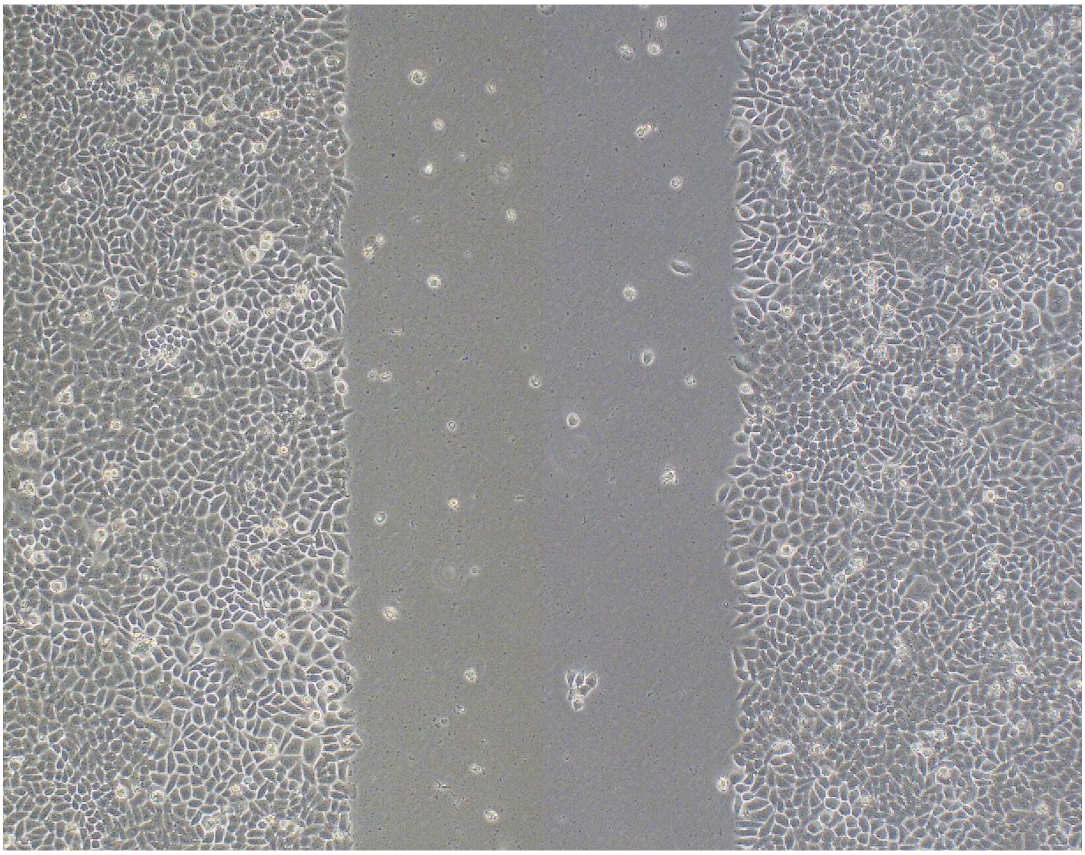

Fig.3A sh ERβ group with 0.085 μM 25-HC in SPC-A1 cells 48h

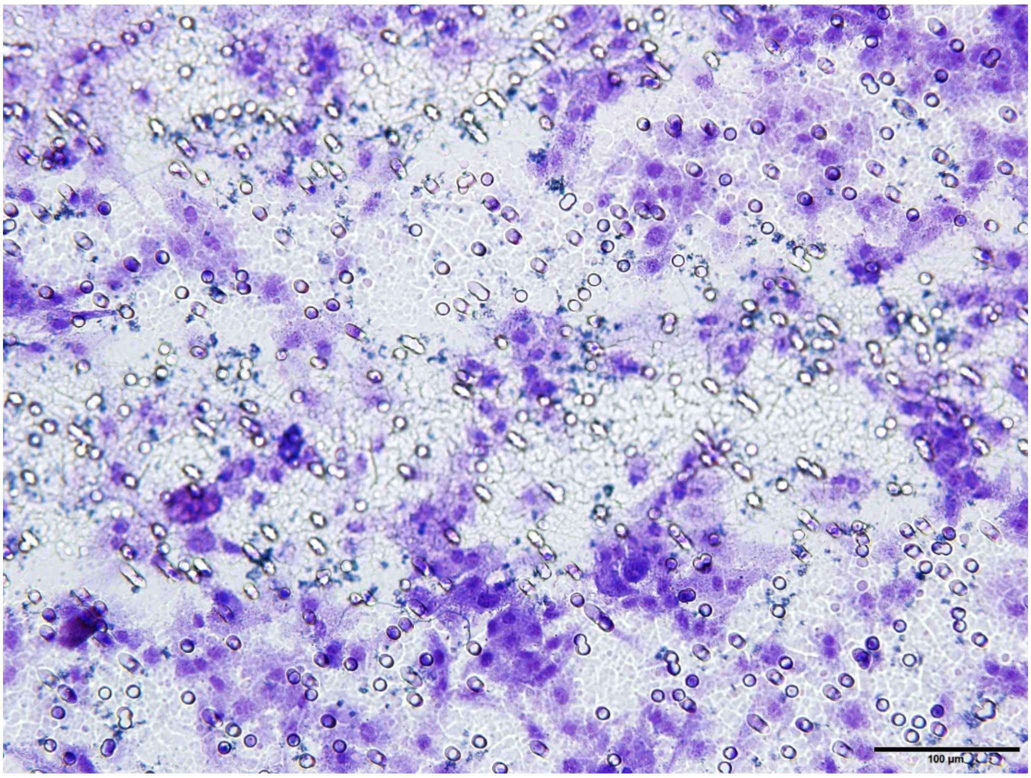

Fig.3B sh NC group with 0 μM 25-HC in A549 cells

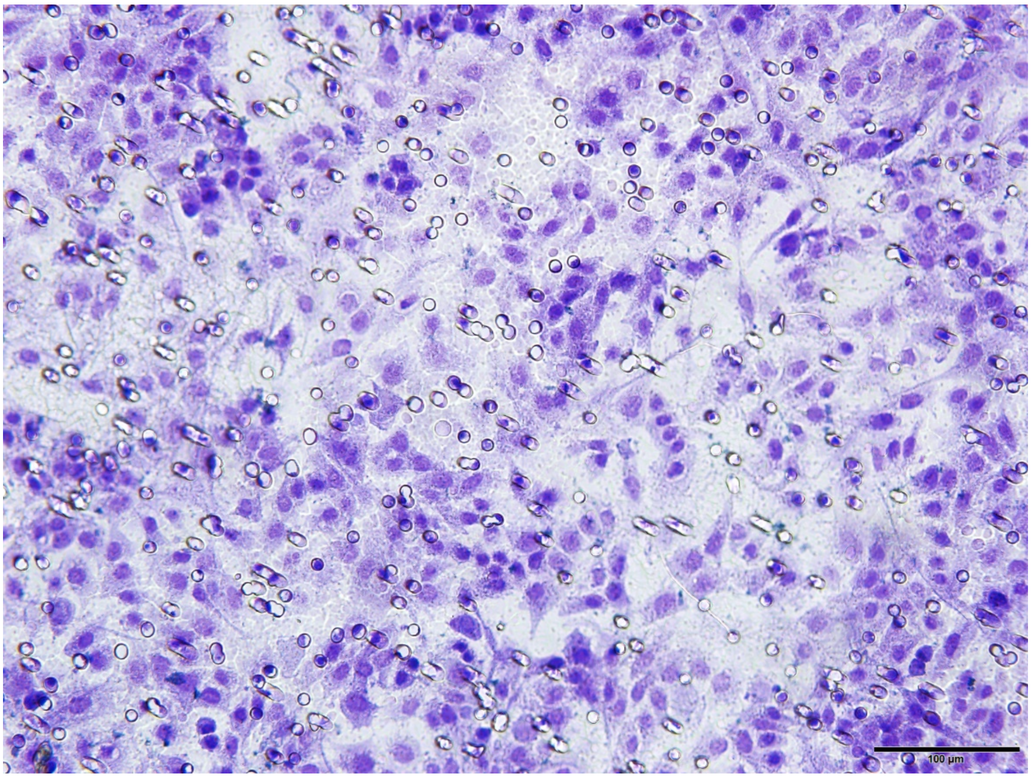

Fig.3B sh NC group with 0.013 μM 25-HC in A549 cells

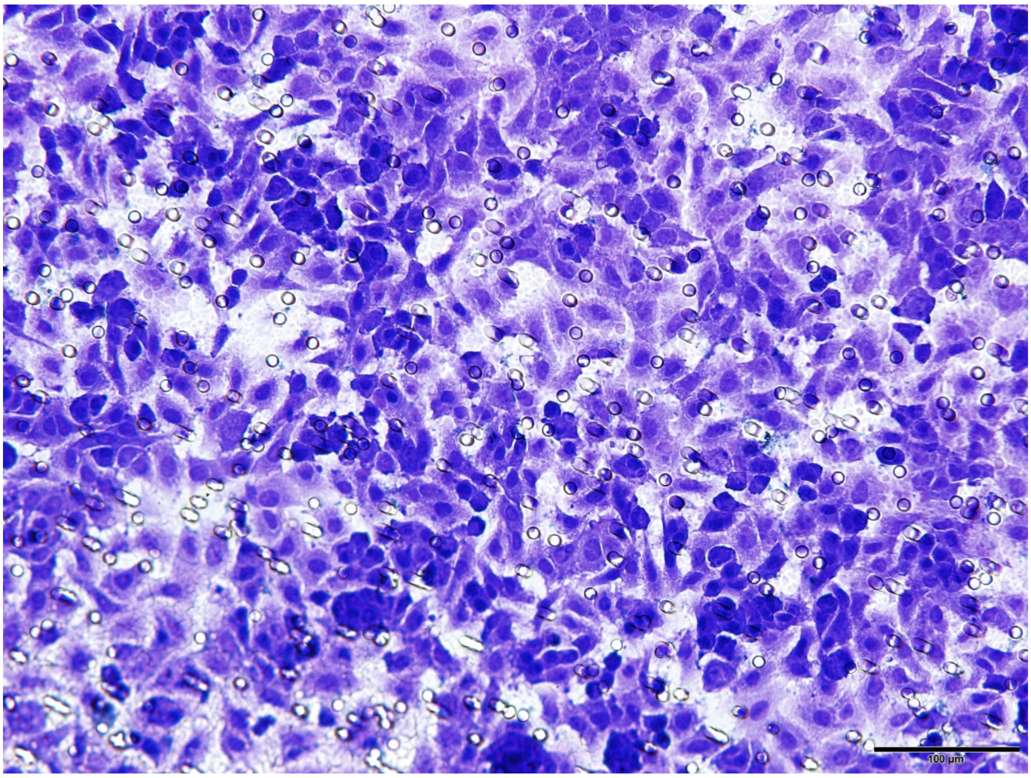

Fig.3B sh NC group with 0.085 μM 25-HC in A549 cells

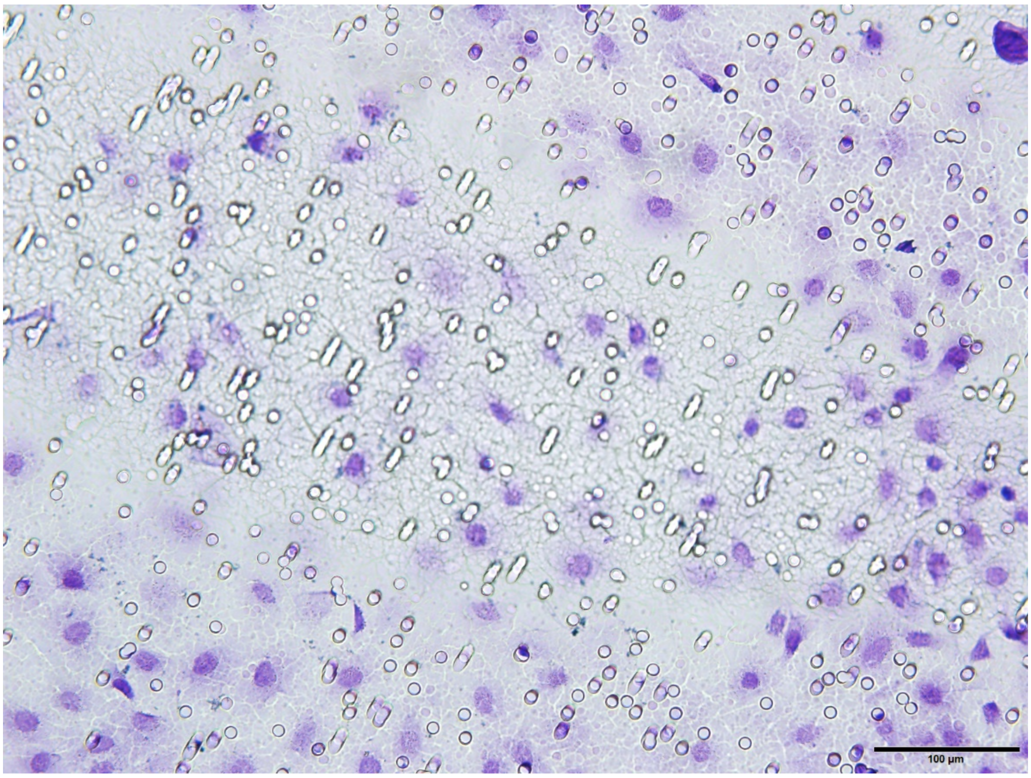

Fig.3B sh ERβ group with 0 μM 25-HC in A549 cells

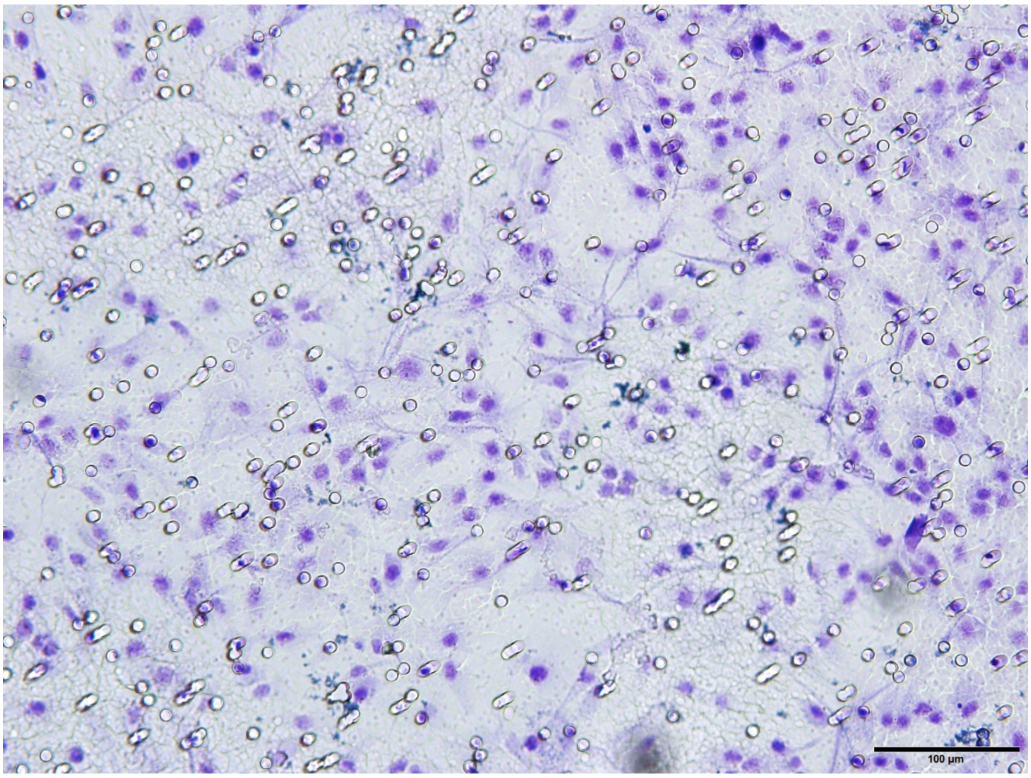

Fig.3B sh ERβ group with 0.013 μM 25-HC in A549 cells

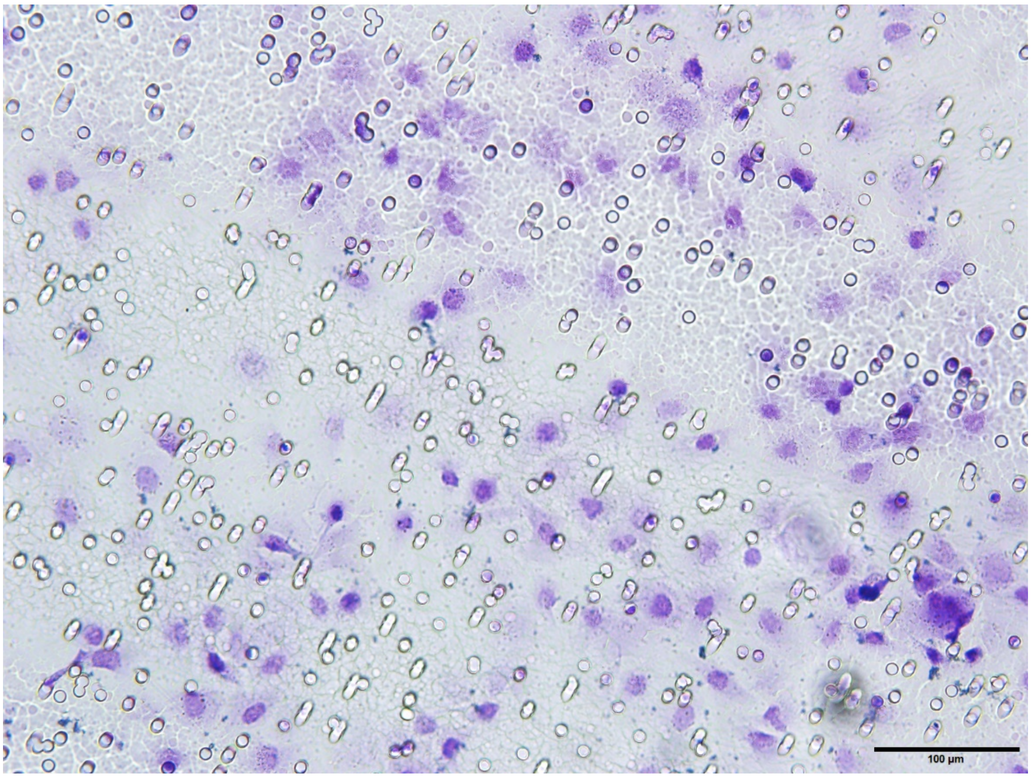

Fig.3B sh ERβ group with 0.085 μM 25-HC in A549 cells

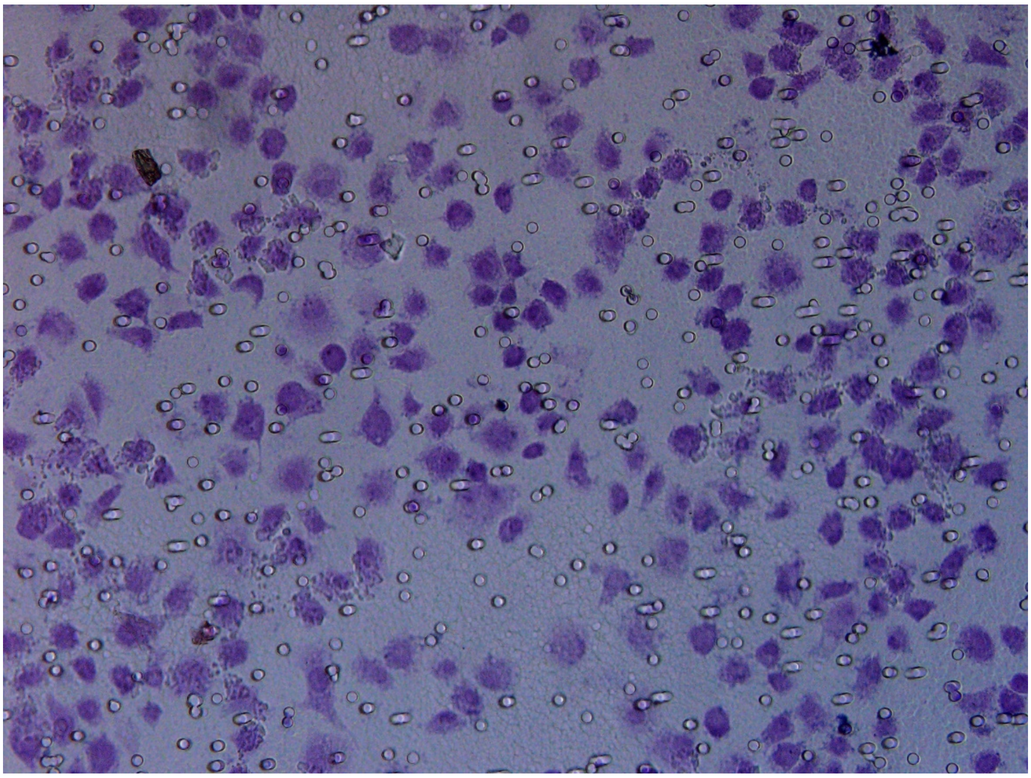

Fig.3B sh NC group with 0 μM 25-HC in SPC-A1 cells

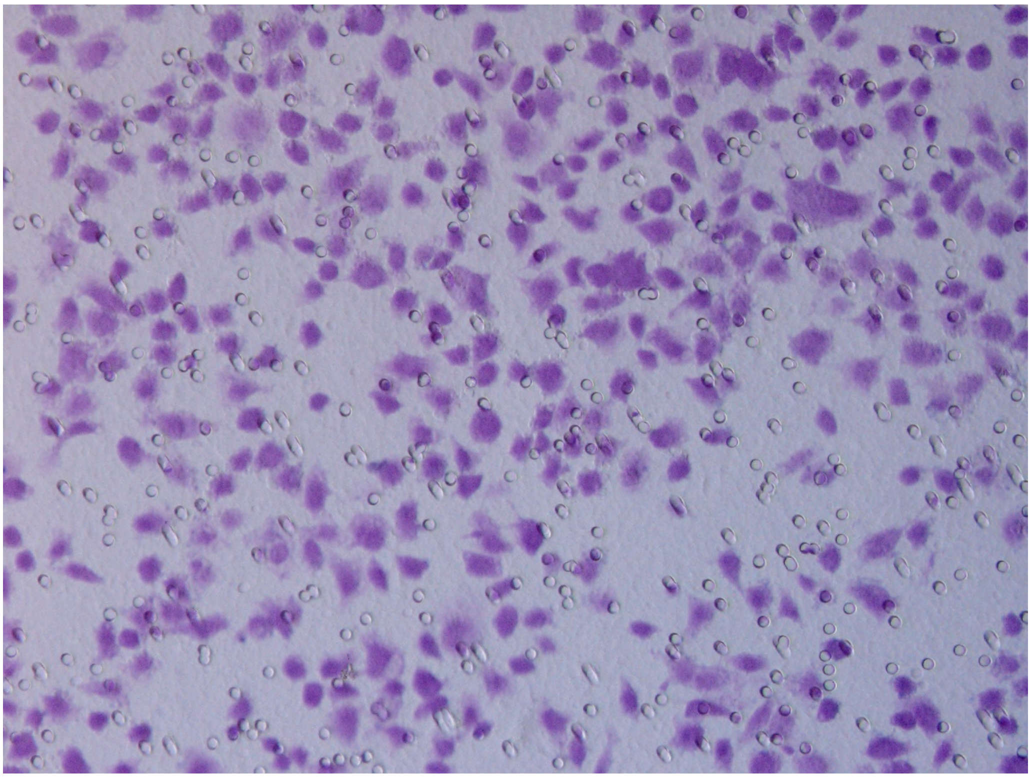

Fig.3B sh NC group with 0.013 μM 25-HC in SPC-A1 cells

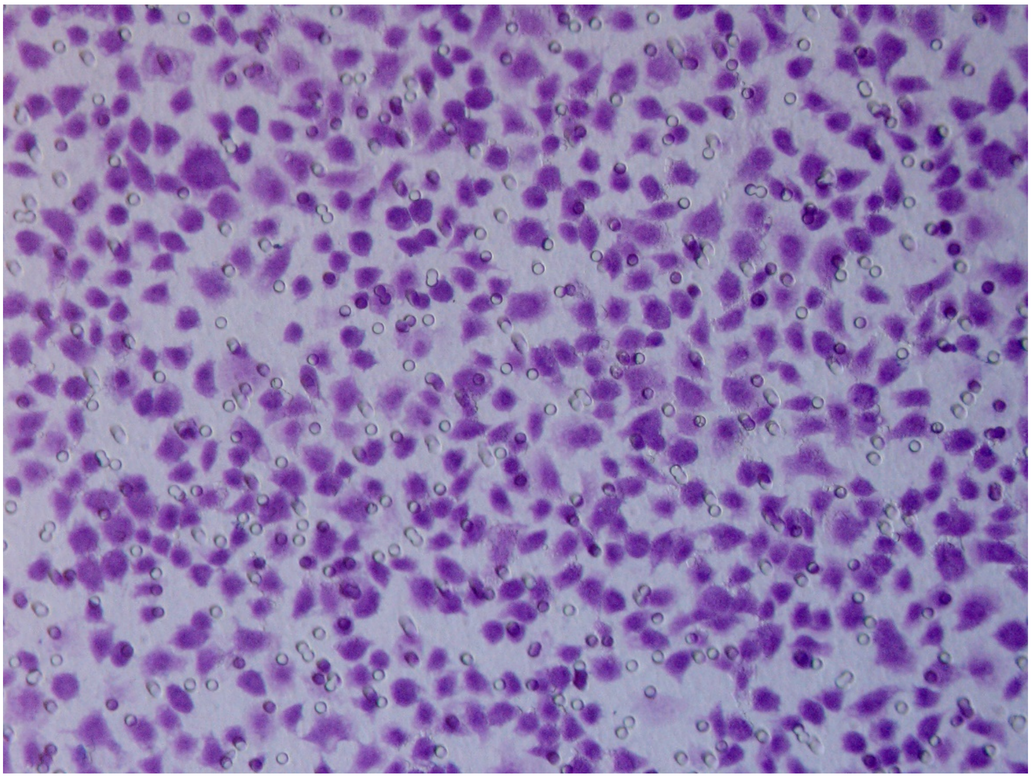

Fig.3B sh NC group with 0.085 μM 25-HC in SPC-A1 cells

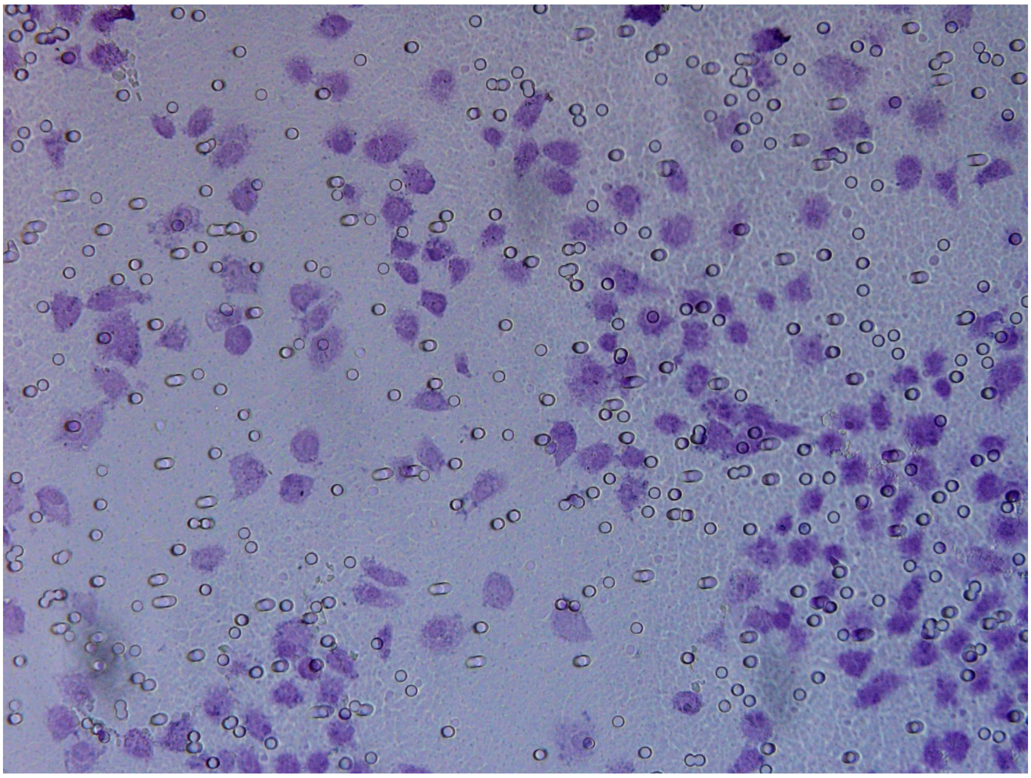
Fig.3B sh ERβ group with 0 μM 25-HC in SPC-A1 cells

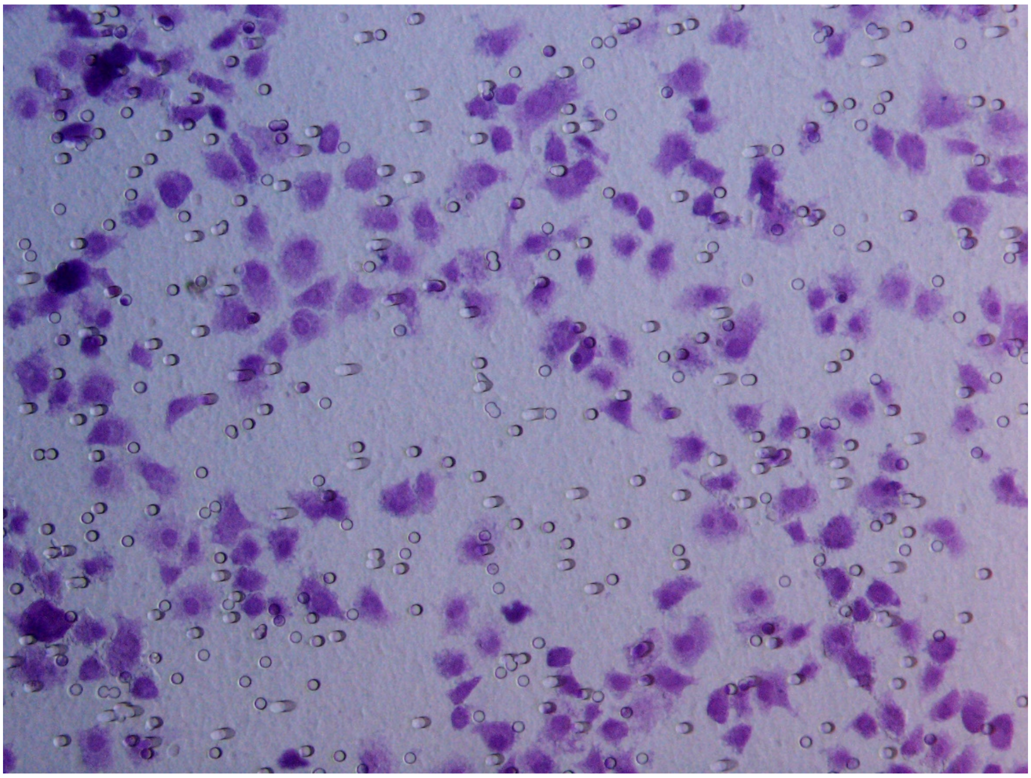
Fig.3B sh ERβ group with 0.013 μM 25-HC in SPC-A1 cells

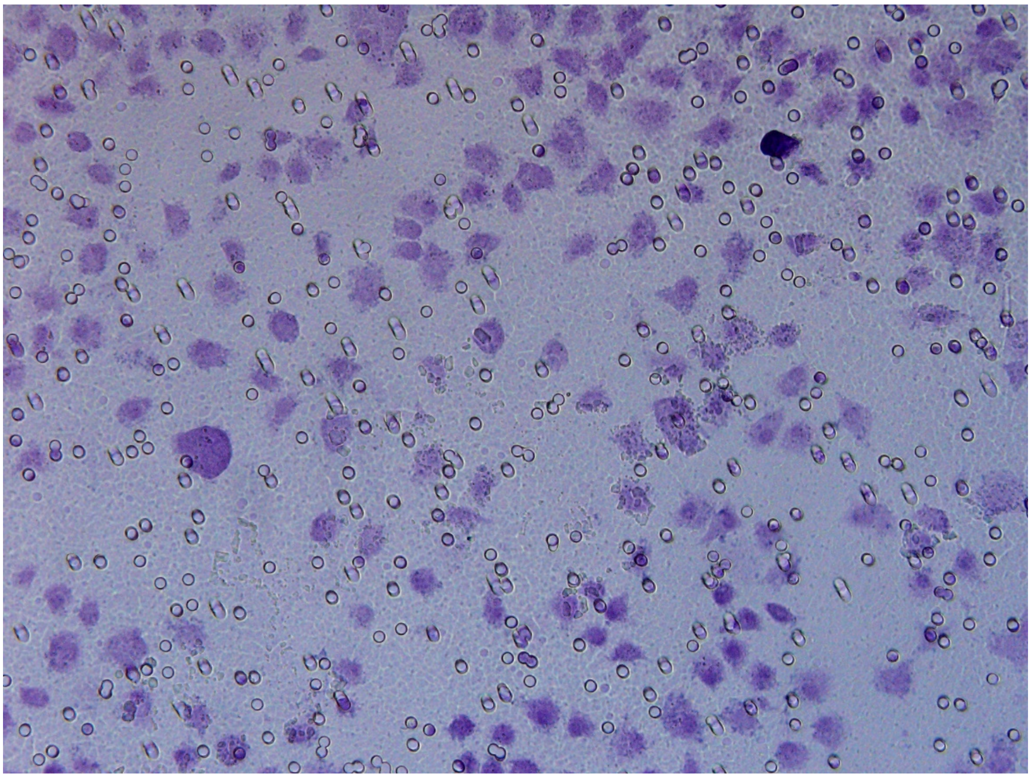

Fig.3B sh ERβ group with 0.085 μM 25-HC in SPC-A1 cells

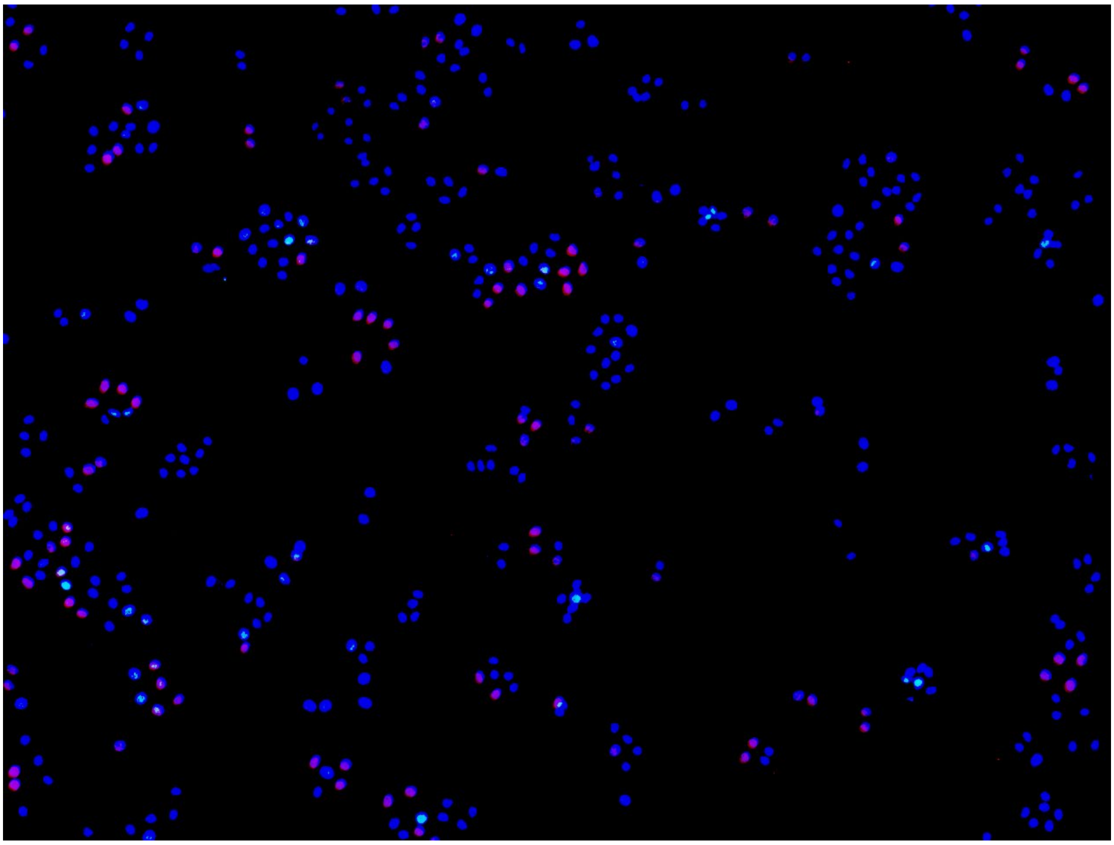

Fig.5B siNC group with 0 μM 25-HC in A549 cells

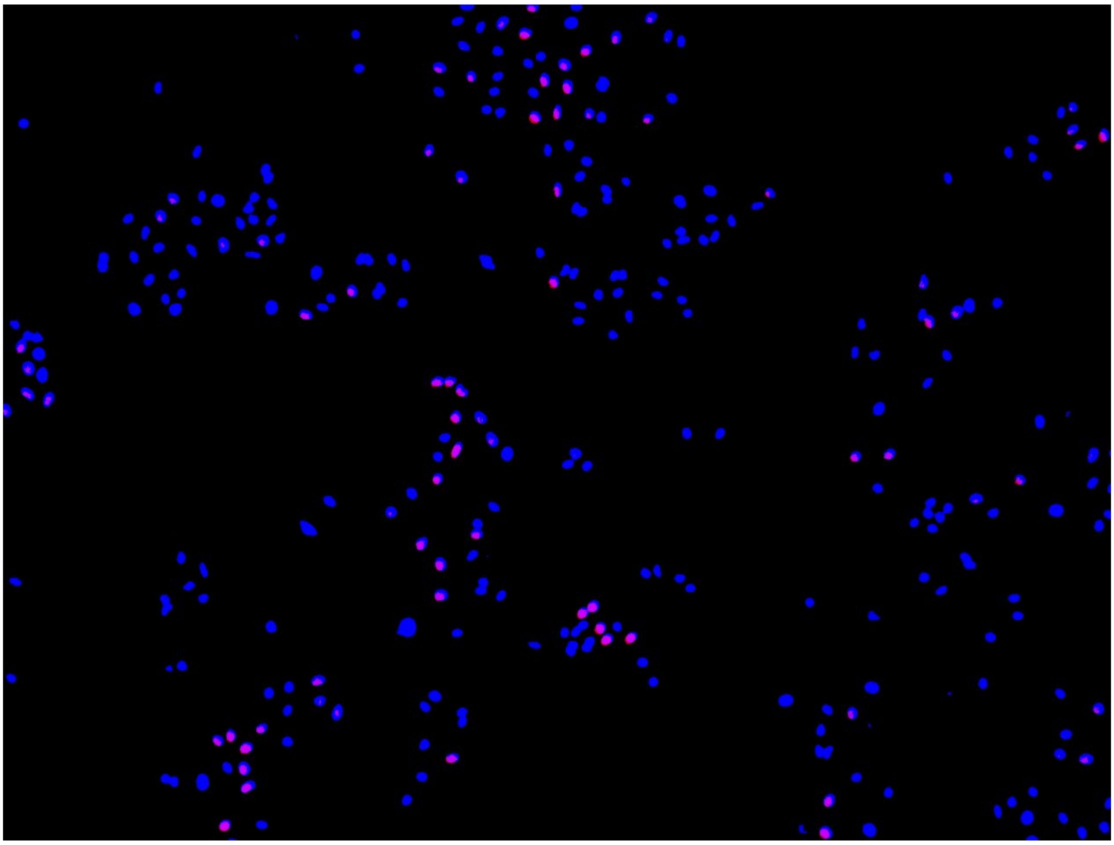

Fig.5B siNC group with 0.013 μM 25-HC in A549 cells

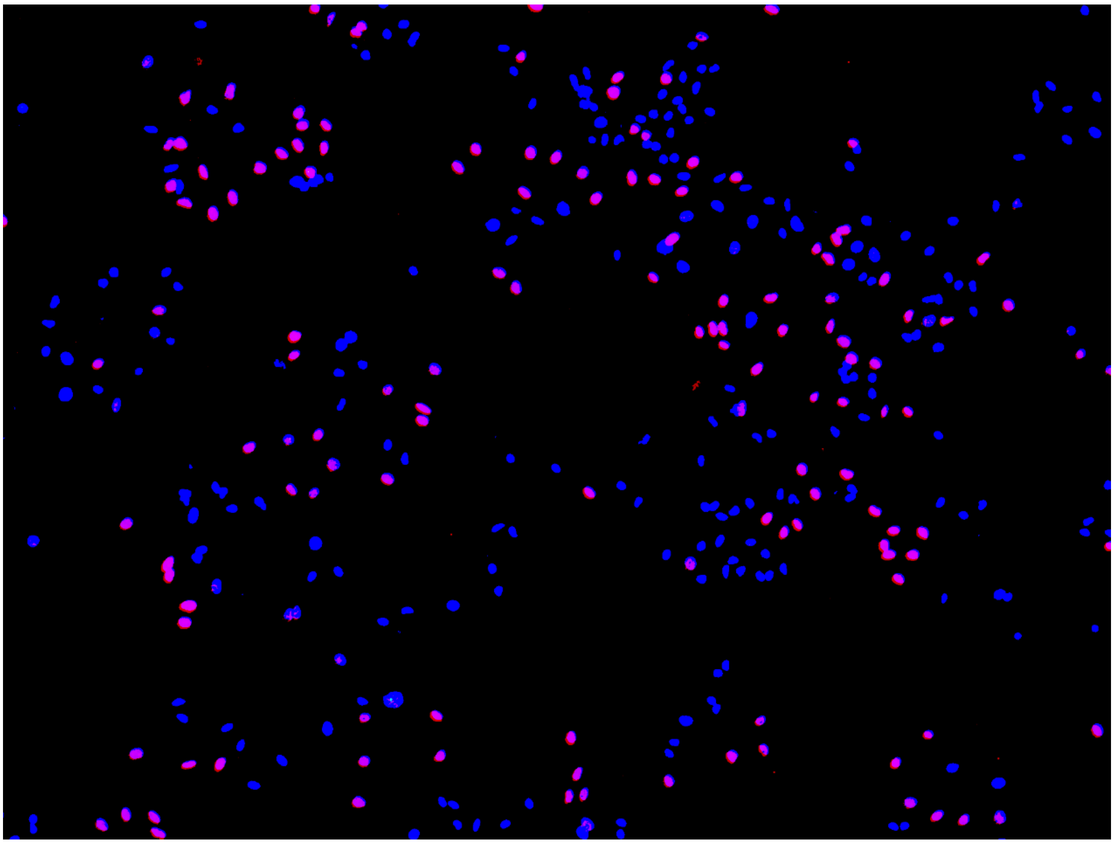

Fig.5B siNC group with 0.085 μM 25-HC in A549 cells

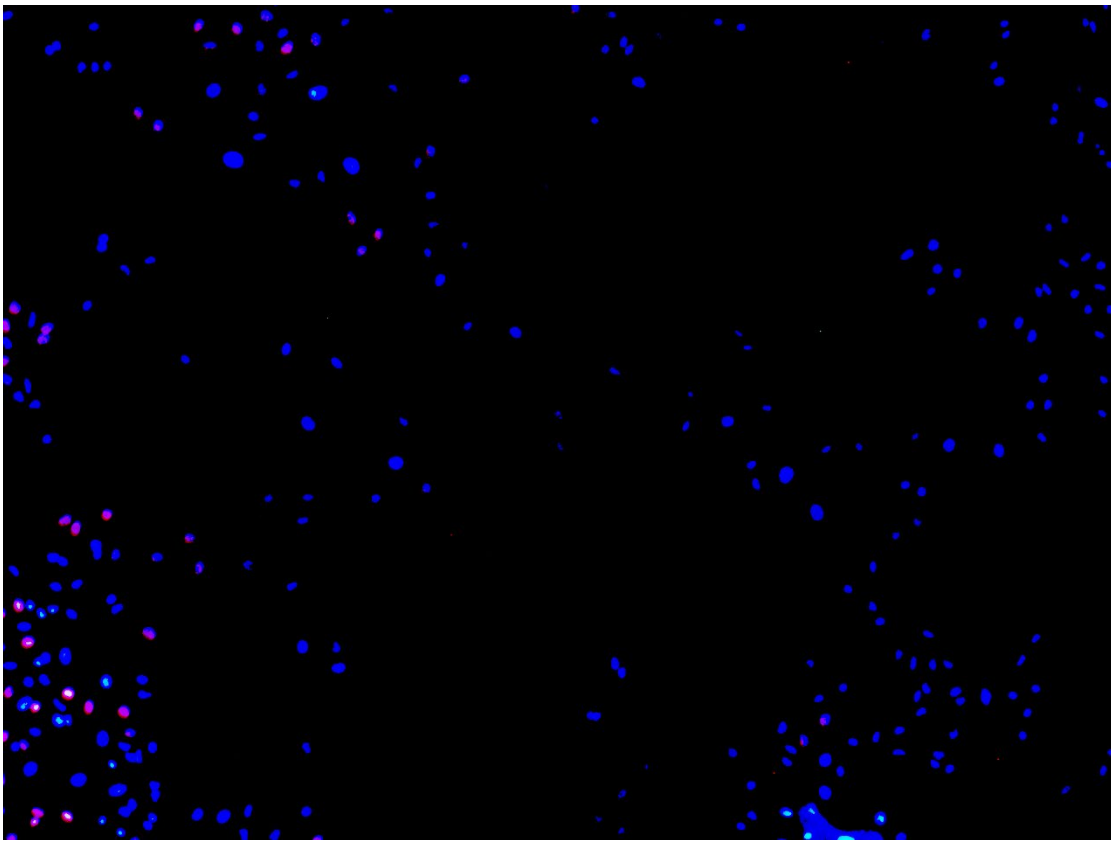

Fig.5B siTNFRSF17 group with 0 μM 25-HC in A549 cells

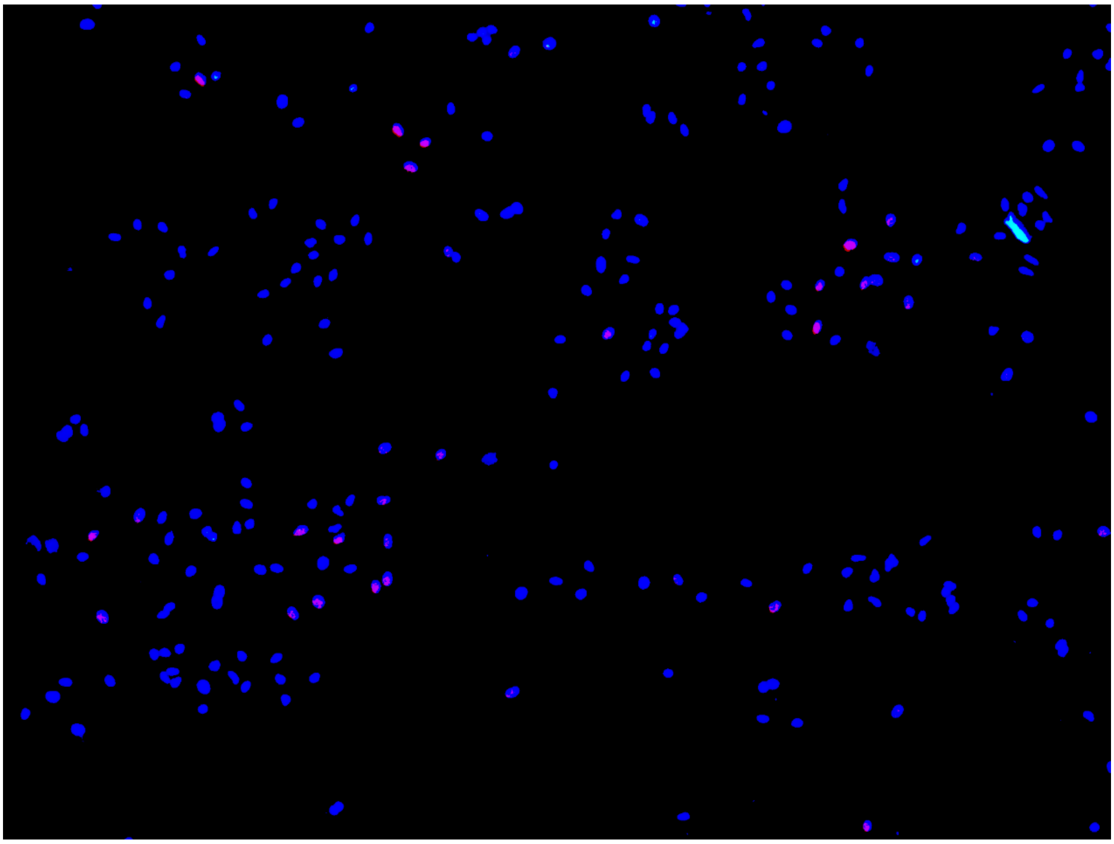

Fig.5B siTNFRSF17 group with 0.013 μM 25-HC in A549 cells

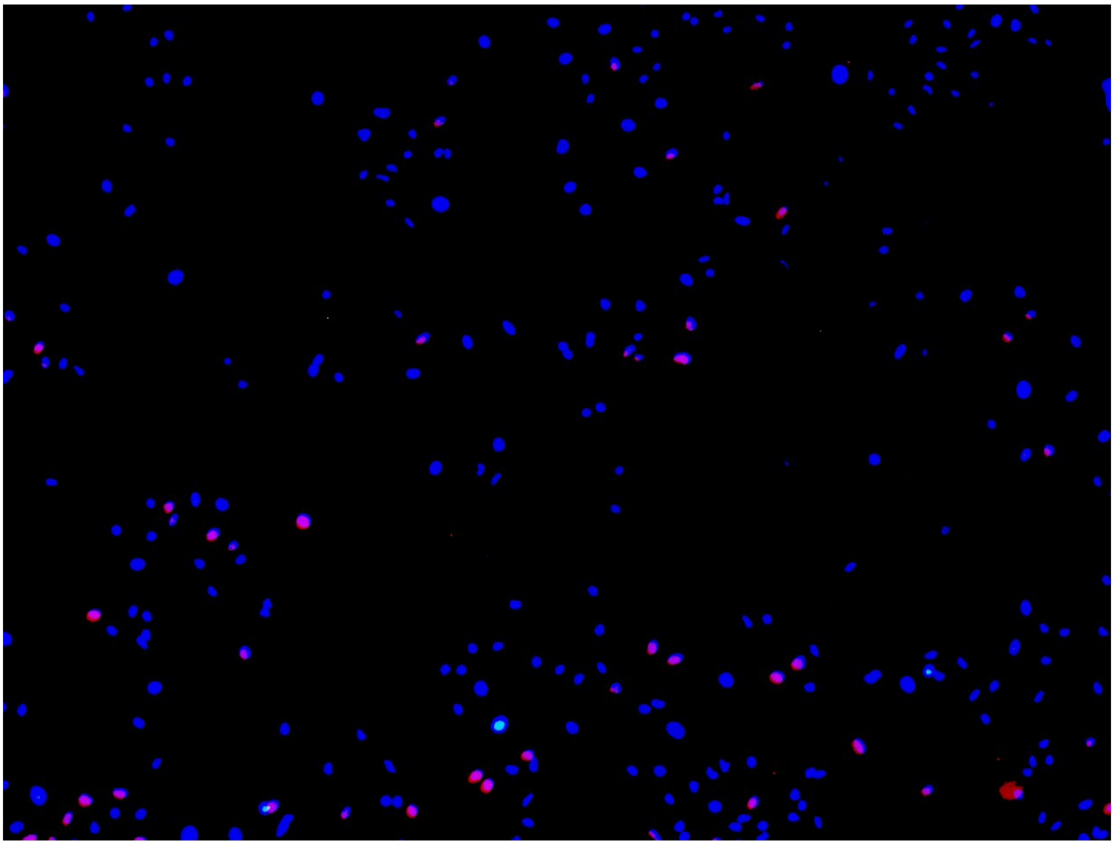

Fig.5B siTNFRSF17 group with 0.085 μM 25-HC in A549 cells

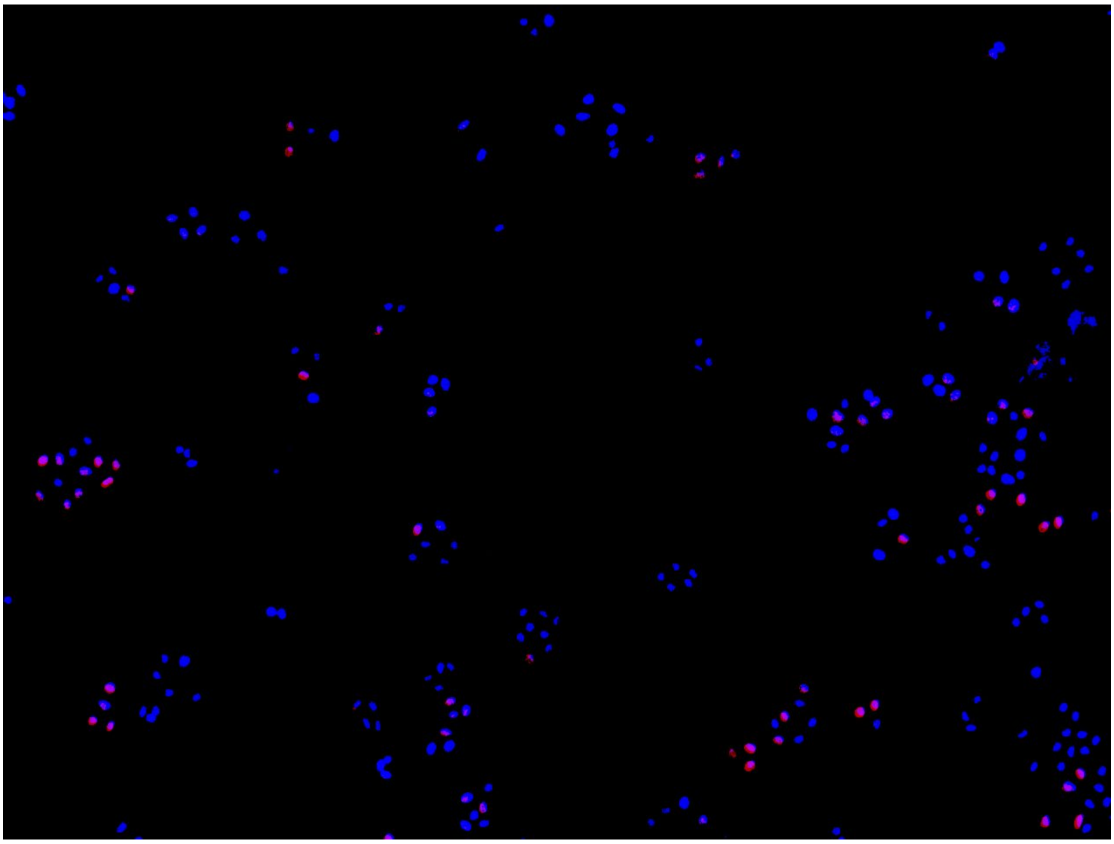

Fig.5B siNC group with 0 μM 25-HC in SPC-A1 cells

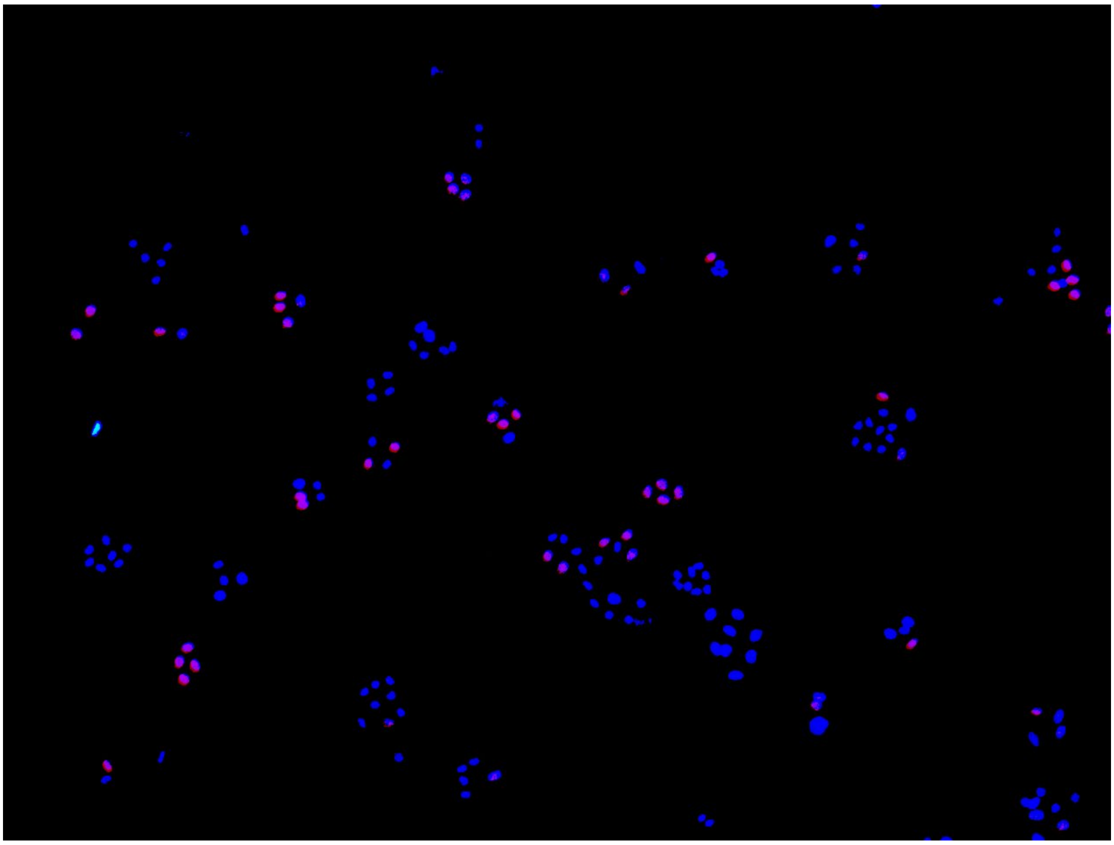

Fig.5B siNC group with 0.013 μM 25-HC in SPC-A1 cells

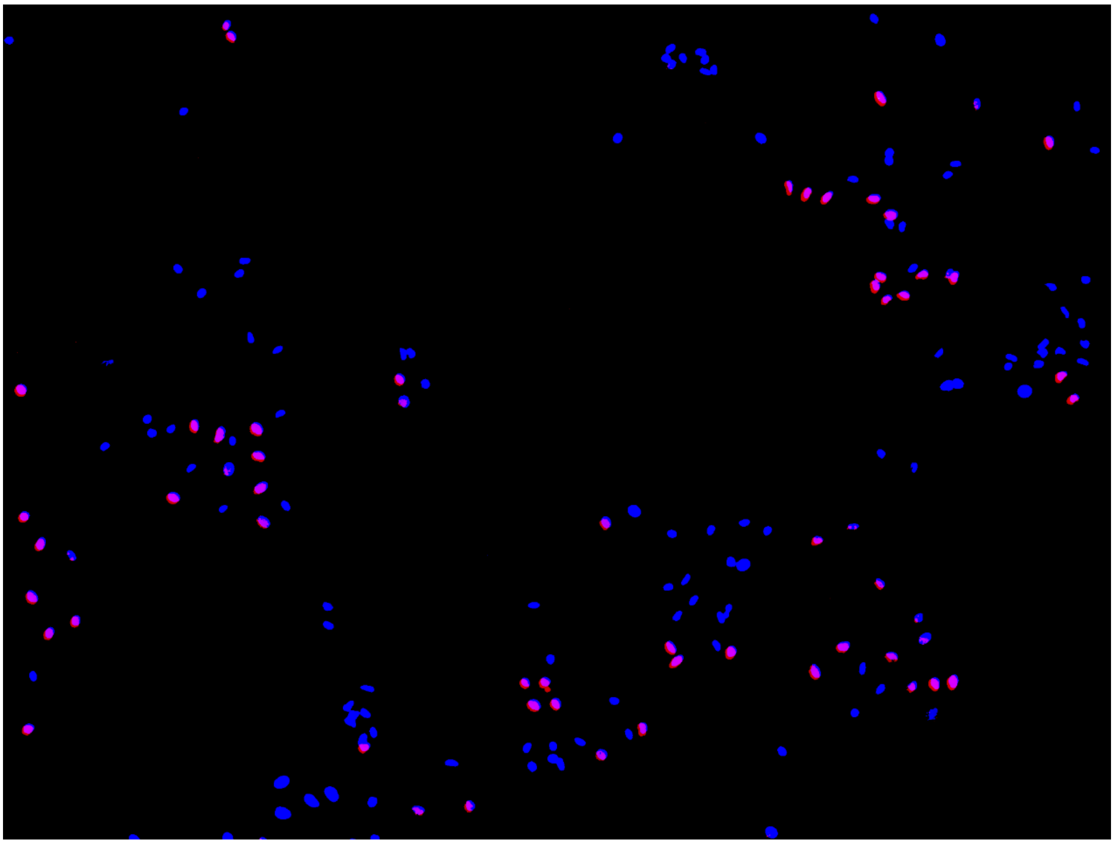

Fig.5B siNC group with 0.085 μM 25-HC in SPC-A1 cells

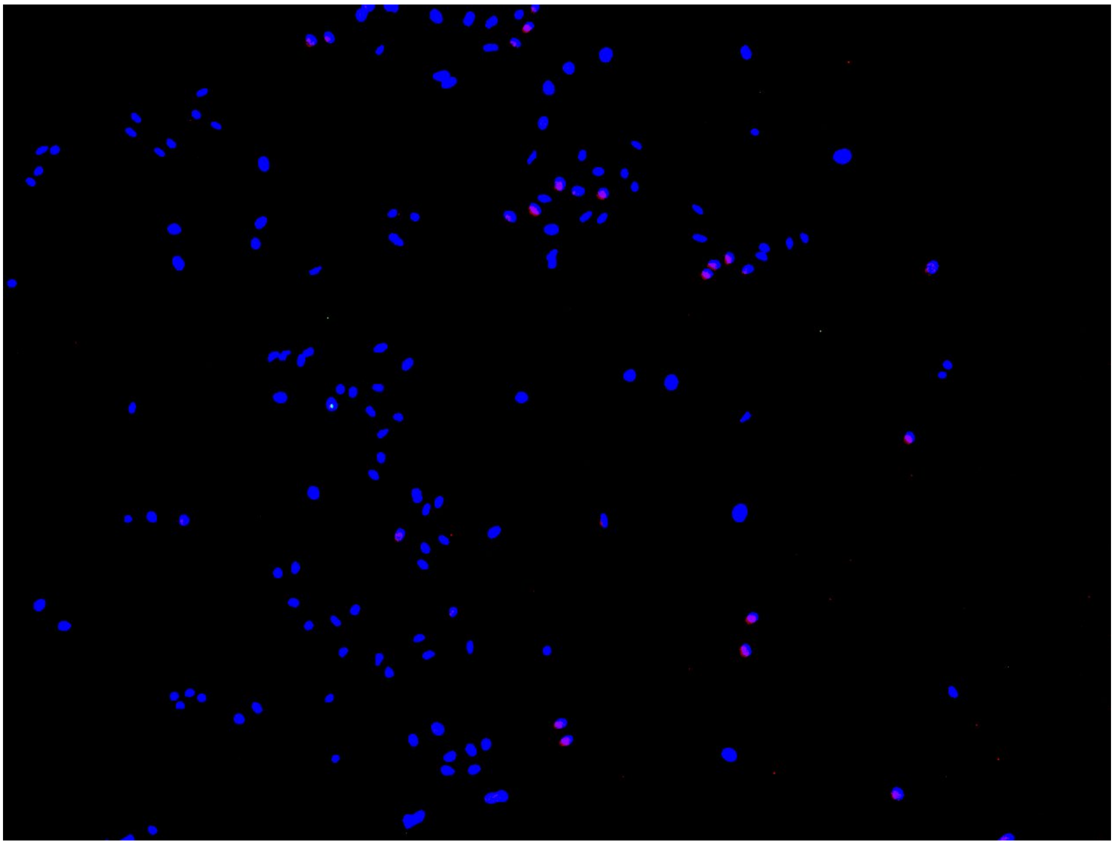

Fig.5B siTNFRSF17 group with 0 μM 25-HC in SPC-A1 cells

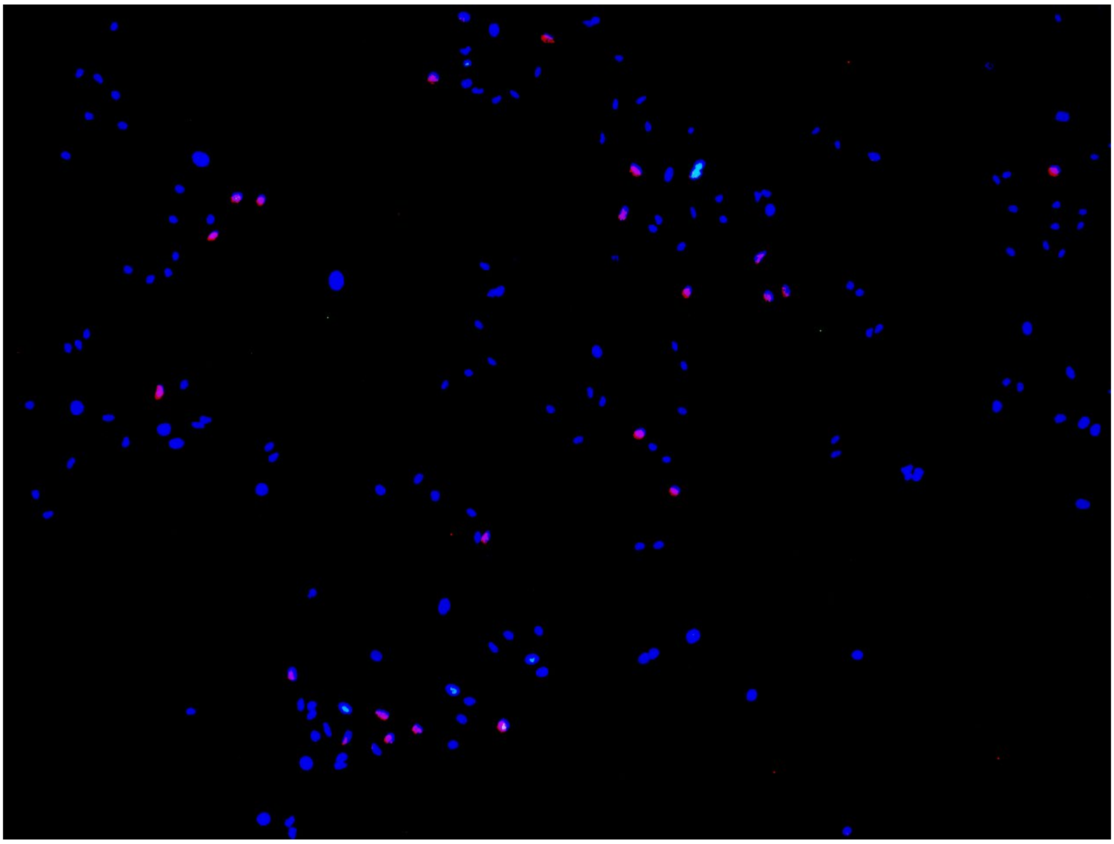

Fig.5B siTNFRSF17 group with 0.013 μM 25-HC in SPC-A1 cells

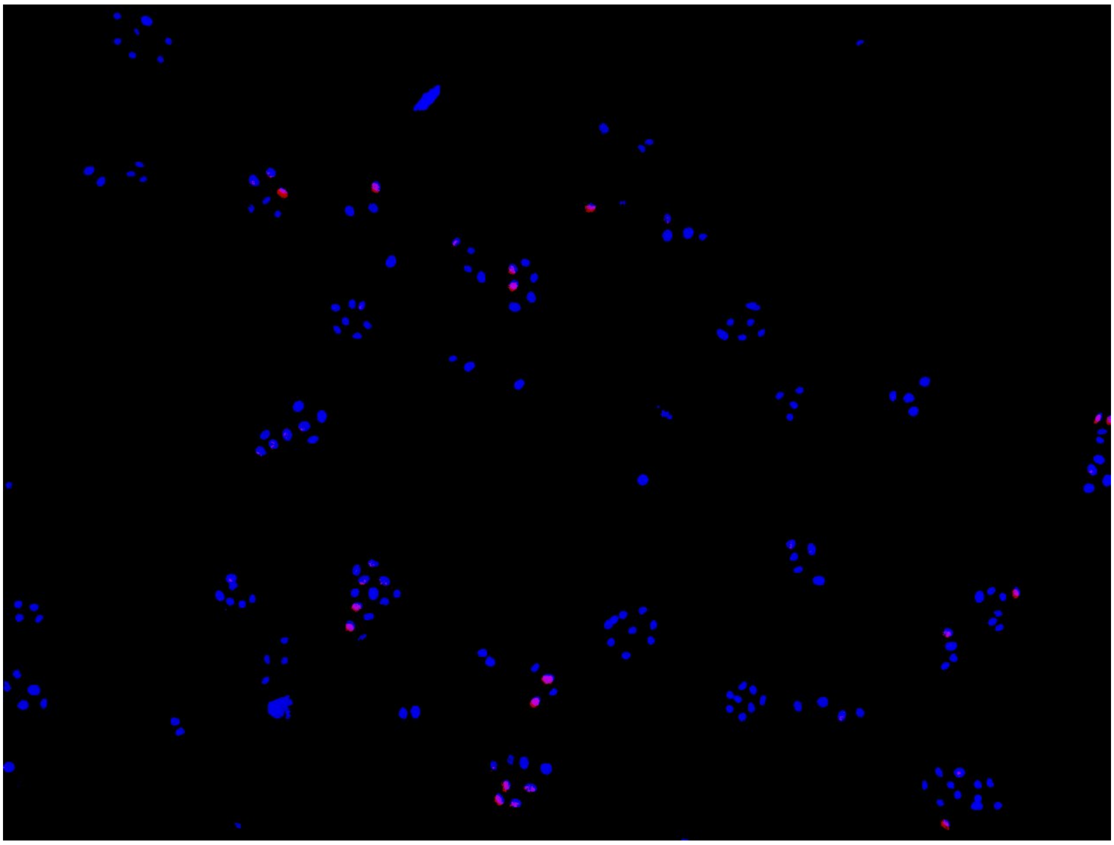

Fig.5B siTNFRSF17 group with 0.085 μM 25-HC in SPC-A1 cells
