# Supplementary material for: 25-hydroxycholesterol promotes proliferation and metastasis of lung adenocarcinoma cells by regulating ERβ/TNFRSF17 axis
Source: BMC Cancer. 2024 Apr 22;24:505. doi: 10.1186/s12885-024-12227-4 (PMC11034116; doi:10.1186/s12885-024-12227-4)
Supplement: Supplementary file 4 — Supplementary Material 4 [file 12885_2024_12227_MOESM4_ESM.pdf]

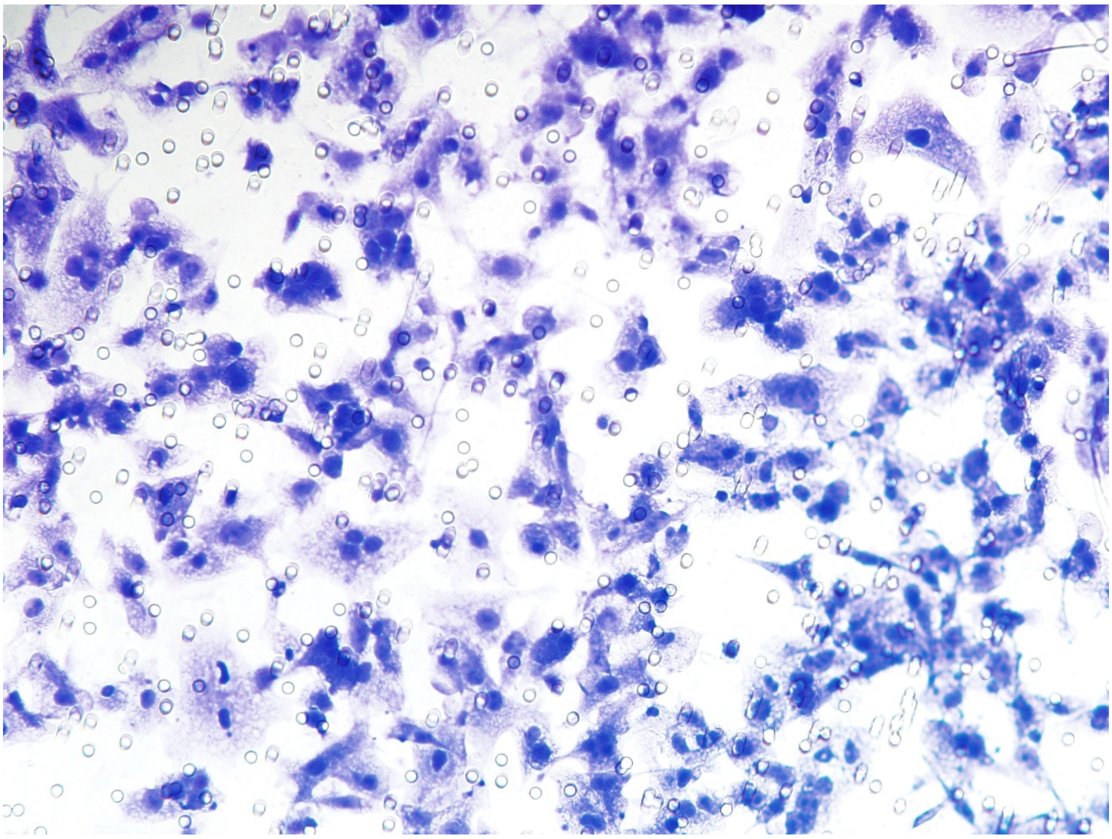

Fig.6A siNC group with 0 μM 25-HC in A549 cells

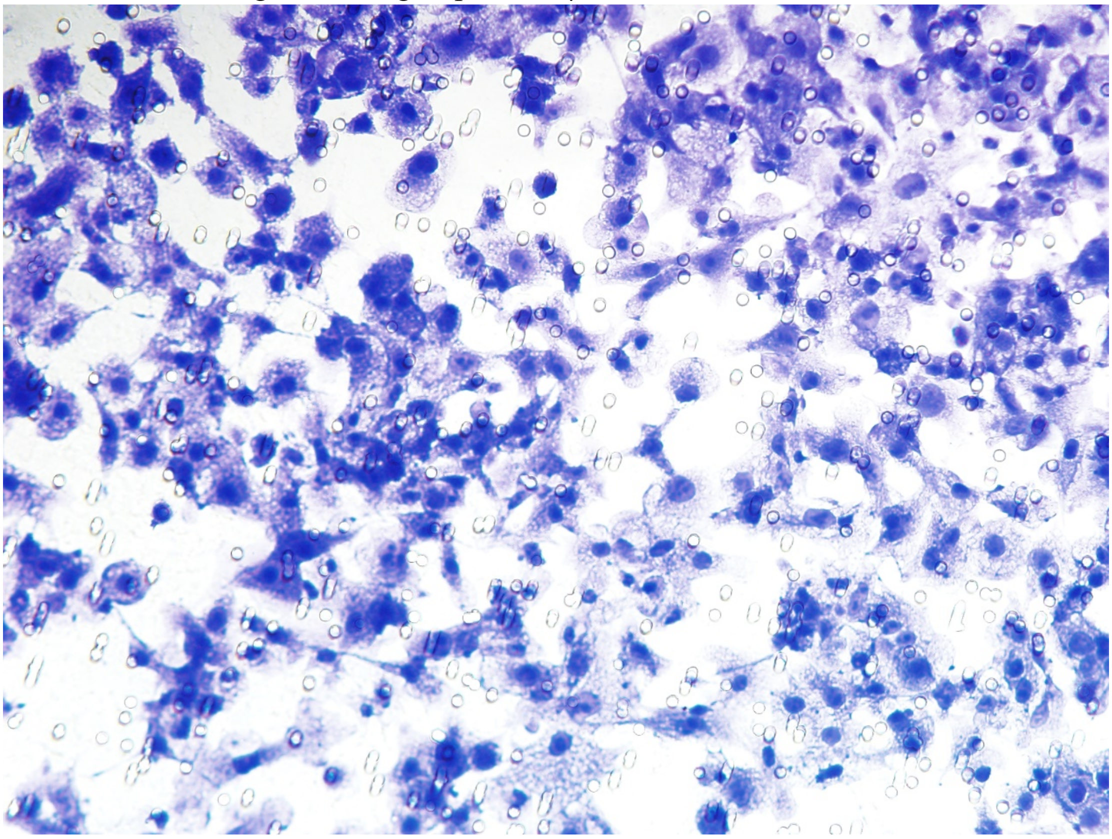

Fig.6A siNC group with 0.013 μM 25-HC in A549 cells

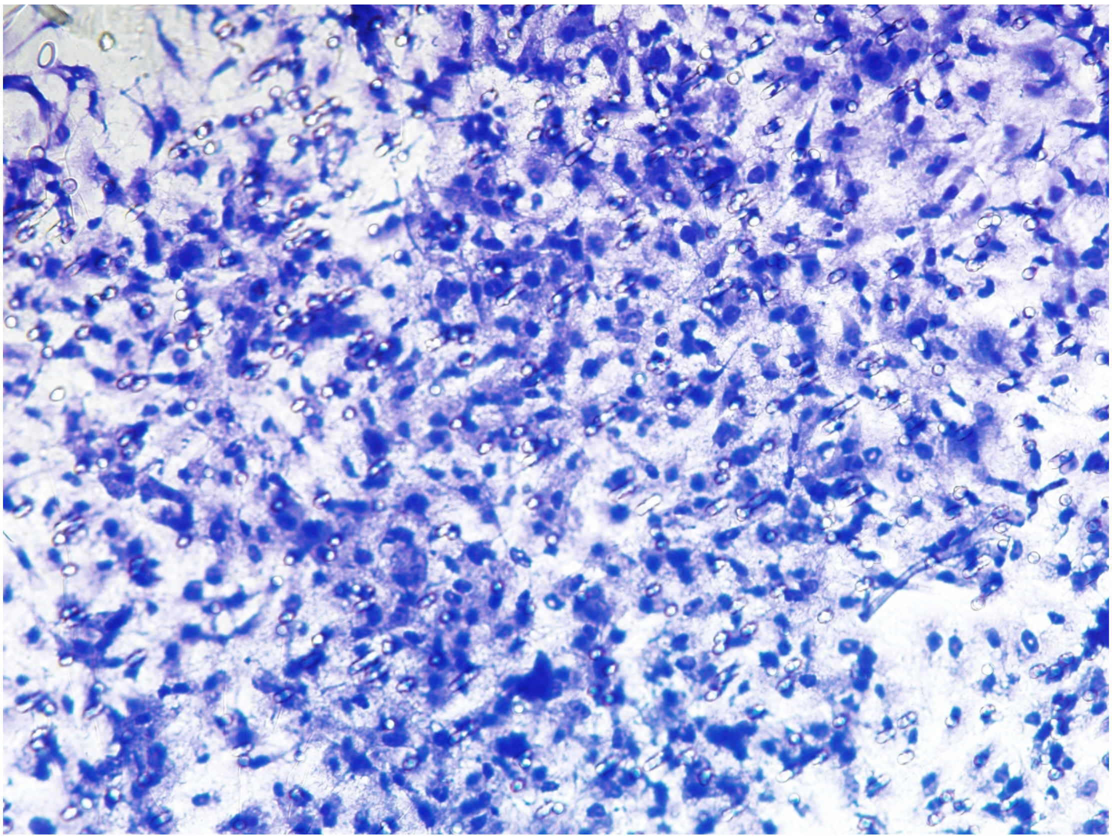
Fig.6A siNC group with 0.085 μM 25-HC in A549 cells

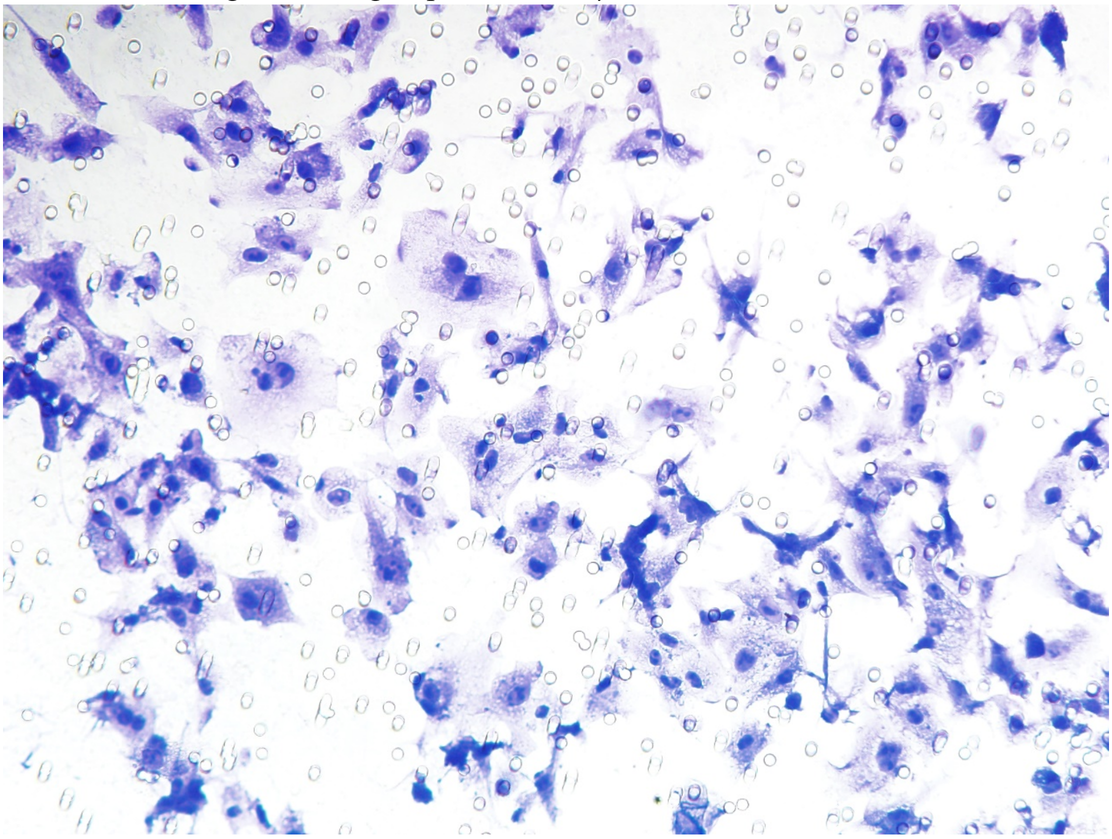
Fig.6A siTNFRSF17 group with 0 μM 25-HC in A549 cells

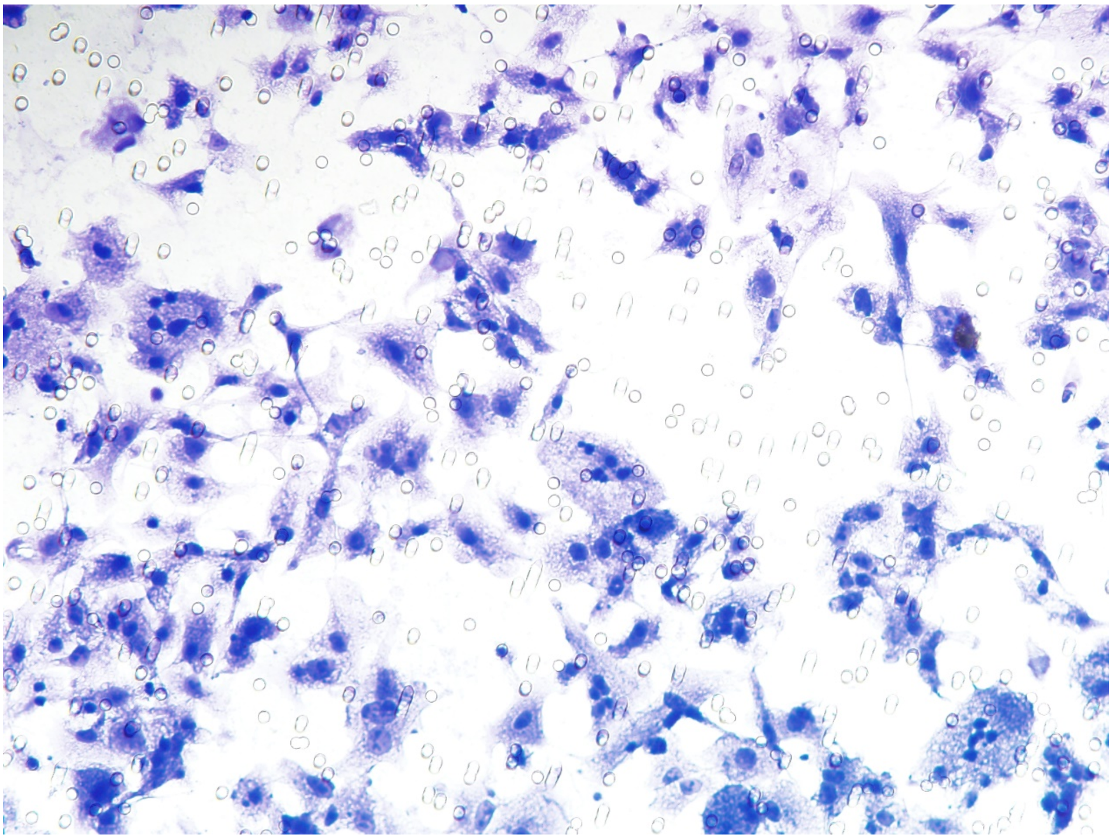

Fig.6A siTNFRSF17 group with 0.013 μM 25-HC in A549 cells

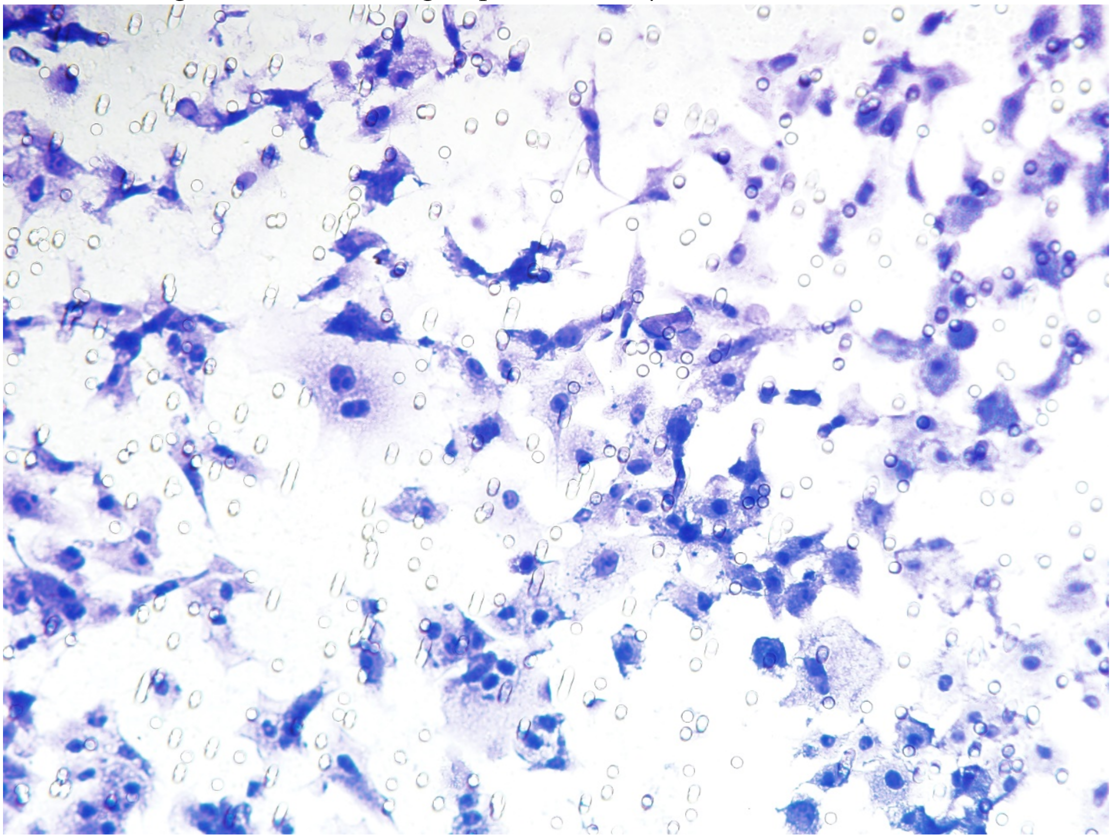

Fig.6A siTNFRSF17 group with 0.085 μM 25-HC in A549 cells

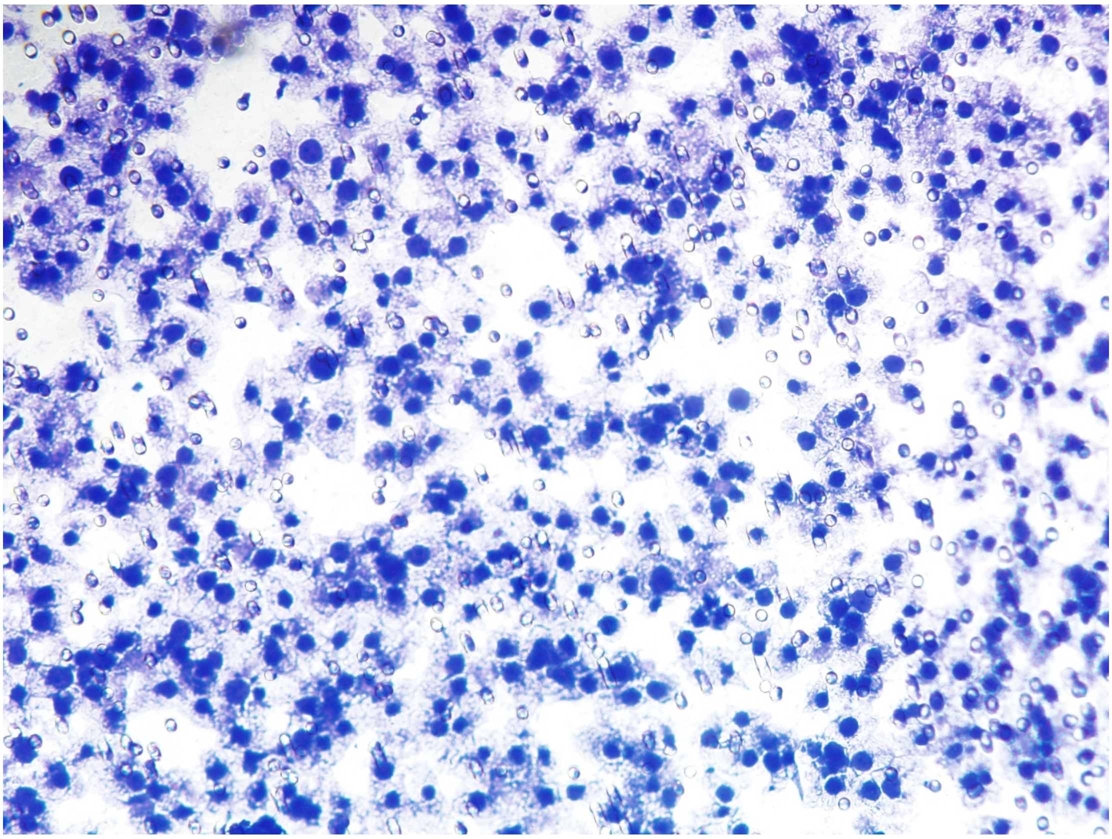

Fig.6A siNC group with 0 μM 25-HC in SPC-A1 cells

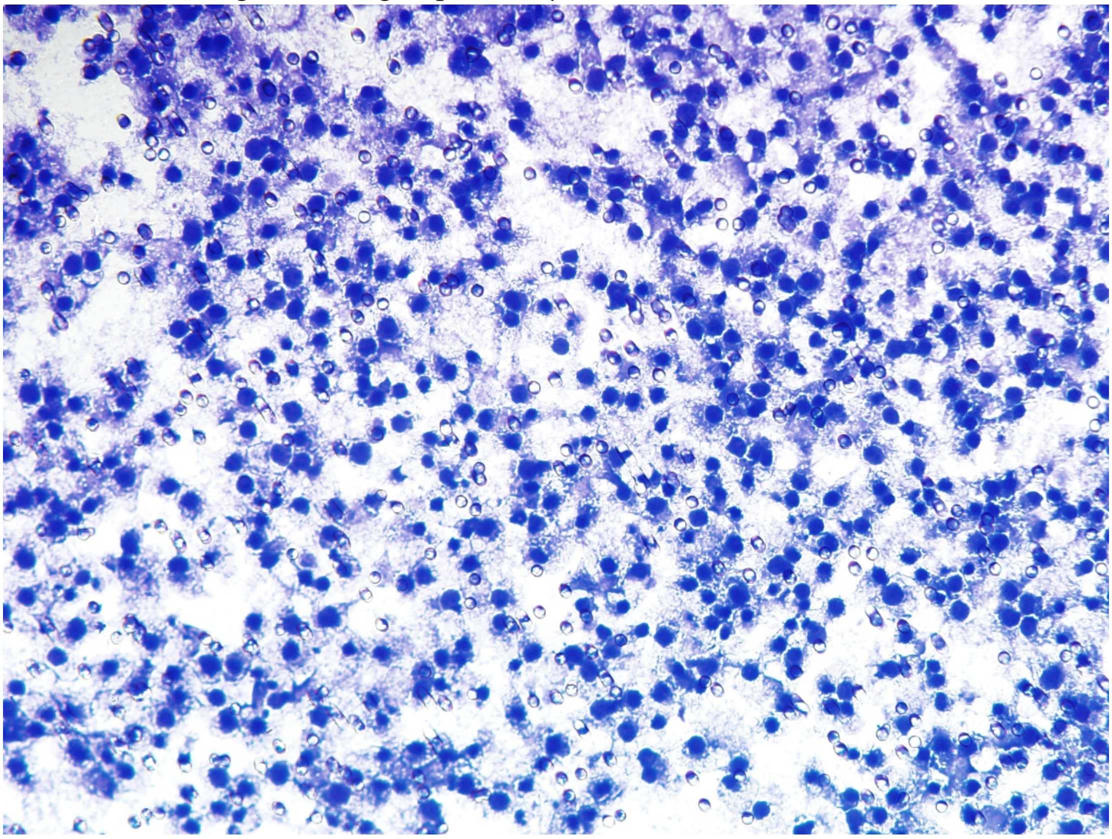

Fig.6A siNC group with 0.013 μM 25-HC in SPC-A1 cells

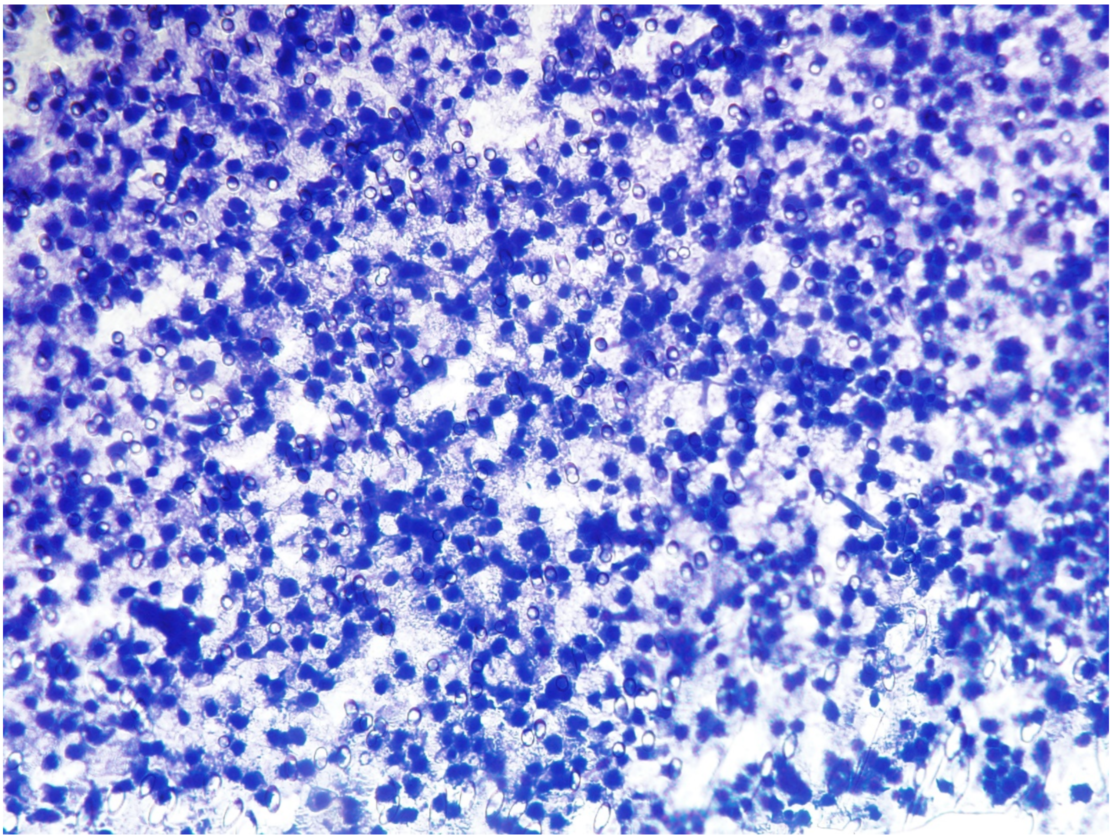

Fig.6A siNC group with 0.085 μM 25-HC in SPC-A1 cells

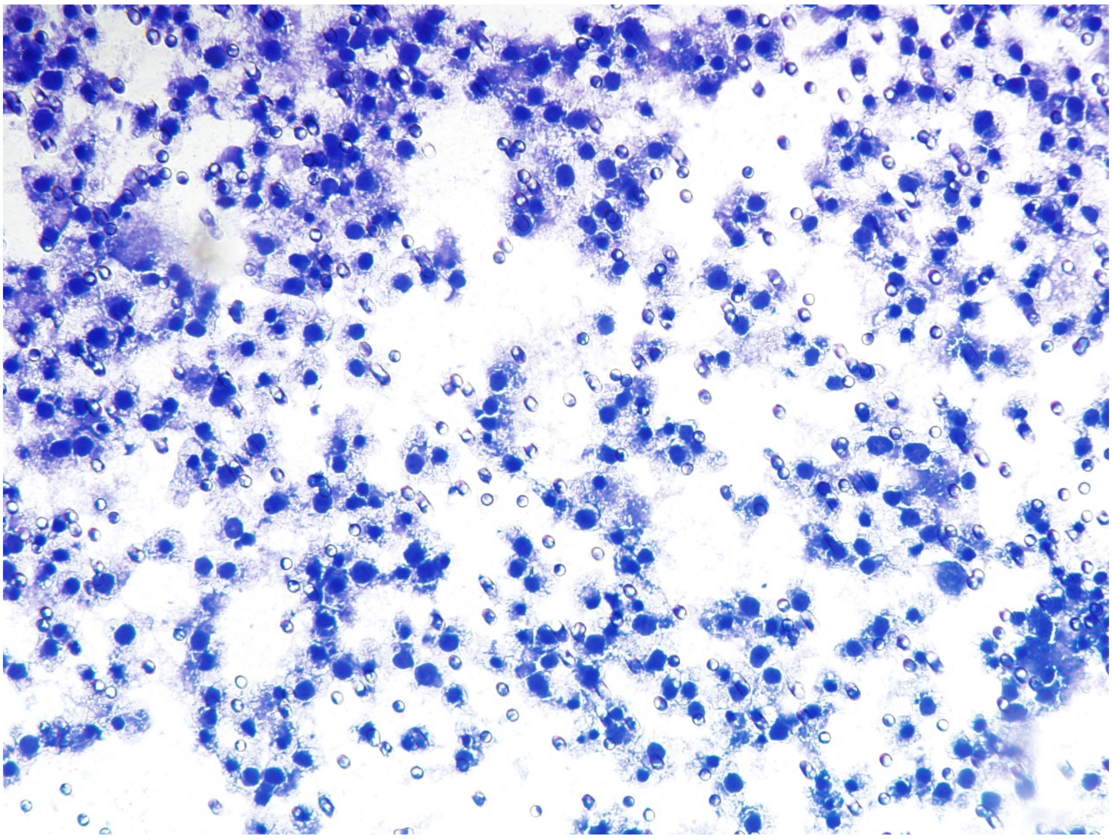

Fig.6A siTNFRSF17 group with 0 μM 25-HC in SPC-A1 cells

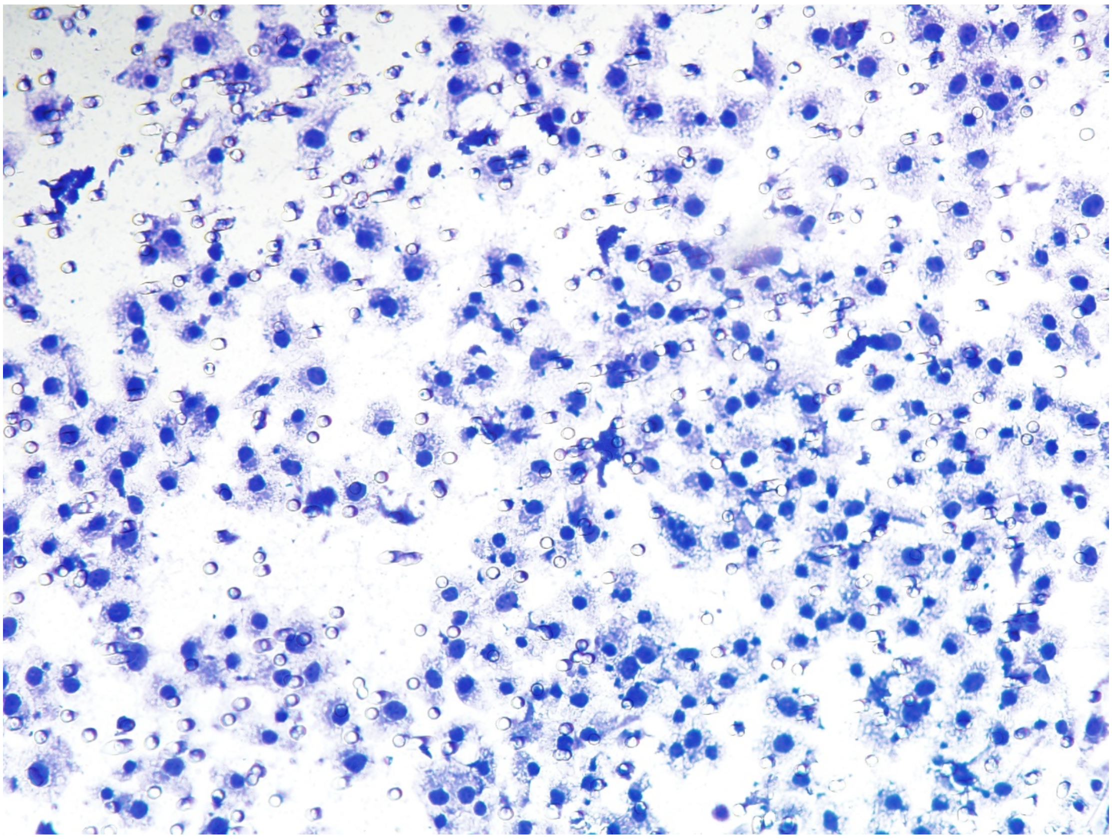
Fig.6A siTNFRSF17 group with 0.013 μM 25-HC in SPC-A1 cells

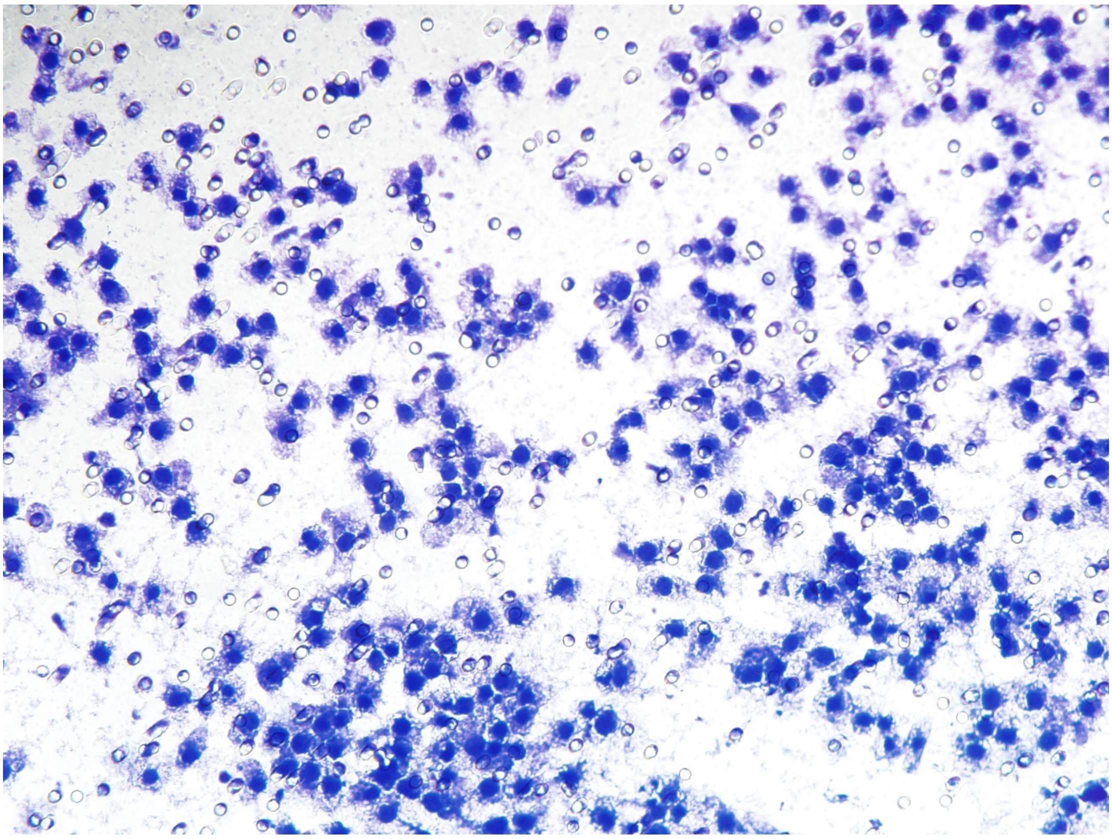
Fig.6A siTNFRSF17 group with 0.085 μM 25-HC in SPC-A1 cells

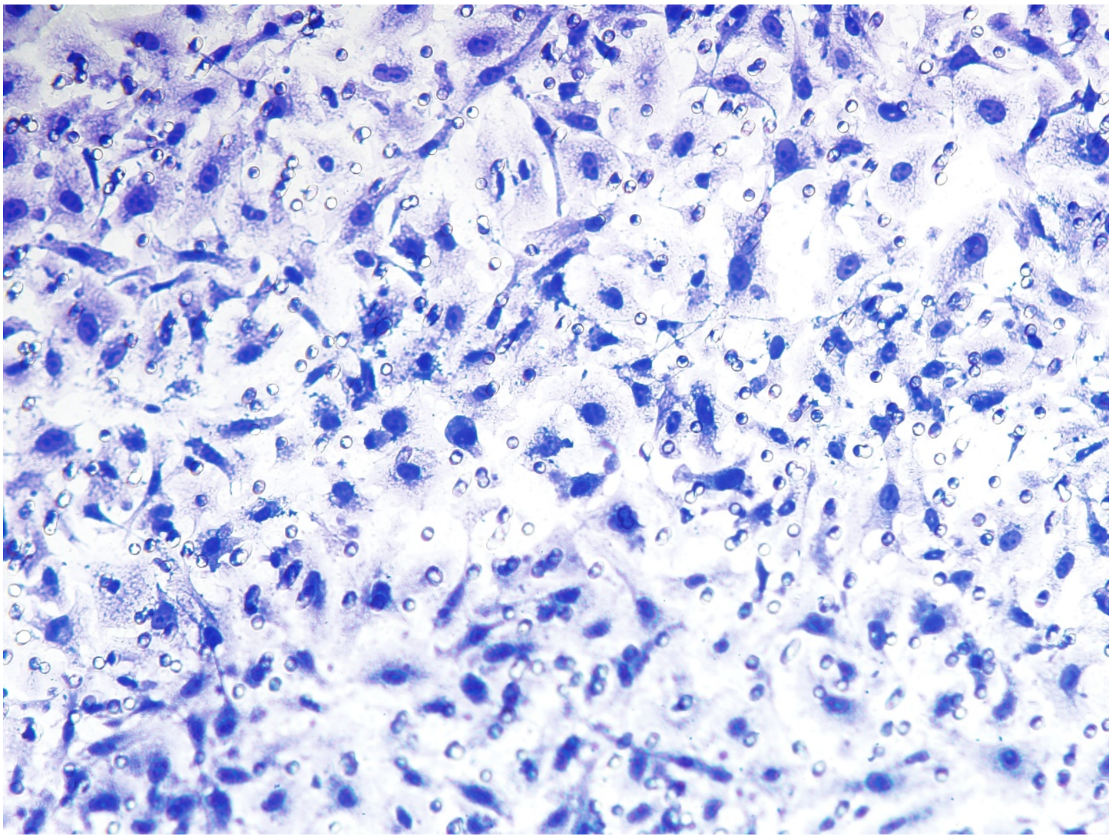
Fig.6B siNC group with 0 μM 25-HC in A549 cells
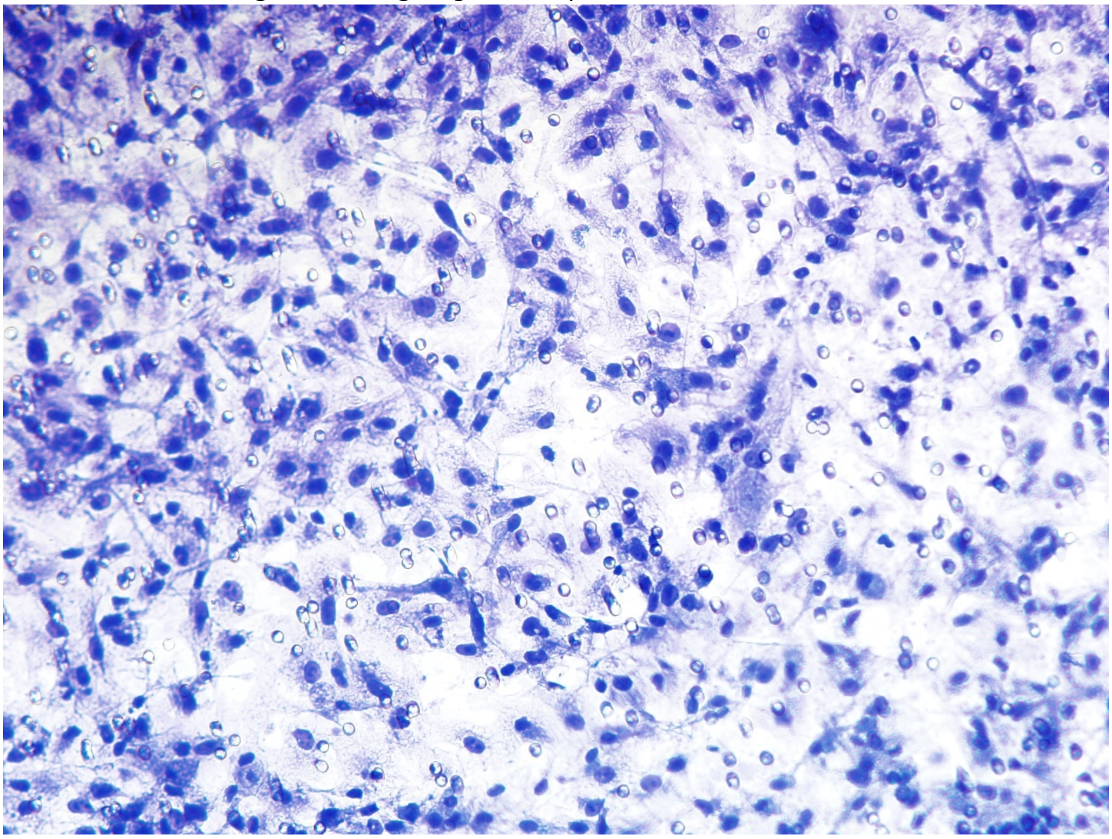
Fig.6B siNC group with 0.013 μM 25-HC in A549 cells

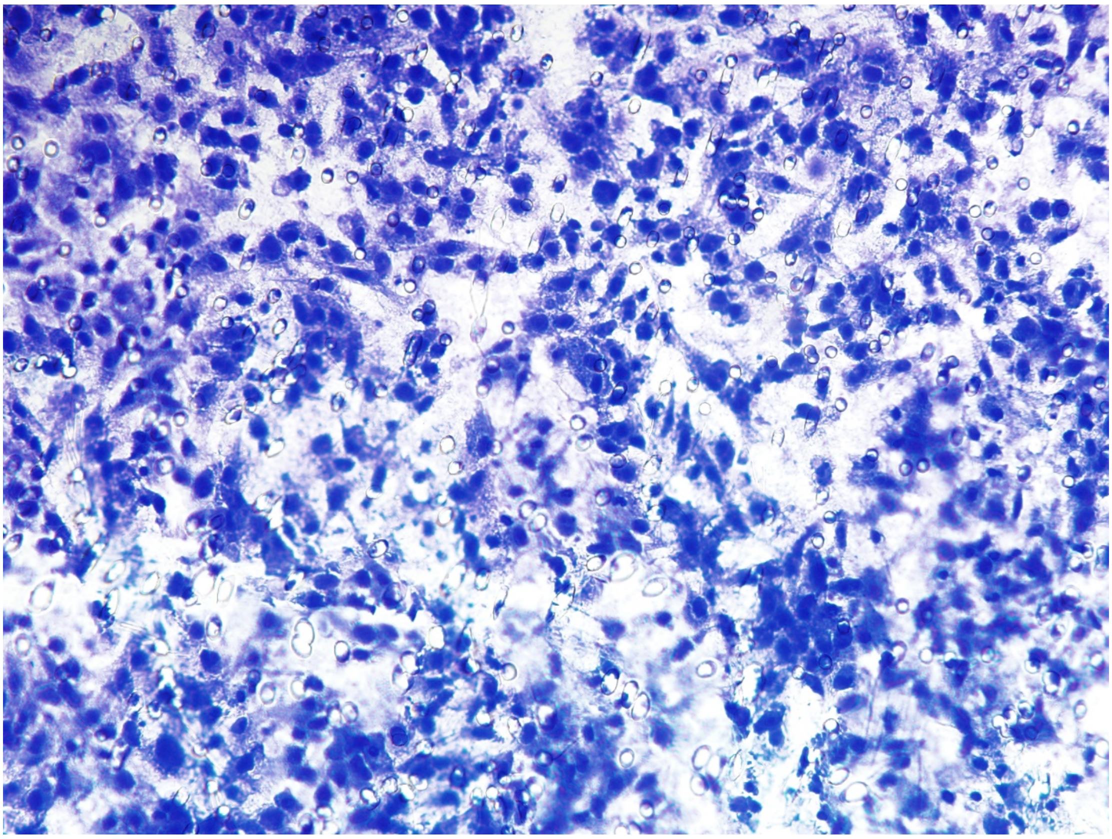
Fig.6B siNC group with 0.085 μM 25-HC in A549 cells
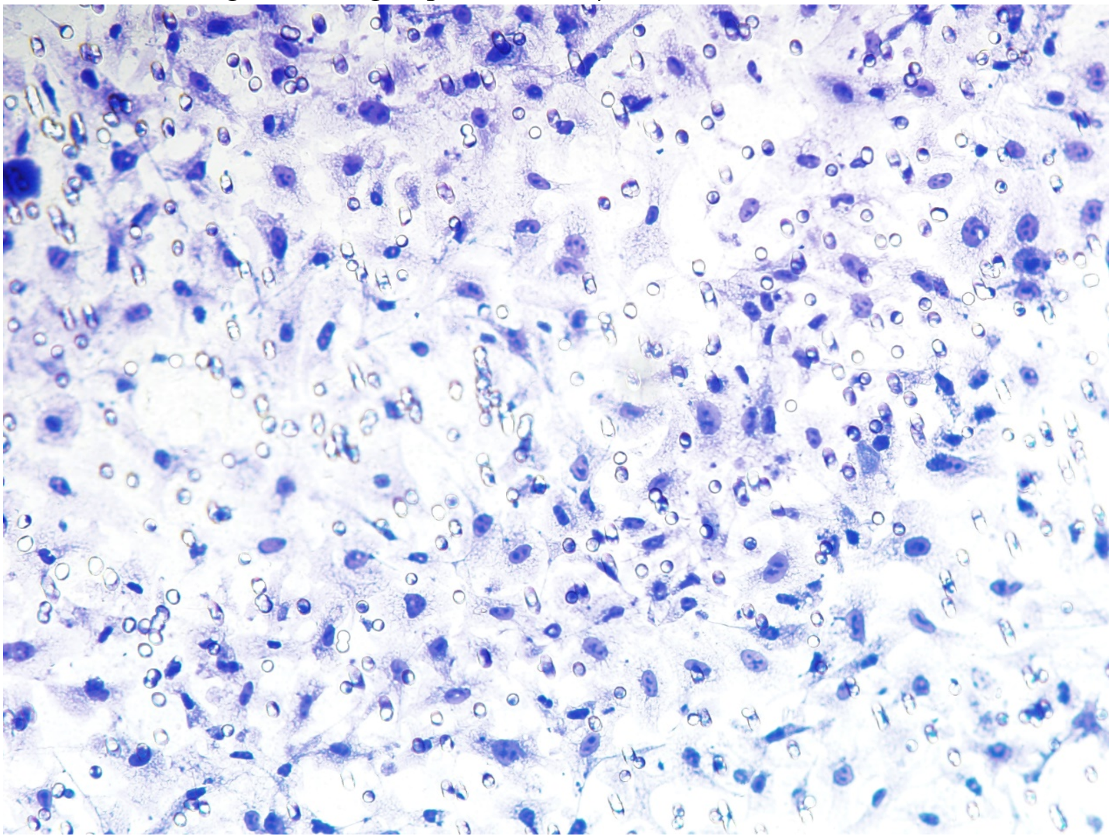
Fig.6B siTNFRSF17 group with 0 μM 25-HC in A549 cells

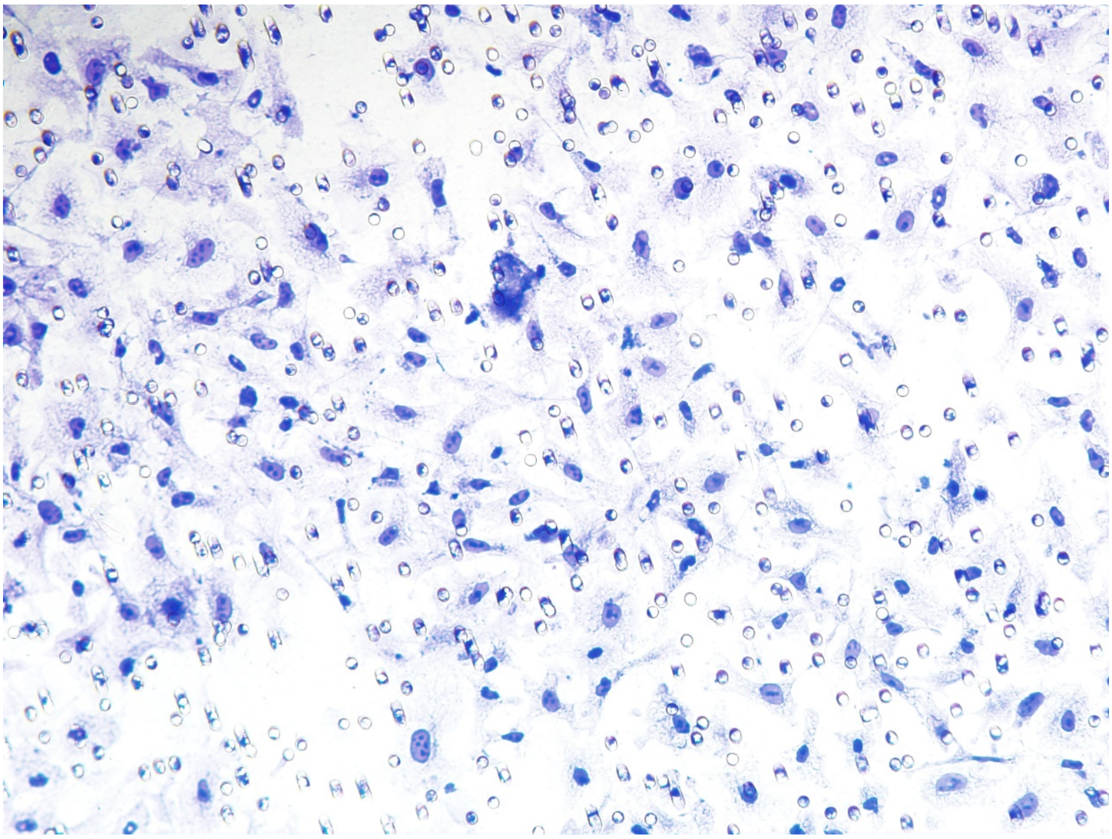

Fig.6B siTNFRSF17 group with 0.013 μM 25-HC in A549 cells

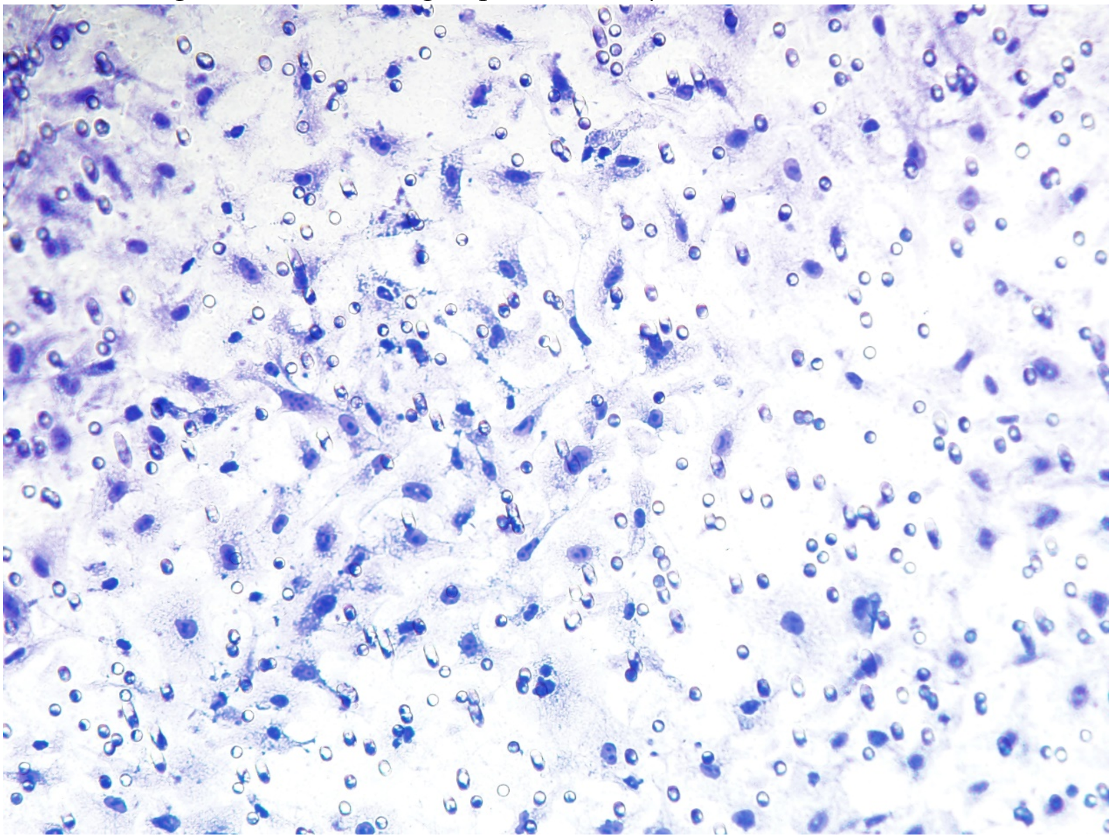

Fig.6B siTNFRSF17 group with 0.085 μM 25-HC in A549 cells

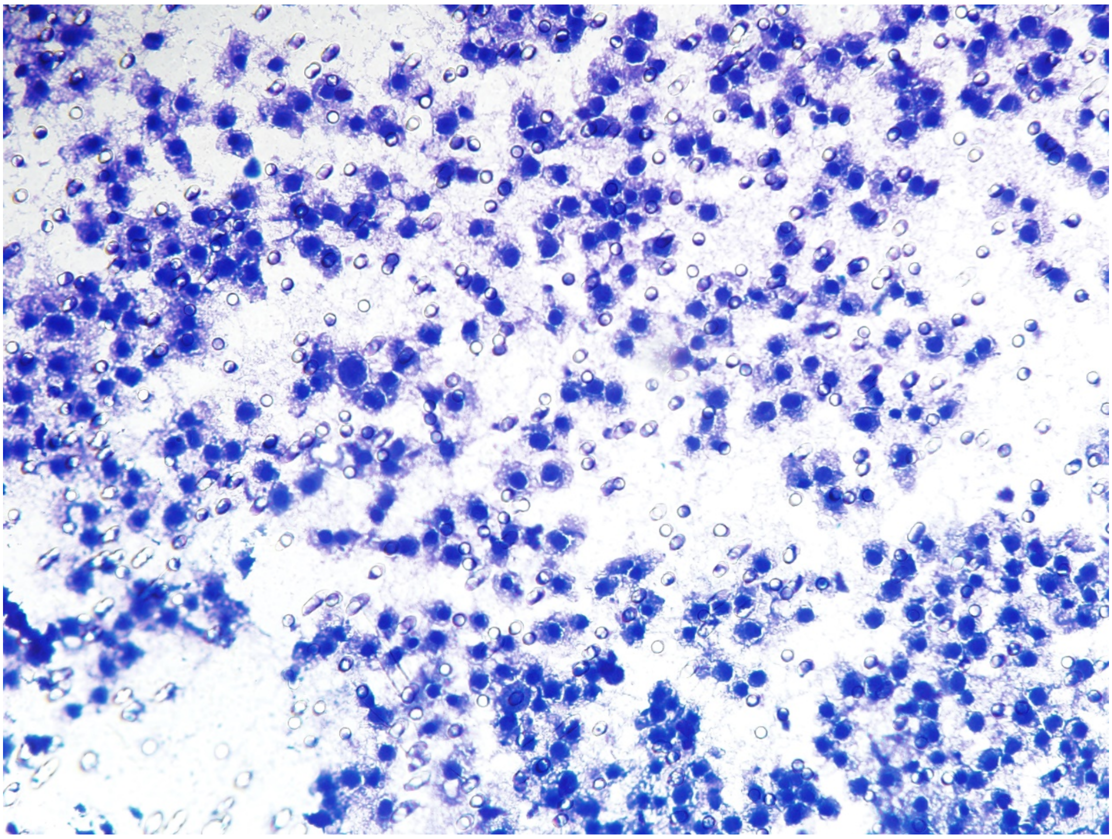

Fig.6B siNC group with 0 μM 25-HC in SPC-A1 cells

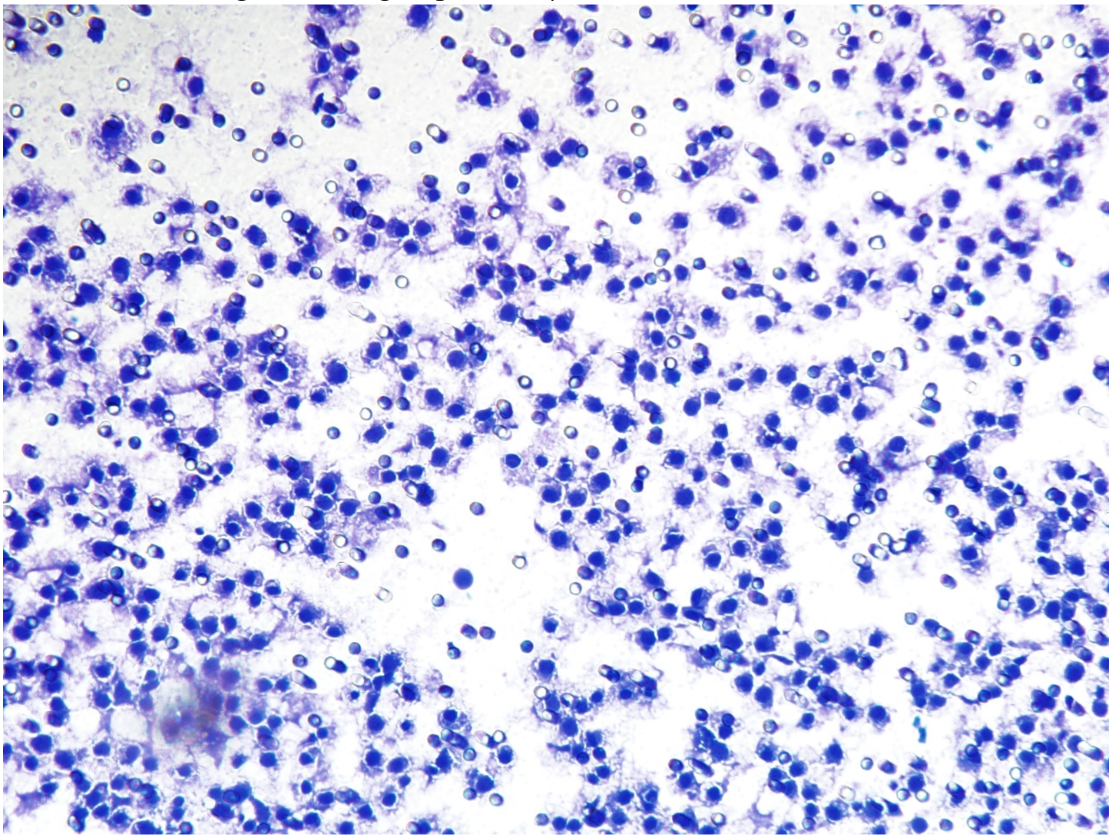

Fig.6B siNC group with 0.013 μM 25-HC in SPC-A1 cells

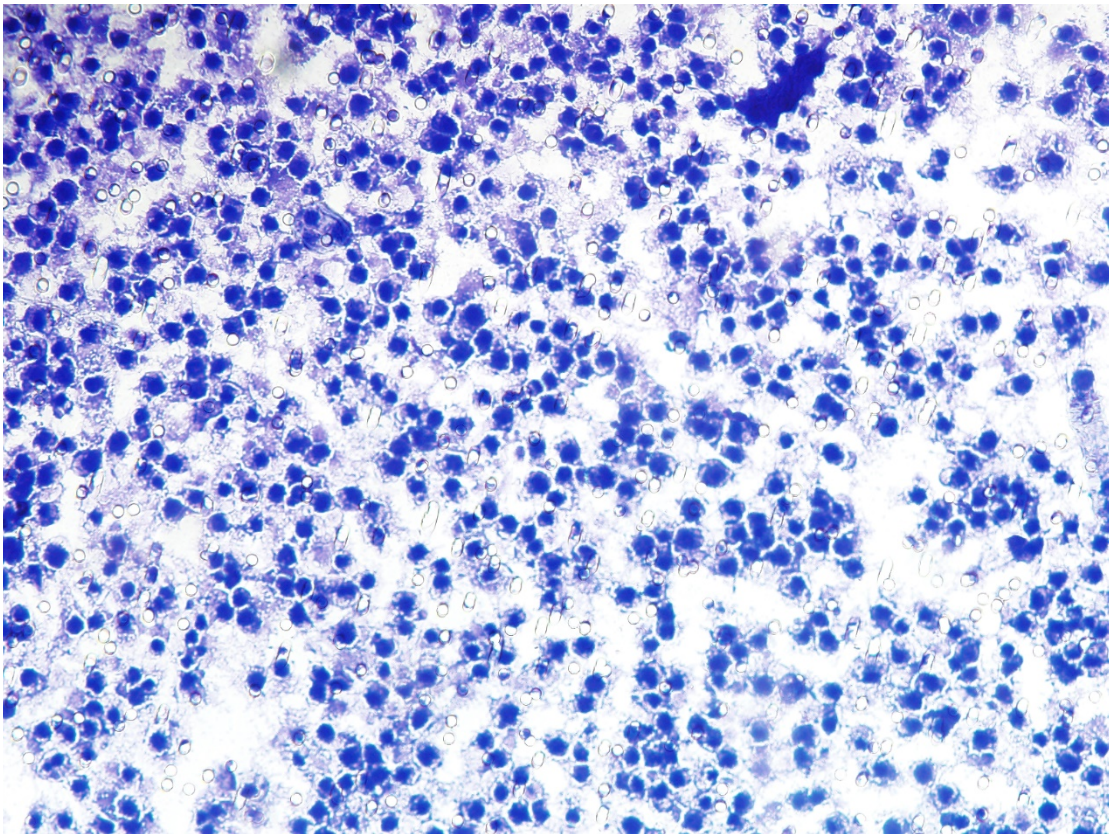

Fig.6B siNC group with 0.085 μM 25-HC in SPC-A1cells

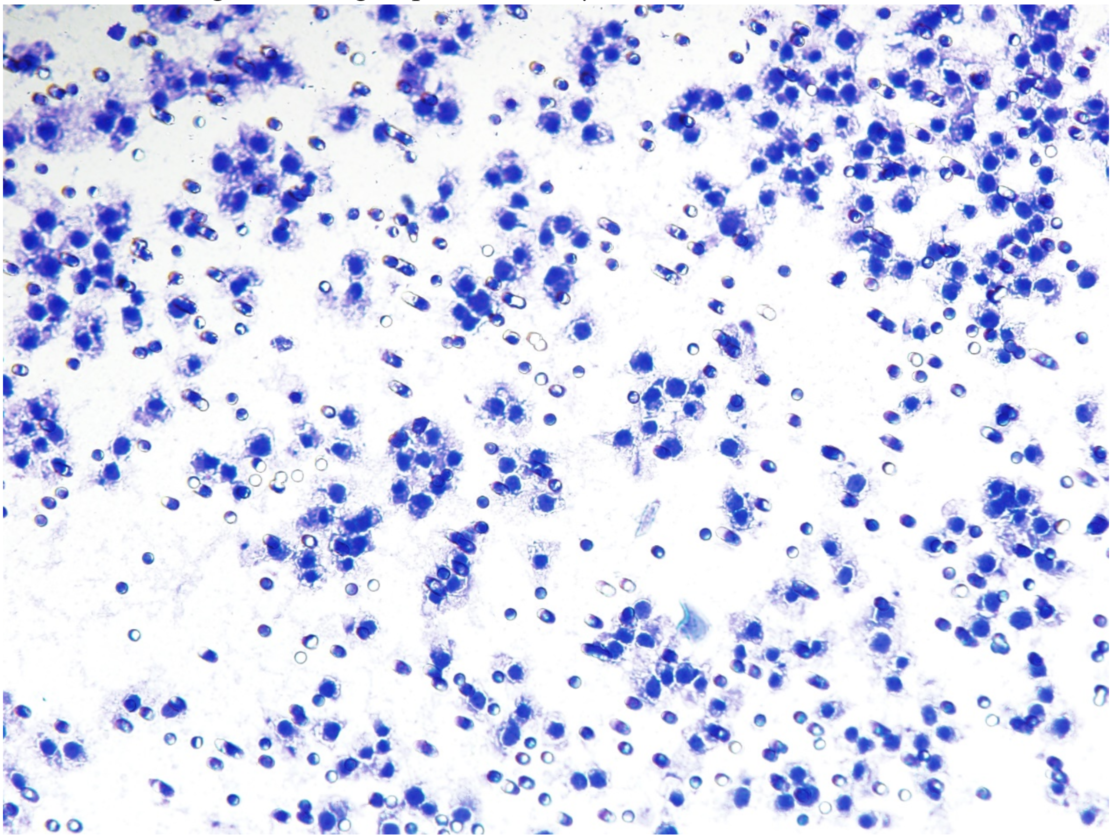

Fig.6B siTNFRSF17 group with 0 μM 25-HC in SPC-A1 cells

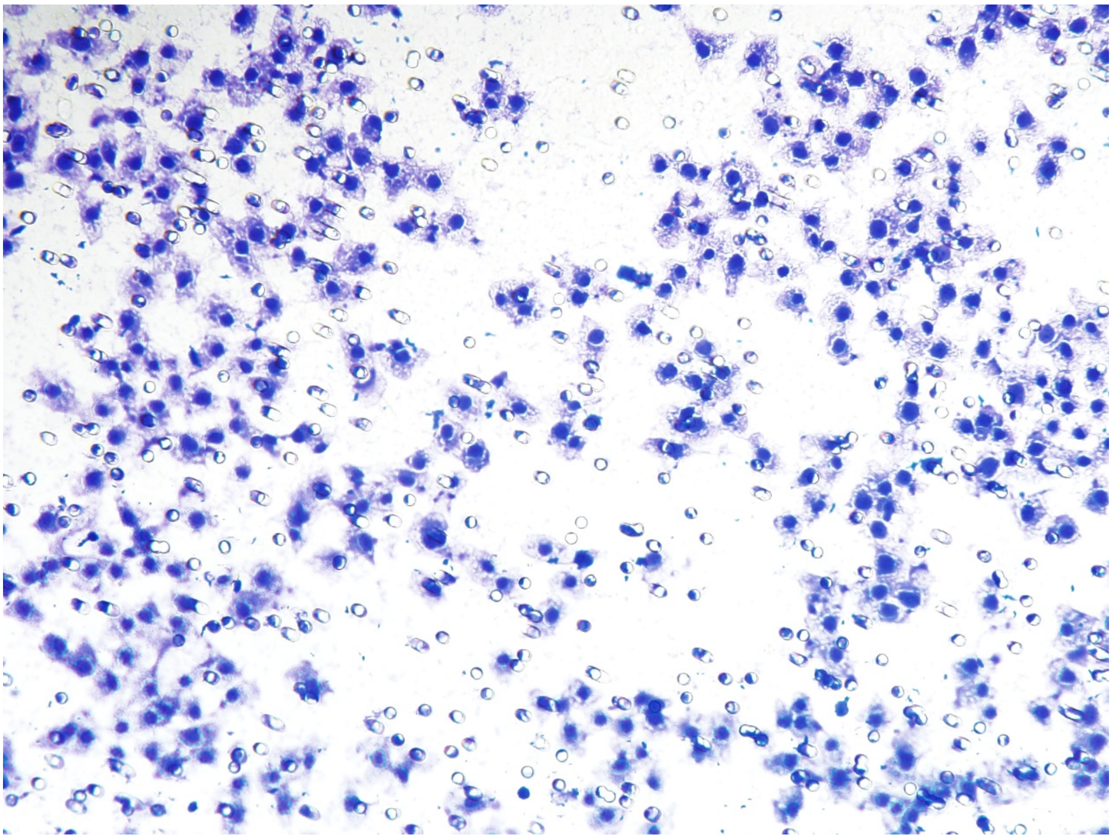

Fig.6B siTNFRSF17 group with 0.013 μM 25-HC in SPC-A1 cells

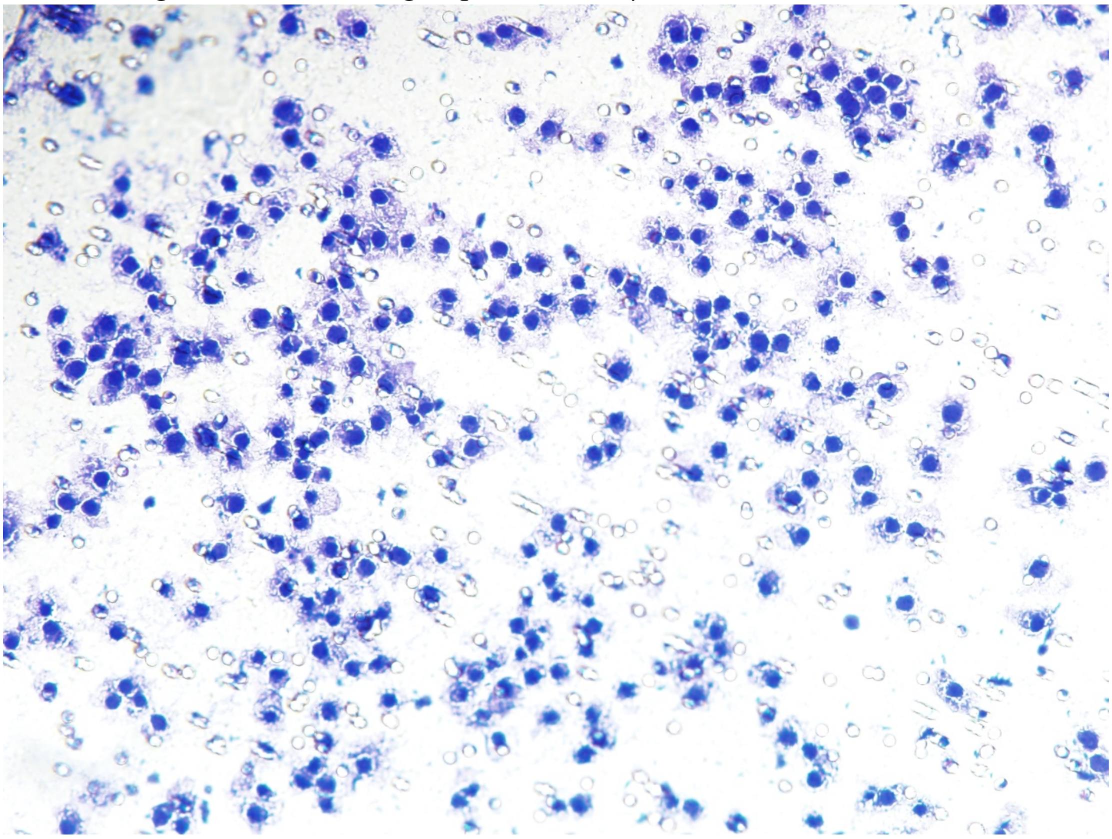

Fig.6B siTNFRSF17 group with 0.085 μM 25-HC in SPC-A1 cells

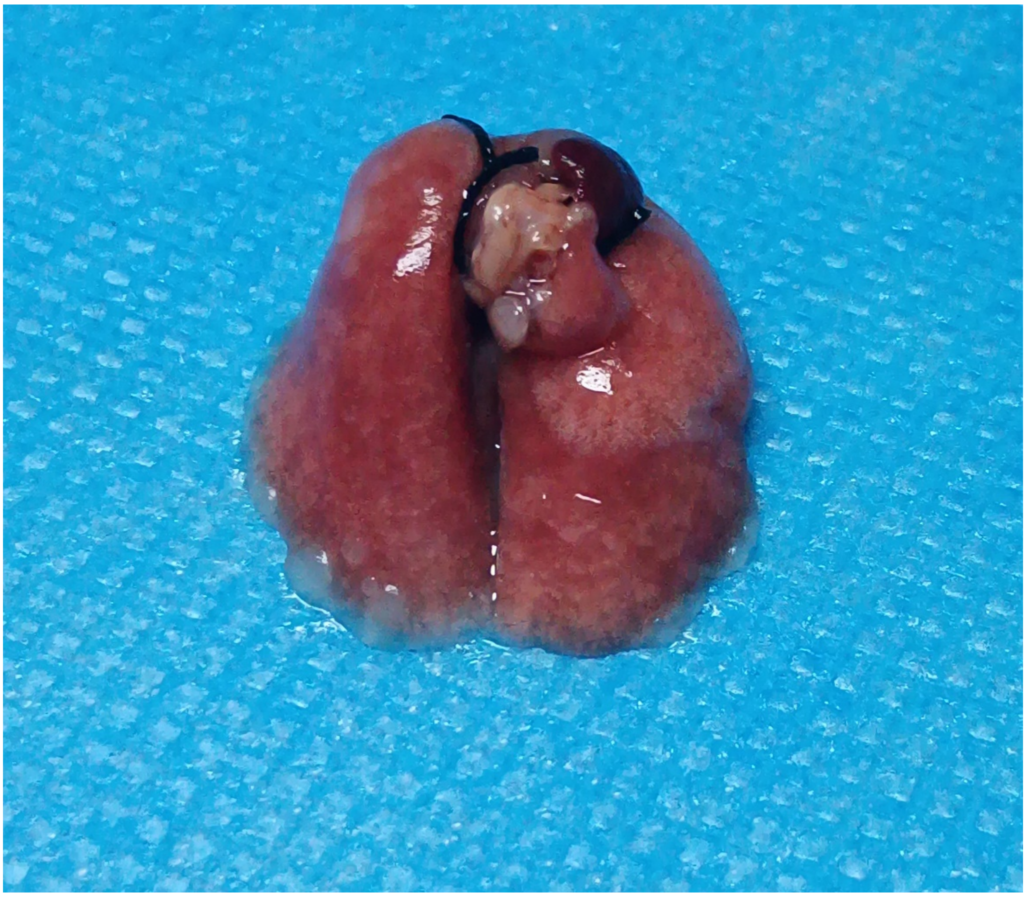

Fig 7A toumor number of sh NC group with 0 μM 25-HC

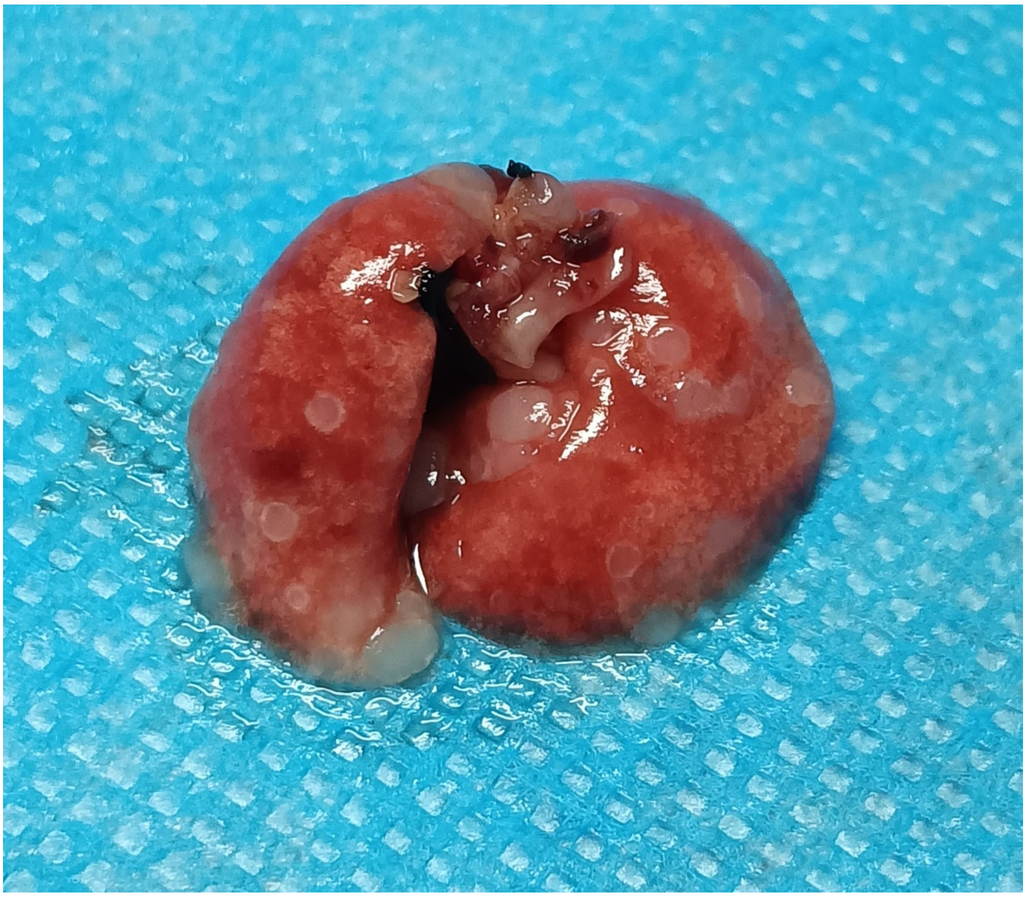

Fig 7A toumor number of sh NC group with 0.085 μM 25-HC

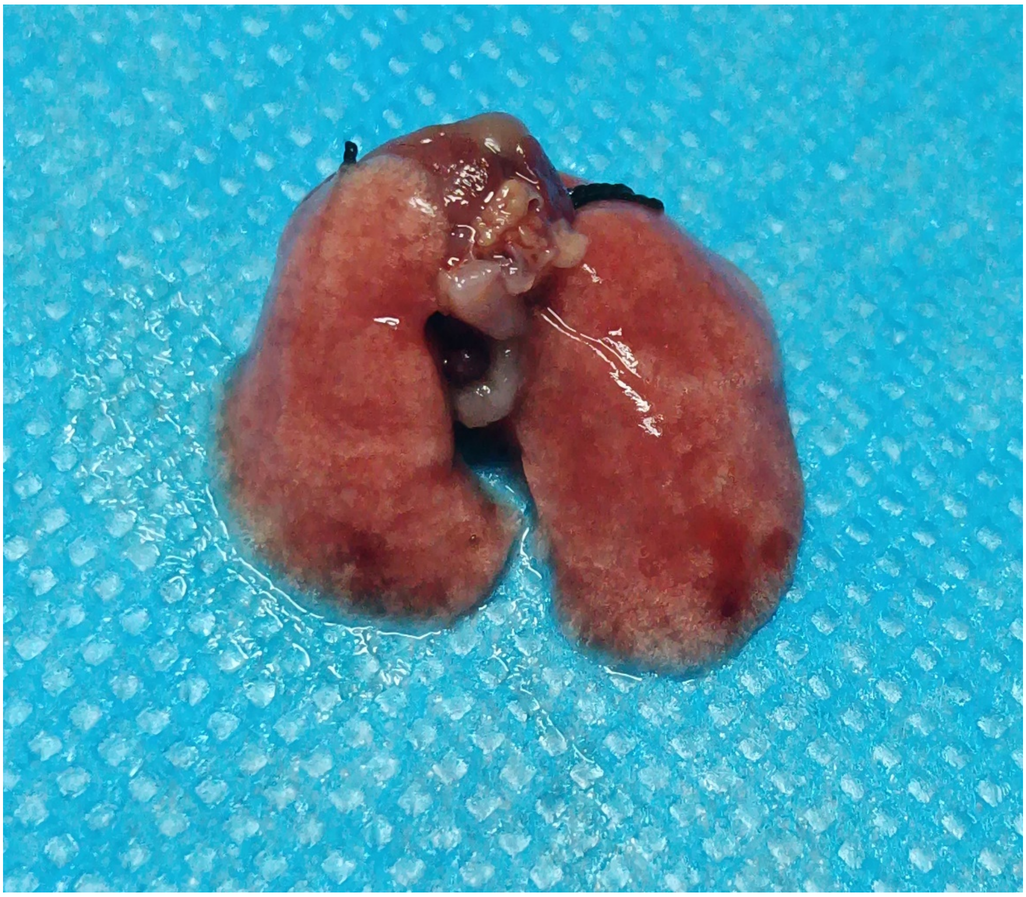

Fig 7A toumor number of sh ERβ group with 0 μM 25-HC

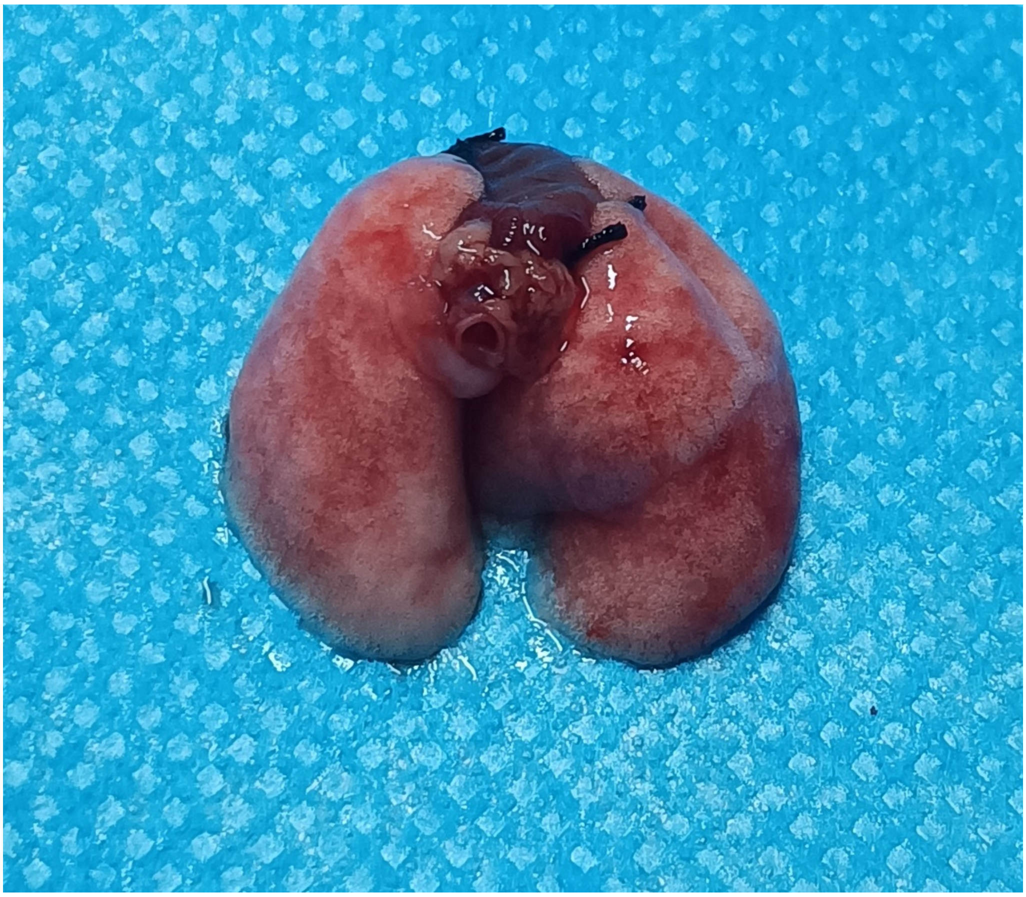

Fig 7A toumor number of sh ERβ group with 0.085 μM 25-HC

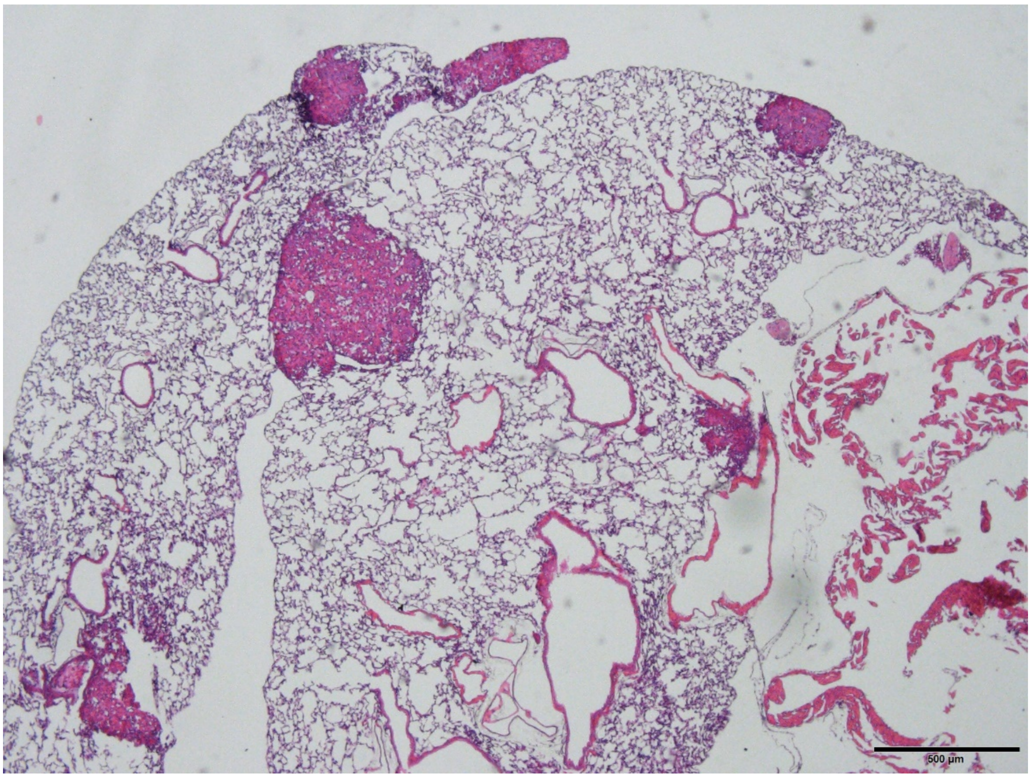

Fig 7C toumor number of sh NC group with 0 μM 25-HC

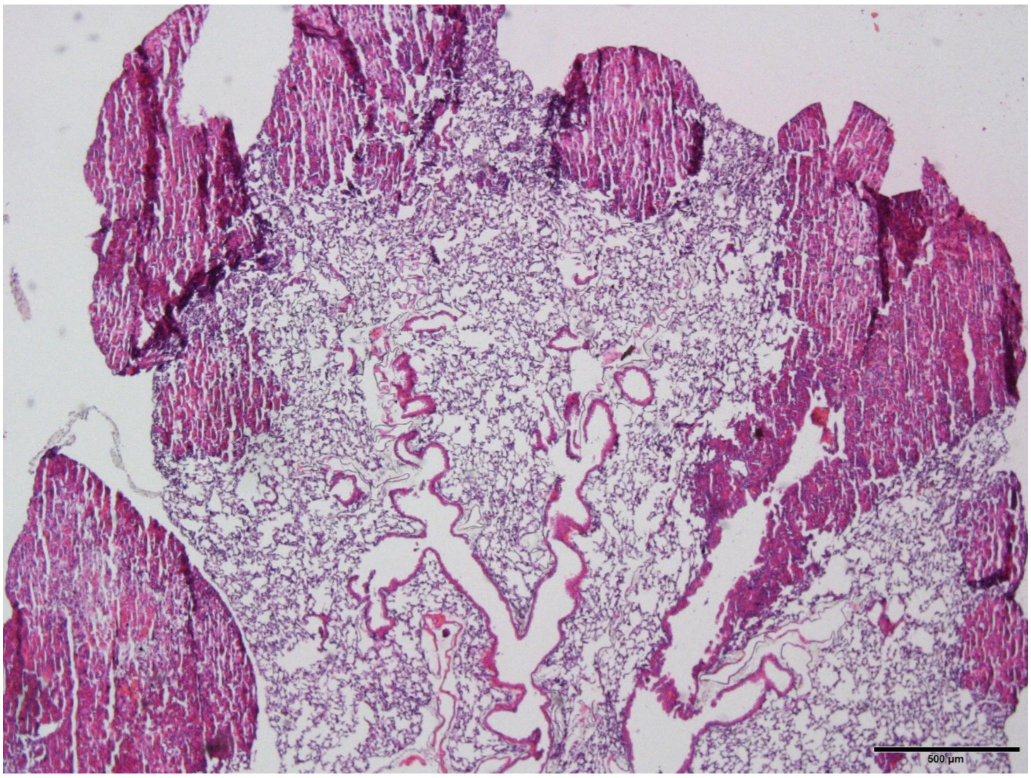

Fig 7C toumor number of sh NC group with 0.085 μM 25-HC

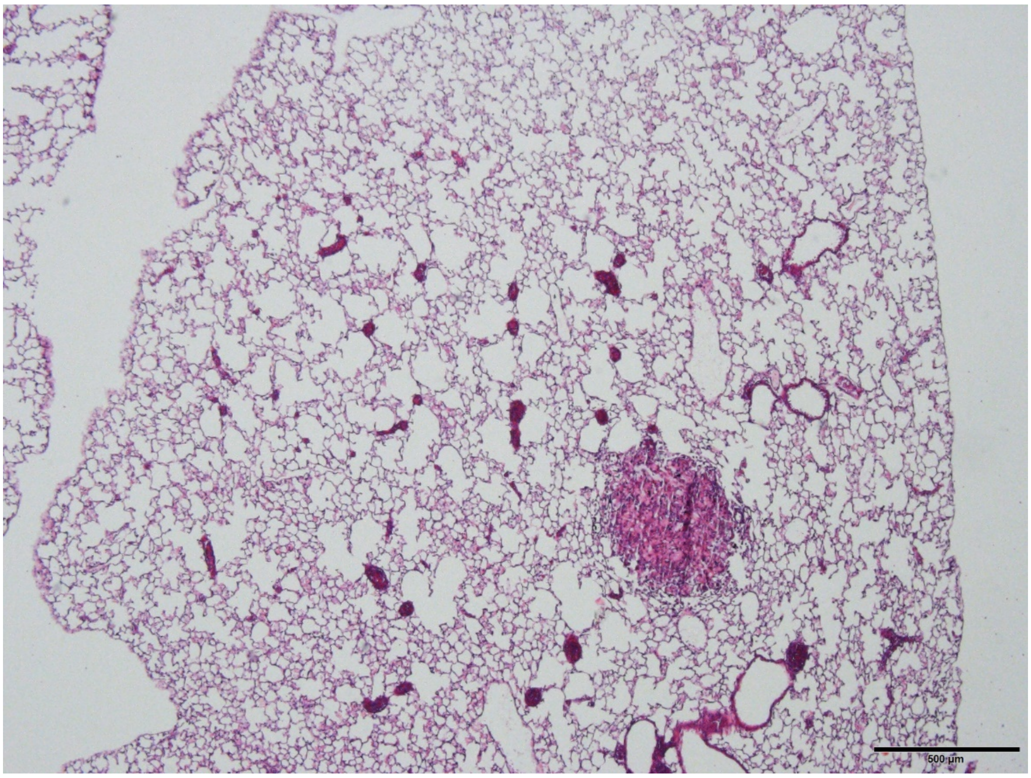

Fig 7C toumor number of sh ERβ group with 0 μM 25-HC

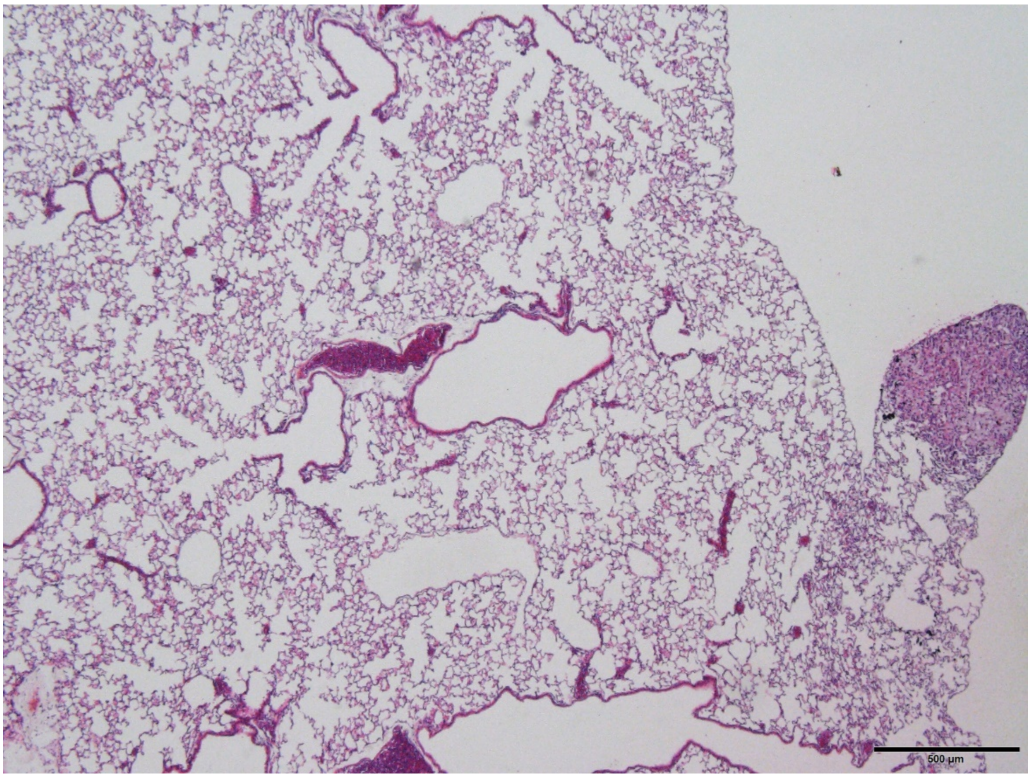

Fig 7C toumor number of sh ERβ group with 0.085 μM 25-HC

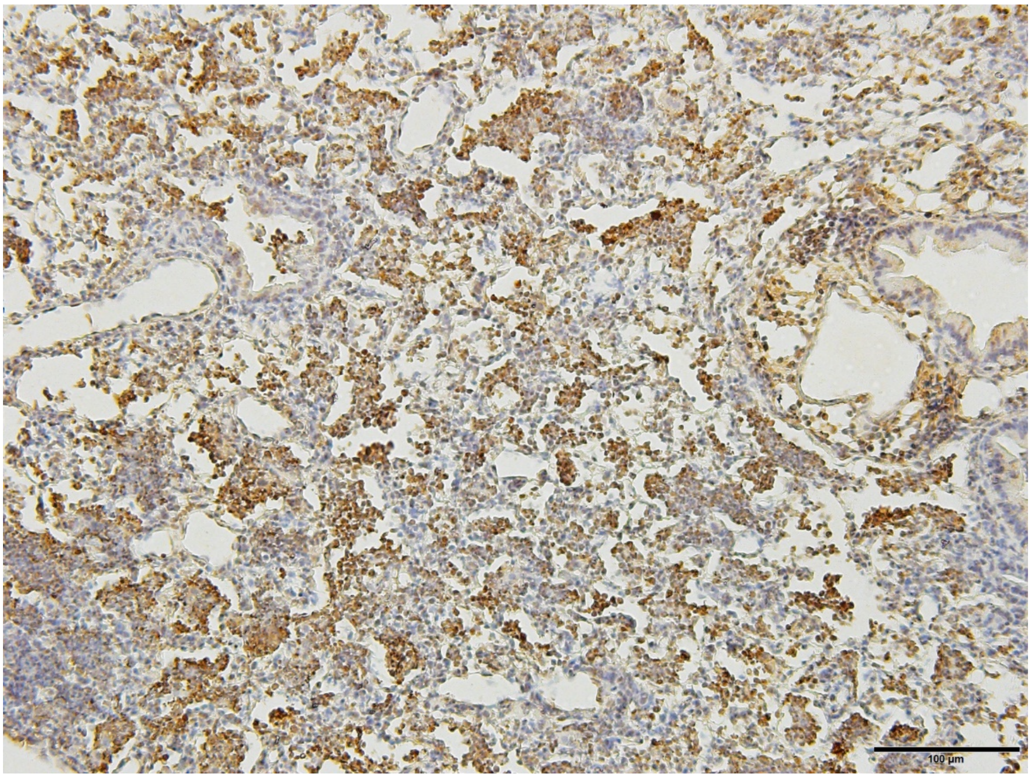

Fig.7D sh NC group with 0 μM 25-HC ERβ expression in tumor tissue

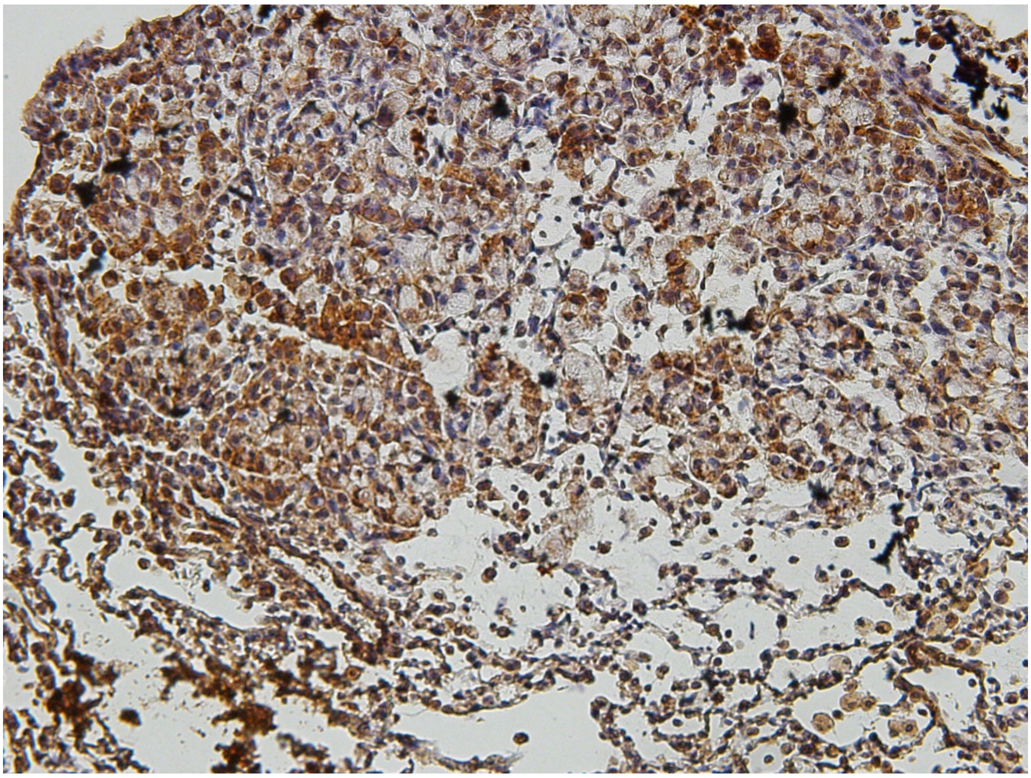

Fig.7D sh NC group with 0.085 μM 25-HC ERβ expression in tumor tissue

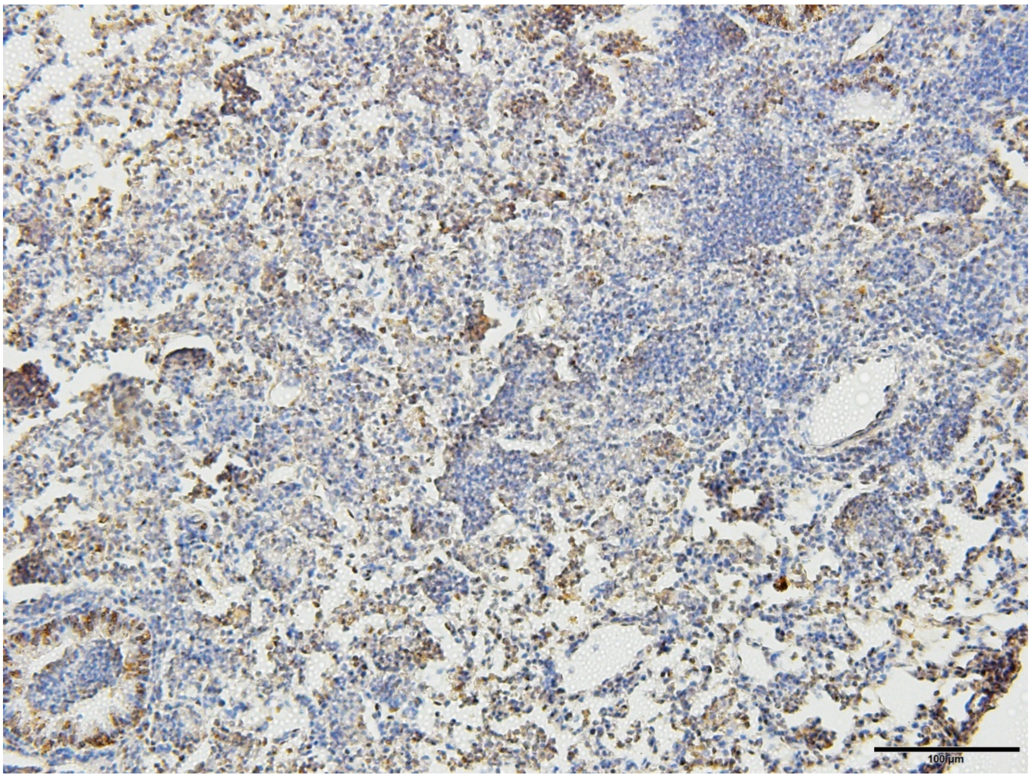

Fig.7D sh ERβ group with 0 μM 25-HC ERβ expression in tumor tissue

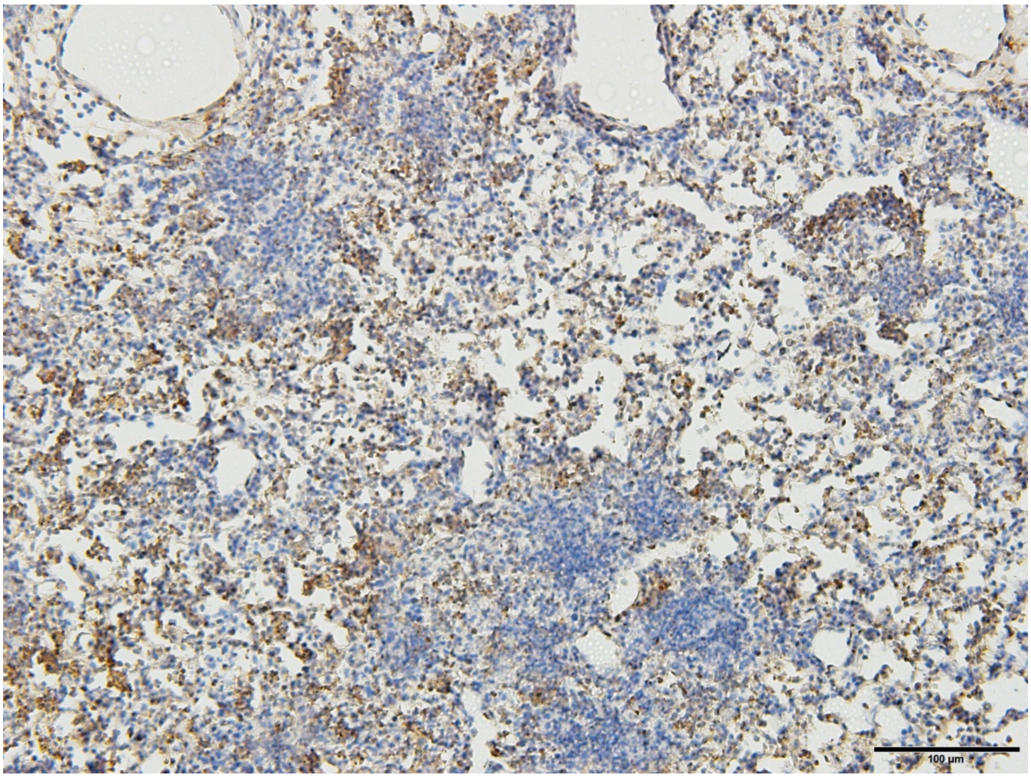

Fig.7D sh ERβ group with 0.085 μM 25-HC ERβ expression in tumor tissue

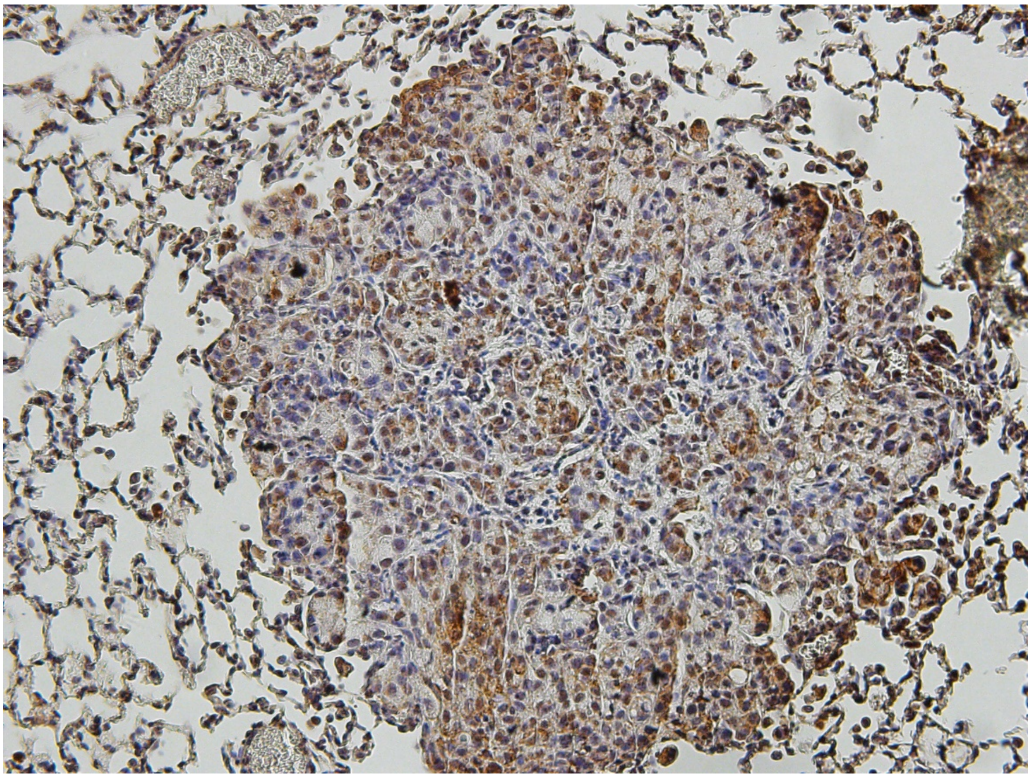

Fig.7E sh NC group with 0 μM 25-HC TNFRSF17 expression in tumor tissue

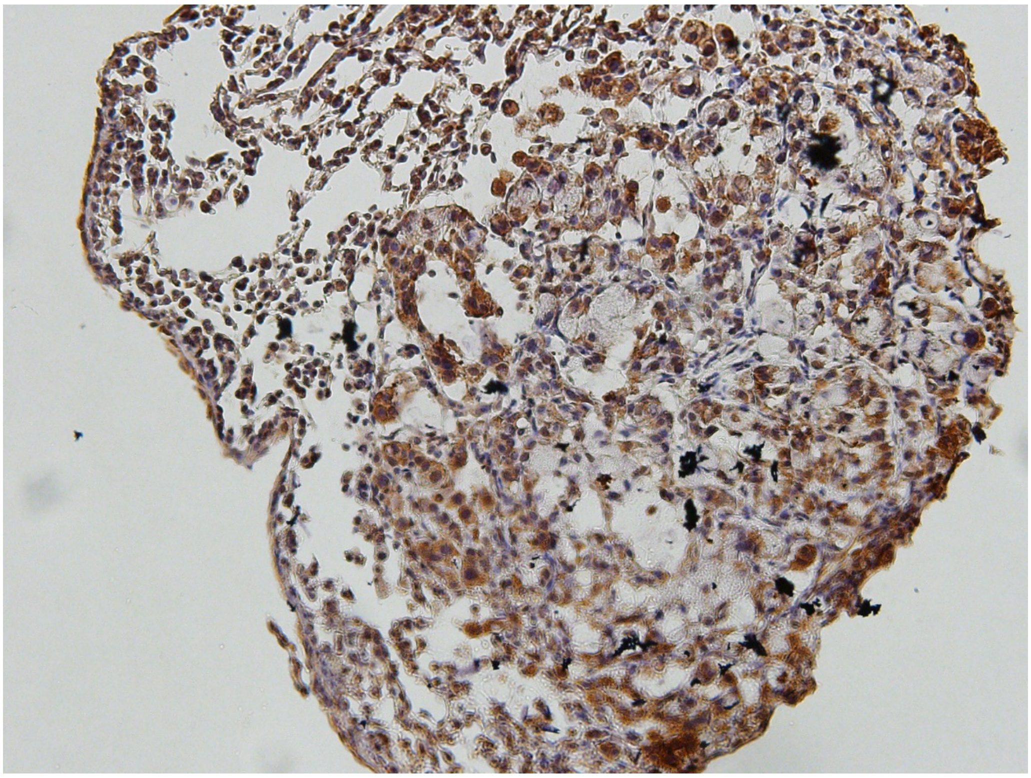

Fig.7E sh NC group with 0.085 μM 25-HC TNFRSF17 expression in tumor tissue

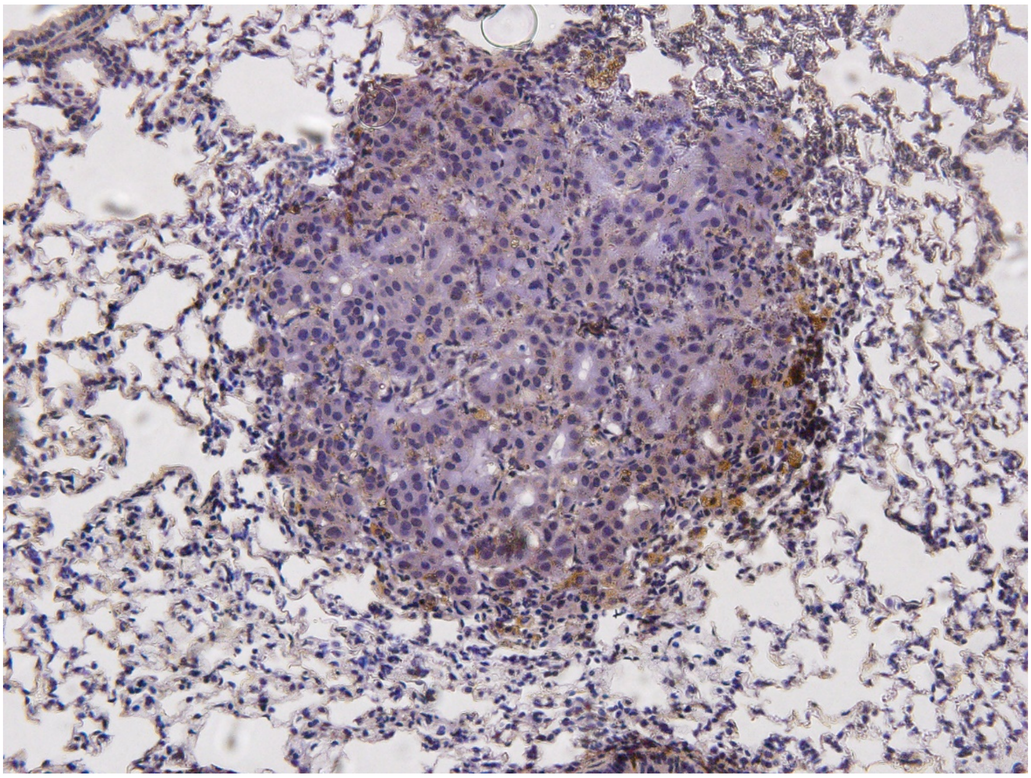

Fig.7E sh ERβ group with 0 μM 25-HC TNFRSF17 expression in tumor tissue

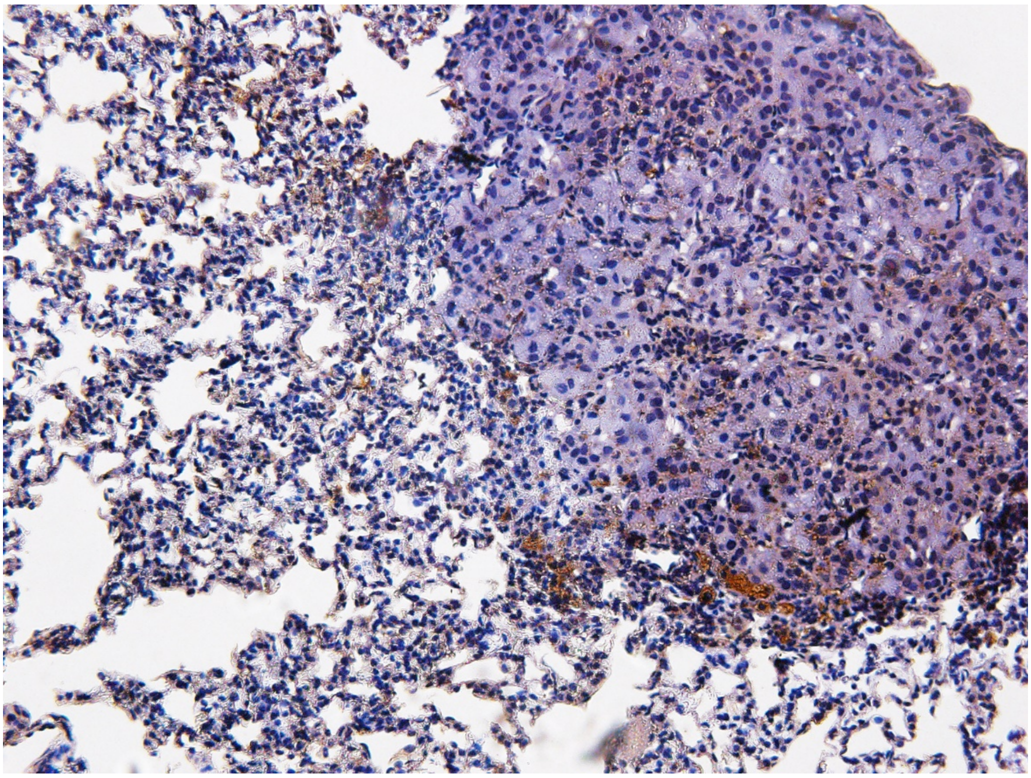

Fig.7E sh ERβ group with 0.085 μM 25-HC TNFRSF17 expression in tumor tissue
