# Supplementary material for: 25-hydroxycholesterol promotes proliferation and metastasis of lung adenocarcinoma cells by regulating ERβ/TNFRSF17 axis
Source: BMC Cancer. 2024 Apr 22;24:505. doi: 10.1186/s12885-024-12227-4 (PMC11034116; doi:10.1186/s12885-024-12227-4)
Supplement: Supplementary file 5 — Supplementary Material 5 [file 12885_2024_12227_MOESM5_ESM.pdf]

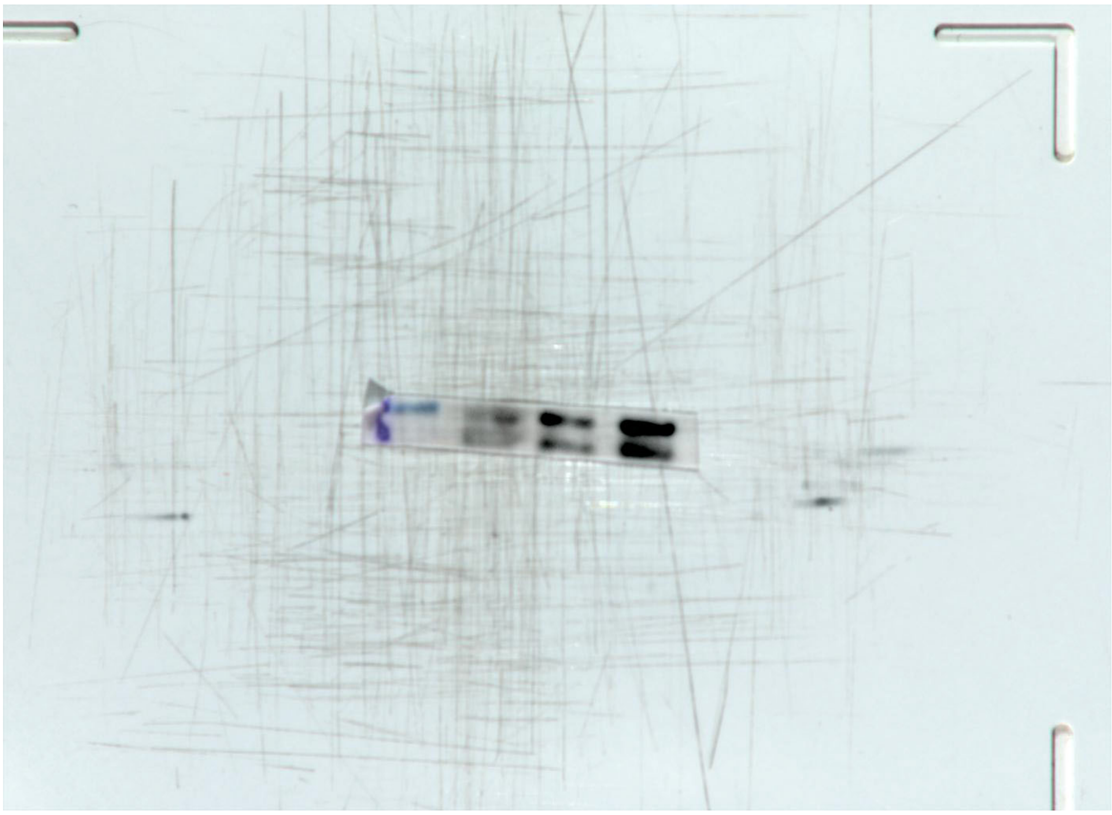

Fig.5B TNFRSF17 expression in A549 cells with siNC

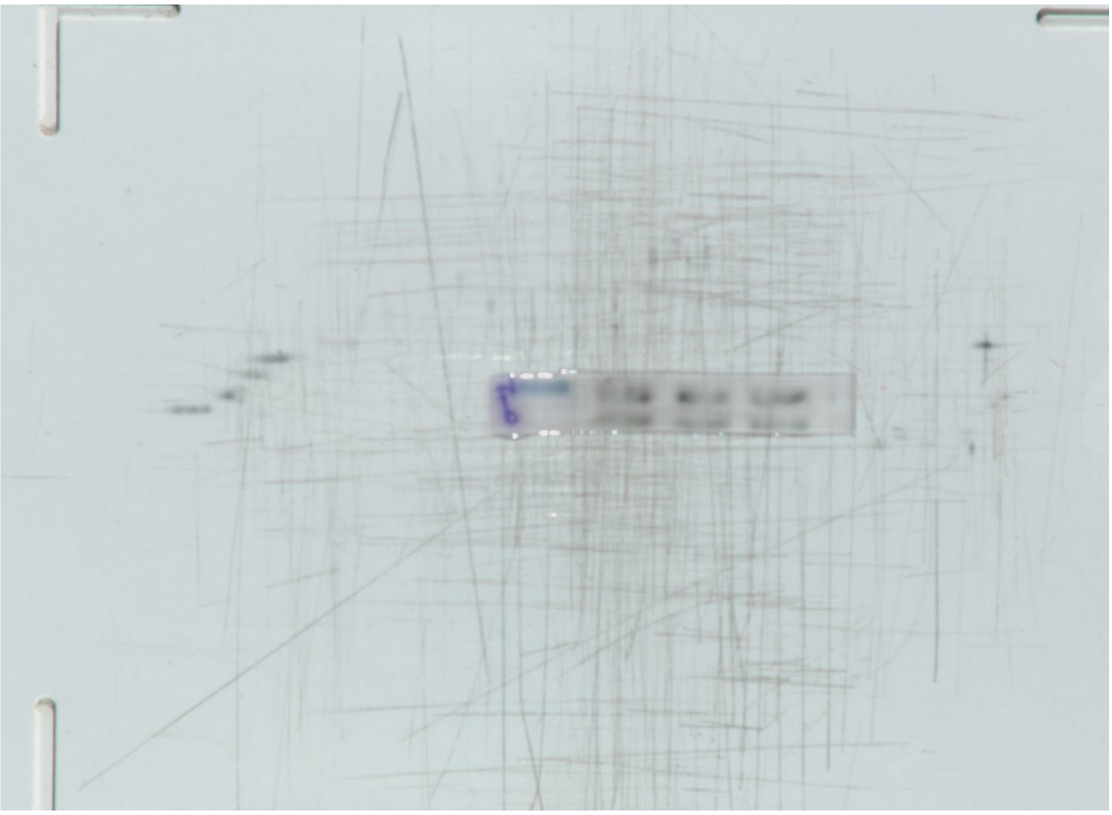

Fig.5B TNFRSF17 expression in A549 cells with siTNFRSF17

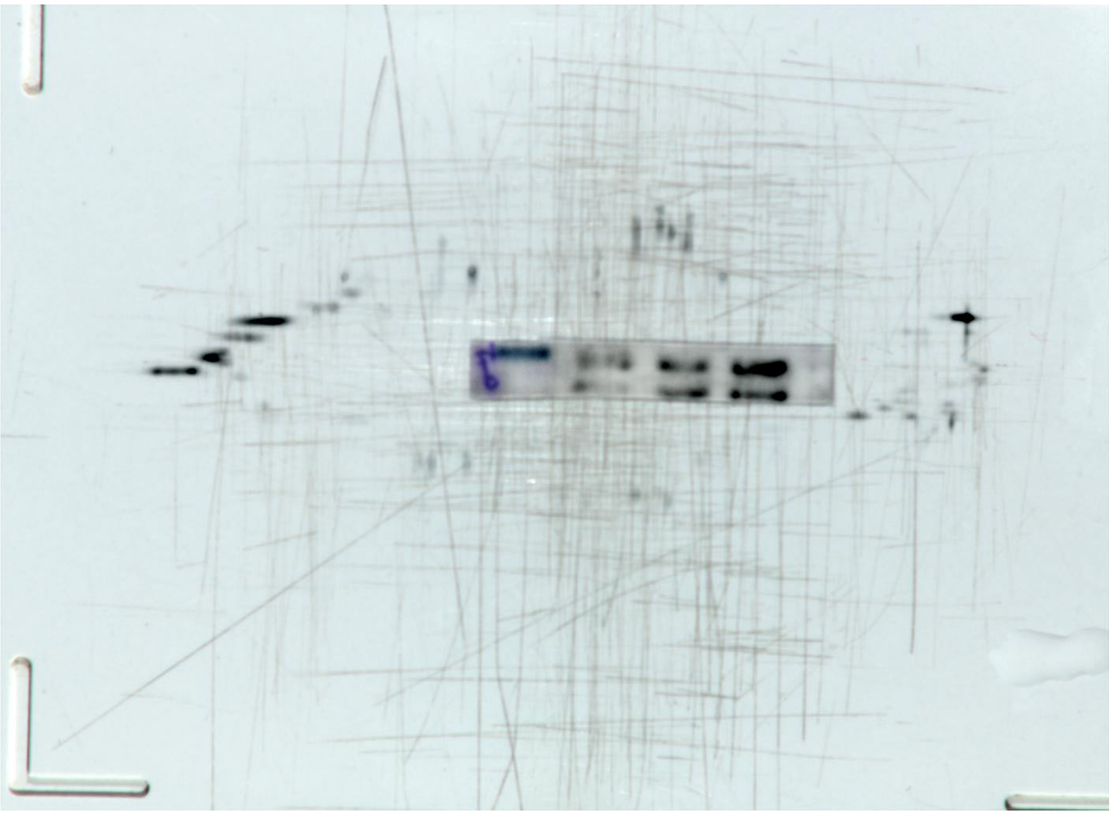

Fig.5B TNFRSF17 expression in SPC-A1 cells with siNC

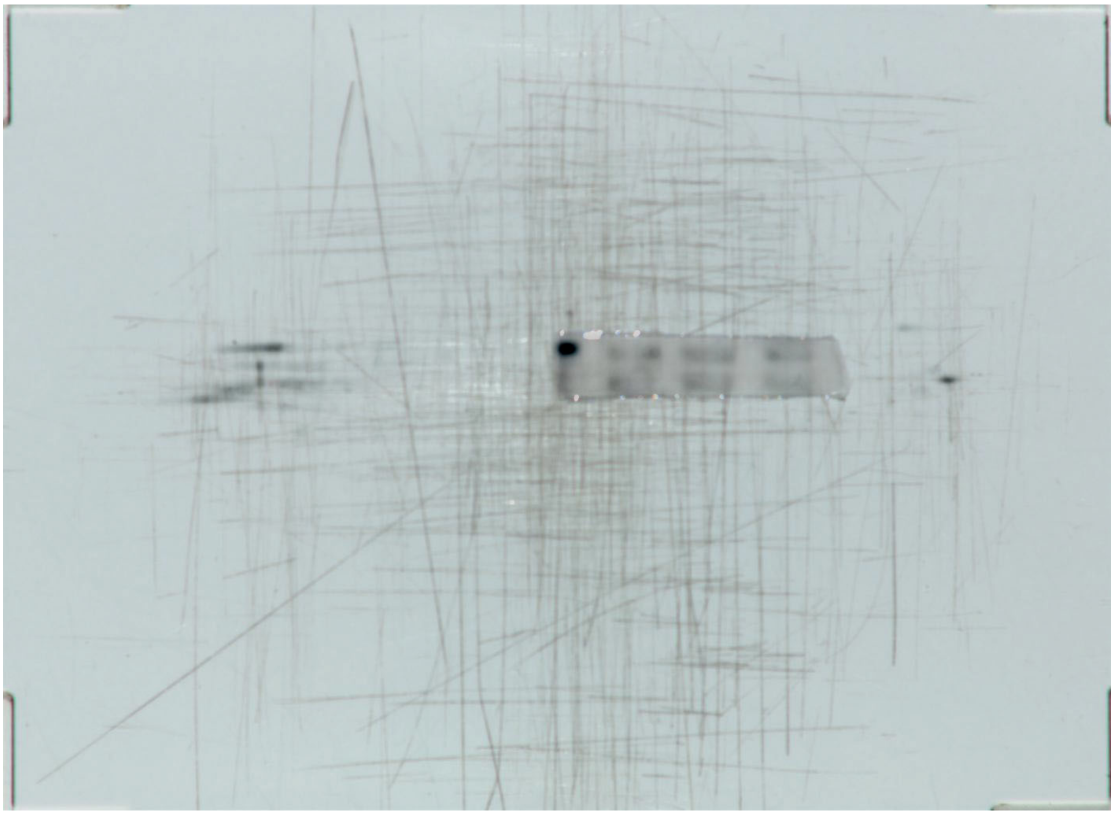

Fig.5B TNFRSF17 expression in SPC-A1 cells with siTNFRSF17

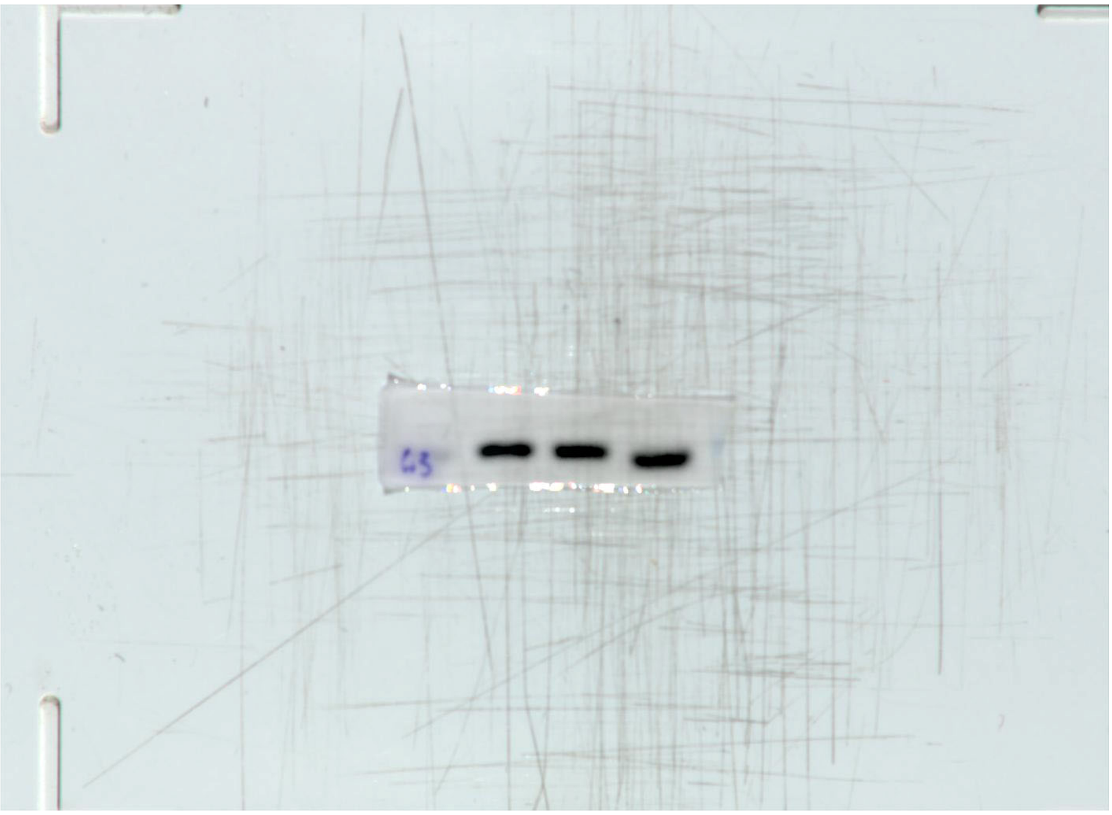

Fig.5B GAPDH expression in A549 cells with siNC

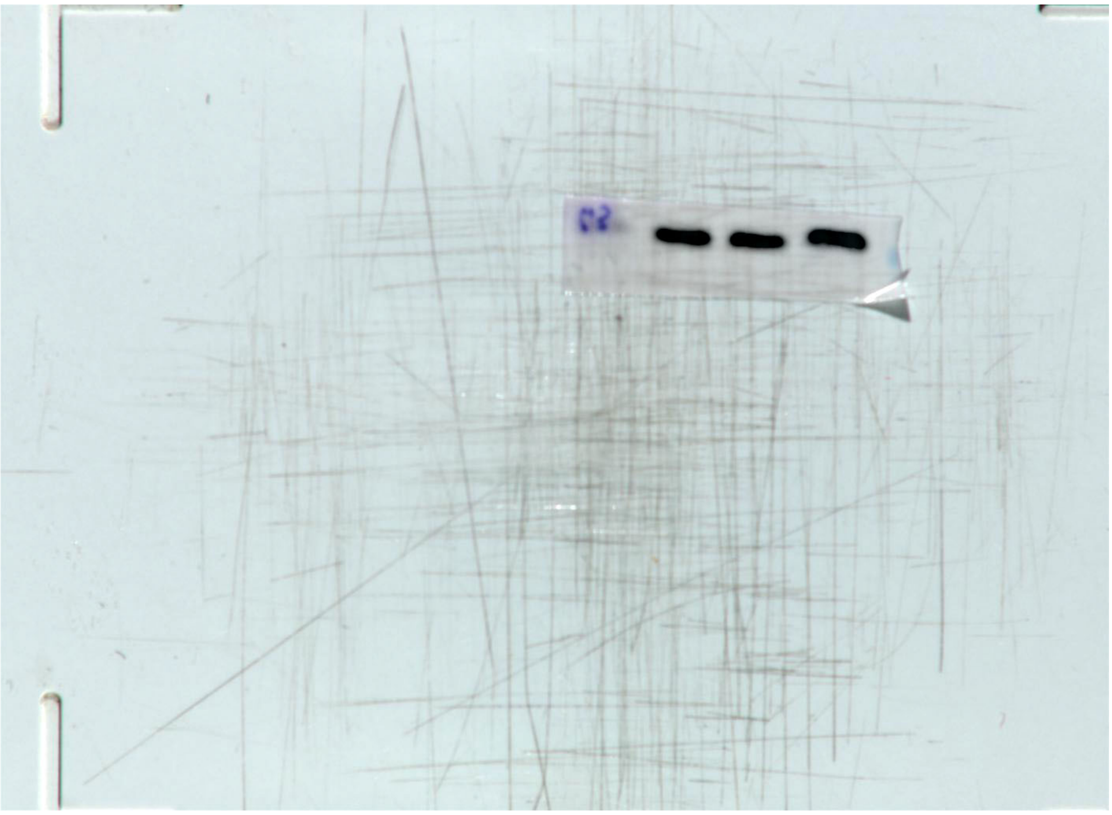

Fig.5B GAPDH expression in A549 cells with siTNFRSF17

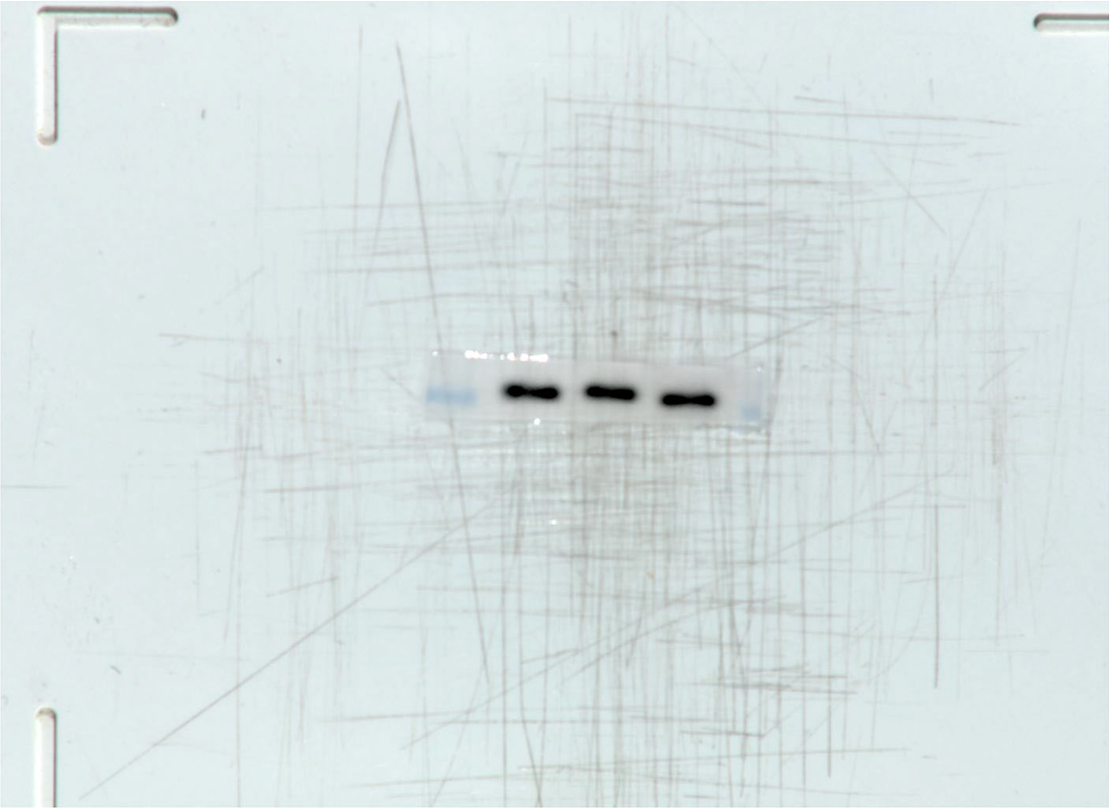

Fig.5B GAPDH expression in SPC-A1 cells with siNC

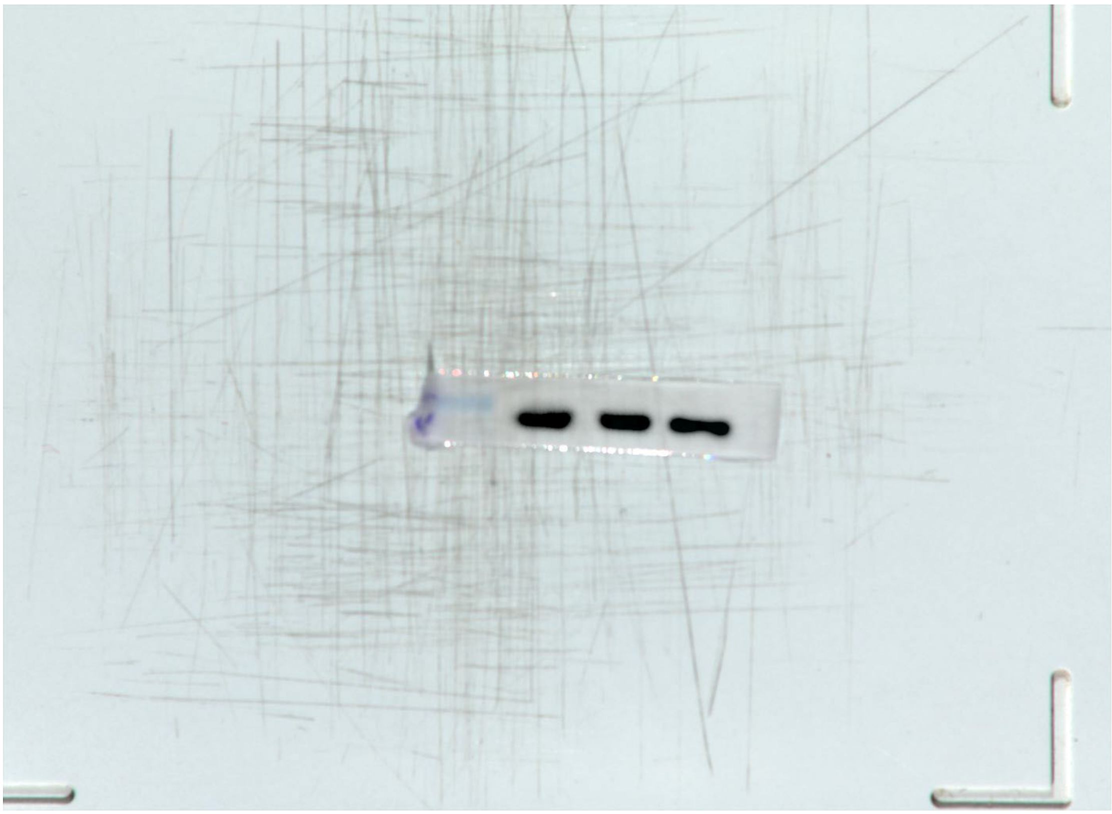

Fig.5B GAPDH expression in SPC-A1 cells with siTNFRSF17

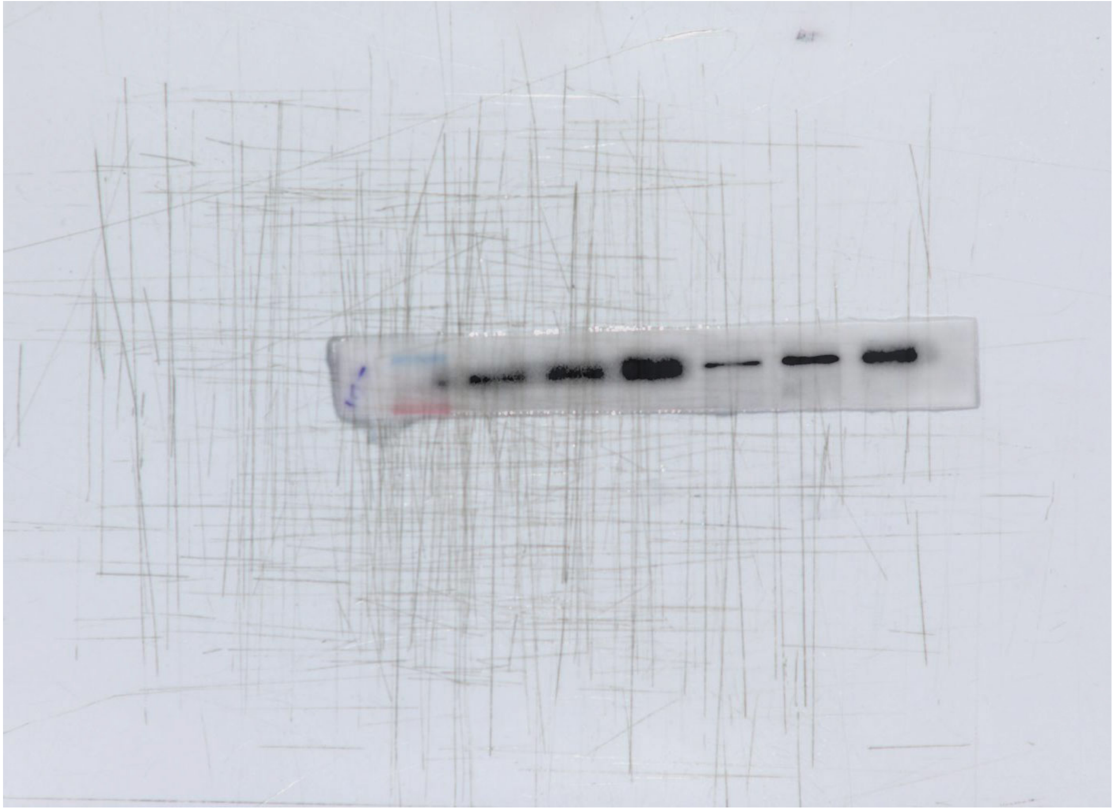

Fig.2s pAKT expression

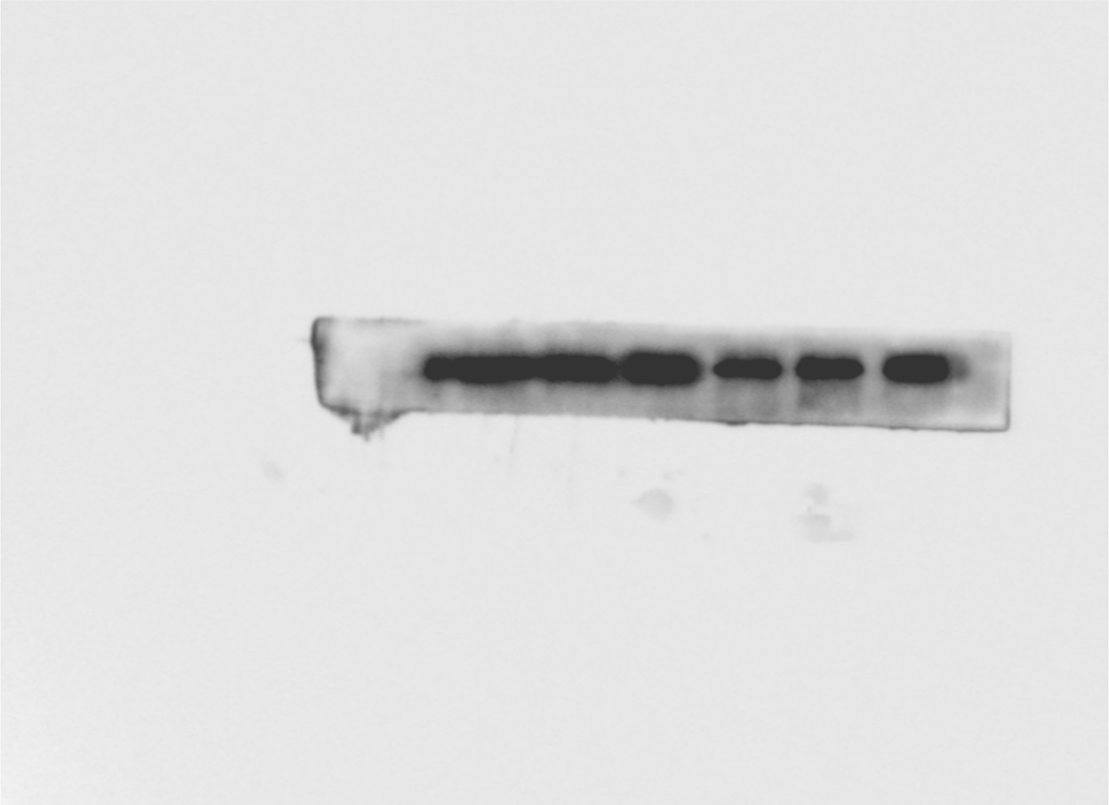

Fig.2s AKT expression

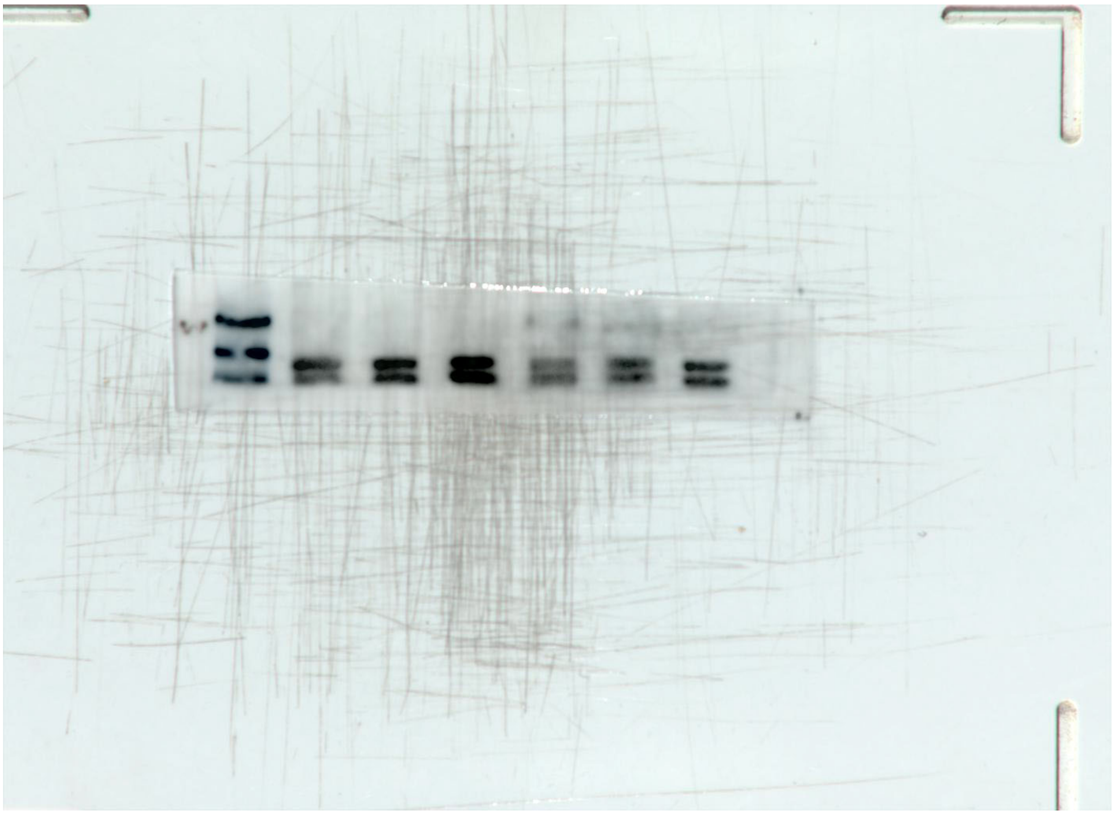

Fig.2s pERK expression

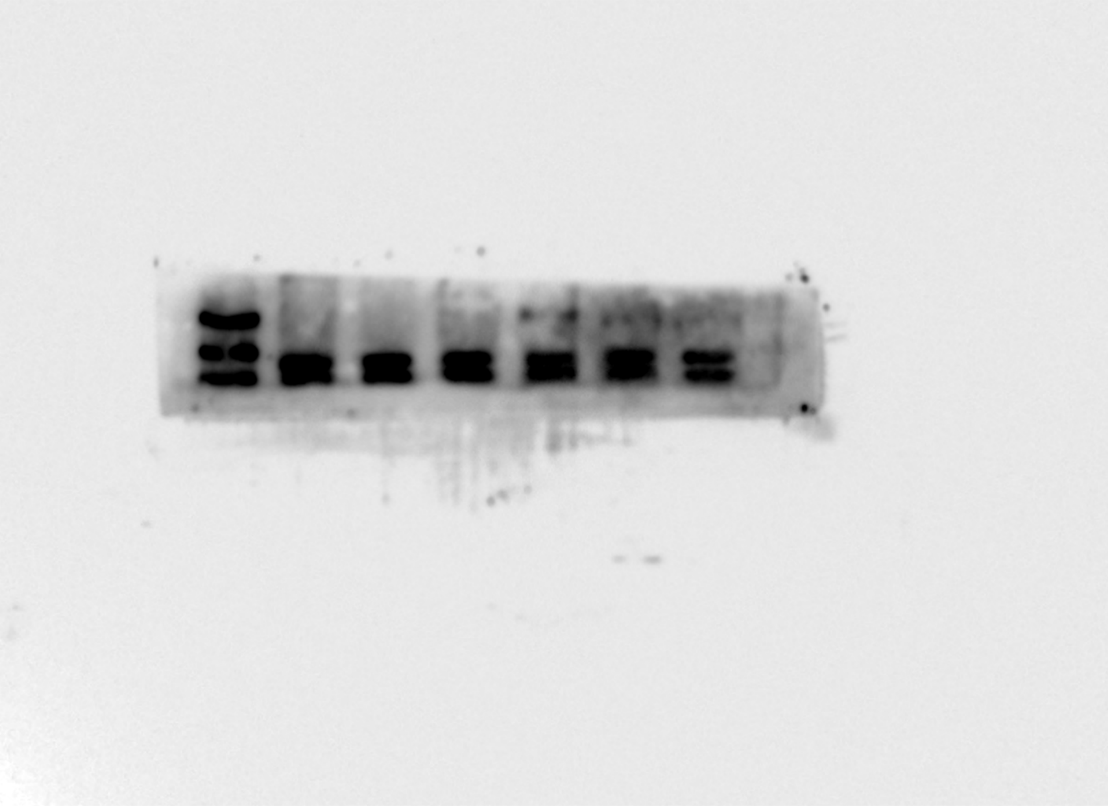

Fig.2s ERK expression

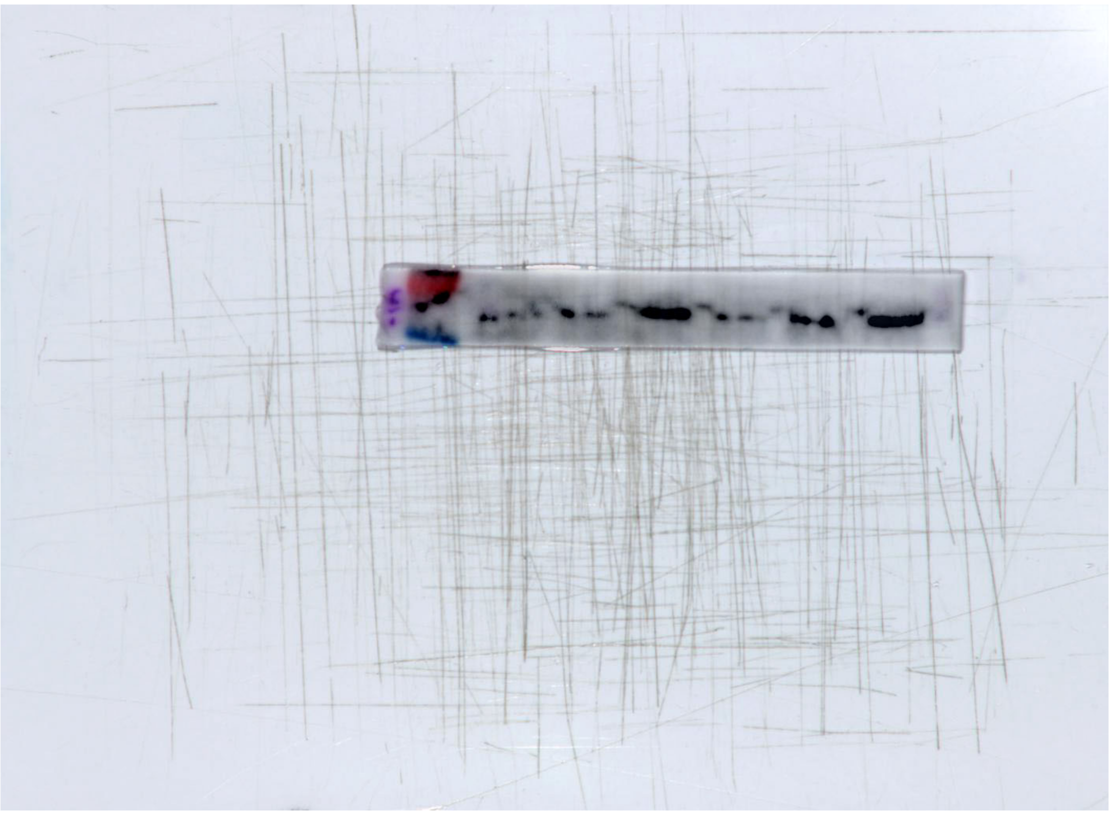

Fig.2s Vimentin expression

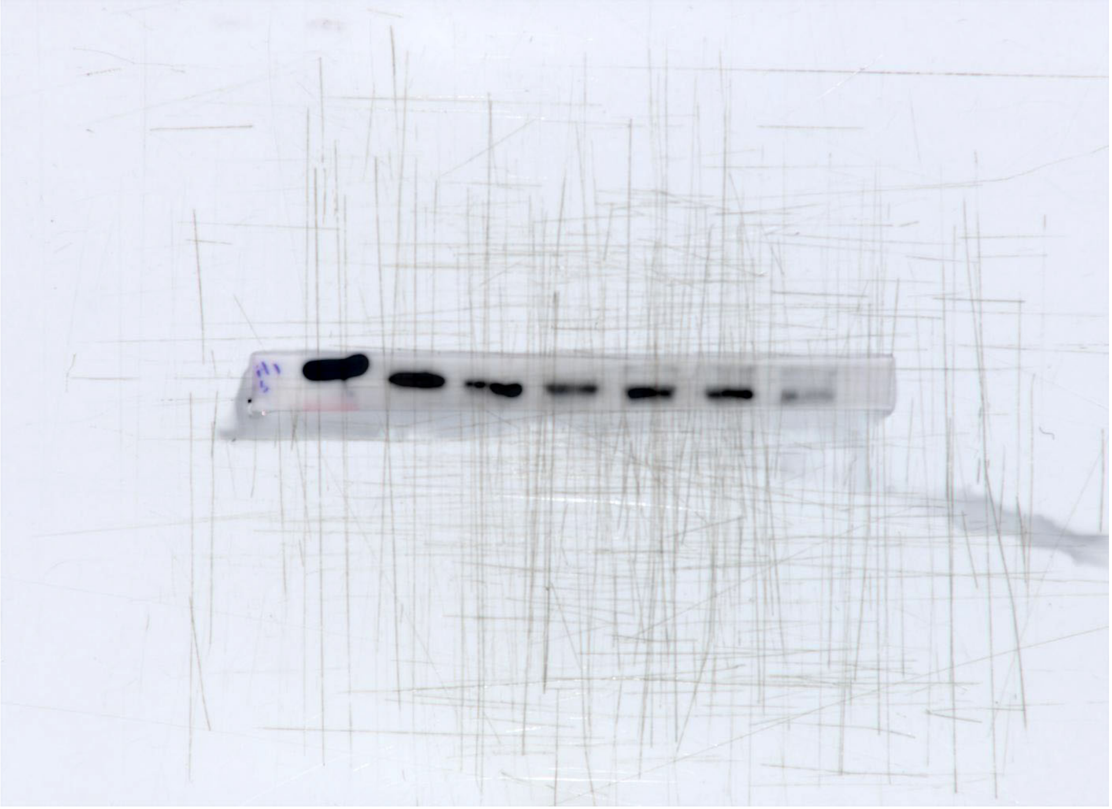

Fig.2s E-Cadherin expression
